# Sequence and Structural Motifs Controlling the Broad Substrate Specificity of the Mycobacterial Hormone-Sensitive Lipase LipN

**DOI:** 10.1021/acsomega.3c00534

**Published:** 2023-03-30

**Authors:** Daniel E. Schemenauer, Emily H. Pool, Stephanie N. Raynor, Gabriela P. Ruiz, Leah M. Goehring, Andrew J. Koelper, Madeleine A. Wilson, Anthony J. Durand, Elexi C. Kourtoglou, Erik M. Larsen, Luke D. Lavis, John J. Esteb, Geoffrey C. Hoops, R. Jeremy Johnson

**Affiliations:** † Department of Chemistry and Biochemistry, Butler University, Indianapolis, Indiana 46208, United States; ‡ Howard Hughes Medical Institute, Janelia Research Campus, Ashburn, Virginia 20147, United States

## Abstract

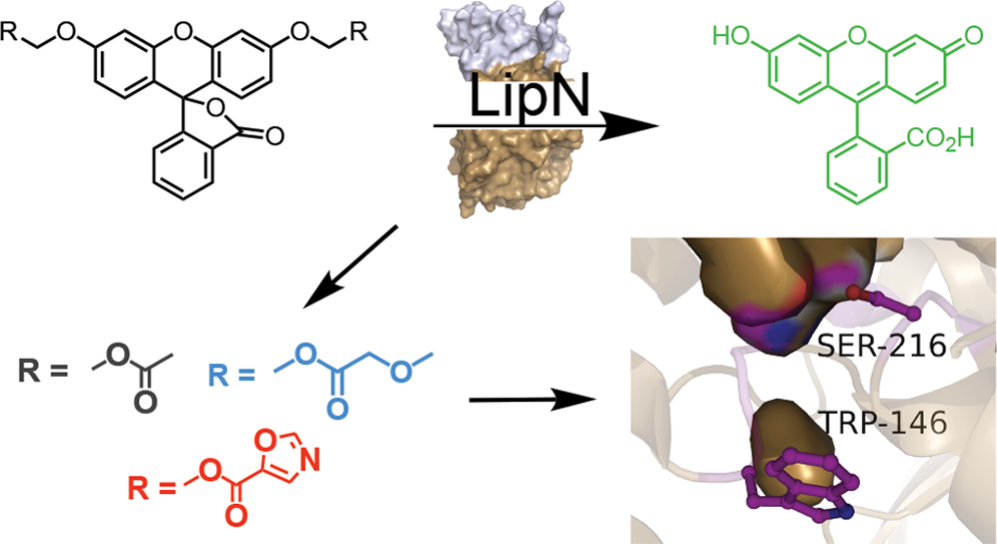

*Mycobacterium tuberculosis* has a complex life cycle transitioning between active and dormant growth states depending on environmental conditions. LipN (Rv2970c) is a conserved mycobacterial serine hydrolase with regulated catalytic activity at the interface between active and dormant growth conditions. LipN also catalyzes the xenobiotic degradation of a tertiary ester substrate and contains multiple conserved motifs connected with the ability to catalyze the hydrolysis of difficult tertiary ester substrates. Herein, we expanded a library of fluorogenic ester substrates to include more tertiary and constrained esters and screened 33 fluorogenic substrates for activation by LipN, identifying its unique substrate signature. LipN preferred short, unbranched ester substrates, but had its second highest activity against a heteroaromatic five-membered oxazole ester. Oxazole esters are present in multiple mycobacterial serine hydrolase inhibitors but have not been tested widely as ester substrates. Combined structural modeling, kinetic measurements, and substitutional analysis of LipN showcased a fairly rigid binding pocket preorganized for catalysis of short ester substrates. Substitution of diverse amino acids across the binding pocket significantly impacted the folded stability and catalytic activity of LipN with two conserved motifs (HGGGW and GDSAG) playing interconnected, multidimensional roles in regulating its substrate specificity. Together this detailed substrate specificity profile of LipN illustrates the complex interplay between structure and function in mycobacterial hormone-sensitive lipase homologues and indicates oxazole esters as promising inhibitor and substrate scaffolds for mycobacterial hydrolases.

## Introduction

Tuberculosis caused by infection from the bacterium *Mycobacterium tuberculosis* is one of the deadliest infectious diseases worldwide. Complicating the treatment and eradication of *M. tuberculosis* is its multi-tiered life cycle where it can transition between active and dormant growth states depending on environmental conditions.^1^ Serine hydrolases are one class of enzymes with a confirmed clinical role in this transition between active and dormant growth and in survival during dormant infection.^2−5^ Serine hydrolases are a diverse class of enzymes catalyzing the hydrolysis of hydrophobic lipid and polar metabolic ester, amide, and thioester substrates using a conserved catalytic mechanism and core α/β hydrolase protein fold.^6,7^ Multiple activity-based protein profiling (ABPP) studies have reaffirmed the oscillation of mycobacterial serine hydrolase activity based on *M. tuberculosis* growth conditions including nutrient starvation, hypoxia, and acidic stress.^4,8−11^ Changes in hydrolase activity also correlated with the utilization of intracellular lipid stores for maintaining dormant growth.^12−14^

LipN (Rv2970c) is a conserved mycobacterial serine hydrolase with diverse data demonstrating an essential role in mycobacterial growth and survival.^13,15,16^ LipN was one of only five mycobacterial serine hydrolases active under dormant growth conditions and remained active under hypoxic, recovery, and high nutrient growth conditions.^9−11,16−18^ LipN is phosphorylated on its catalytic serine and acetylated on various lysines, indicating post-translational regulation of its enzymatic activity.^11,19,20^ LipN was also the highest biomarker for serological detection of active TB infection and was correlated with the development of multiple drug-resistant states.^21,22^ Together these results paint LipN as a key metabolic serine hydrolase for *M. tuberculosis* with regulated catalytic activity at the interface between active and dormant growth conditions. Previous characterization of LipN showed activity for short (∼4 carbon) ester substrates, inhibition by a wide range of general covalent modifiers, and a classic catalytic triad of serine, histidine, and aspartate.^15^ Sequence and motif analysis also placed LipN firmly within the bacterial hormone-sensitive lipase (bHSL) superfamily with high sequence conservation with the C-terminal catalytic domain of human HSL.^15^

bHSLs are prominently expressed across bacterial species from mesophilic, thermophilic, and psychrophilic organisms.^23^ The relative stability, substrate promiscuity, and adaptability of bHSLs have found synthetic, biotechnological, and agricultural applications.^23^ Common features across bHSLs are a core α/β hydrolase domain with conserved GxSxG and HGGG motifs containing the catalytic serine and oxyanion hole, respectively. The majority of bHSLs, including LipN, are classified into family IV of 15 bacterial esterase families.^13,15^ Family IV members are further subdivided based on residues surrounding the conserved catalytic motifs, where distinct motifs impart unique catalytic activity and substrate selectivity to individual bHSLs.^23−25^ For example, the presence of a tryptophan residue following the HGGG motif (HGGGW) is correlated with a strong preference for shorter ester substrates and the ability to catalyze difficult hydrolysis reactions with tertiary ester substrates.^23,26−28^ Substrate preferences of bHSLs are also controlled by a variable N-terminal lid or cap domain whose features control substrate access, protein stability, and even promiscuous acyltransferase activity.^29−31^ Unlike classic lipases, this N-terminal cap domain in bHSLs does not undergo significant structural changes or introduce latency into the kinetic mechanism of bHSLs.^15,23^

One desirable feature of some bHSLs is their ability to catalyze the difficult hydrolysis of tertiary and constrained esters.^28,32^ Preliminary evidence indicated that LipN might have expanded tertiary ester activity as it catalyzed the xenobiotic degradation of the tertiary substrate 4-hydroxyphenyl acetate and contains bHSL motifs (HGGGW and GDSAG) that were connected with bHSLs ability to catalyze tertiary ester hydrolysis.^15^ Since some tertiary esters are orthogonal or only weakly activated by mammalian serine hydrolases,^33−35^ tertiary ester hydrolysis by LipN could have applications in TB-specific prodrug activation or therapeutic detection,^36^ especially as LipN is catalytically active under dormant growth conditions and is a confirmed biomarker for the serological detection of active TB.^11,21^ Herein, we expanded a library of fluorogenic ester substrates to include more tertiary and constrained esters and screened these 33 fluorogenic substrates for activation by LipN, identifying its unique substrate signature. Combined substitutional analysis confirmed the essential roles of the signature bHSL motifs and broader substrate binding pocket residues in regulating the catalytic activity and thermal stability of LipN. Structural modeling with focused substitutions allowed us to identify essential features for maintaining the substrate preferences of LipN.

## Results

### Substrate Library for LipN

**Figure 1:**
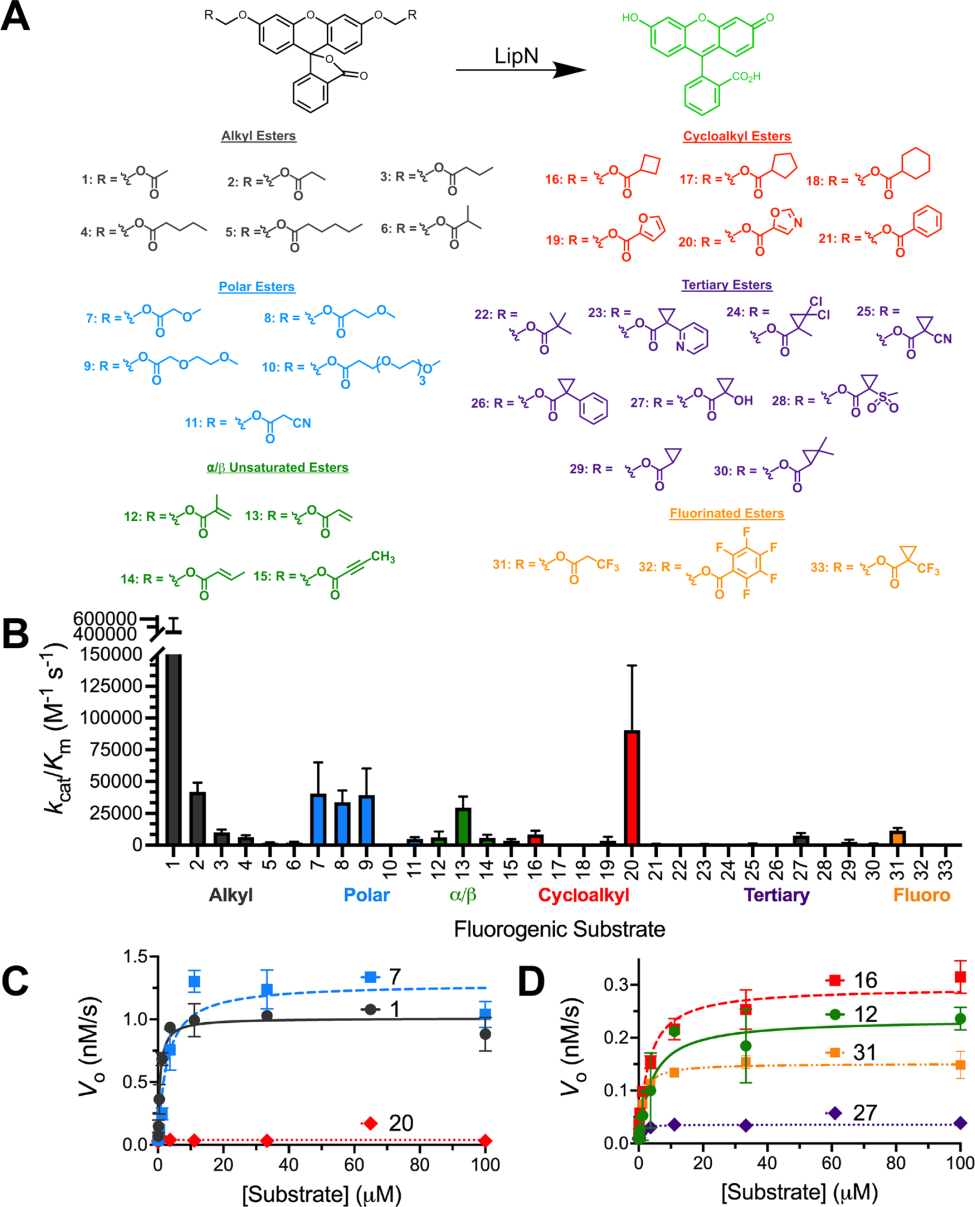
Steady-state kinetic analysis of *Mm*LipN. (A) Activation of fluorogenic ester substrates by *Mm*LipN. Each of the substrates is composed of diacyloxymethyl ether fluorescein with varying R-groups. The differing R-groups have been organized into classes and colored based on chemical functionality. All of the substrates were synthesized as described previously.^38,43^ Substrates **6**, **10**–**11**, **14**–**15**, **20, 23**–**30**, and **33** were newly synthesized for this characterization of *Mm*LipN. Detailed synthetic characterization is provided in the Supporting Information. (B) Global comparison of the catalytic specificity (*k*_cat_/*K*_M_) of *Mm*LipN against each of the 33 substrates with ester classes labeled. Catalytic efficiency values (*k*_cat_/*K*_m_) are given ± SD based on at least three independent kinetic replicates. Detailed kinetic results for each substrate are provided in Table S1. (C, D) Kinetic activity of *Mm*LipN against high-activity ester substrates **1** (black circles), **7** (blue squares), and **20** (red diamonds) and low-activity ester substrates **12** (green circles), **16** (red squares), **27** (purple diamonds), and **31** (orange squares). Low- and high-activity substrates display classic Michaelis–Menten kinetics with *Mm*LipN and activity above background hydrolysis rates. Traces colored based on ester classification from (A, B). All measurements were completed in at least triplicate and shown ± SD.

Building on previous fluorogenic ester substrate libraries, we synthesized new ester substrates to determine the ability of LipN to catalyze the hydrolysis of tertiary and constrained esters (Figure 1).^35,37−42^ In these fluorogenic ester substrates, protection of the phenolic oxygens locks fluorescein into a nonfluorescent lactone form pending hydrolysis of the protecting esters by LipN, which releases the highly fluorescent quinoid form of fluorescein.^43,44^ These fluorogenic substrates have low background hydrolysis, a large dynamic range, and are produced via a divergent synthetic pathway; successful synthesis was confirmed by NMR and high-resolution mass spectrometry (HRMS) (Supporting Information).^37,38^

The fluorogenic library is separated into structural groups based on common subclasses of serine hydrolase activity with expansion of the tertiary and bulky esters in the current substrate library (Figure 1).^37,40,45,46^ Within these subclasses, the alkyl ester substrates (**1**–**6**) allow general classification of serine hydrolases based on substrate length preferences and the introduction of polarity and unsaturation into these substrates (**7**–**11**; **12**–**15**) provide greater solubility, reactivity, and selectivity.^38^ As a proposed arylesterase with specificity for xenobiotics,^15^ LipN may selectively recognize cyclic, hydrophobic, or constrained esters (**16**–**21**, **32**). Pivalic tertiary esters like **22** are common ester prodrug-protecting groups that provide extended cellular stability and solubility.^33^ Tertiary esters including **23**–**30**, and **33** build on the known stability and orthogonal reactivity of cyclopropyl esters to hydrolysis by mammalian serine hydrolases but test the tertiary ester reactivity of bHSLs like LipN.^33,34^ The inherent stability of these tertiary esters to endogenous mammalian serine hydrolases has been applied to selectively remove cyclopropyl esters for imaging and protein engineering applications by the introduction of exogenous esterase activity.^33,34^ Stable tertiary esters could also have applications in designing mycobacterial-specific antibiotic prodrugs as a cyclopropyl ester-protected version of ethambutol, a front-line TB therapeutic, was stable in aqueous solutions.^36,47^

### Substrate Specificity of LipN

Detailed enzyme kinetics were measured for each of the 33 fluorogenic substrates against purified LipN (Figure 1). For our kinetic analysis, we used a purified LipN homologue from *Mycobacterium marinum*, which has 73% sequence identity to LipN from *M. tuberculosis* (Figure S1 and Table S4). Matching with previous reports, our attempts at the isolation of *M. tuberculosis* LipN failed to produce fully folded and active protein upon heterologous expression in *Escherichia coli*.^13,15^ Since one of our experimental goals was to characterize a significant number of LipN variants, we searched for a closely related LipN homologue that could be produced in a fully folded and active state directly from *E. coli*. Testing a limited number of homologues, LipN from *M. marinum* was found to be the most closely related homologue (Figure S1) that produced a significant quantity of protein after IPTG-based expression at 30 °C for 3 h and a single nickel affinity chromatography purification step.

For each fluorogenic ester substrate, plots of initial rates versus substrate concentration were fitted to the Michaelis–Menten equation to determine values for the kinetic constants (*k*_cat_, *K*_M_, *k*_cat_/*K*_M_) (Supporting Material). Both high-activity (Figure 1C) and low-activity (Figure 1D) substrates showed classic saturation enzyme kinetics with similar *K*_M_ values but significant variations in the *k*_cat_ values. Although reduced from high-activity substrates, LipN activity against low-activity substrates is still above background hydrolysis rates for the fluorogenic substrates (Figure 1D). Main substrate specificity comparisons were performed using the *k*_cat_/*K*_M_ ratio or catalytic efficiency (Figure 1B), which provides a weighted comparison of the two catalytic constants and measures the substrate preference of a single enzyme for variable substrates.^38^

Matching with previous activity measurements and reinforcing its assignment as a carboxylesterase,^13,15^ LipN strongly preferred shorter ester substrates with highest activity against the acetyl ester substrate (**1**). This strong selectivity based on alkyl chain length also parallels the strict selectivity measured for LipW, another mycobacterial Lip family member, and the closest mycobacterial serine hydrolase homologue to LipN (37% sequence identity; Figure S1).^40^ For LipW, this selectivity for acetyl esters was controlled by a shallow, hydrophobic binding site for the carboxylic acid portion of the ester substrate and this may be a structural feature that is conserved in LipN.^40^ LipN maintained substantial activity against straight-chain propionyl (**2**) and butyl (**3**) esters, but strongly selected against branching close to the carbonyl (**6**, **12**).

Within other substrate classes, a similar pattern emerged where LipN showed the highest activity against the shortest or least bulky substrates within each class (Figure 1B). The only notable exception to this pattern was the oxazole ester (**20**) within the cycloalkyl esters, where a nonaromatic cyclopentyl ester (**17**) and even a furan ester (**19**) were significantly less active than the oxazole. The oxazole substrate was the second most activated substrate (*k*_cat_/*K*_M_ = 91,000 ± 22,000 M^–1^ s^–1^; Table S1) across the entire fluorogenic substrate library (Figure 1B). This oxazole ester contains an aromatic and hydrogen bond donor/acceptor structure present in xenobiotics. Oxazoles are highly represented in medicinal chemistry libraries, including antimicrobially active compounds, and are present in natural antibacterial compounds constructed through the cyclization and oxidation of serine and threonine in nonribosomal peptides.^48^ To the best of our knowledge, oxazole esters have not been previously tested as selective substrates for esterases, so future investigation will be required to determine if this oxazole ester reactivity is unique to LipN.

For tertiary esters, LipN showed only minimal activity across the full range of ester substrates with highest activity against substrates **27** (*k*_cat_/*K*_M_ = 7400 ± 2200 M^–1^ s^–1^) and **29** (*k*_cat_/*K*_M_ = 2600 ± 1700 M^–1^ s^–1^) and *k*_cat_/*K*_M_ values >500 M^–1^ s^–1^ for substrates **23**, **25**, and **30**. Tertiary ester substrate **29** had similar activity to other closely branched substrates (**6**, **12**, **16**), indicating that the steric branching in the tertiary esters was restraining LipN’s activity. LipN’s highest activity for substrate **27** among tertiary ester substrate also reinforced its preference for substrates with hydrogen bonding and/or polar substitutions within the ester substrate.

**Figure 2:**
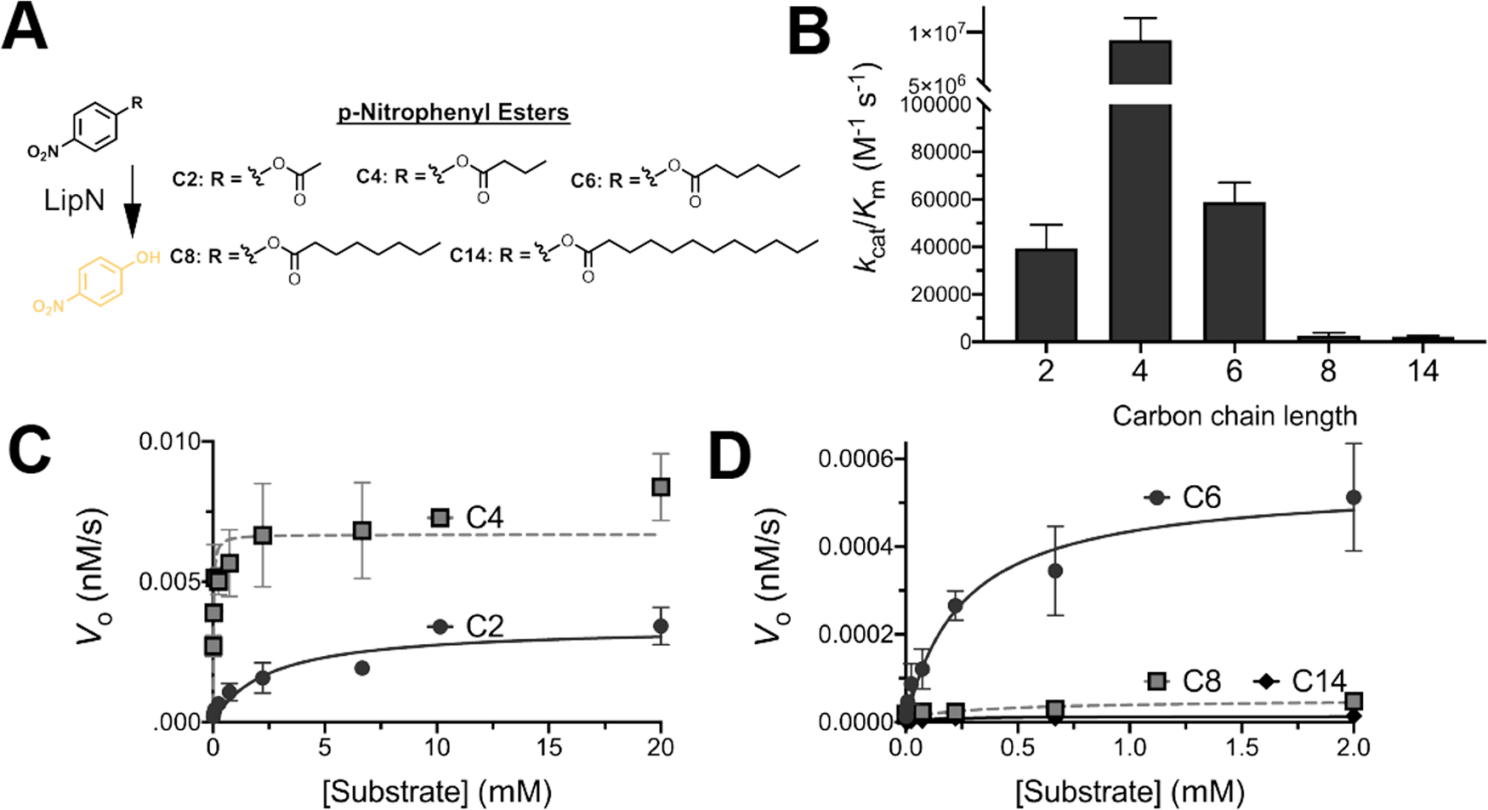
Alkyl ester substrate specificity of LipN. (A) Classic *p-*nitrophenyl ester substrates were used as conformation for fluorogenic substrate measurements (Figure 1). (B) Catalytic efficiency comparison of *Mm*LipN against *p*-nitrophenyl substrates. Catalytic efficiency values (*k*_cat_/*K*_m_) are given ± SD. Detailed kinetic data are provided in Table S2. (C, D) Kinetic activity of *Mm*LipN against (C) short alkyl ester substrates: *p-*nitrophenyl acetate (C2) and *p-*nitrophenyl butyrate (C4) and (D) longer alkyl ester substrates: *p-*nitrophenyl valerate (C6), *p-*nitrophenyl octonoate (C8), and *p-*nitrophenyl myristate (C14). Data points were fitted to the Michaelis–Menten equation and are shown ± SD.

To confirm that our LipN homologue from *M. marinum* expressed heterologously in *E. coli* showed comparable properties to the previously characterized LipN from *M. tuberculosis*,^13,15^ we measured the kinetic activity of LipN from *M. marinum* against classic *p*-nitrophenyl ester substrates (Figure 2). Matching with previous measurements,^15^ LipN showed highest activity against *p*-nitrophenyl butyrate and measurable activity with ester substrates up to six carbons (Figure 2B). LipN’s higher activity for the four-carbon *p*-nitrophenyl substrate contrasts with the fluorogenic ester substrates where the two carbon was strongly preferred (Figure 1B). This substrate specificity shift may reflect the increased size of the fluorescein scaffold vs the *p-*nitrophenyl scaffold or the inherent preference of LipN for the *p*-nitrophenyl alkyl, which mimics the hydroquinine alcohol from its proposed xenobiotic substrate, 4-hydroxyphenyl acetate.^15^

### Substrate Specificity Determinants in LipN

**Figure 3:**
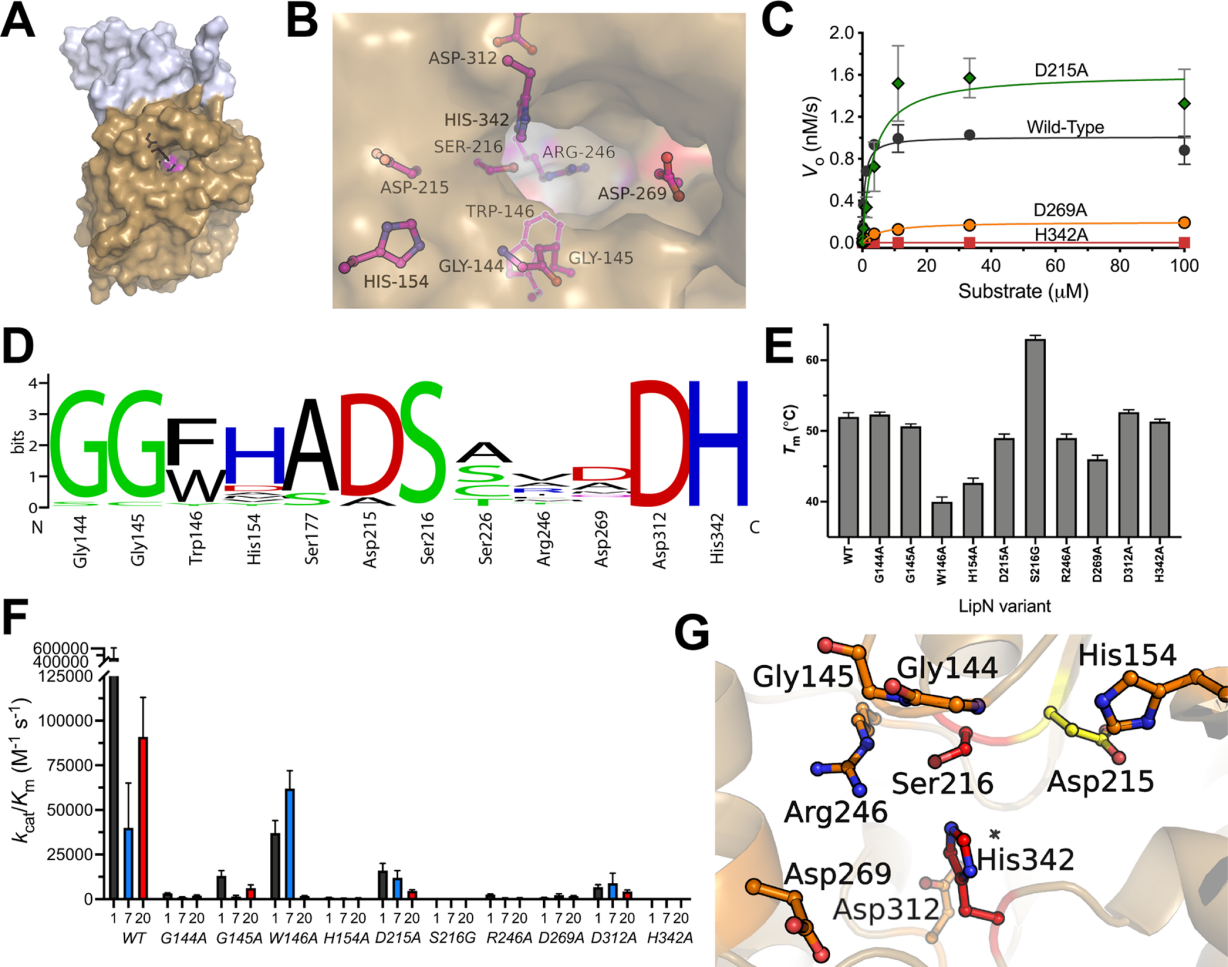
Binding pocket architecture of LipN. (A) Modeled structure of *Mm*LipN. The α/β Hydrolase domain is shown in tan with the cap domain in gray. The catalytic triad residues are colored magenta and shown in sticks (Ser216, His342, and Asp312). The structural homology model was built using the Robetta server based on rPPE (PDB ID: 4OU5) with a confidence interval of 0.8 and overlap between rPPE and *Mm*LipN at 320 of 370 residues in *Mm*LipN.^49^ (B) Binding pocket and catalytic residues substituted with alanine in *Mm*LipN. Each of the residues shown in ball and stick was individually substituted with alanine, and the relative contribution of each side chain to the catalytic activity and thermal stability was determined. (C) Representative kinetic activity of *Mm*LipN binding pocket and active site variants against the highest-activity substrate **1**. Complete kinetic analysis of *Mm*LipN variants is provided in Table S3. All measurements were completed in at least triplicate and shown ± SD. (D) Conservation of binding pocket residues across bacterial homologues of *Mm*LipN. Sequences aligned using Clustal Omega and relative weightings were performed using Weblogo.^51,52^ Detailed sequence analysis is given in Table S4 with phylogenetic analysis in Figure S1. (E) Thermal stability of *Mm*LipN variants. The thermal stability of each *Mm*LipN variant was determined by measuring the increase in Sypro Orange fluorescence in response to increasing temperature.^53^ Measurements were completed in at least triplicate and are reported ± SD. (F) Catalytic activity of *Mm*LipN binding pocket variants. The catalytic activity of each of the *Mm*LipN variants was determined against substrates **1**, **7**, and **20** (Figure 1A). Data points were fitted to the Michaelis–Menten equation and are shown ± SD. Detailed kinetic and thermal stability analyses for binding pocket variants are given in Table S3. (G) Relative contributions of binding pocket and active site residues to the catalytic activity of *Mm*LipN. Exterior surface and stick representation of the binding pocket of *Mm*LipN color-coded for their relative contributions to the catalytic activity of *Mm*LipN against substrate **1**. Colors represent relative decreases to its catalytic activity (*k*_cat_/*K*_M_) upon substitution to alanine: red (>99.9% reduction), orange (99.9–95% reduction), and yellow (95–90% reduction).

Focusing on conserved bHSL motifs,^23^ we analyzed the substrate binding and catalytic residues predicted in a structural model to surround the binding pocket of LipN (Figure 3). The structural homology model was built using the Robetta server based on rPPE (PDB ID: 4OU5),^49^ a bHSL homologue from *Pseudomonas putida*. Like LipN, rPPE has broad substrate specificity and a preference for short ester substrates (≤4 carbons).^50^ The substrate preference of rPPE was also evolved to introduce chiral substrate recognition, further reinforcing the bioengineering applications of bHSLs.^49^ The LipN model had a confidence interval of 0.8 and had overlap between rPPE and LipN at 320 of 370 residues in LipN with the variable N-terminal domain having the greatest uncertainty. The final model of LipN reflects the higher certainty in the core α/β hydrolase domain (tan; Figure 3A) and the lower certainty in the modeled N-terminal domain, which had higher flexibility and uncertainty in the top scoring Robetta models.

Using the structural model (Figure 3A,B), the binding pocket of LipN was mapped with the catalytic serine (Ser216) at the bottom of a fairly shallow binding pocket. The bottom of the binding pocket is defined by a highly variable arginine residue (Arg246; Figure 3D and Table S6) and a conserved aromatic tryptophan (Trp146). To assign the roles of binding pocket residues in the folded stability and catalytic activity of LipN, amino acids surrounding the binding pocket were individually substituted with alanine (glycine for Ser216) and the variants were purified to homogeneity. Thermal stability was measured to confirm proper folding of each variant and to determine the importance of each binding pocket residue to the folded stability of LipN (Figure 3E and Table S4).^53^ Kinetic constants of each individual variant were then remeasured with the three highest-activity fluorogenic substrates **1**, **7**, and **20** from the initial screen. These three substrates were chosen to observe absolute changes in the catalytic activity of LipN and to look for potential shifts in the substrate preference upon substitution (Figure 3C,F and Table S3).

**Figure 4:**
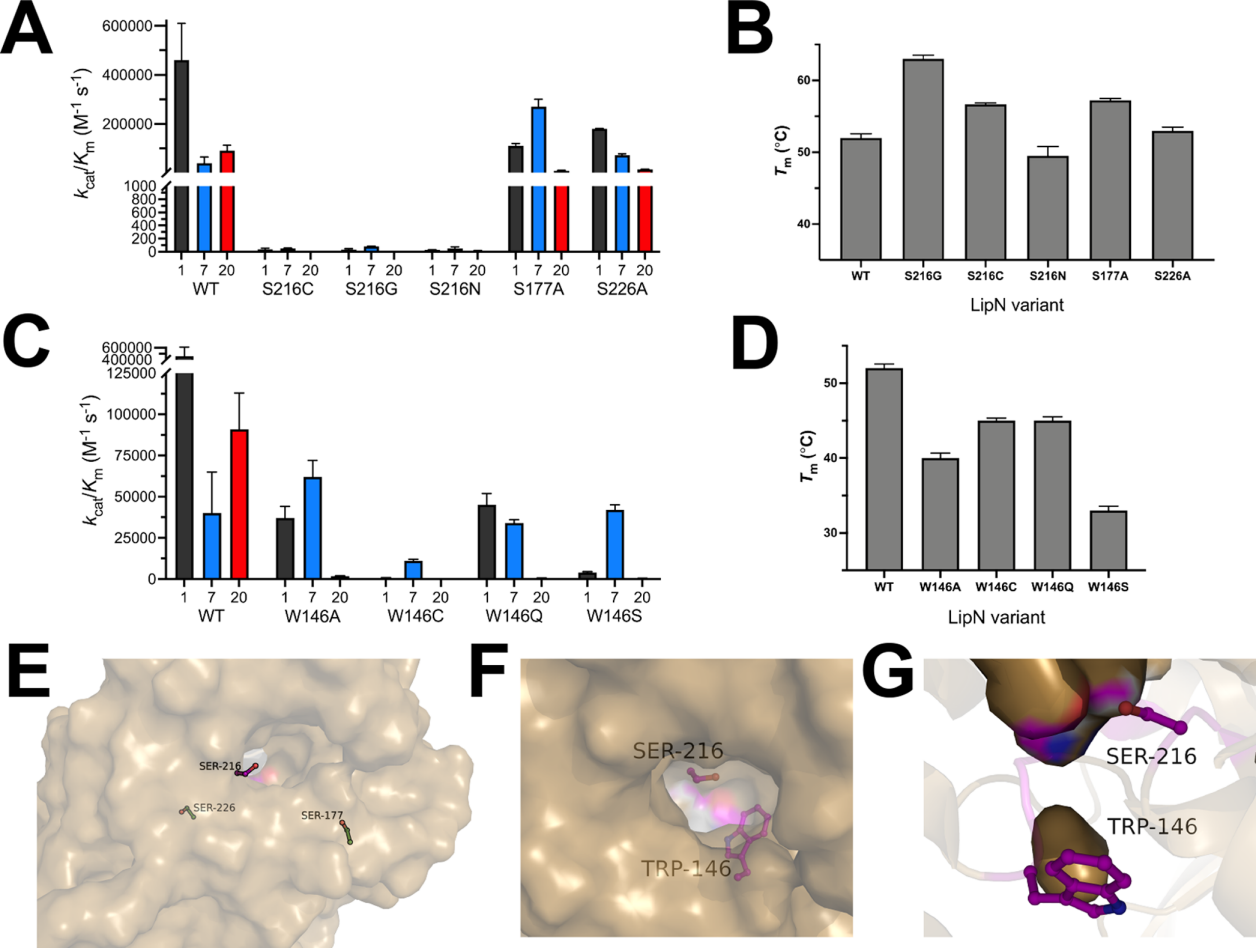
Detailed analysis of two key binding pocket residues. Kinetic analysis of *Mm*LipN variants at Ser216 (A) and Trp146 (C) against substrates **1**, **7**, and **20** (Figure 1A). Thermal stability of *Mm*LipN variants at Ser216 (B) and Trp146 (D). All measurements were completed as outlined in Figure 3 and are shown ± SD. Detailed biochemical analysis of Ser216 and Trp146 is provided in Tables S4 and S5, respectively. (E) Surface structure of *Mm*LipN illustrating the distant location of control serine substitutions (Ser177 and Ser226; green) from the nucleophilic serine (Ser216, magenta). (F, G) Location of Trp146 in relation to the nucleophilic serine (Ser216) from the exterior surface (F) and interior surface (G) of the binding pocket.

Thermal stability measurements showed that all LipN variants were stable above 37 °C with a *T*_M_ value of 52 ± 0.6 °C for wild-type LipN. Substitution of the catalytic serine by glycine increased the thermal stability by 11 °C (*T*_M_ = 63 ± 0.5 °C), which likely reflects the removal of the distorted torsion angle required for proper catalytic orientation of the nucleophilic serine and the increased flexibility imparted by the glycine substitution.^40^ The majority of other alanine variants (G144A, G145A, R246A, D312A, and H342A) maintained *T*_M_ values similar to wild-type LipN (±3 °C). The three positions with the largest shifts in *T*_M_ values are buried in the predicted model (Figure 3B), as D269A had a decreased *T*_M_ of 46 ± 0.6 °C and H154A of 42.7 ± 0.7 °C. The largest decrease in thermal stability was observed upon substitution of Trp146 to alanine (*T*_M_ = 40.0 ± 0.7 °C; Table S3). Trp146 is connected with the oxyanion hole in bHSLs and is hypothesized to be critical to the catalytic activity of LipN.^15^ In the model of LipN, Trp146 packs against the catalytic serine and the majority of the indole ring is buried within the predicted binding pocket (Figure 3B). Trp146 occupies the X position in the conserved HGGGX oxyanion hole motif in bHSLs. This motif is a differentiation point within bHSLs with either an aromatic Trp or Phe present within all closely related LipN homologues (Figure 3D). This large decrease in the thermal stability of LipN with alanine substitution of Trp146 complicates the analysis of the W146A variant’s kinetic results. Further substitutional analysis of Trp146 was performed to differentiate the role of this residue in the folding and catalytic activity of LipN (Figure 4).

Matching with previous analysis, substitution of the nucleophilic serine (Ser216) and general base histidine (His342) in the catalytic triad residues completely abolished LipN activity.^15^ Substitution of the acidic residue (Asp269) of the catalytic triad with alanine led to 4-fold to 70-fold decreases in catalytic activity, depending on the fluorogenic substrate (Figure 3C,F and Table S3). This pattern of essentiality for the nucleophilic serine and catalytic base histidine and lesser role for the acidic asparate/glutamate residue has been observed in a variety of serine hydrolases and other bHSLs.^40,45,46^ Within other conserved bHSL motifs, oxyanion hole residues—Gly 144 and Gly145—were found to be required for complete catalysis, as conservative substitutions with alanine decreased the *k*_cat_/*K*_M_ values by 15- to 140-fold against all three fluorogenic substrates. The only substitution without a near complete loss of catalytic activity was D215A, which had *k*_cat_/*K*_M_ values within 3-fold of wild-type LipN for substrates **1** and **7**. In the modeled structure of LipN, the carboxylic acid side chain of Asp215 is pointed away from the binding pocket of LipN and likely only affects the catalytic activity indirectly through interactions with the nucleophilic serine.

The drastic shifts in catalytic activity with every other substitution across the binding pocket of LipN are unusual in comparison to other bHSLs, including the closest mycobacterial homologue LipW (Figure S1). For LipW, substitution of the catalytic triad completely inhibited all catalytic activity but every other alanine substitution, including an oxyanion hole glycine (Gly91) decreased the *k*_cat_/*K*_M_ by less than 4-fold.^40^ The large decreases in catalytic activity upon all binding pocket substitutions for LipN are especially stark as wild-type LipN has higher baseline catalytic activity (*k*_cat_/*K*_M_ = 4.6 × 10^5^ M^–1^ s^–1^ for substrate **1**) than LipW (*k*_cat_/*K*_M_ = 1.4 × 10^4^ M^–1^ s^–1^ for substrate **2**) and multiple residues (His154, Asp269) for LipN are relatively distant from the catalytic serine (Figure 3B). This essentiality of binding pocket residues in LipN across amino acid properties (hydrophobicity, aromaticity, polarity, charge, size) is reflected in Figure 3G with shifts in catalytic activity upon substitution color-coded by essentiality. Together the substitutional analysis of the LipN binding pocket suggests a fairly rigid binding pocket preorganized for catalysis of short ester substrates where substitution of diverse amino acids across the binding pocket significantly impacted the folded stability and catalytic activity of LipN.

### Detailed Analysis of the Rigidity of the LipN Binding Pocket

To confirm the rigidity and lack of plasticity in the LipN binding pocket, we performed expanded substitutional analysis and focused on two residues (Ser216 and Trp154) with divergent roles in catalysis and folding of LipN (Figure 4). The nucleophilic serine presents a location where substitution increased the thermal stability of LipN and led to the expected loss of catalytic activity. The catalytic serine is also a site of regulation through direct phosphorylation under varying growth conditions.^20^ In comparison, substitution of Trp146 led to a substantial decrease in the thermal stability and catalytic activity of LipN that may be connected or independent roles for this residue. For both Ser216 and Trp146, we constructed additional substitutional variants and remeasured their thermal stability and catalytic activity.

For Ser216, further substitutions with cysteine or asparagine led to a complete loss of catalytic activity similar to the glycine variant (Figure 4A). More interestingly, the 11 °C increase in thermal stability observed for the S216G variant was not observed with other Ser216 variants (Figure 4B). Substitution with cysteine (S216C) led to a smaller ∼5 °C increase in thermal stability, whereas a slightly larger asparagine (S216N) decreased the thermal stability of LipN from wild-type (S216N *T*_M_ = 49.5 ± 1.3 °C). As a control for this thermal stability effect, two serines (Ser177 and Ser226) distantly located from the catalytic site (Figure 4E) were substituted with alanine and the effect on the thermal stability was measured (Figure 4B). Conservative substitution of Ser226 maintained thermal stability in line with wild-type LipN (S226A *T*_M_ = 53.0 ± 0.5 °C) while S177A had increased thermal stability (*T*_M_ = 57.2 ± 0.3 °C) matching with the S216C variant. The increased *T*_M_ value of S177A likely reflects a natural preference for alanine at this position as alanine is present in 20 out of 22 homologues, including all of the closest mycobacterial homologues (Table S6).

In contrast to variable thermal stabilities, every substitution of Ser216 led to complete loss of LipN catalytic activity (Figure 4A). Complete loss of activity with each substitution also suggests that phosphorylation of this serine is likely to completely inhibit LipN activity.^20^ Given that LipN was phosphorylated by 6 out of 9 mycobacterial kinases tested and was phosphorylated under logarithmic and hypoxic growth conditions, phosphorylation is likely a major regulator of LipN activity.^20^ Even with this phosphorylation regulation, LipN is robustly active as it was labeled in every current ABPP study under five different growth conditions using a variety of covalent ligands.^9−11,17,18^ Identifying the growth conditions for LipN phosphorylation will help clarify its biological substrates and roles.

To refine the role of Trp146 in the folding and activity of LipN, four additional Trp146 variants were constructed with variable amino acid substitutions (Figure 4C,D). Matching with the alanine variant (W146A), substitution of Trp146 with the four varying amino acids decreased the thermal stability by >7 °C with serine substitution (W146S) causing the largest loss in thermal stability (Δ*T*_M_ = 19.0 °C; Figure 4D). Shifts in Trp146 thermal stability track with a combination of the amino acids polarity and accessible surface area with larger amino acids (Gln, Cys), showing smaller decreases in thermal stability and more polar amino acids of similar sizes (Ser versus Ala) showing larger decreases in thermal stability. These thermal stability measurements reinforce the key role of Trp146 in controlling the folding of LipN with the size and aromaticity of Trp146 key to LipN’s stable folding.

Trp146 also bookends a key substrate selectivity motif in bHSLs (HGGGW) (Figure 4F,G), which encompasses the oxyanion hole.^23^ Substrate variables including length, branching, and tertiary ester reactivity have all been correlated to differences in the sequence of this motif.^26−28^ The presence of a tryptophan at the end of this motif endowed the RmEstB esterase from *Rhizomucor miehei* with tertiary alcohol ester activity.^28^ Comparison of RmEstB to the homologous RmEstA esterase from *R. miehei* showed that substitution of the tryptophan in RmEstB with phenylalanine in the RmEstA motif (HGGGF) shifted RmEstA’s substrate preference to longer ester substrate (C_6_–C_16_) vs the shorter substrate preference (C_2_–C_6_) for RmEstB.^26^ This strong preference for shorter ester substrates in RmEstB based on the HGGGW motif matches with LipN, which strongly preferred short and minimally branched substrates (Figure 1).

Our combined kinetic, substrate, and substitutional analysis of the Trp146 variants provided a complex picture of Trp146’s role in controlling the substrate specificity of LipN across three ester substrates (**1**, **7**, and **20**). With the acetyl ester (**1**) substrate, Trp variants showed a >10-fold decrease in catalytic efficiency (*k*_cat_/*K*_M_) due to elevated *K*_M_ values (Table S6) with the W146C and W146S variants having *k*_cat_/*K*_M_ shifts of 100- to 1000-fold (Figure 4C). Contrasting substrate **1**, wild-type LipN and all of the Trp146 variants showed minimal variation in their *k*_cat_/*K*_M_ values (<4-fold) for the four-atom chain polar ester substrate (**7**). Values for *k*_cat_ and *K*_M_ for every Trp146 variant were largely constant across these tryptophan substitutions for substrate **7**. Substitution of Trp146 did not however increase the catalytic activity of LipN as seen for RmEstB versus RmEstA,^26^ as none of the Trp146 variants showed a substantial increase (>2-fold) in their *k*_cat_/*K*_M_ values against this longest substrate (Figure 4C). The most drastic shifts in the catalytic activity were observed with the oxazole substrate (**20**) where all Trp146 substitutions decreased the *k*_cat_/*K*_M_ values between 50- and 100,000-fold, which were correlated with large increases in *K*_M_ values (Table S5). The selectivity of LipN for oxazole esters is unlikely to be due to favorable π-stacking of the aromatic surfaces of Trp146 and the oxazole ring, as the modeled structure of LipN places Trp146’s indole ring in a side orientation toward the binding pocket where its π-cloud would not stack with the oxazole ring (Figure 4F,G). Other aromatic substrates had substantially lower activity with LipN, suggesting that the increased activity of LipN with **20** is specific to the properties of the oxazole ring.^48^ Further dissecting the complex role of Trp146 in controlling the substrate specificity of LipN will require detailed structural analysis, as substitution of this large, aromatic tryptophan residue with the smaller and mostly polar substituents may lead to larger shifts in the binding pocket structure of LipN. Our current attempts to crystallize LipN and its variants have not been fruitful, but efforts are ongoing.

## Discussion

Serine hydrolases like esterases and lipases are components of many industrial solvents from detergents and cleaners to green synthesis reagents.^54,55^ To expand their industrial applications, bioengineering efforts have rationally and combinatorially expanded their substrate specificity and reactivity.^55−57^ Among serine hydrolases, bHSLs are a promising subfamily for biocatalyst development as subfamily members show significant stability and activity in organic and aqueous solutions across a range of pHs and ionic strengths with diverse substrates.^23,28^ These desirable properties of bHSLs are related to sequence motifs surrounding the binding pocket, to the organization of the lid/cap domain, and to the quaternary structure of bHSLs.^23^

The bHSL family is well represented in *Mycobacteria* with twelve members encoded in *M. tuberculosis*.^13^ Among mycobacterial bHSLs, the most well-studied member is LipY, which catalyzes the cleavage of triacylglycerols and breaks down intracellular lipid stores during low nutrient growth conditions.^58,59^ The biological substrates and functions of the majority of mycobacterial bHSLs are however unknown, and identifying these substrates is complicated by the overlapping substrate specificity of most mycobacterial bHSLs against short (∼4 carbon) substrates.^13^*In vivo* ABPP characterization against varying covalent substrates however shows that mycobacterial bHSLs are active under diverse growth conditions and that general inhibition of these mycobacterial bHSLs is a viable strategy for inhibiting mycobacterial growth.^9,11,60^

Matching with other mycobacterial bHSLs, previous substrate specificity measurements with LipN showed it had highest activity toward three- and four-carbon ester substrates with emergent activity toward the xenobiotic substrate, 4-hydroxyphenyl acetate.^13,15^ LipN was also covalently labeled with diverse ABPP ligands including tetrahydrolipstatin, a PEG-based fluorophosphonate, a desthiobiotin fluorophosphonate, the oxadioazolone inhibitor M*m*PPOX, and a bulky cyclopostin inhibitor with a 17 carbon alkyl chain, indicating a diverse substrate reactivity profile.^9−11,17,18^ We have now expanded the substrate specificity profile of LipN using a library of 33 fluorogenic ester substrates. The overall LipN substrate profile indicates a strong substrate preference for shorter (<4 atom) ester substrates across all substrate classes, but with significant activity against at least one ester substrate across all six substrate classes.

Given the ability of other bHSLs to catalyze reactions with tertiary esters and the ability of LipN to catalyze a reaction with the tertiary ester substrate, 4-hydroxyphenyl acetate,^15^ we included 9 new tertiary ester substrates in our comprehensive LipN substrate screen. LipN however had only minimal activity against the majority of these tertiary esters with highest catalytic activity against two of the smallest tertiary esters. (**27**; **29**)

LipN did however have unexpectedly high activity against an oxazole ester substrate (**20**). Oxazoles are common moieties in antibiotics and other pharmaceuticals with their heterocyclic system imparting preferential specificities.^48^ Closely related oxazoline rings are found in the natural *M. tuberculosis* iron chelator, mycobactin, and potent mycobacterial inhibitors were developed around the mycobactin scaffold with oxazoles in place of oxazolines.^61,62^ LipN selects specifically for the oxazole ester, as other five-membered ring substrates (**17**; **19**) were over 25-fold less active. A furan ester (**19**) which only differs from the oxazole ester (**20**) by a single nitrogen in the ring has the same *k*_cat_ value but has a 25-fold higher *K*_M_ value (Table S1), indicating that the difference is mainly related to shifts in substrate binding affinity. Although oxazole esters are not common ester substrates, the M*m*PPOX inhibitor for mycobacterial bHSLs contains a five-membered heterocyclic core with oxygen and nitrogen substituents similar to the oxazole ester.^13,18^ Among mycobacterial bHSLs, LipN was also most strongly inhibited by M*m*PPOX at stoichiometric concentrations of inhibitor, suggesting that the selectivity of LipN for oxazole esters might have therapeutic relevance.^13^

LipN has this fairly narrow substrate specificity due to a predicted shallow binding pocket with a mixture of polar and nonpolar residues encircling the nucleophilic serine. The majority of binding pocket residues are well conserved with the exception of three polar residues (His154, Arg246, and Asp269). Given the importance of each of these three polar residues to the catalytic activity of LipN, these polar residues may represent substrate differentiation points between LipN and other similar mycobacterial bHSLs.^40^ The overall picture of the substitutional analysis is a LipN binding pocket intransigent to substitution at least with alanine or other small amino acids. This decrease in catalytic activity upon substitution was not a universal feature to LipN as control alanine substitutions (S177A; S226A) far from the binding pocket retained near wild-type catalytic activity (Table S4). This immutability of LipN to substitution is surprising, as bHSLs are common bioengineering targets,^55^ and LipW, the closest mycobacterial bHSL homologue to LipN, retained high catalytic activity even with alanine substitutions to predicted oxyanion hole residues.^40^ Disentangling this intractability to mutation in LipN is another goal of our ongoing attempts at structural analysis for LipN.

The one outlier to this pattern of universal loss in catalytic activity upon substitution was Trp146, as the W146A variant showed differential shifts in catalytic activity based on fluorogenic ester substrate with wild-type level activity against a four-atom chain alkyl ether ester (**7**) but with >10-fold decreases in activity for an acetyl ester (**1**) and oxazole ester (**20**) substrate. More complex patterns in the catalytic activity were also observed with variable amino acid substitutions to Trp146, pinpointing Trp146 as an interesting point for substrate differentiation in LipN. Previously Trp146 had been identified as a key stabilization point in the folded stability of LipN and we further confirm this essential role for Trp146 but also add an essential role as a substrate selectivity residue.^15^ This substrate selectivity role matches with other bHSLs where this residue flanking the oxyanion hole motif (HGGGX) imparts substrate selectivity based on substrate size and length and the ability to recognize tertiary esters.^23^ The most studied example is *RmEstB* from the thermophilic fungus *R. miehei* where its HGGGW motif imparted selectivity for short ester substrates, preferring acetyl and butyl esters like LipN, but facilitated tertiary ester hydrolysis.^26,28^ Conversion of the terminal tryptophan to phenylalanine in *RmEstB* or its homologue *RmEstA* lengthened their substrate preferences and reduced tertiary ester hydrolysis.^26^ A similar relationship was seen in the carboxylesterase EstA from *Streptomyces coelicolor* where substitution of this tryptophan in the HGGGW motif increased its relative activity toward longer ester substrates while maintaining its absolute preference for acetyl esters.^27,63^ Trp146 in LipN displays similar complex effects on its substrate specificity and future studies will focus on examining a more complete list of amino acid substitutions at position 146.

## Conclusions

Controlling *M. tuberculosis* is an ongoing public health issue with new approaches for treatment and diagnosis needed.^64,65^ Mycobacterial bHSLs are an expanded enzyme family in *M. tuberculosis* with confirmed expression and catalytic activity across a range of therapeutically relevant growth conditions.^13^ Accumulating evidence indicates that the mycobacterial bHSL LipN is a key metabolic enzyme in *M. tuberculosis* with post-translationally regulated catalytic activity during hypoxic, recovery, and high nutrient growth conditions.^10,11,20^ Although the biological substrate of LipN still remains to be discovered, LipN strongly prefers small, unbranched esters across multiple substrate libraries. Previous analysis showed that LipN is active against the xenobiotic tertiary ester of 4-hydroxyphenyl acetate,^15^ but our expanded tertiary esters showed that the tertiary ester activity of LipN is fairly limited even though LipN contains a conserved HGGGW motif, correlated with tertiary ester activity in other bHSLs.^23^ The terminal tryptophan in this motif (Trp146) is however central to the folded stability of LipN, providing multifaceted control over the substrate preference of LipN and representing a site for future bioengineering efforts. Besides Trp146, LipN showed limited plasticity toward substitution, indicating a pre-arranged binding pocket and catalytic arrangement. Unique among the LipN substrate specificity map was a strong selectivity for an oxazole ester substrate even from other polar substituted five-membered aromatic substrates. Intriguingly, this oxazole ester is present in previous general mycobacterial HSL inhibitors^13,18,61,62^ and is a promising scaffold for designing new mycobacterial HSL inhibitors and diagnostic substrates.

## Materials and Methods

### Overexpression and Purification of LipN

A bacterial expression plasmid (AVA0421; a derivative of pET14b) containing the *LipN* gene from *M. marinum* strain ATCC BAA-535/M (Genbank: WP_036455371.1; UniProt: B2HIK1; protein name *Mm*LipN) was obtained from BEI Resources (NR-27748). The LipN gene was subcloned into pET-28a using *Xba*I and *Xho*I restriction enzymes to yield an inframe N-terminal 6X his tag. This bacterial plasmid (pET-28a-*Mm*LipN) was transformed into *E. coli* LOBSTR (DE3) cells (Kerafast, Boston, MA). Cells were grown in LB media (1 L), containing 40 mg/ml kanamycin and 25 mg/mL chloramphenicol, while shaking at 225 rpm, 37 °C. Upon reaching an optical density, OD_600_, of about 0.8, temperature was decreased to 30 °C and cells were induced by adding isopropyl β-d-1-thiogalactopyranoside (IPTG) to a final concentration of 1.0 mM, followed by continued shaking 3 h. Cells were centrifuged at 6000*g* (10 min, 4 °C); the pellet was resuspended in PBS pH 7.4. BugBuster detergent and lysozyme (500 mg) were added and the mixture rotated for 2–3 h (4 °C). The lysate was centrifuged (16,000*g*, 4 °C, 10 min), and the pellet was discarded. Nickel-NTA agarose resin (GoldBio; St. Louis, MO) was added (0.6 mL) to the supernatant, and the mixture was gently rotated (1 h, 4 °C), followed by centrifugation (3000*g*, 2 min). The resin was washed in five iterations (30 mL each) with PBS +10 mM imidazole. Purified LipN protein was eluted with PBS + 250 mM imidazole (1.0 mL). The eluted protein was dialyzed exhaustively at 4 °C against PBS containing 2 mM DTT. LipN purity was confirmed by sodium dodecyl sulfate–polyacrylamide gel electrophoresis (SDS–PAGE) on 4–20% acrylamide gels (Invitrogen) stained with colloidal Coomassie brilliant blue; theoretical *M*_W_ = 46.4 kDa. Protein concentration was determined by measuring absorbance at 280 nm, using the theoretical molar absorptivity 47,440 M^–1^ cm^–1^, assuming all cysteines are reduced. With the loss of a chromophore, the extinction coefficient was adjusted to 41,940 M^–1^ cm^–1^ for all Trp146 variants.

### Site-Directed Mutagenesis of LipN

Mutagenesis of LipN was performed using the standard procedure for the Quikchange site-directed mutagenesis kit (Agilent; La Jolla, CA). Proper mutations in the *Mm*LipN DNA sequence were confirmed by DNA sequencing (Genewiz, South Plainfield, NJ) using T7 and/or T7-terminal sequencing primers. Plasmids coding for *Mm*LipN variants were transformed into *E. coli* LOBSTR (DE3) cells and variants of *Mm*LipN were overexpressed and purified using the same procedure as for wild-type *Mm*LipN.

### Kinetic Analysis of Enzymatic Activity

The enzyme kinetic activity of LipN was assayed via the hydrolysis of the fluorogenic substrates (Figure 1) in accordance with past kinetic analysis of mycobacterial serine hydrolases.^37,38,40,66,67^ Kinetic assays were run in triplicate on black 96-well plates in a Synergy H1 Hybrid Reader (BioTek). Fluorogenic ester substrates (10 mM in DMSO) were diluted to a starting concentration of 100 μM in PBS containing acetylated BSA (Sigma; 0.1 mg/mL). Eight serial dilutions (1:1; 120 μL into 240 μL total volume) of each substrate were made using PBS–BSA. Fluorogenic substrate dilutions (95 μL) were then transferred to opaque 96-well microplate (Corning, Lowell, MA). Reactions were initiated by quickly adding enzyme (5 μL, 3.0 nM final concentration) to each assay well, mixed, and the fluorescence counts measured every 26–30 s for 4.5 min with a BioTek H1 Synergy multimode plate reader (Biotek Instruments; Winooski, VT) at ambient temperature. The instrument measured relative fluorescence through excitation at λ = 485 nm and emission at λ = 520 nm. The fluorescence change was converted to molar concentrations using a fluorescein standard curve (300–2.3 nM), whose fluorescence was measured simultaneously. The initial rates of the enzyme-catalyzed reactions were measured in triplicate and plotted versus fluorogenic substrate concentration. The initial rate of change in fluorescence in each reaction was plotted against substrate concentration in GraphPad Prism 9.0 (GraphPad Software, La Jolla, CA), fitted to a hyperbolic curve for standard Michaelis–Menten kinetics, and the *k*_cat_, *K*_M_, and *k*_cat_/*K*_M_ values were calculated.

### Kinetic Measurements with *p*-Nitrophenyl Substrate

The enzymatic activity of *Mm*LipN was measured against *p*-nitrophenyl acetate, *p*-nitrophenyl butyrate, *p*-nitrophenyl valerate, *p*-nitrophenyl octanoate, and *p*-nitrophenyl myristate (Sigma-Aldrich) using a 96-well microplate assay (Figure 2).^43,45,46^ Substrates (*p*-nitrophenyl acetate (2 M), *p*-nitrophenyl butyrate (2 M), *p*-nitrophenyl valerate (2 M), *p*-nitrophenyl octanoate (200 mM), and *p*-nitrophenyl myristate (200 mM)) were prepared as stock solutions in acetonitrile and diluted into PBS containing acetylated BSA (PBS–BSA; 0.1 mg/mL). The starting concentration for *p*-nitrophenyl acetate and *p*-nitrophenyl butyrate was 20 mM and for *p*-nitrophenyl valerate, *p*-nitrophenyl octanoate, and *p*-nitrophenyl myristate was 2 mM. Eight serial dilutions (1:2; 60 μL into 180 μL total volume; 20 mM to 9.1 μM final concentrations for acetate and butyrate and 2 mM to 0.91 μM for valerate, octanoate, and laurate) were made using PBS–BSA containing 1% acetonitrile. Substrate dilutions (95 μL) were transferred to a clear 96-well microplate and *Mm*LipN (5 μL; final concentration = 36 nM) was added to start the reaction. The absorbance change at 412 nm was measured on a Biotek Synergy H1 multimode plate reader (Biotek Instruments; Winooski, VT) for 4 min at 25 °C. The change in absorbance was converted to molar concentrations using the extinction coefficient of *p*-nitrophenol (Δε_412_ = 1.034 mM^–1^ cm^–1^). The initial rates of the reactions were measured in triplicate and plotted versus substrate concentration. The saturation enzyme kinetic traces were fitted to a standard Michaelis–Menten equation using GraphPad Prism 9.0 (GraphPad Software, La Jolla, CA), and the *k*_cat_, *K*_M_, and *k*_cat_/*K*_M_ values were calculated.

### Thermal Stability

The thermal stability was determined through differential scanning fluorimetry with a slight modification.^42,53^ Briefly, protein samples were diluted to 0.60 mg/mL in pH 7.4 buffer containing Sypro Orange dye (1000× dilution). Samples, in at least triplicate, were heated from 15 to 99 °C at a rate of 1 °C/min in a Bio-Rad C1000 Thermocycler and data were analyzed using the Bio-Rad CFX Manager software.

### Phylogenetic Analysis of LipN

The amino acid sequence of *Mm*LipN was aligned using Clustal Omega (EMBL EBI). A linear dendogram with decreasing ladderizing was then constructed using FastTree from NgPhylogeny.fr (Figure S1 and Tables S6 and S7).^68^ The catalytic triad amino acids were extracted from the alignment based on sequence conservation and the presence of the catalytic motif (G-x-S-x-G). The sequences used in the alignment were from *M. marinum* (WP_036455371.1), *Mycobacterium kansasii* (ORB86246.1), *Mycobacterium palustre* (WP_085077243.1), *M. tuberculosis* LipN (COV27923.1), *Mycobacterium avium* (WP_029248963.1), *Mycolicibacterium smegmatis* (WP_011728324.1), *Mycobacterium kubicae* (WP_085075701.1), *Mycobacteroides abscessus* (WP_145044121.1), *Bacillus cereus* (WP_098523648.1), *Sulfolobus acidocaldarius* (WP_011277970.1), *Listeria monocytogenes* (WP_099183763.1), *Schizosaccharomyces pombe* (NP_593998.1), *E. coli* str. K-12 (NP_415009.1), *Pseudomonas aeruginosa* PAO1 (NP_254071.1), *M. tuberculosis* LipW (NP_214731.1), *Streptomyces* multispecies (WP_003972012.1), *Shewanella oneidensis* (WP_011071097.1), *Dictyostelium discoideum* (XP_638888.1), *P. putida* (WP_110963922.1), *Pseudomonas fluorescens* (WP_150715891.1), *Deinococcus radiodurans* (WP_027480227.1), *Burkholderia multivorans* (WP_105844151.1), and *Burkholderia cenocepacia* (WP_060264533.1). Sequences for alignment were chosen based on protein BLAST analysis of *Mm*LipN and extracting unique protein sequences from model organisms with significant percent similarity (>20%).

## Supporting Information Available

Kinetic parameters, mutagenic oligonucleotides, and characterization data for newly synthesized fluorogenic ester substrates (PDF)

## Abbreviations and Symbols

DSF: differential scanning fluorimetry
IPTG: isopropyl β-d-1-thiogalactopyranoside
MOAME: fluorescein bis((2-methoxy)acetoxymethyl ether)
MOPS: 3-(*N*-morpholino)propanesulfonic acid
NMR: nuclear magnetic resonance spectroscopy
HRMS: high-resolution mass spectrometry
SDS–PAGE: sodium dodecyl sulfate–polyacrylamide gel electrophoresis
