# Supplementary material for: Sequence and Structural Motifs Controlling the Broad Substrate Specificity of the Mycobacterial Hormone-Sensitive Lipase LipN
Source: ACS Omega. 2023 Mar 30;8(14):13252–64. doi: 10.1021/acsomega.3c00534 (PMC10099132; doi:10.1021/acsomega.3c00534)
Supplement: Supplementary file 1 — ao3c00534_si_001.pdf [file ao3c00534_si_001.pdf]

Sequence and structural motifs controlling the broad substrate specificity of the mycobacterial hormone sensitive lipase LipN

Daniel E. Schemenauer,[a] Emily H. Pool,[a] Stephanie N. Raynor,[a] Gabriela P. Ruiz,[a] Leah M. Goehring,[a] Andrew J. Koelper,[a] Madeleine A. Wilson,[a] Anthony J. Durand Jr.,[a] Elexi C. Kourtoglou,[a] Erik M. Larsen,[a] Luke D. Lavis,[b] John J. Esteb,[a] Geoffrey C. Hoops,[a] and R. Jeremy Johnson [a,*]

[a]Department of Chemistry and Biochemistry, Butler University, Indianapolis, IN 46208

[b]Howard Hughes Medical Institute, Janelia Research Campus, Ashburn, VA

Running title: Substrate specificity of LipN

Supplemental Information

Supplemental Tables and Figures

Table S1:	Kinetic characterization of LipN with fluorogenic esters.	S3
Table S2:	Kinetic characterization of LipN with *p*-nitrophenyl esters.	S4
Table S3:	Biochemical characterization of active site and binding pocket variants of LipN.	S5
Table S4:	Biochemical characterization of Ser216 variants of LipN.	S6
Table S5:	Biochemical characterization of Trp146 variants of LipN.	S7
Table S6:	Substitutions in substrate binding and catalytic residues among hydrolases similar to LipN from *M. marinum.*	S9
Table S7:	Substitutions in bHSL motifs among hydrolases similar to LipN from *M. marinum*.	S9
Figure S1:	Phylogenetic relationship between *Mm*LipN and homologous serine hydrolases across various model organisms.	S10
	Supplemental Methods	S11-S13
Figures S2-S139	Supplemental NMR and HRMS for substrates **6**, **10-11**, **14-15**, **20**, **23-30**, **33**	S15-S154

Table S1: Kinetic characterization of LipN with fluorogenic esters.

	k_{cat} (s^{-1})	K_m (μM)	k_{cat}/K_m ($M^{-1}s^{-1}$)
**Alkyl Esters**			
**1**[a]	0.10 ± 0.038	0.22 ± 0.12	460000 ± 150000
**2**	0.18 ± 0.06	4.2 ± 0.5	42000 ± 15000
**3**	0.062 ± 0.029	6.2 ± 0.1	10000 ± 5000
**4**	0.024 ± 0.001	3.8 ± 0.5	6400 ± 1400
**5**	0.0074 ± 0.0047	4.4 ± 0.9	1700 ± 600
**6**	0.017 ± 0.014	9.5 ± 2.2	1800 ± 800
**Polar Esters**			
**7**	0.11 ± 0.12	2.6 ± 1.1	40000 ± 25000
**8**	0.093 ± 0.048	2.8 ± 0.5	34000 ± 9000
**9**	0.12 ± 0.12	3.0 ± 1.1	39000 ± 21000
**10**	0.0005 ± 0.0004	21 ± 4	26 ± 11
**11**	0.015 ± 0.008	3.1 ± 0.6	4900 ± 1400
**α/β Unsaturated Esters**			
**12**	0.020 ± 0.030	3.4 ± 1.7	6100 ± 4700
**13**	0.0093 ± 0.0050	0.032 ± 0.08	29000 ± 9000
**14**	0.0029 ± 0.0023	0.051 ± 0.19	5700 ± 2500
**15**	0.0061 ± 0.0047	1.8 ± 0.5	3300 ± 1400
**Cycloalkyl Esters**			
**16**	0.025 ± 0.017	3.0 ± 0.7	8300 ± 3100
**17**	0.0012 ± 0.0001	33.9 ± 7.71	29 ± 4
**18**	0.00070 ± 0.00060	29 ± 5	25 ± 11
**19**	0.0030 ± 0.0048	0.84 ± 0.60	3500 ± 3100
**20**	0.0030 ± 0.0001	0.033 ± 0.016	91000 ± 22000
**21**	0.0003 ± 0.0013	1.2 ± 1.7	310 ± 690
**Tertiary Esters**			
**22**	0.000024 ± 0.000030	0.71 ± 0.41	34 ± 23
**23**	0.000026 ± 0.000020	0.049 ± 0.027	530 ± 250
**24**	0.000011 ± 0.000030	0.87 ± 1.1	12 ± 19
**25**	0.0012 ± 0.0018	1.7 ± 1.1	670 ± 590
**26**	0.000058 ± 0.00014	13 ± 8	4.4 ± 5.4
**27**	0.0029 ± 0.0016	0.40 ± 0.10	7400 ± 2200
**28**	0.000014 ± 0.000050	0.084 ± 0.15	170 ± 330
**29**	0.00090 ± 0.0011	0.37 ± 0.20	2600 ± 1700
**30**	0.0011 ± 0.0018	1.7 ± 1.1	660 ± 590
**Fluorinated Esters**			
**31**	0.013 ± 0.004	1.1 ± 0.2	11000 ± 2000
**32**	0.00023 ± 0.00020	47 ± 8	4.8 ± 2.2
**33**	0.000037 ± 0.000030	0.37 ± 0.13	98 ± 43

[a]Kinetic constants for substrates **1–33** were determined by measuring the increase in fluorogenic enzyme substrate fluorescence over time. Data were fitted to a standard Michaelis-Menten equation to determine the values for k_{cat}, K_M, and k_{cat}/K_M. Kinetic measurements for each substrate were repeated at least three times and the values are given ± SD.

Table S2: Kinetic characterization of LipN with *p*-nitrophenyl esters.

Substrate	k_{cat} (s^{-1})	K_m (mM)	k_{cat}/K_m ($M^{-1}\ s^{-1}$)
**C2**[a]	94 ± 8	2.4 ± 1.2	39000 ± 10000
**C4**	190 ± 10	0.020 ± 0.009	9300000 ± 2100000
**C6**	15 ± 1	0.25 ± 0.07	59000 ± 8000
**C8**	1.1 ± 0.2	0.41 ± 0.33	2700 ± 1100
**C14**	0.28 ± 0.02	0.12 ± 0.05	2300 ± 400

[a]Kinetic constants for *p*-nitrophenyl substrates were determined by measuring the change in A_{412} due to ester hydrolysis. Data were fitted to a standard Michaelis-Menten equation to determine the values for k_{cat}, K_M, and k_{cat}/K_M. Kinetic measurements for each substrate were repeated at least three times and the values are given ± SD. Substrates represent different carbon chain lengths: *p*-nitrophenyl acetate (C2), *p*-nitrophenyl butyrate (C4), *p*-nitrophenyl valerate (C6), *p*-nitrophenyl octanoate (C8), and *p*-nitrophenyl myristate (C14).

Table S3: Biochemical characterization of active site and binding pocket variants of LipN.

LipN	k_{cat} (10^{-3} s^{-1}) [a]	K_m (μM)	k_{cat}/K_m ($M^{-1}s^{-1}$)	T_m (°C)[b]
Wild-type				52.0 ± 0.6
1	0.10 ± 0.038	0.22 ± 0.12	460000 ± 150000	
7	0.11 ± 0.12	2.6 ± 1.1	40000 ± 25000	
20	0.0030 ± 0.0001	0.033 ± 0.016	91000 ± 22000	
G144A				52.3 ± 0.3
1	0.017 ± 0.001	5.4 ± 0.5	3300 ± 200	
7	0.066 ± 0.024	60 ± 15	1100 ± 200	
20	0.0029 ± 0.0001	1.5 ± 0.4	2000 ± 300	
G145A				50.7 ± 0.3
1	0.045 ± 0.012	3.4 ± 1.3	13000 ± 3000	
7	0.18 ± 0.11	120 ± 60	1500 ± 600	
20	0.0019 ± 0.0005	0.30 ± 0.16	6200 ± 1800	
H154A				42.7 ± 0.7
1	0.030 ± 0.004	32 ± 4	940 ± 80	
7	0.28 ± 0.15	694 ± 422	410 ± 170	
20	0.0024 ± 0.0002	3.4 ± 0.4	700 ± 50	
D215A				49.0 ± 0.6
1	0.038 ± 0.012	2.5 ± 1.2	16000 ± 4000	
7	0.039 ± 0.017	3.3 ± 2.1	12000 ± 4000	
20	0.0023 ± 0.0003	0.49 ± 0.13	4600 ± 700	
S216G				63.0 ± 0.5
1	0.00070 ± 0.00027	21 ± 16	34 ± 15	
7	0.00018 ± 0.00006	22 ± 2	82 ± 4	
20	0.000021 ± 0.000003	3.6 ± 1.8	5.7 ± 1.5	
R246A				49.0 ± 0.6
1	0.054 ± 0.011	21 ± 4	2500 ± 370	
7	0.10 ± 0.02	150 ± 40	680 ± 100	
20	0.089 ± 0.010	120 ± 20	760 ± 80	
D269A				46.0 ± 0.6
1	0.0055 ± 0.0003	5.8 ± 0.4	960 ± 50	
7	0.032 ± 0.017	15 ± 8	2200 ± 800	
20	0.046 ± 0.0001	5.7 ± 1.9	1700 ± 300	
D312A				52.7 ± 0.3
1	0.19 ± 0.06	28 ± 7	6700 ± 1400	
7	0.31 ± 0.29	35 ± 27	9000 ± 5500	
20	0.0039 ± 0.0008	5.7 ± 1.9	4300 ± 800	
H342A				51.3 ± 0.3
1	0.0000032 ± 0.000001	0.92 ± 1.15	3.5 ± 2.2	
7	0.000053 ± 0.000017	20 ± 6	2.6 ± 0.6	
20	0.0000033 ± 0.0000033	0.31 ± 0.63	11 ± 12	

[a]Kinetic constants for substrates **1**, **7**, and **20** were determined by measuring the increase in fluorogenic enzyme substrate fluorescence over time. Data were fitted to a standard Michaelis-Menten equation to determine the values for k_{cat}, K_M, and k_{cat}/K_M. Kinetic measurements for each substrate were repeated at least three times and the values are given ± SD.

[b] Values for T_m were determined by following the change in Sypro Orange fluorescence with increasing temperature. Melting curves were repeated at least three times for each variant and the T_m values reported ± SD.

Table S4: Biochemical characterization of Ser216 variants of LipN.

LipN	k_{cat} (10^{-3} s^{-1}) [a]	K_m (μM)	k_{cat}/K_m ($M^{-1}s^{-1}$)	T_m (°C) [b]
Wild-type				52.0 ± 0.6
1	0.10 ± 0.038	0.22 ± 0.12	460000 ± 150000	
7	0.11 ± 0.12	2.6 ± 1.1	40000 ± 25000	
20	0.0030 ± 0.0001	0.033 ± 0.016	91000 ± 22000	
S216C				56.7 ± 0.2
1	0.0031 ± 0.0019	82 ± 68	37 ± 19	
7	0.0032 ± 0.0003	60 ± 9	53 ± 5	
20	0.000061 ± 0.000022	120 ± 70	0.53 ± 0.17	
S216G				63.0 ± 0.5
1	0.00070 ± 0.00027	21 ± 16	34 ± 15	
7	0.00018 ± 0.00006	22 ± 2	82 ± 4	
20	0.000021 ± 0.000003	3.6 ± 1.8	5.7 ± 1.5	
S216N				49.5 ± 1.3
1	0.00082 ± 0.0003	26 ± 3	31 ± 2	
7	0.0021 ± 0.0010	41 ± 33	51 ± 24	
20	0.00012 ± 0.00003	26 ± 15	15 ± 5	
S177A				57.2 ± 0.3
1	1.3 ± 0.1	1.1 ± 0.1	110000 ± 10000	
7	1.8 ± 0.1	6.4 ± 0.6	270000 ± 30000	
20	0.019 ± 0.001	2.0 ± 0.7	9400 ± 1700	
S226A				53.0 ± 0.5
1	3.6 ± 0.1	20 ± 2	180000 ± 20000	
7	2.3 ± 0.1	31 ± 4	73000 ± 5000	
20	0.017 ± 0.001	1.1 ± 0.1	15000 ± 1000	

[a]Kinetic constants for substrates **1**, **7**, and **20** were determined by measuring the increase in fluorogenic enzyme substrate fluorescence over time. Data were fitted to a standard Michaelis-Menten equation to determine the values for k_{cat}, K_M, and k_{cat}/K_M. Kinetic measurements for each substrate were repeated at least three times and the values are given ± SD.

[b] Values for T_m were determined by following the change in Sypro Orange fluorescence with increasing temperature. Melting curves were repeated at least three times for each variant and the T_m values reported ± SD.

Table S5: Biochemical characterization of Trp146 variants of LipN.

LipN	k_{cat} (10^{-3} s^{-1}) [a]	K_m (μM)	k_{cat}/K_m ($M^{-1}s^{-1}$)	T_m (°C)[b]
Wild-type				52.0 ± 0.6
1	0.10 ± 0.038	0.22 ± 0.12	460000 ± 150000	
7	0.11 ± 0.12	2.6 ± 1.1	40000 ± 25000	
20	0.0030 ± 0.0001	0.033 ± 0.016	91000 ± 22000	
W146A				40.0 ± 0.7
1	0.075 ± 0.006	2.0 ± 0.8	37000 ± 7000	
7	0.067 ± 0.004	1.1 ± 0.3	62000 ± 10000	
20	0.0013 ± 0.0001	0.71 ± 0.11	1800 ± 200	
W146C				45.0 ± 0.4
1	0.013 ± 0.006	23 ± 26	570 ± 340	
7	0.15 ± 0.01	13 ± 1	11000 ± 1000	
20	0.000061 ± 0.000022	120 ± 70	0.53 ± 0.17	
W146Q				45.0 ± 0.5
1	0.064 ± 0.004	1.4 ± 0.4	45000 ± 7000	
7	0.075 ± 0.0023	2.2 ± 0.3	34000 ± 2000	
20	0.0020 ± 0.0002	3.5 ± 1.3	550 ± 110	
W146S				33.0 ± 0.6
1	0.091 ± 0.007	23 ± 5	4000 ± 500	
7	0.17 ± 0.01	4.1 ± 0.5	42000 ± 3000	
20	0.0017 ± 0.0001	3.5 ± 0.4	500 ± 30	

[a]Kinetic constants for substrates **1**, **7**, and **20** were determined by measuring the increase in fluorogenic enzyme substrate fluorescence over time. Data were fitted to a standard Michaelis-Menten equation to determine the values for k_{cat}, K_M, and k_{cat}/K_M. Kinetic measurements for each substrate were repeated at least three times and the values are given ± SD.
[b] Values for T_m were determined by following the change in Sypro Orange fluorescence with increasing temperature. Melting curves were repeated at least three times for each variant and the T_m values reported ± SD.

Table S6: Substitutions in substrate binding and catalytic residues among hydrolases similar to LipN from *M. marinum*.

Organism	144	145	146	154	177	215	216	226	246	269	312	342	% Identity
M. marinum	G	G	W	H	S	D	S	S	R	D	D	H	-
M. kansasii				A	A			C					78
M. palustre					A			C					75
M. tuberculosis (LipN)					A			C					73
M. avium			F		A			A	V				64
M. smegmatis			F		A				I				53
M. kubicae			F		A				G				56
M. abscessus					A			A	A	A			53
B. cereus				M	A			T	T	Q			36
S. acidocaldarius			F	Y	A				A	Q			39
L. monocytogenes			F		A			T	A	S			36
S. pombe	S			D	A	A			T	I			30
E. coli			F					A	L				26
P. aeruginosa					A			C	L				37
M. tuberculosis (LipW)			Y	D	A			A	M	A			37
S. multispecies						A		T	V	A			35
S. oneidensis		C	F		A			C	M	T			31
D. discoideum			F	V	A			A	I	F			32
P. putida			F		A				V	M			44
P. fluorescens			F		A				V	M			44
D. radiodurans			F		A			A	A	R			44
B. multivorans			F		A			A	V	A			40
B. cenocepacia			F		A			A	V	A			40

[a]The amino acid sequence of *Mm*LipN was aligned using Clustal Omega (EMBL EBI). The sequences used in the alignment were from *Mycobacterium marinum* (WP_036455371.1), *Mycobacterium kansasii* (ORB86246.1), *Mycobacterium palustre* (WP_085077243.1), *Mycobacterium tuberculosis* LipN (COV27923.1), *Mycobacterium avium* (WP_029248963.1), *Mycolicibacterium smegmatis* (WP_011728324.1), *Mycobacterium kubicae* (WP_085075701.1), *Mycobacteroides abscessus* (WP_145044121.1), *Bacillus cereus* (WP_098523648.1), *Sulfolobus acidocaldarius* (WP_011277970.1), *Listeria monocytogenes* (WP_099183763.1), *Schizosaccharomyces pombe* (NP_593998.1), *Escherichia coli* str. K-12 (NP_415009.1), *Pseudomonas aeruginosa* PAO1 (NP_254071.1), *Mycobacterium tuberculosis* LipW (NP_214731.1), *Streptomyces* multi-species (WP_003972012.1), *Shewanella oneidensis* (WP_011071097.1), *Dictyostelium discoideum* (XP_638888.1), *Pseudomonas putida* (WP_110963922.1), *Pseudomonas fluorescens* (WP_150715891.1), *Deinococcus radiodurans* (WP_027480227.1), *Burkholderia multivorans* (WP_105844151.1), *Burkholderia cenocepacia* (WP_060264533.1). Sequences for alignment were chosen based on protein BLAST analysis of *Mm*LipN and extracting unique protein sequences from model organisms with significant percent similarity (> 20%).
[b]Catalytic triad residues are highlighted in blue.

Table S7: Substitutions in bHSL motifs among hydrolases similar to LipN from *M. marinum*.

**Organism**	**142**	**143**	**144**	**145**	**146**	**214**	**215**	**216**	**217**	**218**	**% Identity**
M. marinum	H	G	G	G	W	G	D	S	A	G	-
M. kansasii											78
M. palustre											75
M. tuberculosis (LipN)											73
M. avium					F						64
M. smegmatis					F						53
M. kubicae					F						56
M. abscessus											53
B. cereus									V		36
S. acidocaldarius					F						39
L. monocytogenes					F				V		36
S. pombe		S	S				A				30
E. coli					F						26
P. aeruginosa											37
M. tuberculosis (LipW)		A			Y						37
S. multispecies							A		V		35
S. oneidensis				C	F						31
D. discoideum		S			F				V		32
P. putida					F						44
P. fluorescens					F						44
D. radiodurans					F						44
B. multivorans					F						40
B. cenocepacia					F						40

[a]The amino acid sequence of *Mm*LipN was aligned using Clustal Omega (EMBL EBI). The sequences used in the alignment were from *Mycobacterium marinum* (WP_036455371.1), *Mycobacterium kansasii* (ORB86246.1), *Mycobacterium palustre* (WP_085077243.1), *Mycobacterium tuberculosis* LipN (COV27923.1), *Mycobacterium avium* (WP_029248963.1), *Mycolicibacterium smegmatis* (WP_011728324.1), *Mycobacterium kubicae* (WP_085075701.1), *Mycobacteroides abscessus* (WP_145044121.1), *Bacillus cereus* (WP_098523648.1), *Sulfolobus acidocaldarius* (WP_011277970.1), *Listeria monocytogenes* (WP_099183763.1), *Schizosaccharomyces pombe* (NP_593998.1), *Escherichia coli* str. K-12 (NP_415009.1), *Pseudomonas aeruginosa* PAO1 (NP_254071.1), *Mycobacterium tuberculosis* LipW (NP_214731.1), *Streptomyces* multi-species (WP_003972012.1), *Shewanella oneidensis* (WP_011071097.1), *Dictyostelium discoideum* (XP_638888.1), *Pseudomonas putida* (WP_110963922.1), *Pseudomonas fluorescens* (WP_150715891.1), *Deinococcus radiodurans* (WP_027480227.1), *Burkholderia multivorans* (WP_105844151.1), *Burkholderia cenocepacia* (WP_060264533.1). Sequences for alignment were chosen based on protein BLAST analysis of *Mm*LipN and extracting unique protein sequences from model organisms with significant percent similarity (> 20%).
[b]HGGGW motif shaded gray.

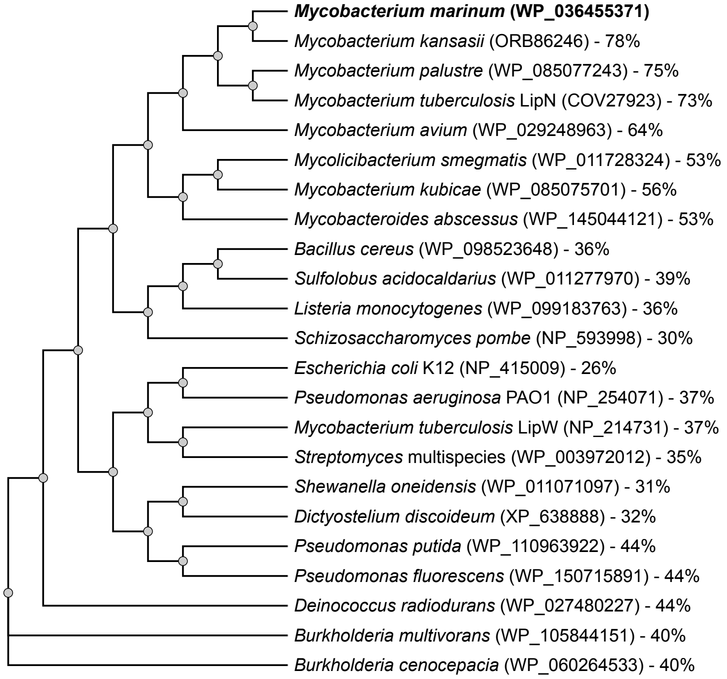

Figure S1: Phylogenetic relationship between *Mm*LipN and homologous serine hydrolases across various model organisms. The amino acid sequence of LipN was aligned with the 22 homologues and a cladogram of the aligned proteins was constructed with percent similarities from Clustal Omega. A linear dendogram with decreasing ladderizing was then constructed using FastTree from NgPhylogeny.fr. Sequences for alignment were chosen based on protein BLAST analysis of *Mm*LipN and extracting unique protein sequences from model organisms with significant percent similarity (> 20%). Detailed sequence analysis is given in Tables S6 and S7.

Supplemental Methods:

The syntheses of compounds (**1, 2, 12, 16, 22**)[1], (**7, 8, 9, 13, 17, 18, 19, 21, 31, 32**)[2], and (**3, 4, 5**)[3] have been previously described. The synthesis of the common starting material for divergent synthesis, Fluorescein bis(chloromethyl ether), has been previously described[4]. Carboxylic acids were obtained from Sigma Aldrich, Alfa Aesar, Enamine, Matrix Scientific, and Chem-Bridge. Compounds **24** and **30** are each mixtures of stereoisomers that presumably resemble the isomeric mixture of the corresponding carboxylic acid starting material in distribution. All other chemicals were purchased from Sigma Aldrich and used without further purification. Nuclear magnetic resonance (NMR) spectra were obtained using a Bruker Biospin Avance III HD 400 operating at 400.19 MHz for ^{1}H and 100.64 MHz for ^{13}C. High resolution mass spectrometry (HRMS) was performed with electrospray ionization (ESI) by the Mass Spectrometry facility at the Department of Chemistry, Indiana University using an Agilent 1200 HPLC-6130 MSD mass spectrometer.

The following procedure is representative for the synthesis of all compounds.
Synthesis of Fluorescein bis((2-methyl)propionyloxymethyl ether) (**6**). Fluorescein bis(chloromethyl ether) (30.0 mg, 69.9 μmol, 1.0 Eq), (2-methyl)propanoic acid (20.1 mg, 280 μmol, 4.0 Eq.), and Cs_2CO_3 (91.1 mg, 279.6 μmol, 4.0 Eq.) were dissolved in dry CH_3CN (1 mL). Molecular sieves (100 mg) were added, and the reaction was covered in foil and allowed to stir for 24 h at ambient temperature. The reaction mixture was adsorbed onto celite and purified via silica column chromatography.

Fluorescein bis((2-methyl)propionyloxymethyl ether) (**6**)
Data for **6**: (79%, white solid). ^{1}H NMR ($CDCl_3$, 400 MHz): δ = 8.03 (d, 1H), 7.7-7.6 (m, 2H), 7.16 (d, 1H), 6.96 (m, 2H), 6.73 (m, 4H), 5.79 (s, 4H), 2.67-2.56 (sept., 2H), 1.15 (d, 12 H) ppm. ^{13}C NMR ($CDCl_3$, 100 MHz): δ = 175.8, 169.2, 158.4, 152.8, 152.2, 135.1, 129.8, 129.3, 126.6, 125.1, 123.9, 113.1, 112.7, 103.5, 84.9, 82.5, 33.9, 18.7 ppm. HRMS (ESI): calc'd for MH^+ $C_{30}H_{29}O_9$: 533.1806; found: 533.1801.

Fluorescein bis(3-(2-methoxyethoxy)propionyloxymethyl ether) (**10**)
Data for **10**: (27%, white solid). ^{1}H NMR ($CDCl_3$, 400 MHz): δ = 8.03 (d, 1H), 7.61-7.70 (m, 2H), 7.16 (d, 1H), 6.96 (t, 2H), 6.73 (d, 4H), 5.80 (s, 4H), 3.77 (t, 4H), 3.53 (dq, 8H), 3.34 (s, 6H), 2.69 (t, 4H) ppm. ^{13}C NMR ($CDCl_3$, 100 MHz): δ = 170.4, 169.2, 158.4, 153.0, 152.2, 135.1, 129.9, 129.4, 126.6, 125.2, 123.9, 113.3, 112.8. 103.6, 85.0, 82.4, 71.8, 70.4, 66.2, 59.1, 35.0 ppm. HRMS (ESI): calc'd for MNa^+ $C_{34}H_{36}O_{13}Na$: 675.2054; found: 675.2041.

Fluorescein bis(cyanoacetyloxymethyl ether) (**11**)
Data for **11**: (6%, white solid). ^{1}H NMR ($CDCl_3$, 400 MHz): δ = 8.03 (d, 1H), 7.7-7.6 (m, 2H), 7.16 (d, 1H), 6.75 (m, 2H), 6.72 (m, 4H), 5.87(s, 4H), 3.55 (s, 4H) ppm. ^{13}C NMR ($CDCl_3$, 100 MHz): δ = 169.5, 162.0, 157.8, 152.9, 152.3, 135.3, 129.9, 129.5, 126.6, 125.2, 123.9, 113.9, 112.7, 112.2, 103.7, 86.5, 82.8, 24.8 ppm. HRMS (ESI): calc'd for MH^+ $C_{28}H_{19}N_2O_9$: 527.1085; found:. 527.1091.

Fluorescein bis(crotonyloxymethyl ether) (**14**)
Data for **14**: (65%, white solid). ^{1}H NMR ($CDCl_3$, 400 MHz): δ = 8.03 (d, 1H), 7.70-7.61 (m, 2H), 7.16 (d, J = 8.05 Hz, 1H), 7.14-7.05 (m, 2H), 6.98 (s, 2H), 6.74 (s, 4H), 5.89 (d, J = 16. 8 Hz, 2H), 5.85 (s, 4H), 1.91 (d, J = 7.02 Hz, 6H) ppm. ^{13}C NMR ($CDCl_3$, 100 MHz): δ = 169.2, 164.9, 158.5, 152.9, 152.2, 147.2, 135.1, 129.9, 129.4, 126.7, 125.2, 123.9, 121.7, 113.2, 112.7, 103.5, 84.9, 82.5, 18.2 ppm. HRMS (ESI): calc'd for MH^+ $C_{30}H_{25}O_9$: 529.1498; found: 529.1516.

Fluorescein bis((3-methyl)propargyloxymethyl ether) (**15**)
Data for **15**: (68%, white solid). ^{1}H NMR ($CDCl_3$, 400 MHz): δ = 8.03 (d, 1H), 7.7-7.6 (m, 2H), 7.15 (d, 1H), 6.98 (m, 2H), 6.75 (d, 4H), 5.83 (s, 4H), 2.01 (s, 6H) ppm. ^{13}C NMR ($CDCl_3$, 100 MHz): δ = 170.6,

169.2, 158.2, 153.0, 152.2, 135.1, 129.9, 129.4, 126.5, 125.1, 123.9, 113.6, 112.7, 103.7, 88.1, 85.6, 82.3, 71.7, 3.9 ppm. HRMS (ESI): calc'd for MH^+ $C_{30}H_{21}O_9$: 525.1180; found: 525.1183.

Fluorescein bis((5-oxazolyl)carboxymethyl ether) (**20**)
Data for **20**: (59%, white solid). ^{1}H NMR ($CDCl_3$, 400 MHz): δ = 8.08 (s, 2H), 8.05 (d, 1H), 7.89 (s, 2H), 7.72-7.64 (m, 2H), 7.17 (d, 1H), 7.04 (d, 2H), 6.83-6.77 (m, 4H), 6.04 (s, 4H) ppm. ^{13}C NMR ($CDCl_3$, 100 MHz): δ = 169.1, 158.1, 156.1, 154.0, 152.8, 152.2, 141.8, 135.2, 134.9, 130.0, 129.6, 126.5, 125.2, 123.8, 113.8, 112.7, 103.7, 85.6, 82.1 ppm. HRMS (ESI): calc'd for MH^+ $C_{30}H_{19}N_2O_{11}$: 583.0989; found: 583.0981.

Fluorescein bis((((1-(2-pyridinyl))cyclopropyl)carboxymethyl ether) (**23**)
Data for **23**: (56%, white solid). ^{1}H NMR ($CDCl_3$, 400 MHz): δ = 8.53 (d, 2H), 8.04 (d, 1H), 7.7-7.6 (m, 2H), 7.58 (dd, 2H), 7.41 (d, 2H), 7.17-7.13 (dd, 2H), 7.16 (s, 1H), 6.69 (m, 2H), 6.63-6.61 (m, 4H), 5.75 (s, 4H), 1.74-1.71 (m, 4H), 1.53-1.52 ppm. ^{13}C NMR ($CDCl_3$, 100 MHz): δ = 172.3, 169.0, 158.1, 157.0, 152.7, 151.9, 148.8, 136.0, 134.9, 129.7, 129.0, 126.4, 124.9, 124.7, 123.7, 122.0, 113.0, 112.5, 103.5, 85.0, 82.2, 30.4, 17.5 ppm. HRMS (APCI): calc'd for MH+ = $C_{40}H_{31}N_2O_9$: 683.2029; found: 683.2024.

Fluorescein bis(((2,2-dichloro-1-methyl)cyclopropyl)carboxymethyl ether) (**24**)
Data for **24**: (57%, white solid). ^{1}H NMR ($CDCl_3$, 400 MHz): δ = 8.04 (d, 1H), 7.7-7.6 (m, 2H), 7.18 (d, 1H), 6.98 (m, 2H), 6.75 (m, 4H), 5.92-5.82 (d, 4H), 2.31 (m, 2H), 1.60 (s, 6H), 1.47 (m, 2H) ppm. ^{13}C NMR ($CDCl_3$, 100 MHz): δ = 169.2, 168.2, 158.3, 152.9, 152.2, 135.2, 129.9, 129.5, 126.6, 125.2, 123.9, 113.59, 113.56, 112.8, 112.6, 103.8, 103.7, 86.2, 82.3, 62.4, 35.3, 31.0, 18.1 ppm. HRMS (APCI): calc'd for MH^+ = $C_{32}H_{25}Cl_4O_9$: 693.0247; found =693.0253; calc'd for MNa^+ = $C_{32}H_{24}Cl_4O_9Na$: 715.0067; found = 715.0071.

Fluorescein bis(((1-cyano)cyclopropyl)carboxymethyl ether) (**25**)
Data for **25**: (78%, white solid). ^{1}H NMR ($CDCl_3$, 400 MHz): δ = 7.98 (d, 1H), 7.6-7.5 (m, 2H), 7.12 (d, 1H), 6.95 (m, 2H), 6.71 (m, 4H), 5.79 (s, 4H), 1.75-1.65 (m, 8H) ppm. ^{13}C NMR ($CDCl_3$, 100 MHz): δ = 169.2, 167.0, 158.1, 152.8, 152.2, 135.3, 130.0, 129.6, 126.6, 125.2, 123.9, 117.9, 113.9, 112.7, 112.4, 104.0, 103.1, 86.5, 82.2, 19.9, 13.4 ppm. HRMS (APCI): calc'd for MH^+ = $C_{32}H_{23}N_2O_9$: 579.1398, found: 579.1399; calc'd for MNa^+ for $C_{32}H_{22}N_2O_9Na$: 601.1218, found: 601.1218

Fluorescein bis(((1-phenyl)cyclopropyl)carboxymethyl ether) (**26**)
Data for **26**: (29%, white solid). ^{1}H NMR ($CDCl_3$, 400 MHz): δ = 8.05 (d, 1H), 7.7-7.6 (m, 2H), 7.29-7.26 (m, 10H), 7.16 (d, 1H), 6.78 (m, 2H), 6.68-6.58 (m, 4H), 5.70 (s, 4H), 1.67-1.63 (m, 4H), 1.28-1.26 (m, 4H) ppm. ^{13}C NMR ($CDCl_3$, 100 MHz): δ = 173.4, 169.3, 158.4, 153.0, 152.1, 138.6, 135.1, 130.5, 129.9, 129.2, 128.3, 127.5, 126.6, 125.2, 123.9, 113.2, 112.8, 104.0, 85.4, 82.4, 29.2, 17.2 ppm. HRMS (APCI): calc'd for MH+ = $C_{42}H_{33}O_9$: 681.2119; found: 681.2125.

Fluorescein bis(((1-hydroxy)cyclopropyl)carboxymethyl ether)) (**27**)
Data for **27**: (43%, white solid). ^{1}H NMR ($CDCl_3$, 400 MHz): δ = 8.03 (d, 1H), 7.7-7.6 (m, 2H), 7.16 (d, 1H), 6.92 (m, 2H), 6.73 (s, 4H), 5.84 (m, 4H), 2.97 (s, 2H), 1.36-1.33 (m, 4H), 1.24-1.21 (m, 4H) ppm. ^{13}C NMR ($CDCl_3$, 100 MHz): δ = 174.0, 169.2, 158.2, 152.87, 152.2, 135.2, 130.0, 129.5, 126.6, 125.2, 123.9, 113.5, 112.6, 103.5, 85.6, 82.3, 54.9, 17.4 ppm. HRMS (APCI): calc'd for MH^+ = $C_{30}H_{25}O_{11}$: 561.1391; found = 561.1392; calc'd for MNa^+ = $C_{30}H_{24}O_{11}Na$: 583.1211; found = 583.1212.

Fluorescein bis(((1-methanesulfonyl)cyclopropyl)carboxymethyl ether) (**28**)
Data for **28**: (30%, white solid). ^{1}H NMR ($CDCl_3$, 400 MHz): δ = 8.04 (d, 1H), 7.7-7.6 (m, 2H), 7.17 (d, 1H), 6.96 (m, 2H), 6.75 (m, 4H), 5.88 (s, 4H), 3.17 (s, 6H), 1.89-1.86 (m, 4H), 1.73-1.70 (m, 4H) ppm. ^{13}C NMR ($CDCl_3$, 100 MHz): δ = 169.1, 166.47, 158.0, 152.6, 152.2, 135.3, 130.1, 129.7, 126.6, 125.3, 123.9, 114.0, 112.6, 103.8, 86.2, 82.1, 42.9, 41.1, 16.3 ppm. HRMS (ESI): calc'd for MH^+ = $C_{32}H_{29}O_{13}S_2$: 685.1044; found: 685.1047; calc'd for MNa^+ = $C_{32}H_{28}O_{13}S_2Na$: 707.0864; found: 707.0867.

Fluorescein bis(cyclopropyl)carboxymethyl ether) (**29**)
Data for **29**: (21%, white solid). ^{1}H NMR ($CDCl_3$, 400 MHz): δ = 8.03 (d, 1H), 7.7-7.6 (m, 2H), 7.17 (d, 1H), 6.98 (m, 2H), 6.74 (d, 4H), 5.78 (s, 4H), 1.70-1.63 (m, 2H), 1.08-1.06 (m, 4H), 0.96-0.93 (m, 4H) ppm. ^{13}C NMR ($CDCl_3$, 100 MHz): δ = 174.0, 169.5, 158.7, 153.2, 152.5, 135.4, 130.1, 129.6, 126.9, 125.4, 124.1, 113.5, 113.0, 103.8, 85.1, 82.7, 13.1, 9.5 ppm. HRMS (ESI): calc'd for MH+ = $C_{30}H_{25}O_9$: 529.1493; found: 529.1494.

Fluorescein bis(((2,2-dimethyl)cyclopropyl)carboxymethyl ether) (**30**)
Data for **30**: (73%, white solid). ^{1}H NMR ($CDCl_3$, 400 MHz): δ = 8.03 (d, 1H), 7.7-7.6 (m, 2H), 7.17 (d, 1H), 6.98 (m, 2H), 6.74 (m, 4H), 5.80-5.75 (dd, 4H), 1.56-1.53 (m, 2H), 1.21 (s, 6H), 1.17 (s, 6H), 1.17-1.15 (m, 2H), 0.95-0.92 (m, 2H) ppm. ^{13}C NMR ($CDCl_3$, 100 MHz): δ = 171.5, 169.1, 158.3, 152.8, 152.1, 134.9, 129.7, 129.2, 126.5, 125.0, 123.7, 113.0, 112.9, 112.7, 112.6, 103.4, 103.3, 84.6, 82.4, 26.7, 26.4, 24.3, 22.8, 18.6 ppm. HRMS (ESI): calc'd for MH+ = $C_{34}H_{33}O_9$: 585.2119; found: 585.2121.

Fluorescein bis(((1-trifluoromethyl)cyclopropyl)carboxymethyl ether) (**33**)
Data for **33**: (39%, white solid). ^{1}H NMR ($CDCl_3$, 400 MHz): δ = 8.05 (d, 1H), 7.7-7.6 (m, 2H), 7.18 (d, 1H), 6.98 (m, 2H), 6.75 (s, 4H), 5.84 (m, 4H), 1.50 (s, 4H), 1.42 (m, 4H) ppm. ^{13}C NMR ($CDCl_3$, 100 MHz): δ = 169.1, 167.4, 158.2, 152.7, 152.1, 135.1, 129.8, 129.3, 126.5, 125.4, 125.1, 123.8, 122.7, 113.6, 112.6, 103.8, 85.8, 82.2, 27.0-26.0 (q), 13.80/13.79 ppm. HRMS (ESI): calc'd for MH+ = $C_{32}H_{23}F_6O_9$: 665.1241; Found: 665.1245.

References for Supplemental Methods:

1. Tian, Lin; Yang, Yunlei; Wysocki, Laura M.; Arnold, Alma C; Hu, Amy; Ravichandran, Balaji; Sternson, Scott M.; Looger, Loren L. & Lavis, Luke D.; Selective esterase-ester pair for targeting small molecules with cellular specificity. *Proc. Natl. Acad. Sci. U. S. A.* **109** 4756–4761 (2012)
2. Ellis, E. E.; Adkins, C. T.; Galovska, N. M.; Lavis, L. D. & Johnson, R. J.; Decoupled roles for the atypical, bifurcated binding pocket of the ybfF hydrolase. *ChemBioChem* **14**, 1134-1144 (2013).
3. Hedge, Matthew K; Gehring, Alexandra M; Adkins, Chinessa T; Weston, Leigh A.; Lavis, Luke D. & Johnson, R. Jeremy; The structural basis for the narrow substrate specificity of an acetyl esterase from *Thermotoga maritima*. *Biochim. Biophys. Acta* **1824** 1024–1030 (2012)
4. Bassett, Braden; Waibel, Brent; White, Alex; Hansen, Heather; Stephens, Dominique; Koelper, Andrew; Larsen, Erik M.; Kim, Charles; Glanzer, Adam; Lavis, Luke D.; Hoops, Geoffrey C. & Johnson, R. Jeremy; Measuring the global substrate specificity of Mycobacterial serine hydrolases using a library of fluorogenic ester substrates. *ACS Infect. Dis.* **4** 904-911 (2018)

Figure S2

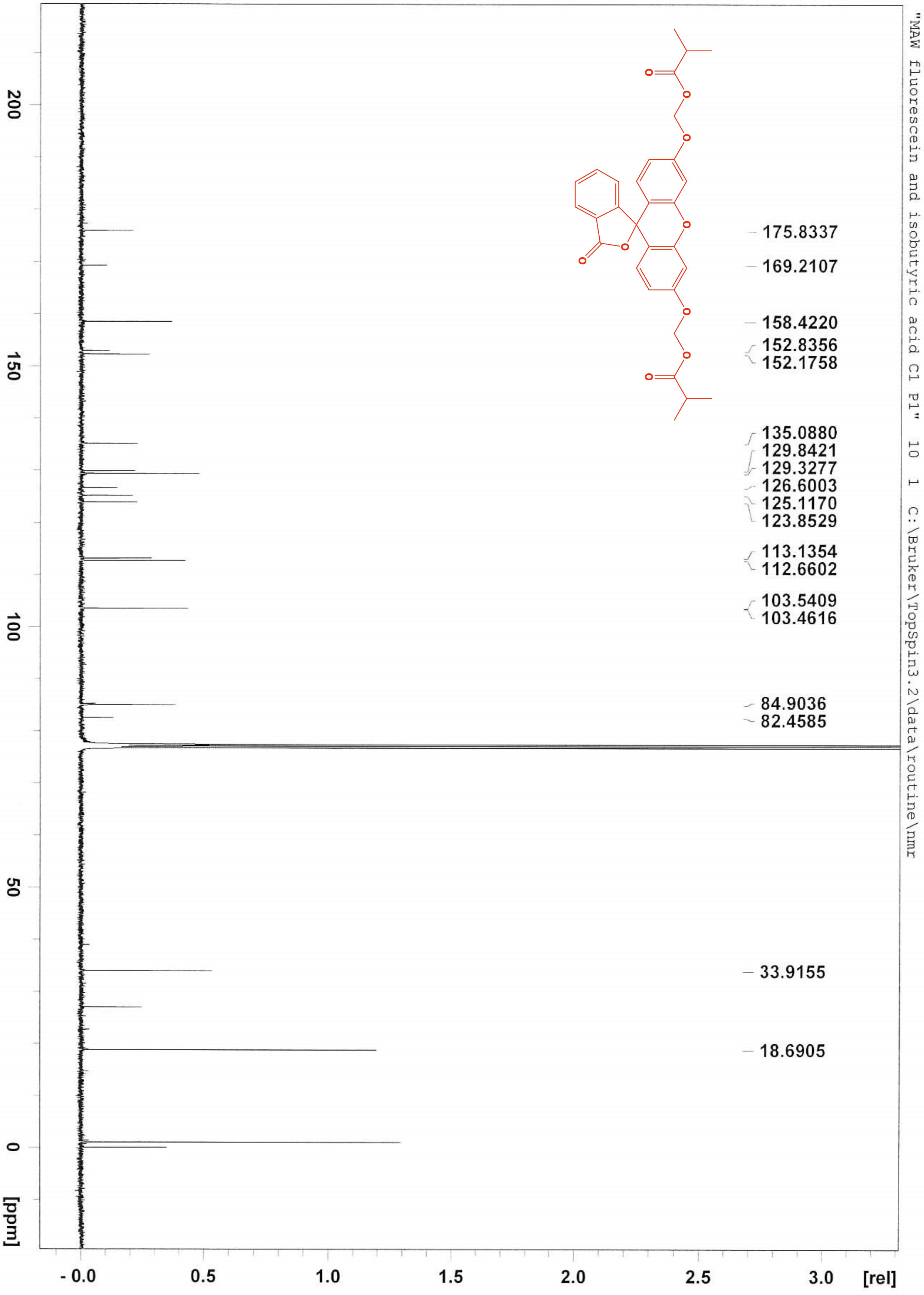

175.8337
169.2107
158.4220
152.8356
152.1758
135.0880
129.8421
129.3277
126.6003
125.1170
123.8529
113.1354
112.6602
103.5409
103.4616
84.9036
82.4585
33.9155
18.6905
"MAW fluorescein and isobutyric acid C1 P1" 10 1 C:\Bruker\TopSpin3.2\data\routine\nmr
200
150
100
50
0
[ppm]
- 0.0
0.5
1.0
1.5
2.0
2.5
3.0
[rel]

Figure S3

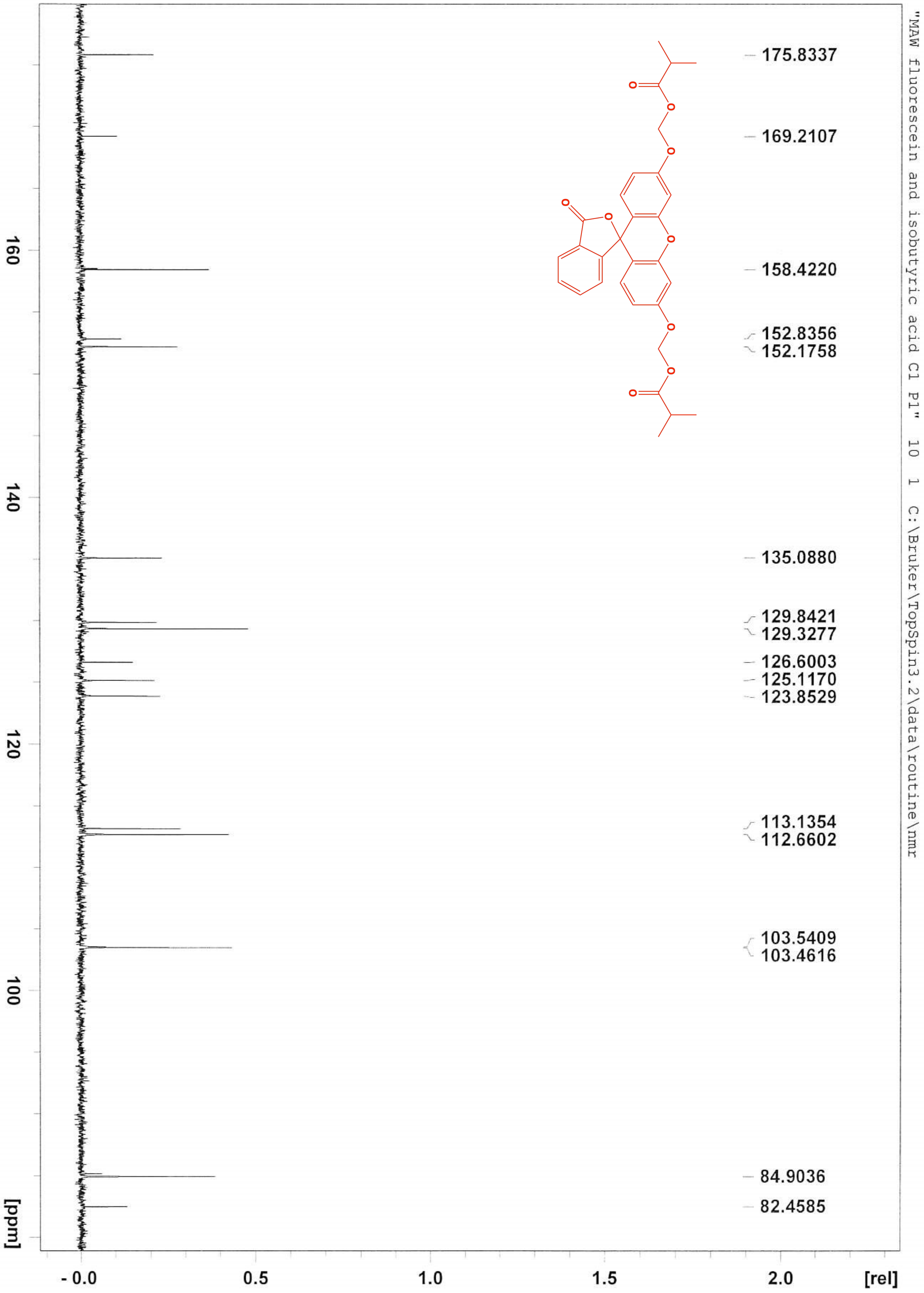
"MAW fluorescein and isobutyric acid C1 P1" 10 1 C:\Bruker\TopSpin3.2\data\routine\nmr
175.8337
169.2107
158.4220
152.8356
152.1758
135.0880
129.8421
129.3277
126.6003
125.1170
123.8529
113.1354
112.6602
103.5409
103.4616
84.9036
82.4585
160
140
120
100
[ppm]
- 0.0
0.5
1.0
1.5
2.0
[rel]

Figure S4

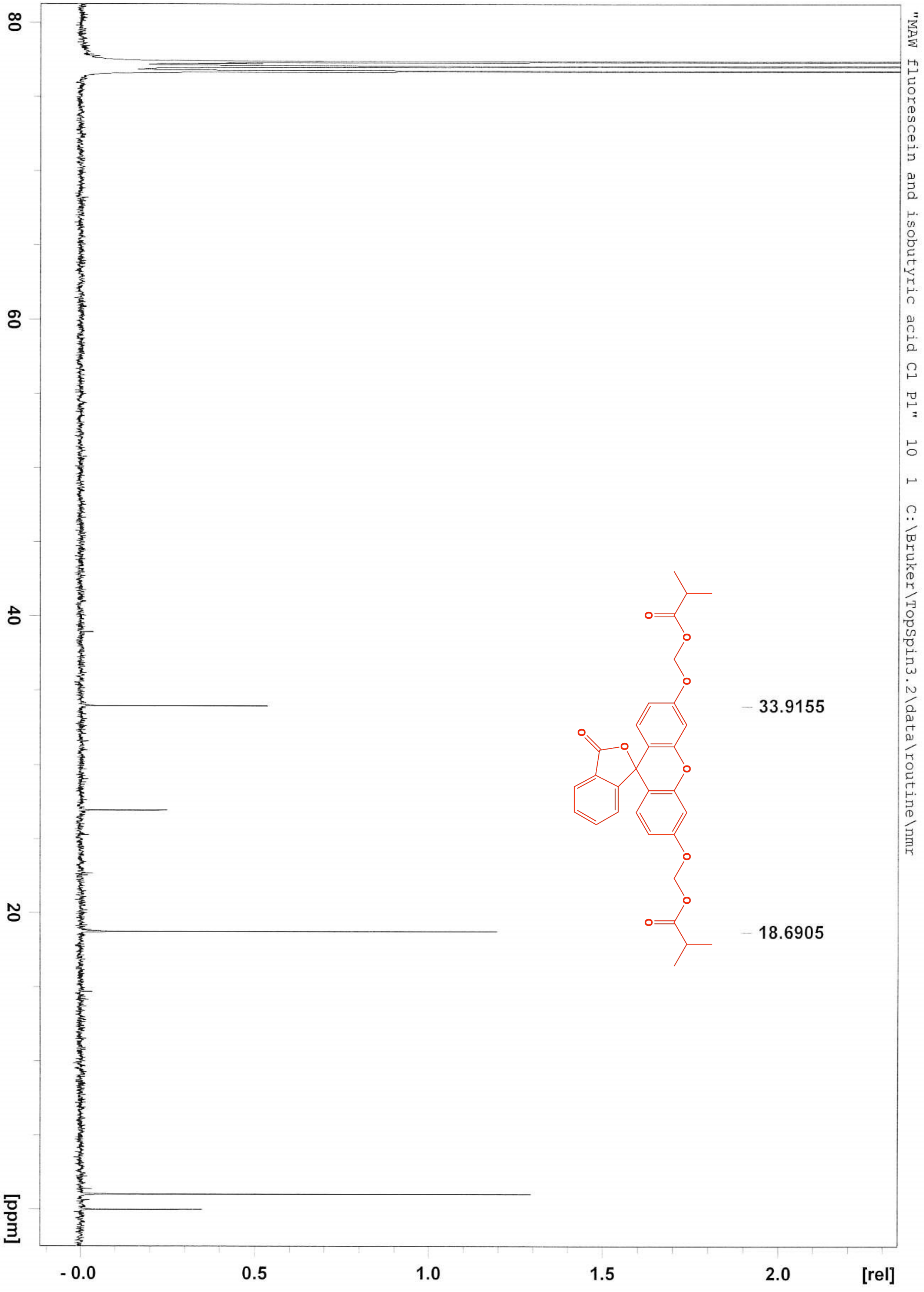
"MAW fluorescein and isobutyric acid C1 P1" 10 1 C:\Bruker\TopSpin3.2\data\routine\nmr
33.9155
18.6905
80
60
40
20
[ppm]
- 0.0
0.5
1.0
1.5
2.0
[rel]

Figure S5

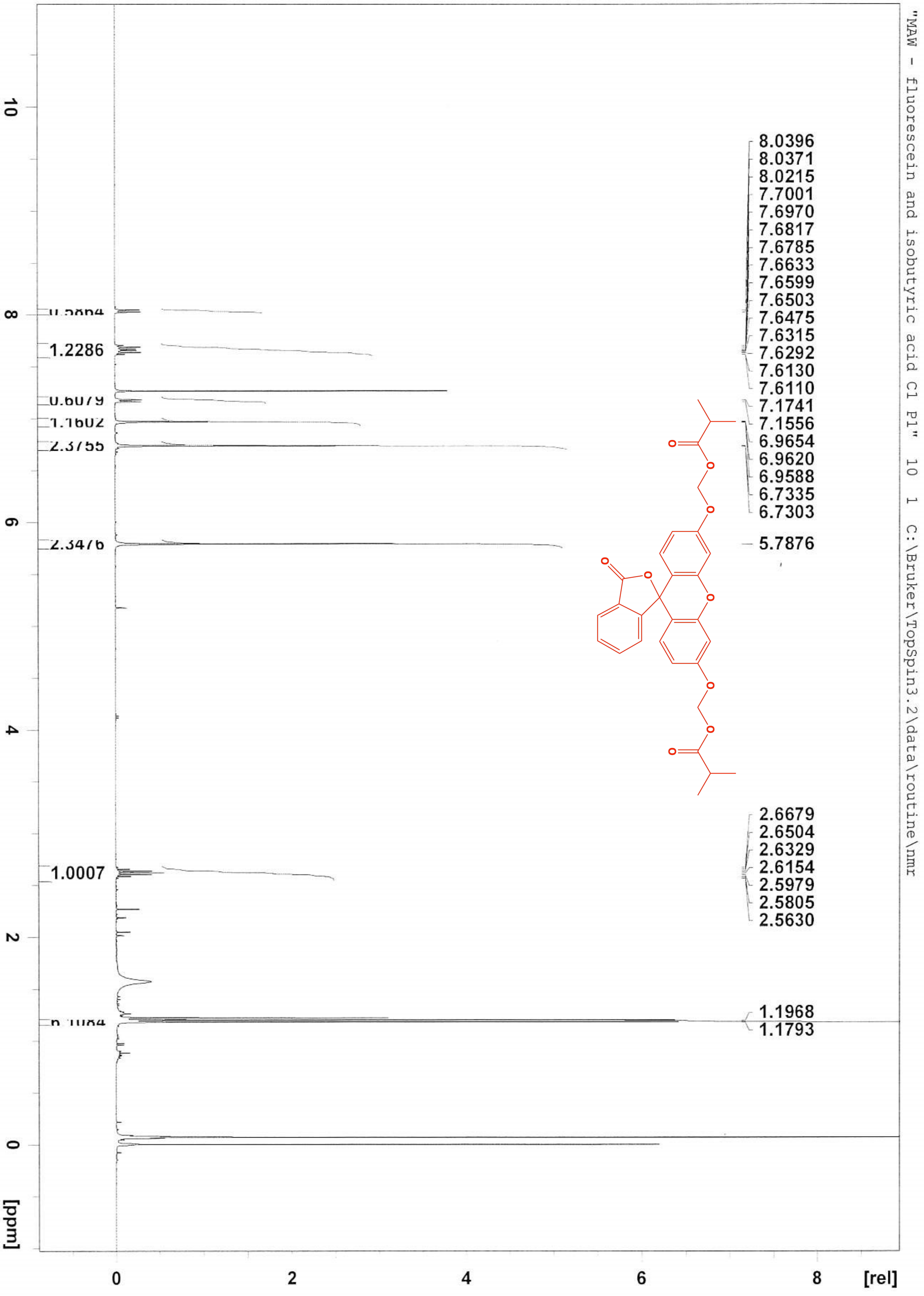
"MAW - fluorescein and isobutyric acid C1 P1" 10 1 C:\Bruker\TopSpin3.2\data\routine\nmr
8.0396
8.0371
8.0215
7.7001
7.6970
7.6817
7.6785
7.6633
7.6599
7.6503
7.6475
7.6315
7.6292
7.6130
7.6110
7.1741
7.1556
6.9654
6.9620
6.9588
6.7335
6.7303
5.7876
2.6679
2.6504
2.6329
2.6154
2.5979
2.5805
2.5630
1.1968
1.1793
1.2286
1.0007
[ppm]
[rel]

Figure S6

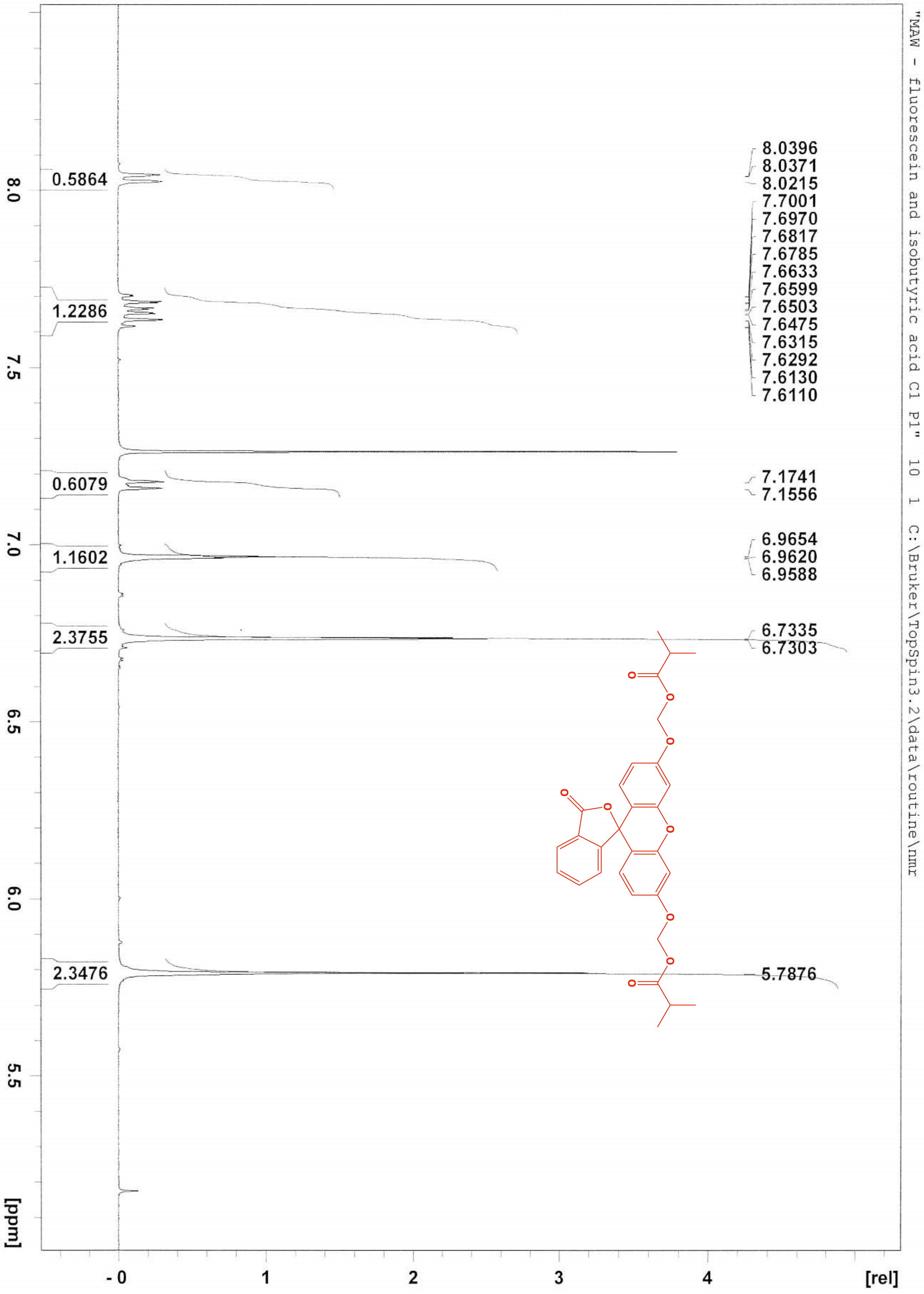

"MAW - fluorescein and isobutyric acid C1 P1" 10 1 C:\Bruker\TopSpin3.2\data\routine\nmr
8.0396
8.0371
8.0215
7.7001
7.6970
7.6817
7.6785
7.6633
7.6599
7.6503
7.6475
7.6315
7.6292
7.6130
7.6110
7.1741
7.1556
6.9654
6.9620
6.9588
6.7335
6.7303
5.7876
0.5864
1.2286
0.6079
1.1602
2.3755
2.3476
8.0
7.5
7.0
6.5
6.0
5.5
[ppm]
- 0
1
2
3
4
[rel]

Figure S7

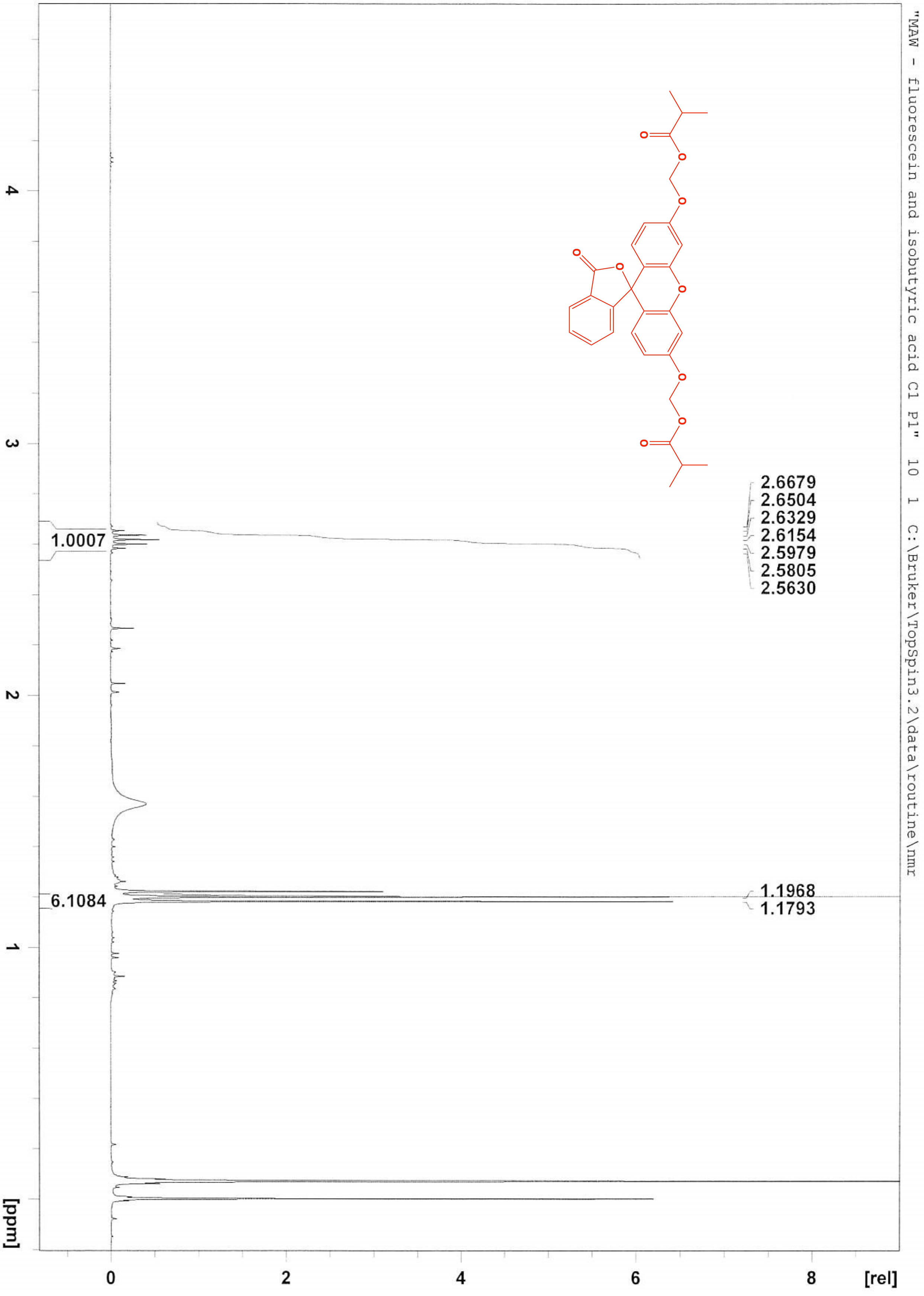
"MAW - fluorescein and isobutyric acid C1 P1" 10 1 C:\Bruker\TopSpin3.2\data\routine\nmr
2.6679
2.6504
2.6329
2.6154
2.5979
2.5805
2.5630
1.1968
1.1793
1.0007
6.1084
[ppm]
[rel]

Figure S8

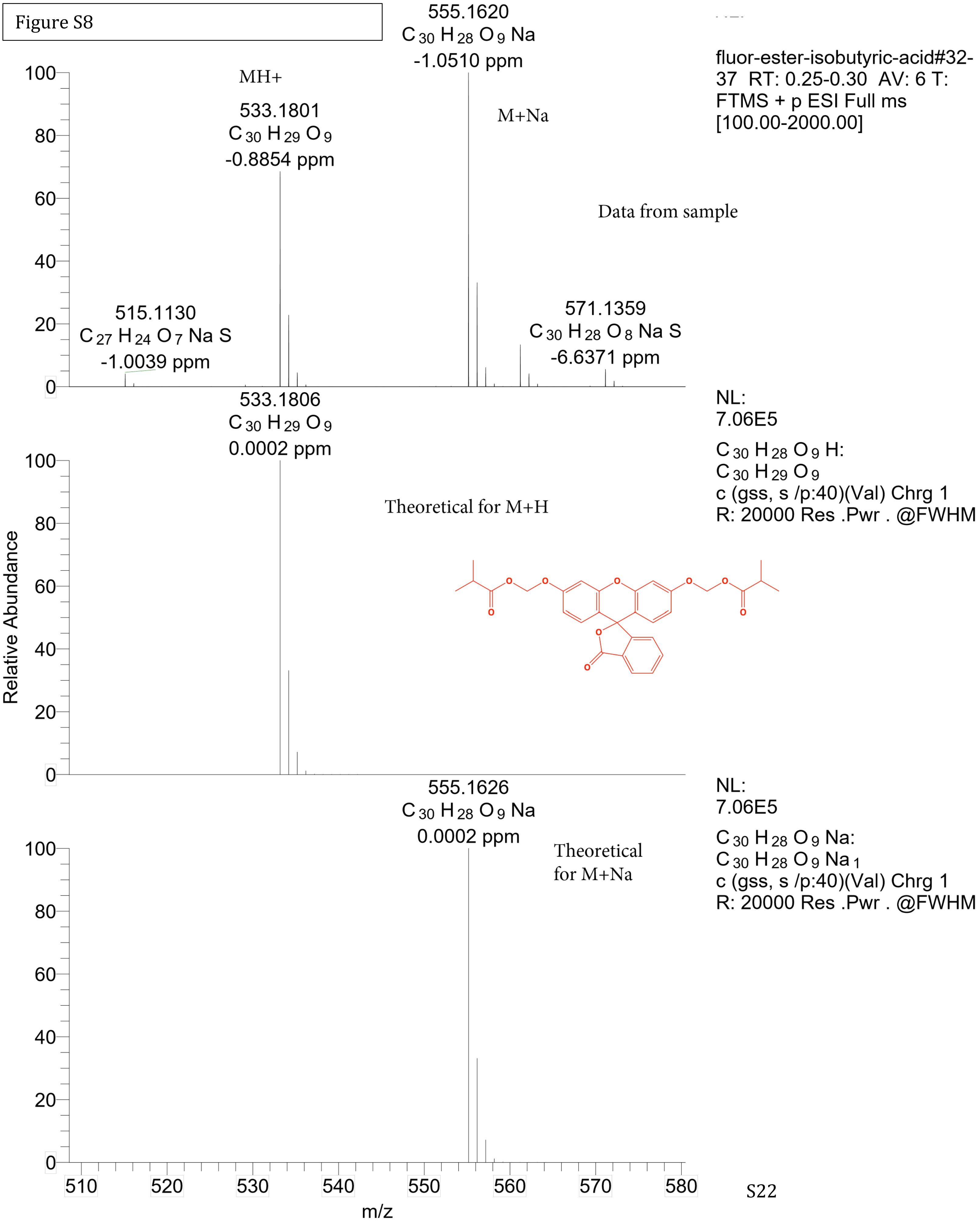

555.1620
C 30 H 28 O 9 Na
-1.0510 ppm
fluor-ester-isobutyric-acid#32-37 RT: 0.25-0.30 AV: 6 T: FTMS + p ESI Full ms [100.00-2000.00]
MH+
533.1801
C 30 H 29 O 9
-0.8854 ppm
M+Na
Data from sample
515.1130
C 27 H 24 O 7 Na S
-1.0039 ppm
571.1359
C 30 H 28 O 8 Na S
-6.6371 ppm
NL:
7.06E5
533.1806
C 30 H 29 O 9
0.0002 ppm
C 30 H 28 O 9 H:
C 30 H 29 O 9
c (gss, s /p:40)(Val) Chrg 1
R: 20000 Res .Pwr . @FWHM
Theoretical for M+H
Relative Abundance
555.1626
C 30 H 28 O 9 Na
0.0002 ppm
NL:
7.06E5
Theoretical
for M+Na
C 30 H 28 O 9 Na:
C 30 H 28 O 9 Na 1
c (gss, s /p:40)(Val) Chrg 1
R: 20000 Res .Pwr . @FWHM
m/z

[illegible]

Figure S9

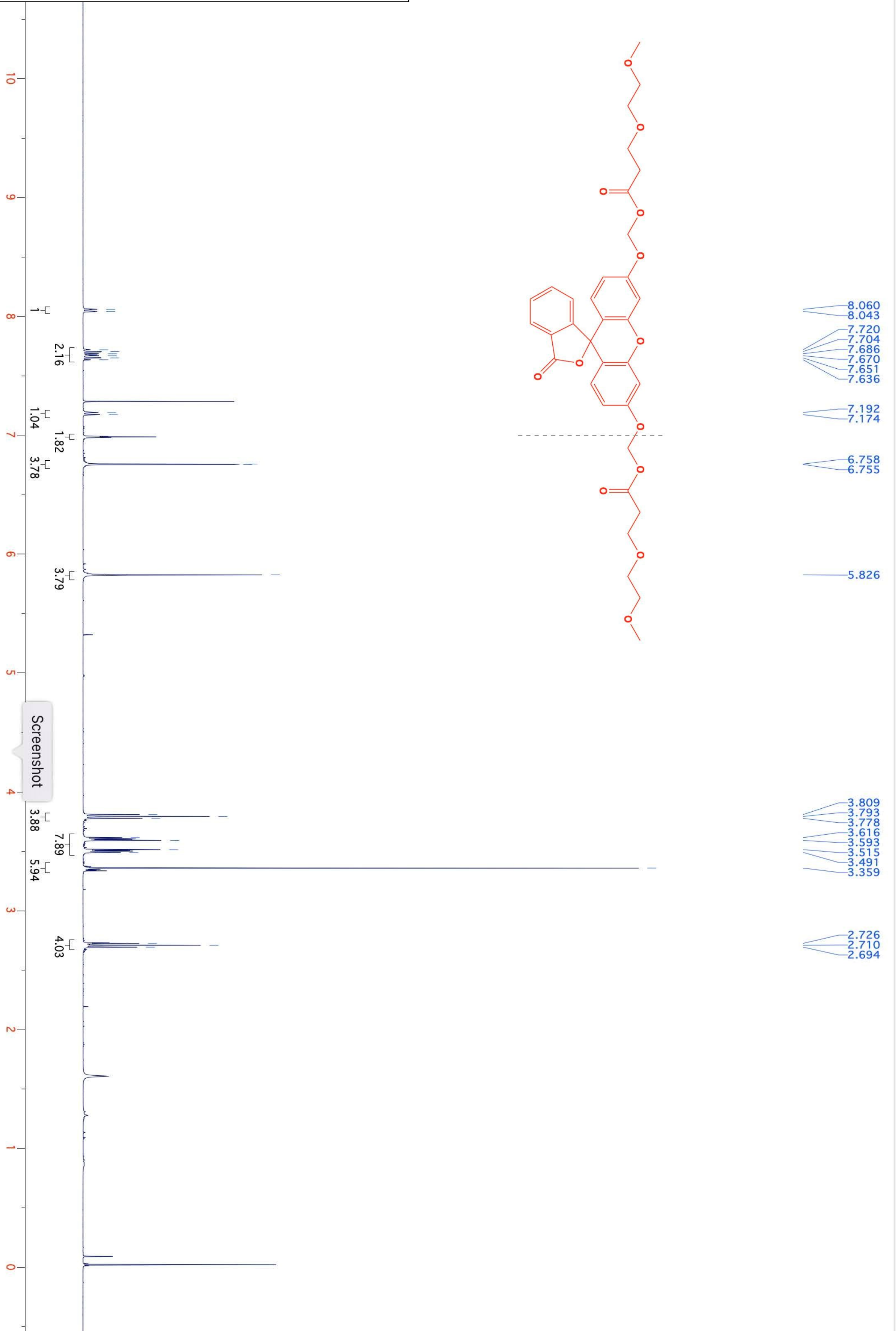
8.060
8.043
7.720
7.704
7.686
7.670
7.651
7.636
7.192
7.174
6.758
6.755
5.826
3.809
3.793
3.778
3.616
3.593
3.515
3.491
3.359
2.726
2.710
2.694
1
2.16
1.04
1.82
3.78
3.79
3.88
7.89
5.94
4.03
Screenshot

Figure S10

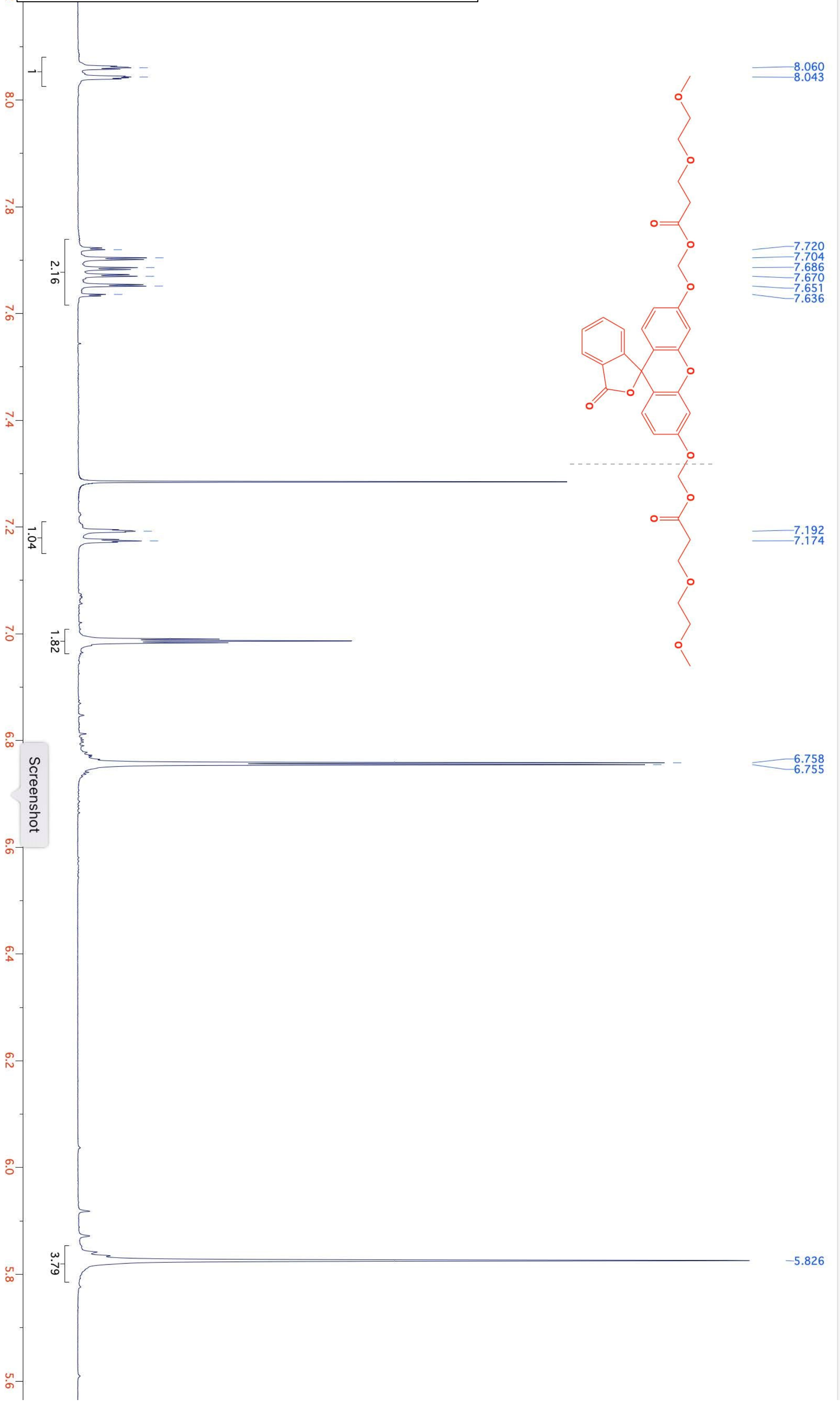

ppm
8.2
8.0
7.8
7.6
7.4
7.2
7.0
6.8
6.6
6.4
6.2
6.0
5.8
5.6
1
2.16
1.04
1.82
3.79
8.060
8.043
7.720
7.704
7.686
7.670
7.651
7.636
7.192
7.174
6.758
6.755
5.826
Screenshot

Figure S11

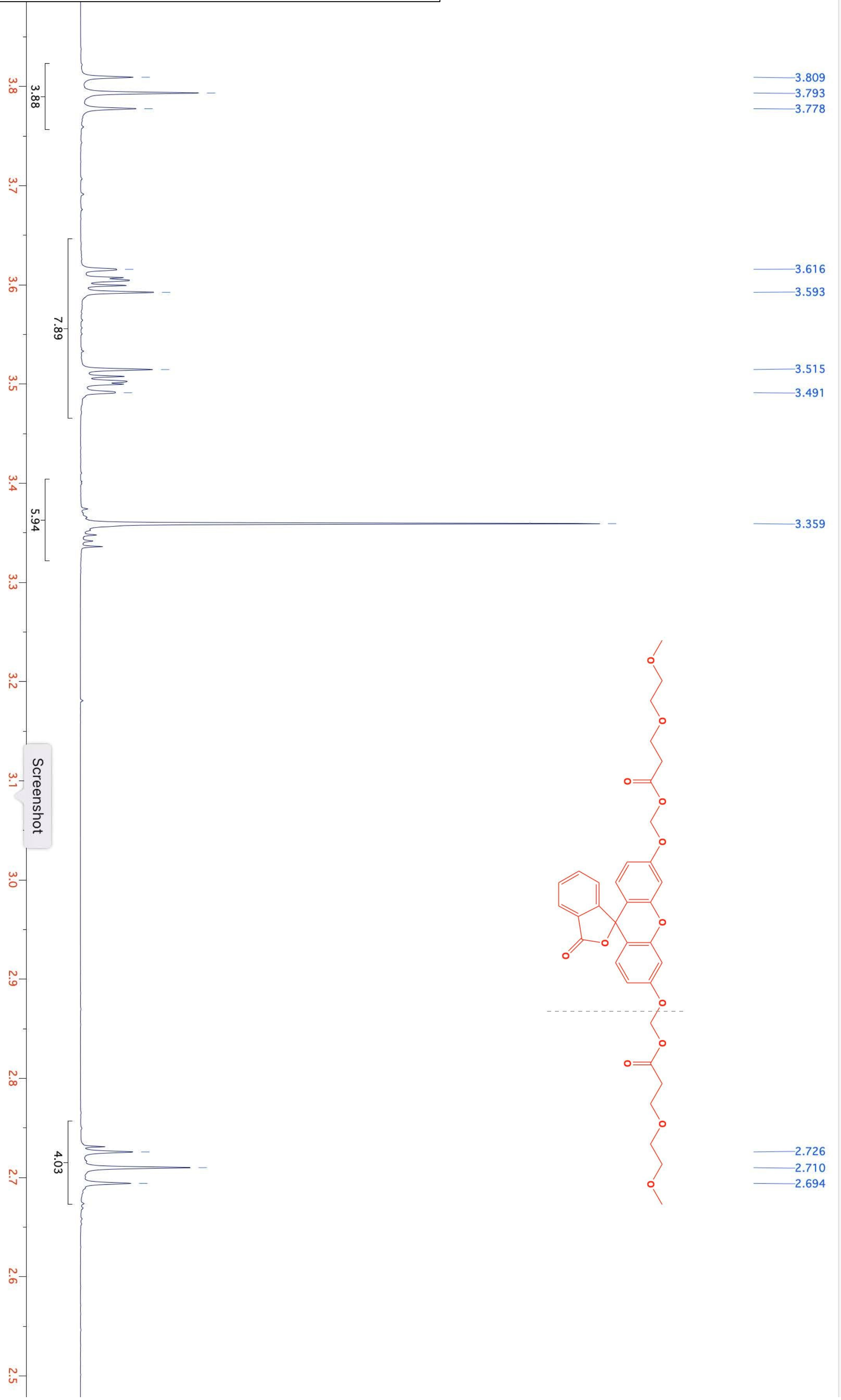
3.809
3.793
3.778
3.616
3.593
3.515
3.491
3.359
2.726
2.710
2.694
3.88
7.89
5.94
4.03
3.8
3.7
3.6
3.5
3.4
3.3
3.2
3.1
3.0
2.9
2.8
2.7
2.6
2.5

Figure S12

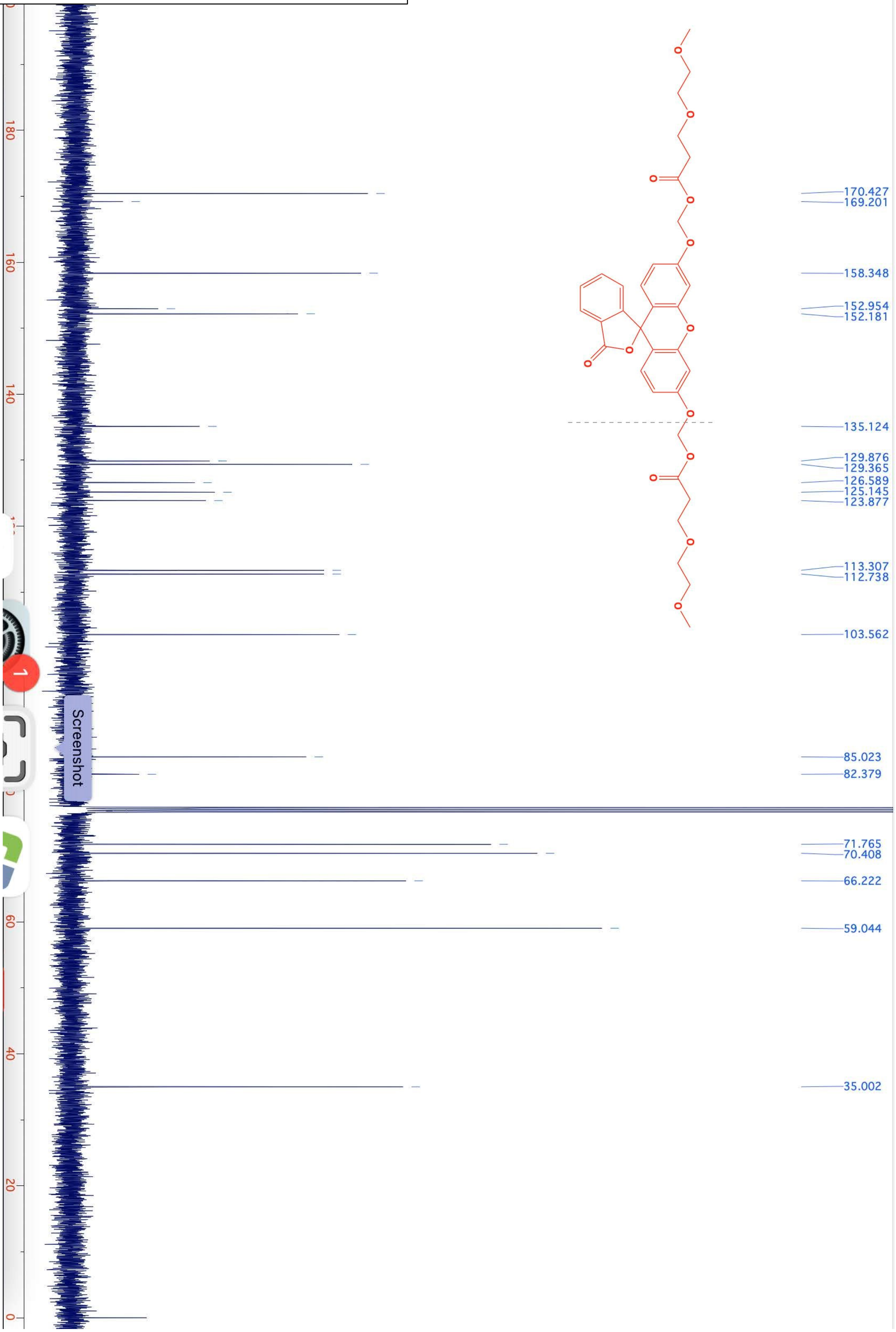
170.427
169.201
158.348
152.954
152.181
135.124
129.876
129.365
126.589
125.145
123.877
113.307
112.738
103.562
85.023
82.379
71.765
70.408
66.222
59.044
35.002
180
160
140
60
40
20
0

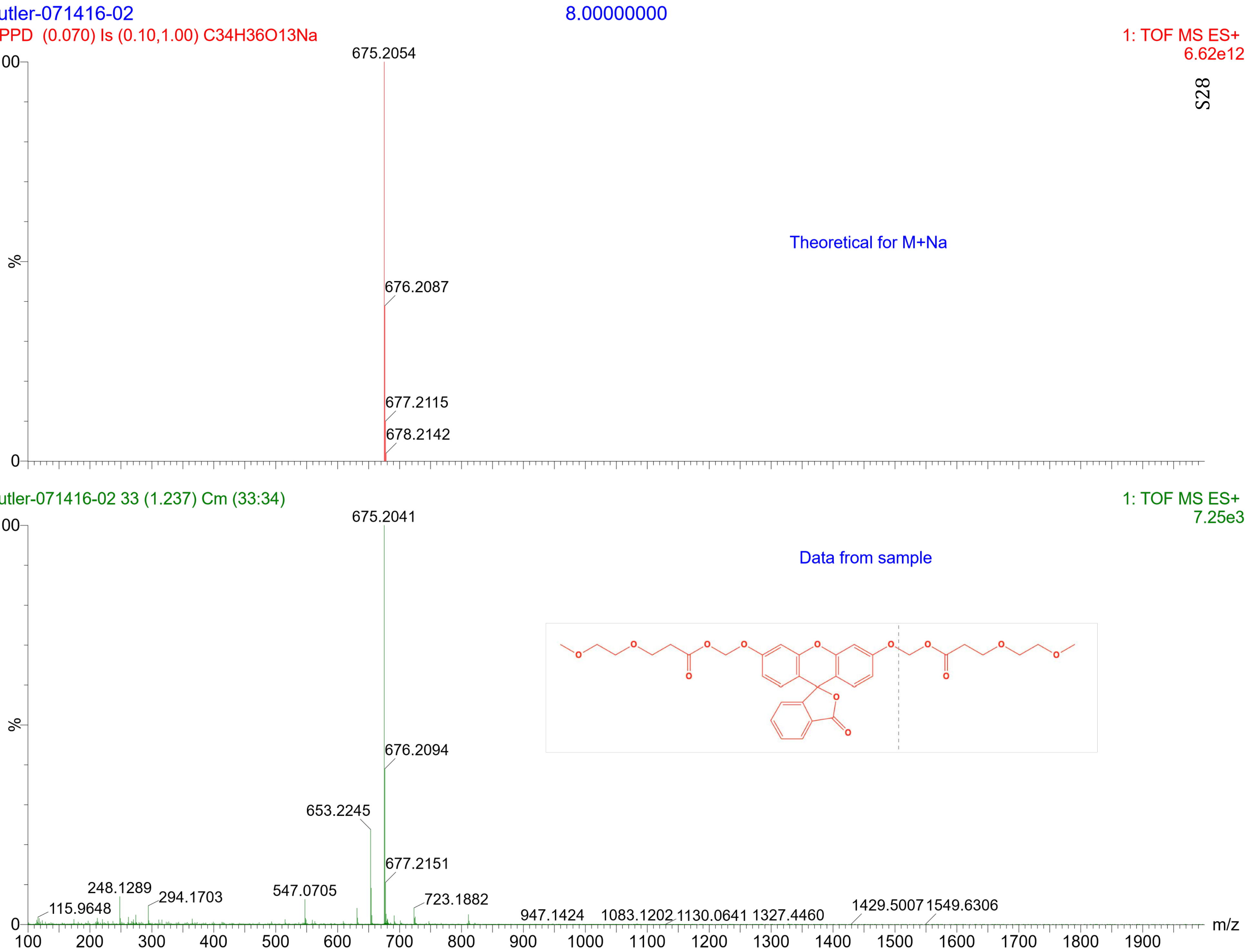

butler-071416-02
8.00000000
TPPD (0.070) Is (0.10,1.00) C34H36O13Na
1: TOF MS ES+
6.62e12
675.2054
676.2087
677.2115
678.2142
Theoretical for M+Na
100
%
0
butler-071416-02 33 (1.237) Cm (33:34)
1: TOF MS ES+
7.25e3
675.2041
Data from sample
676.2094
653.2245
677.2151
248.1289
294.1703
547.0705
723.1882
115.9648
947.1424
1083.1202
1130.0641
1327.4460
1429.5007
1549.6306
100
%
0
100 200 300 400 500 600 700 800 900 1000 1100 1200 1300 1400 1500 1600 1700 1800 1900
m/z

Figure S13

Figure S14

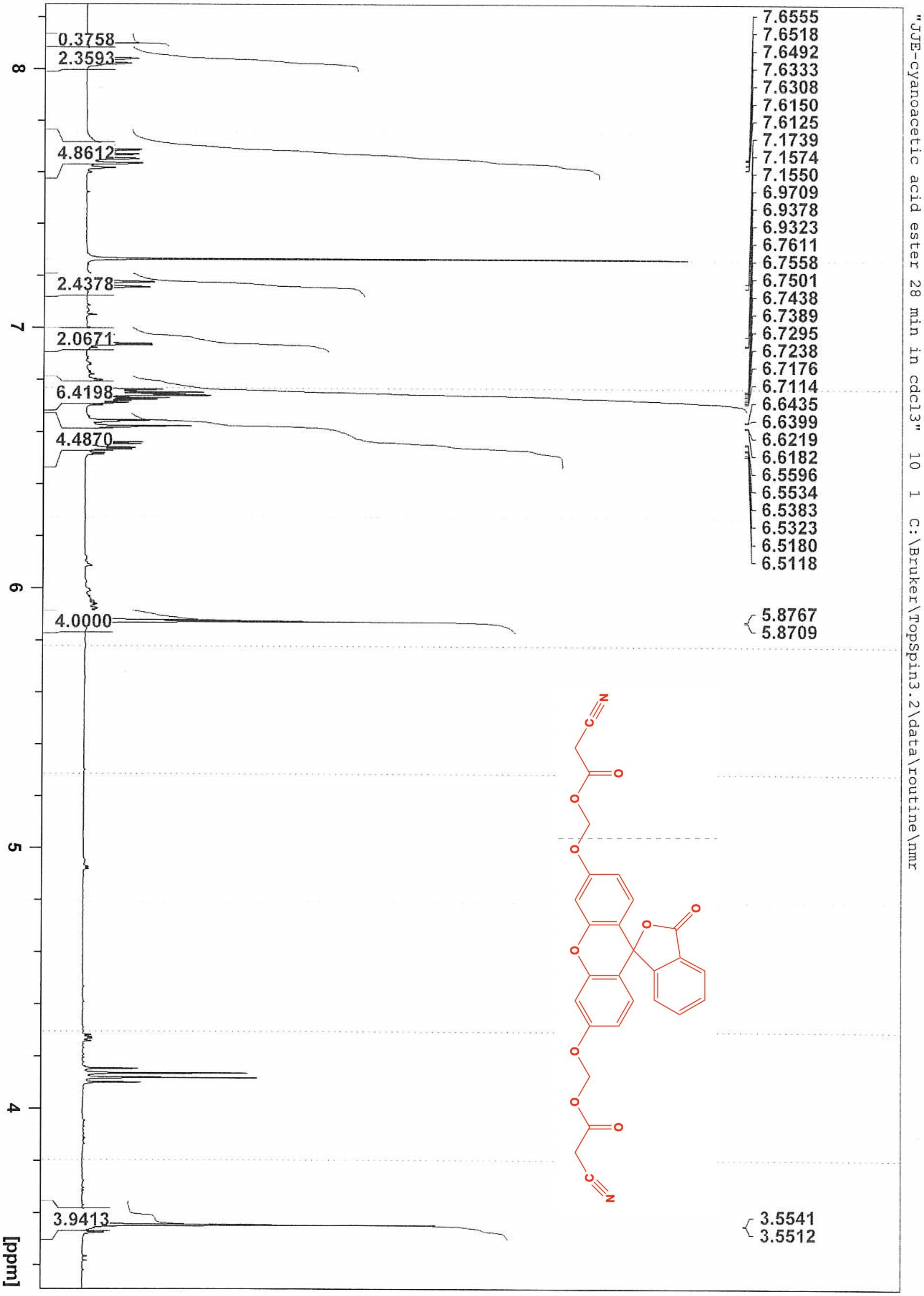
"JJE-cyanoacetic acid ester 28 min in cdcl3" 10 1 C:\Bruker\TopSpin3.2\data\routine\nmr
7.6555
7.6518
7.6492
7.6333
7.6308
7.6150
7.6125
7.1739
7.1574
7.1550
6.9709
6.9378
6.9323
6.7611
6.7558
6.7501
6.7438
6.7389
6.7295
6.7238
6.7176
6.7114
6.6435
6.6399
6.6219
6.6182
6.5596
6.5534
6.5383
6.5323
6.5180
6.5118
5.8767
5.8709
3.5541
3.5512
0.3758
2.3593
4.8612
2.4378
2.0671
6.4198
4.4870
4.0000
3.9413
8
7
6
5
4
[ppm]

Figure S15

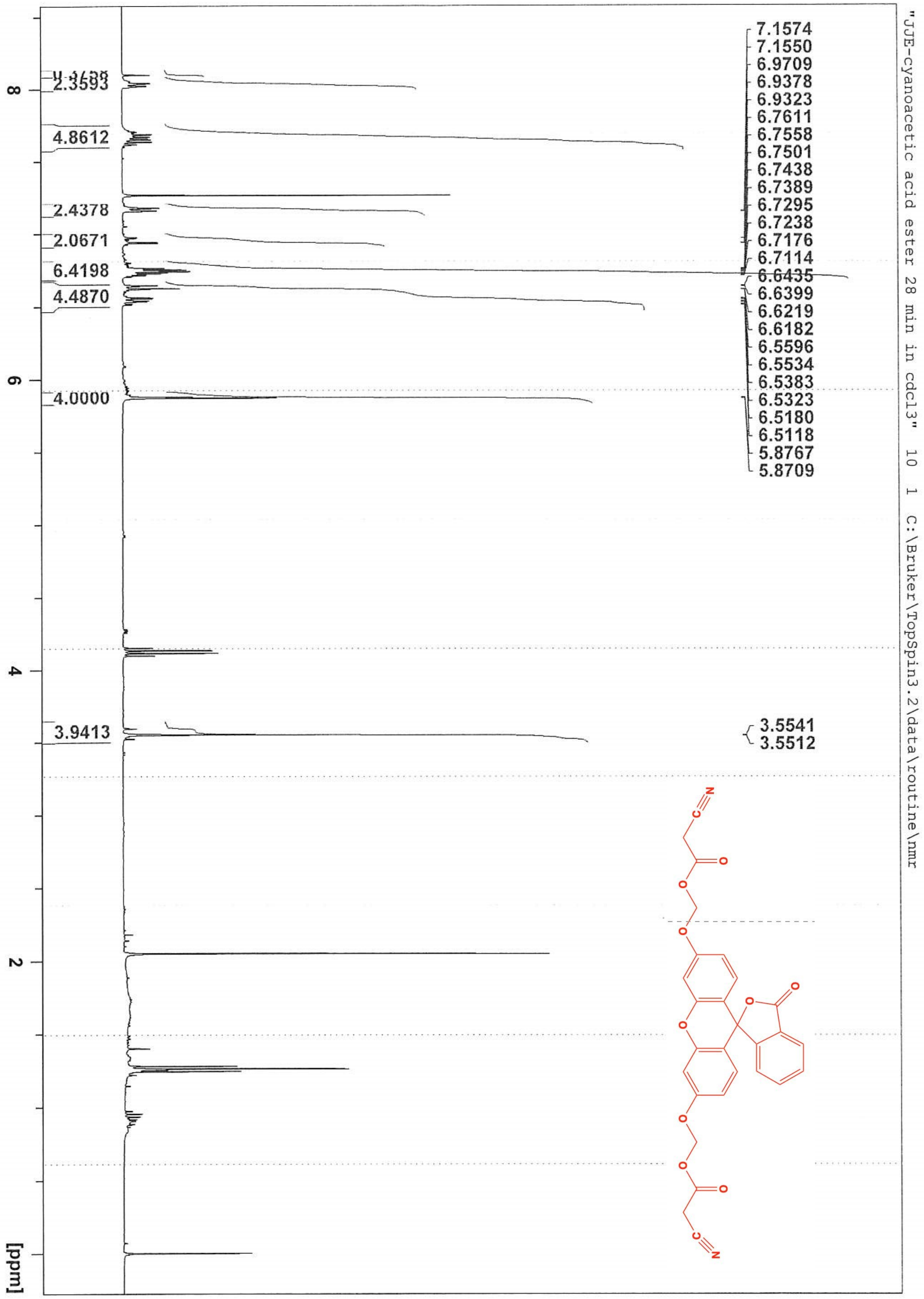
"JJE-cyanoacetic acid ester 28 min in cdcl3" 10 1 C:\Bruker\TopSpin3.2\data\routine\nmr
7.1574
7.1550
6.9709
6.9378
6.9323
6.7611
6.7558
6.7501
6.7438
6.7389
6.7295
6.7238
6.7176
6.7114
6.6435
6.6399
6.6219
6.6182
6.5596
6.5534
6.5383
6.5323
6.5180
6.5118
5.8767
5.8709
3.5541
3.5512
2.3593
4.8612
2.4378
2.0671
6.4198
4.4870
4.0000
3.9413
8
6
4
2
[ppm]

Figure S16

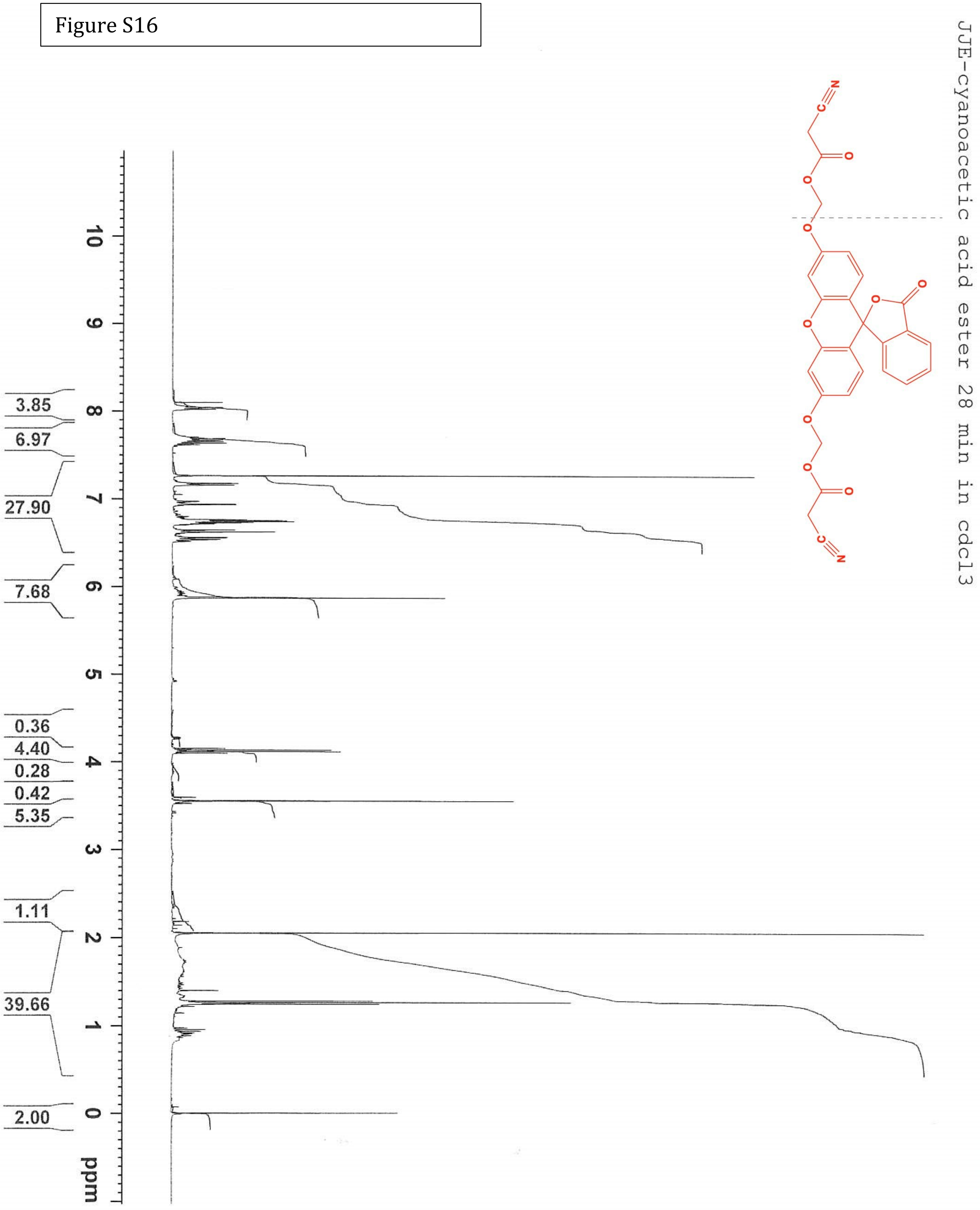
JJE-cyanoacetic acid ester 28 min in cdcl3
3.85
6.97
27.90
7.68
0.36
4.40
0.28
0.42
5.35
1.11
39.66
2.00
10
9
8
7
6
5
4
3
2
1
0
ppm

Figure S17

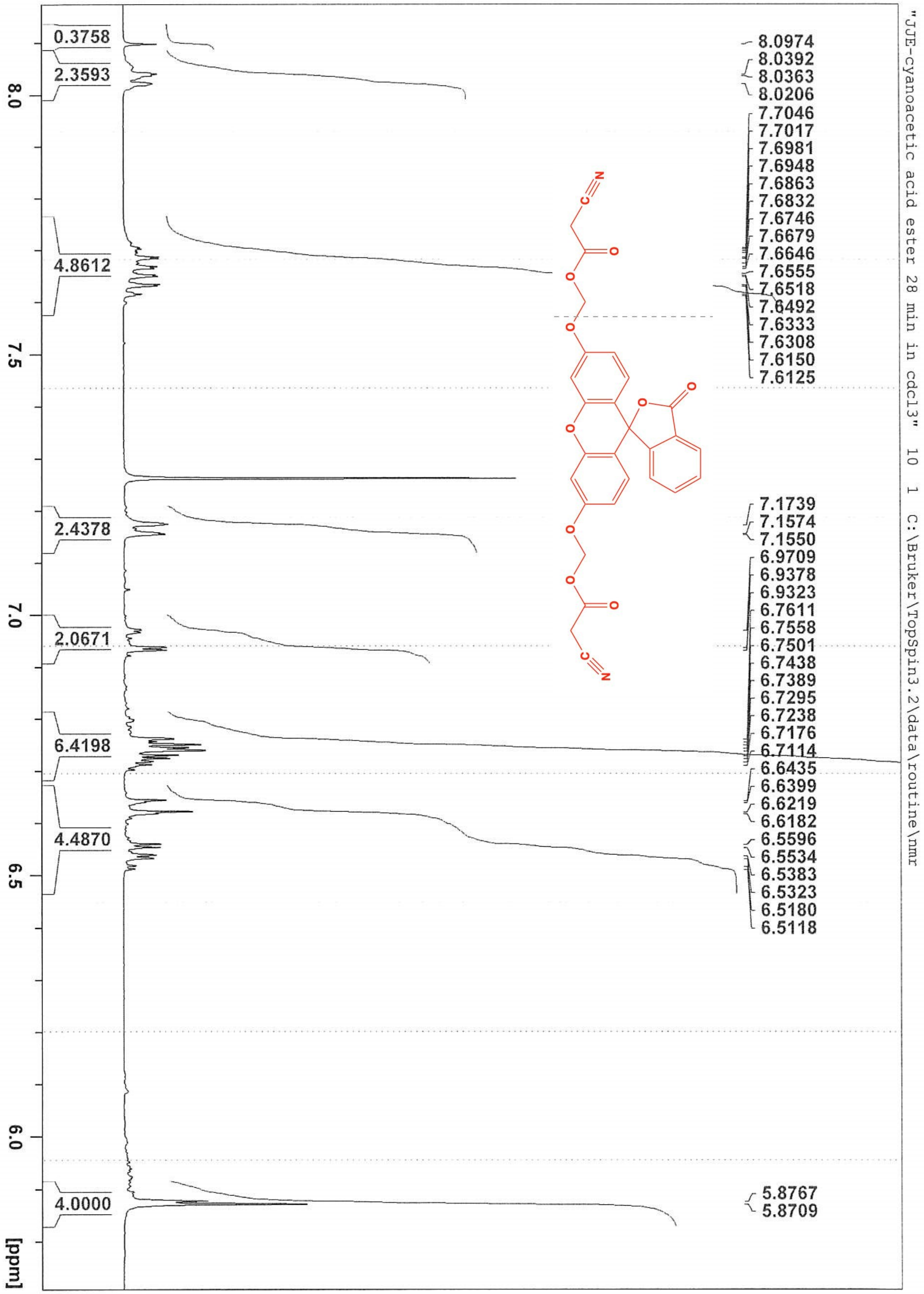
"JJE-cyanoacetic acid ester 28 min in cdcl3" 10 1 C:\Bruker\TopSpin3.2\data\routine\nmr
8.0974
8.0392
8.0363
8.0206
7.7046
7.7017
7.6981
7.6948
7.6863
7.6832
7.6746
7.6679
7.6646
7.6555
7.6518
7.6492
7.6333
7.6308
7.6150
7.6125
7.1739
7.1574
7.1550
6.9709
6.9378
6.9323
6.7611
6.7558
6.7501
6.7438
6.7389
6.7295
6.7238
6.7176
6.7114
6.6435
6.6399
6.6219
6.6182
6.5596
6.5534
6.5383
6.5323
6.5180
6.5118
5.8767
5.8709
0.3758
2.3593
4.8612
2.4378
2.0671
6.4198
4.4870
4.0000
8.0
7.5
7.0
6.5
6.0
[ppm]

Figure S18

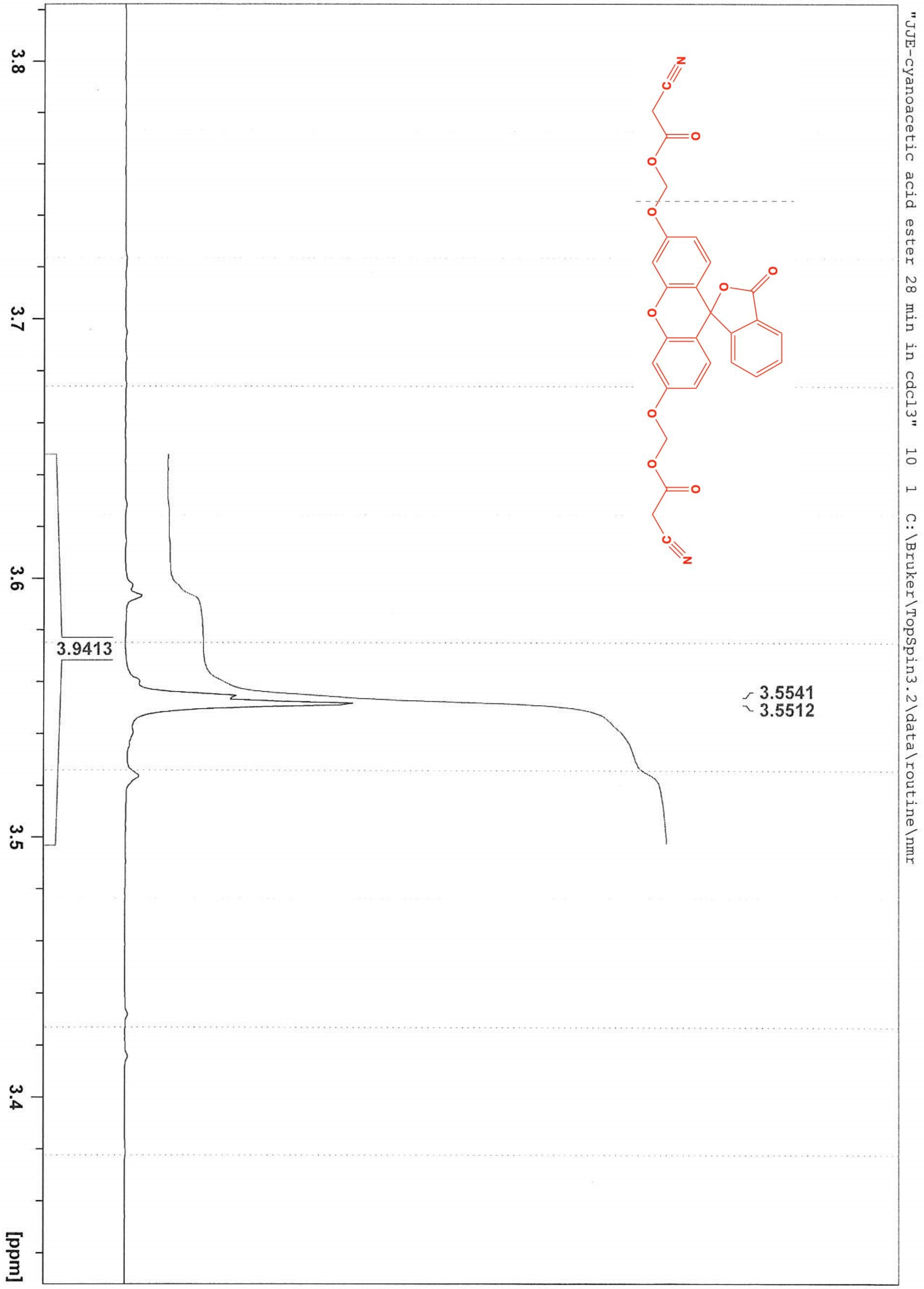
"JJE-cyanoacetic acid ester 28 min in cdcl3" 10 1 C:\Bruker\TopSpin3.2\data\routine\nmr
3.5541
3.5512
3.9413
3.8
3.7
3.6
3.5
3.4
[ppm]

Figure S19

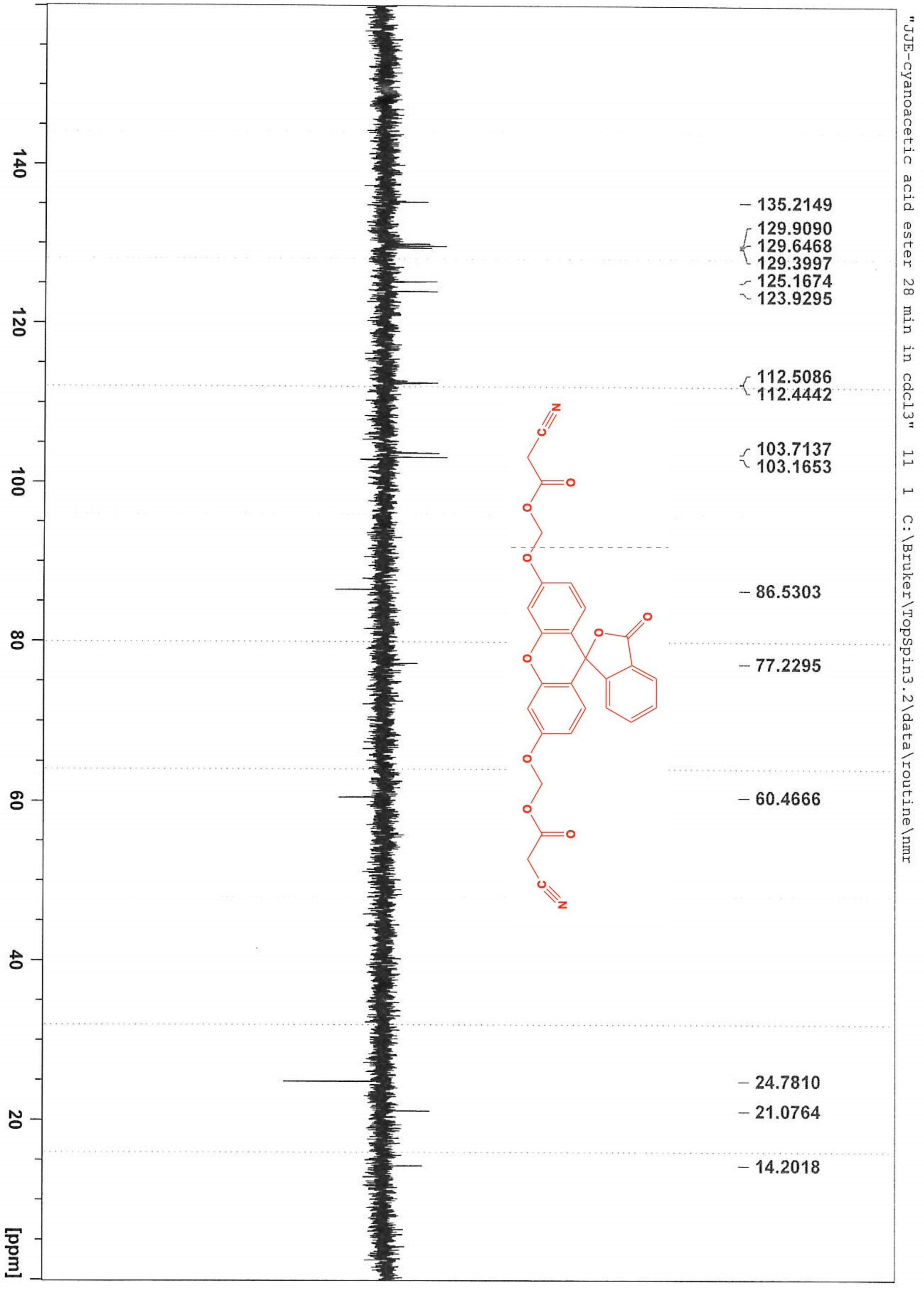

"JJE-cyanoacetic acid ester 28 min in cdcl3" 11 1 C:\Bruker\TopSpin3.2\data\routine\nmr
135.2149
129.9090
129.6468
129.3997
125.1674
123.9295
112.5086
112.4442
103.7137
103.1653
86.5303
77.2295
60.4666
24.7810
21.0764
14.2018
140
120
100
80
60
40
20
[ppm]

Figure S20

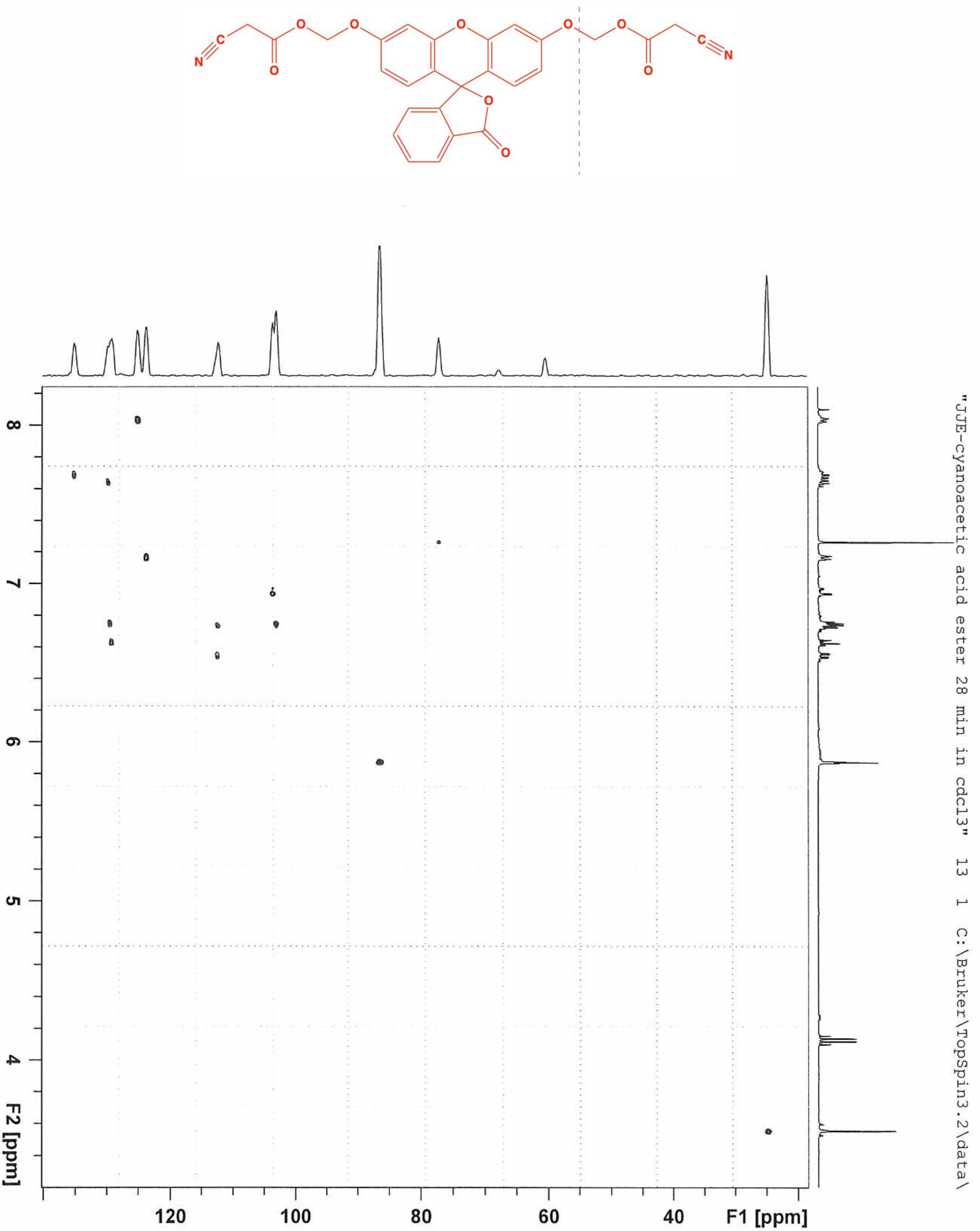
N
C
O
O
O
O
O
O
O
O
O
O
C
N
8
7
6
5
4
F2 [ppm]
120
100
80
60
40
F1 [ppm]
"JJE-cyanoacetic acid ester 28 min in cdcl3" 13 1 C:\Bruker\TopSpin3.2\data\

Figure S21

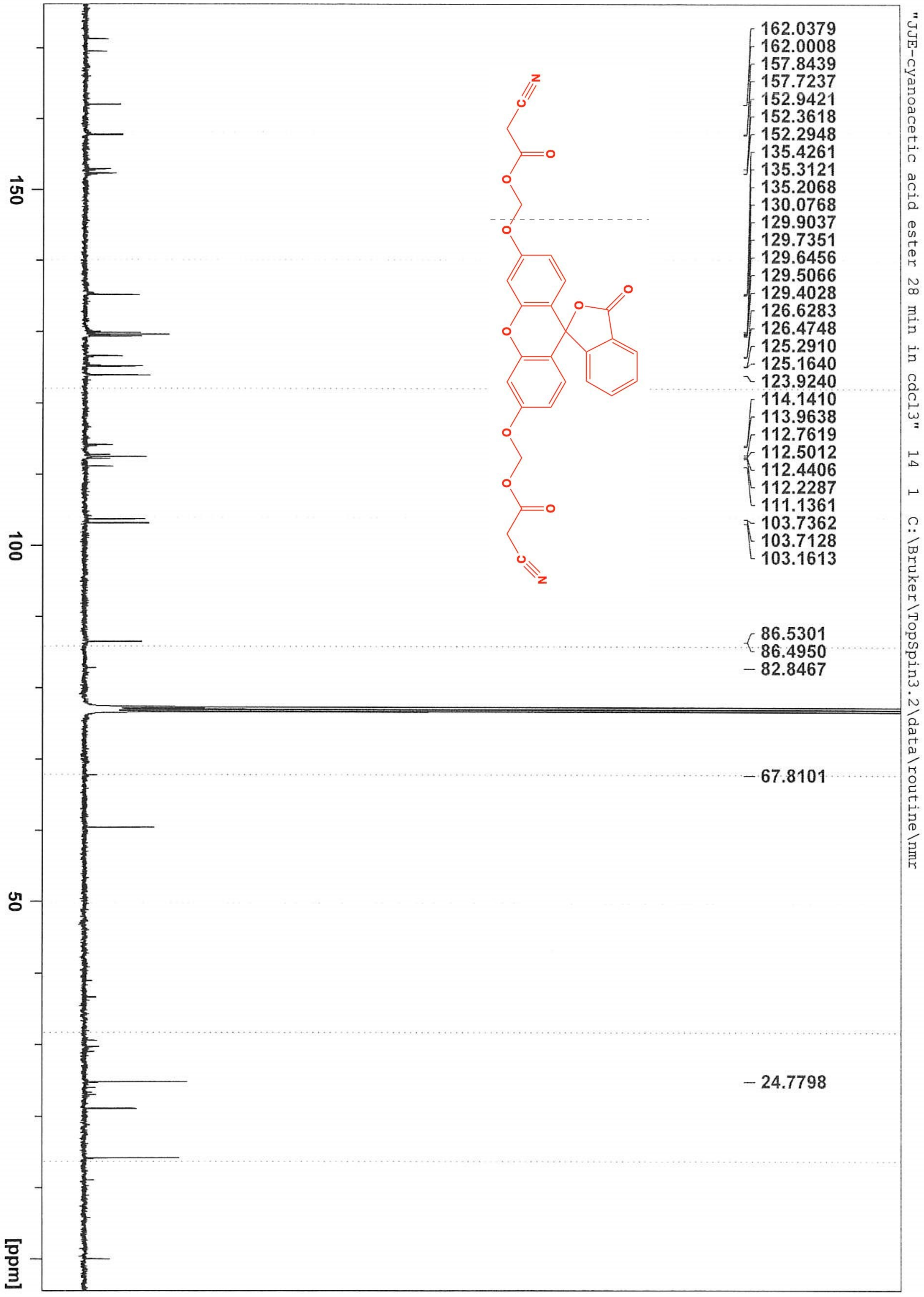
"JJE-cyanoacetic acid ester 28 min in cdcl3" 14 1 C:\Bruker\TopSpin3.2\data\routine\nmr
162.0379
162.0008
157.8439
157.7237
152.9421
152.3618
152.2948
135.4261
135.3121
135.2068
130.0768
129.9037
129.7351
129.6456
129.5066
129.4028
126.6283
126.4748
125.2910
125.1640
123.9240
114.1410
113.9638
112.7619
112.5012
112.4406
112.2287
111.1361
103.7362
103.7128
103.1613
86.5301
86.4950
82.8467
67.8101
24.7798
150
100
50
[ppm]

C:\Xcalibur\data\2022\August\BU-4 8/29/2022 9:33:47 AM

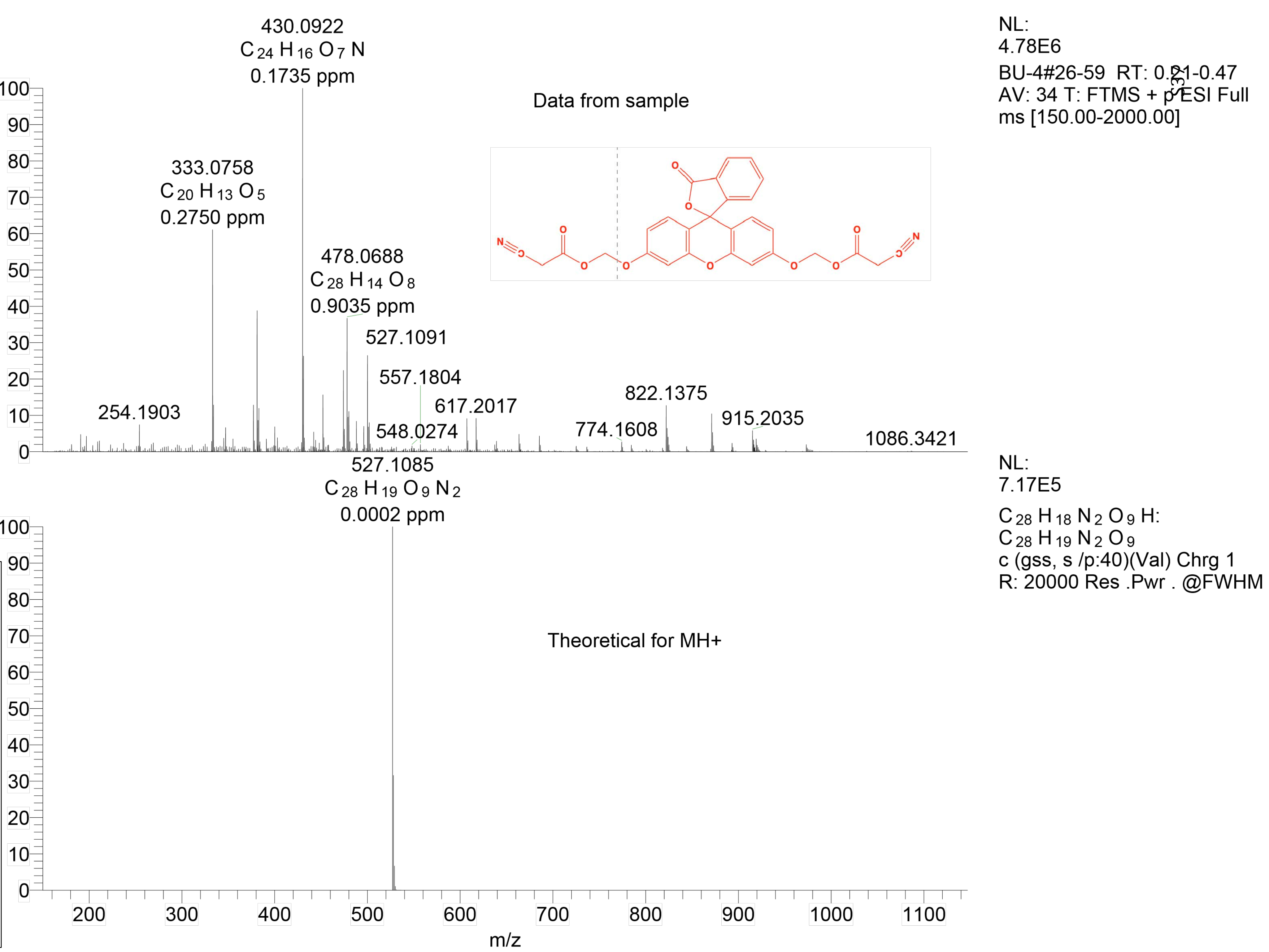

Figure S22

Figure S23

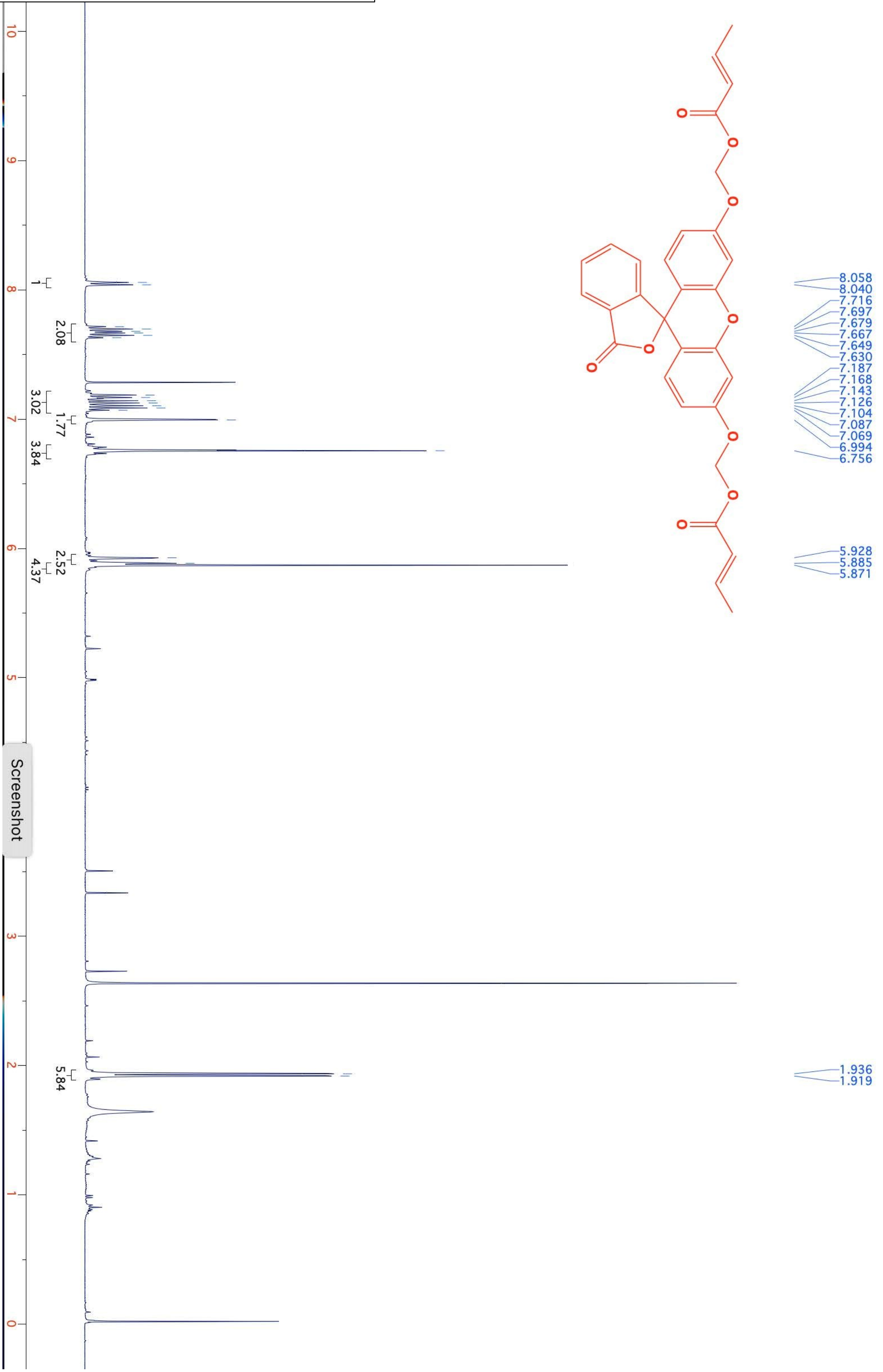
8.058
8.040
7.716
7.697
7.679
7.667
7.649
7.630
7.187
7.168
7.143
7.126
7.104
7.087
7.069
6.994
6.756
5.928
5.885
5.871
1.936
1.919

Figure S24

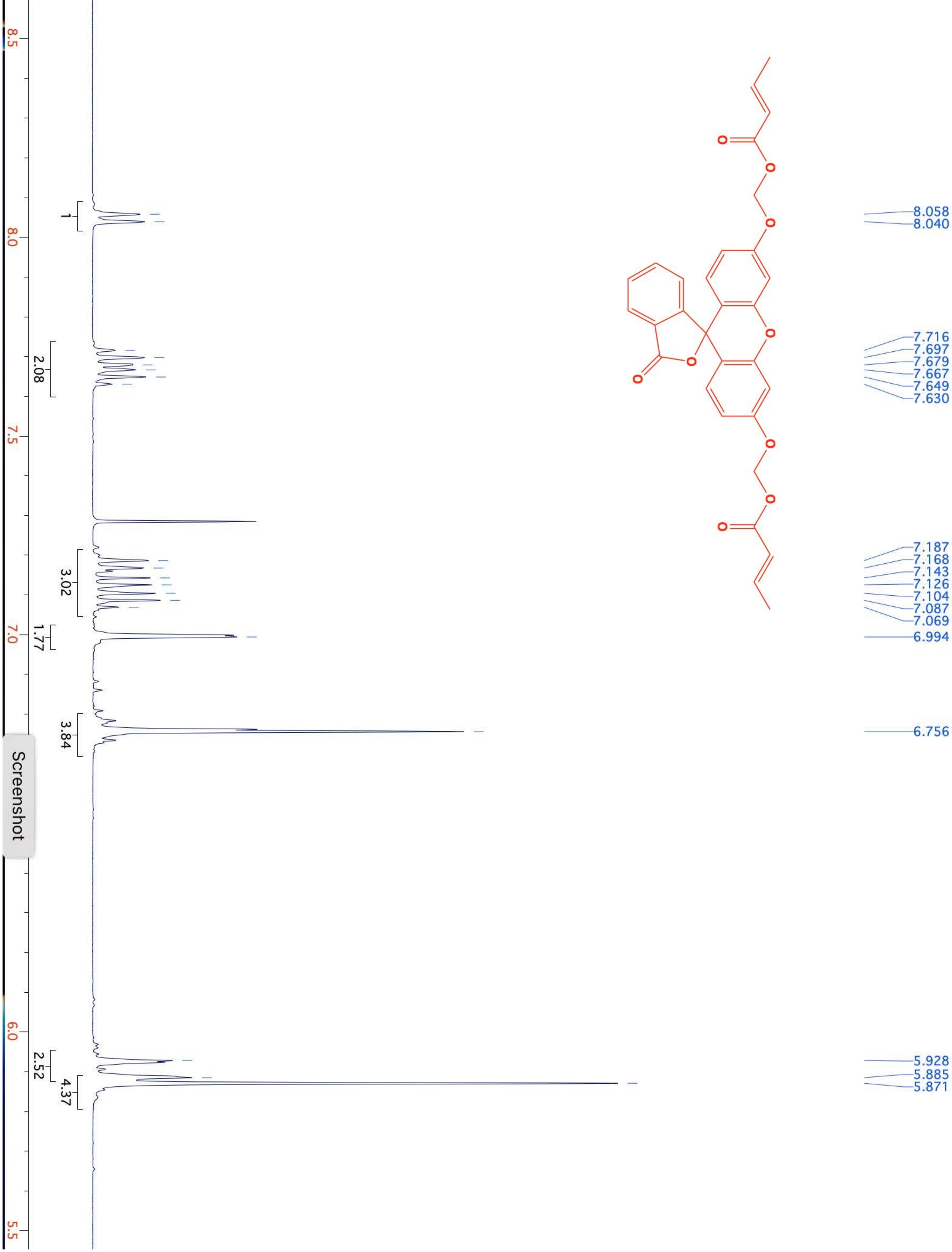

8.058
8.040
7.716
7.697
7.679
7.667
7.649
7.630
7.187
7.168
7.143
7.126
7.104
7.087
7.069
6.994
6.756
5.928
5.885
5.871
1
2.08
3.02
1.77
3.84
2.52
4.37
8.5
8.0
7.5
7.0
6.0
5.5

Figure S25

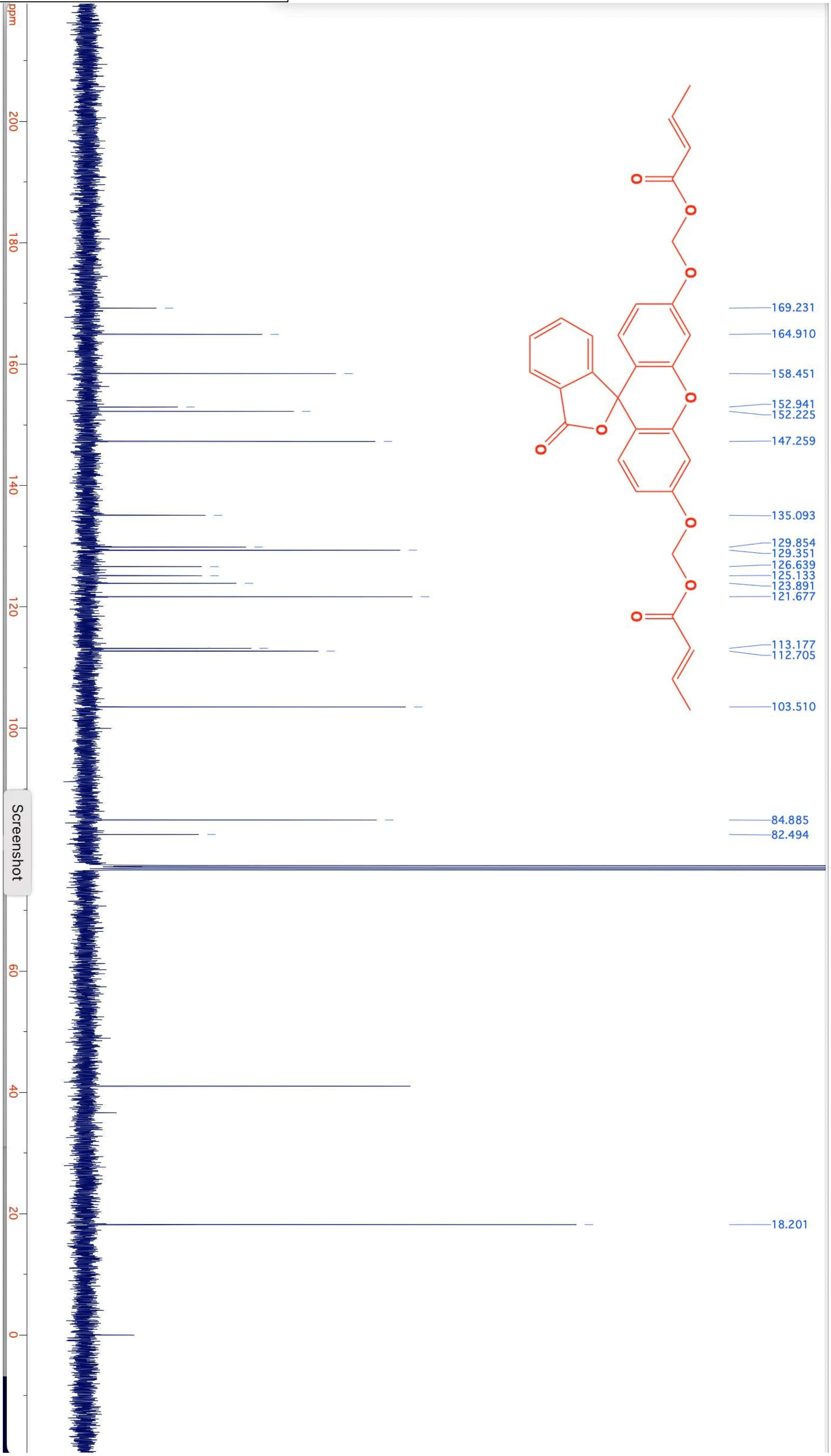
ppm
200
180
160
140
120
100
60
40
20
0
Screenshot
169.231
164.910
158.451
152.941
152.225
147.259
135.093
129.854
129.351
126.639
125.133
123.891
121.677
113.177
112.705
103.510
84.885
82.494
18.201

Figure S26

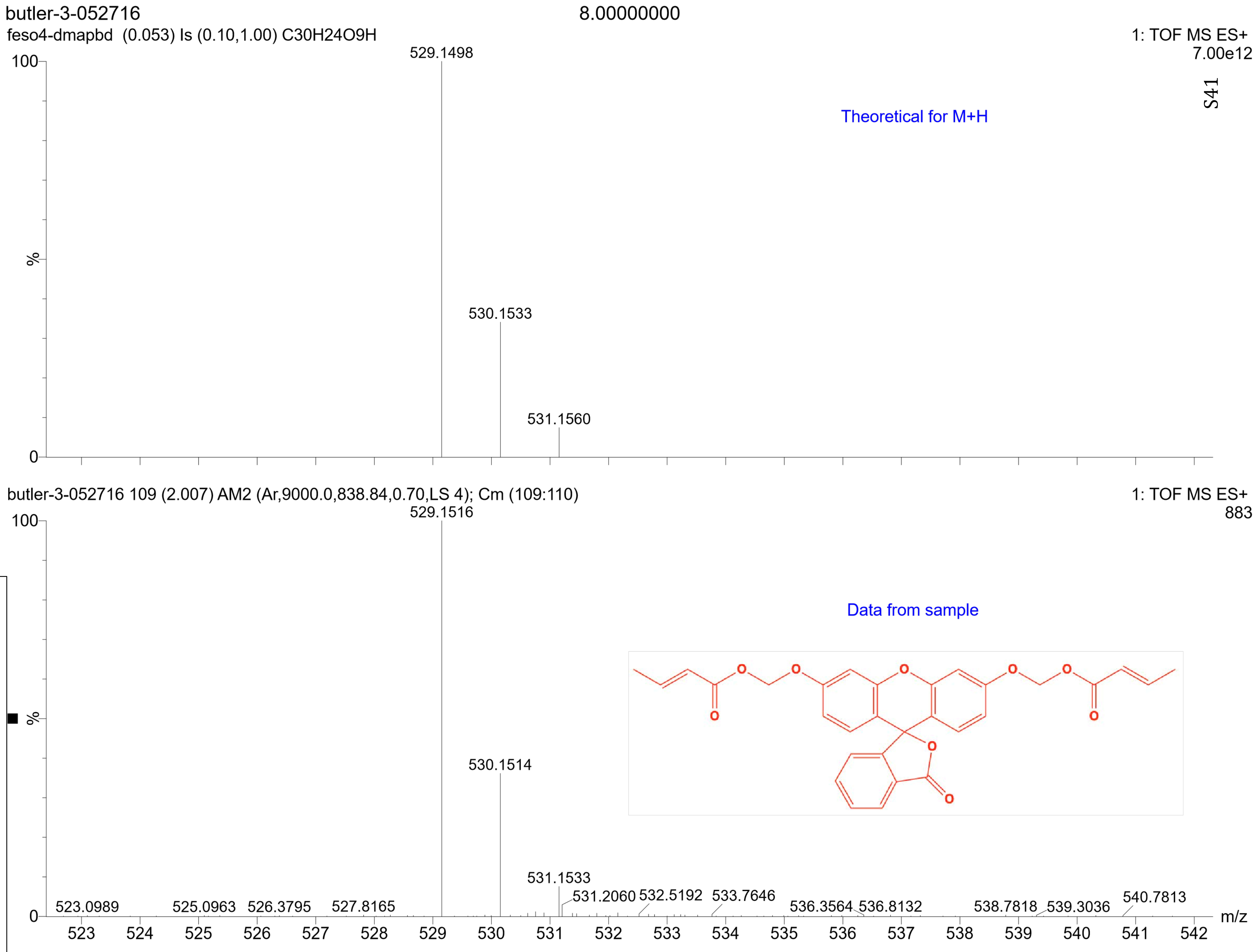
butler-3-052716
8.00000000
feso4-dmapbd (0.053) Is (0.10,1.00) C30H24O9H
1: TOF MS ES+
7.00e12
Theoretical for M+H
529.1498
530.1533
531.1560
butler-3-052716 109 (2.007) AM2 (Ar,9000.0,838.84,0.70,LS 4); Cm (109:110)
1: TOF MS ES+
883
Data from sample
529.1516
530.1514
531.1533
531.2060
532.5192
533.7646
536.3564
536.8132
538.7818
539.3036
540.7813
523.0989
525.0963
526.3795
527.8165
100
%
0
m/z

Figure S27

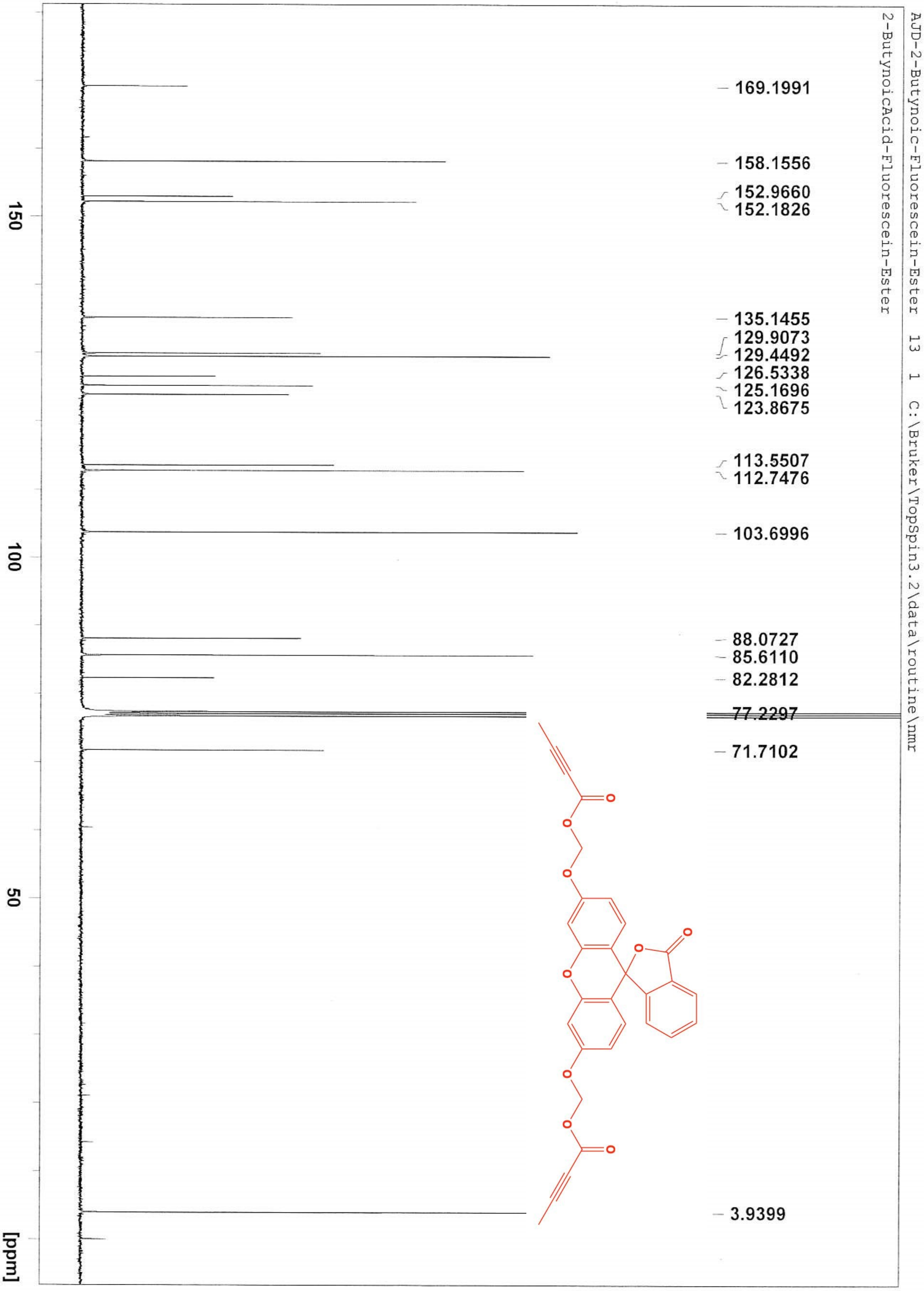

AJD-2-Butynoic-Fluorescein-Ester 13 1 C:\Bruker\TopSpin3.2\data\routine\nmr
2-ButynoicAcid-Fluorescein-Ester
169.1991
158.1556
152.9660
152.1826
135.1455
129.9073
129.4492
126.5338
125.1696
123.8675
113.5507
112.7476
103.6996
88.0727
85.6110
82.2812
77.2297
71.7102
3.9399
150
100
50
[ppm]

Figure S28

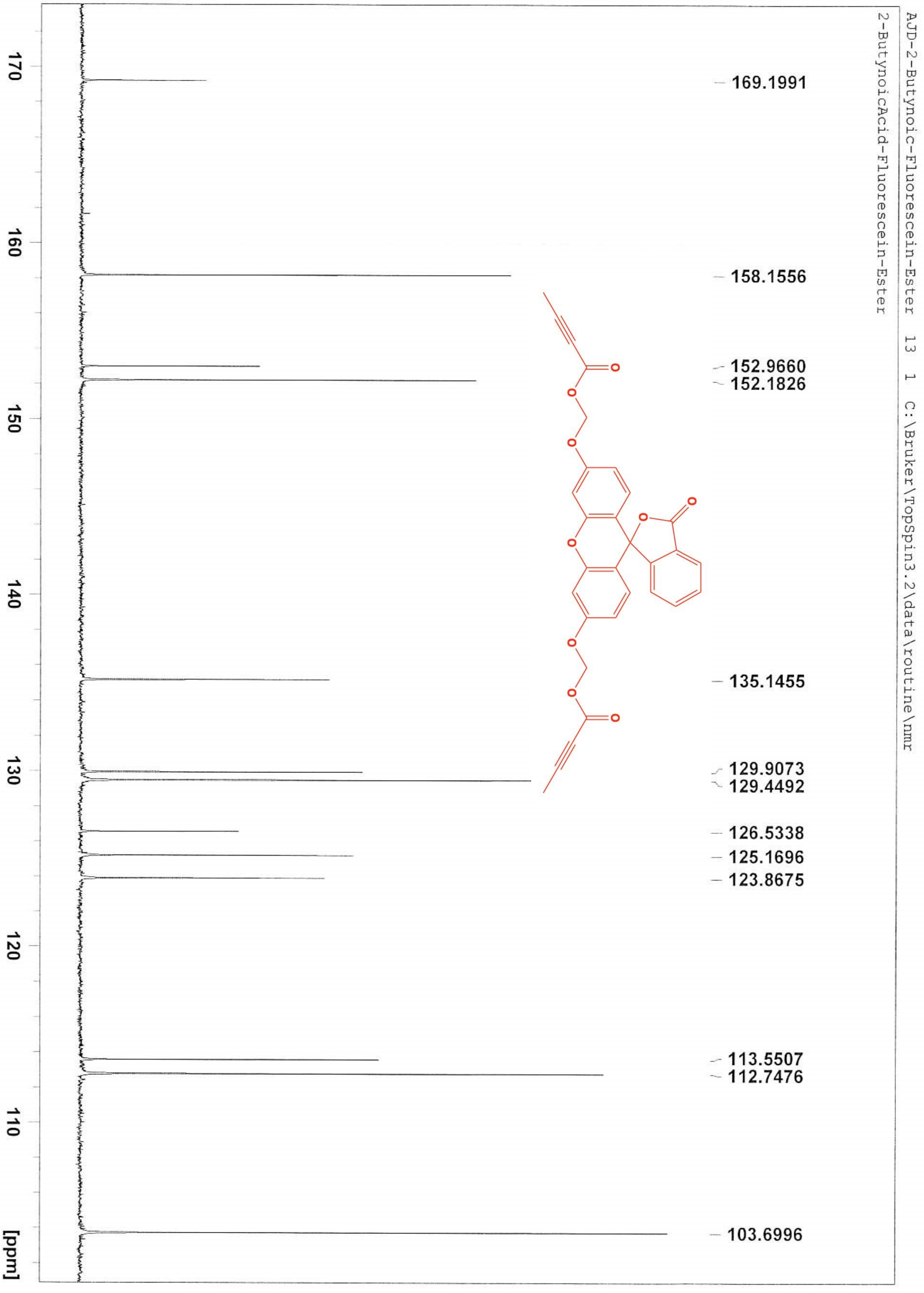
AJD-2-Butynoic-Fluorescein-Ester 13 1 C:\Bruker\TopSpin3.2\data\routine\nmr
2-ButynoicAcid-Fluorescein-Ester
169.1991
158.1556
152.9660
152.1826
135.1455
129.9073
129.4492
126.5338
125.1696
123.8675
113.5507
112.7476
103.6996
170
160
150
140
130
120
110
[ppm]

Figure S29

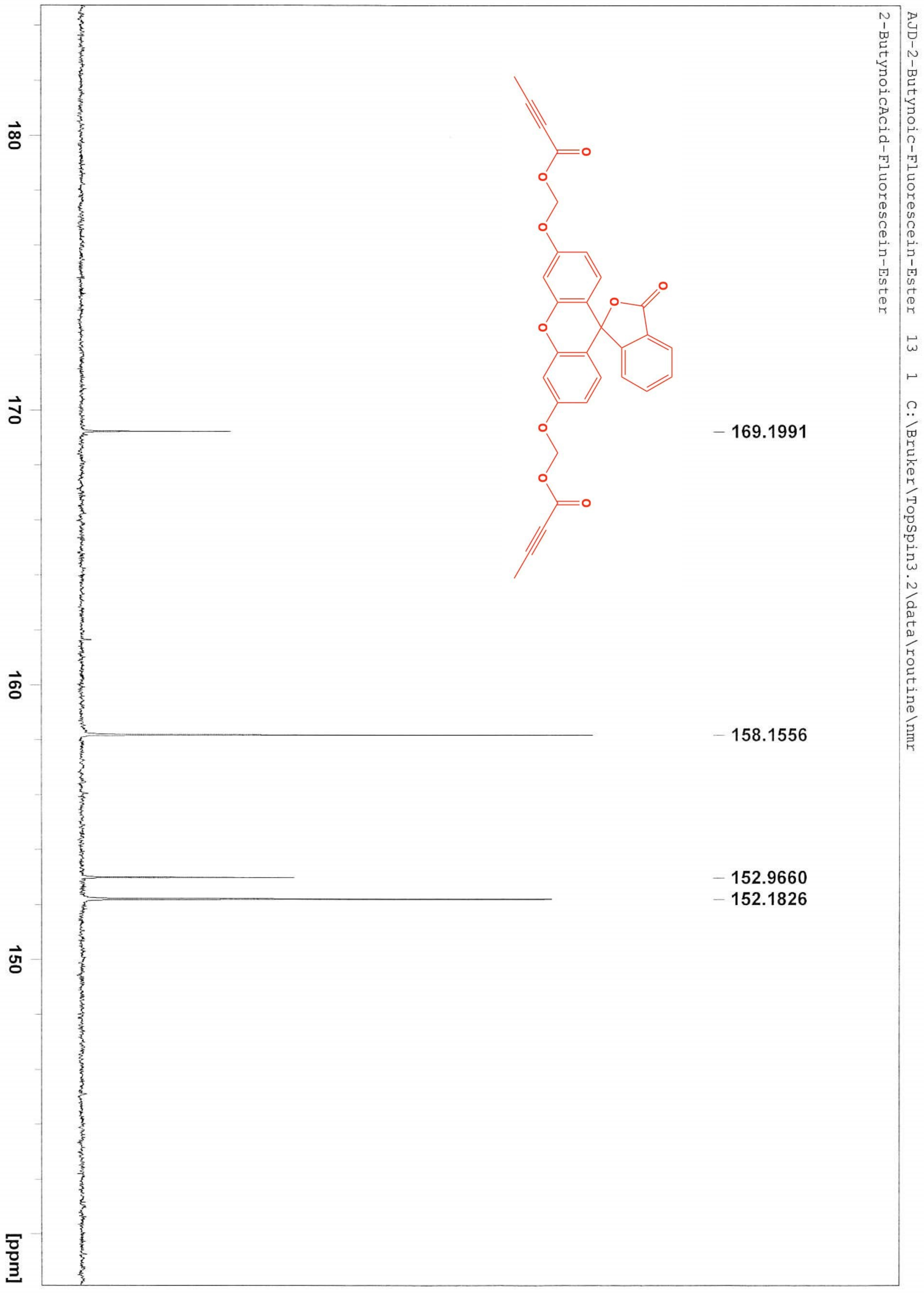
AJD-2-Butynoic-Fluorescein-Ester 13 1 C:\Bruker\TopSpin3.2\data\routine\nmr
2-ButynoicAcid-Fluorescein-Ester
169.1991
158.1556
152.9660
152.1826
180
170
160
150
[ppm]

Figure S30

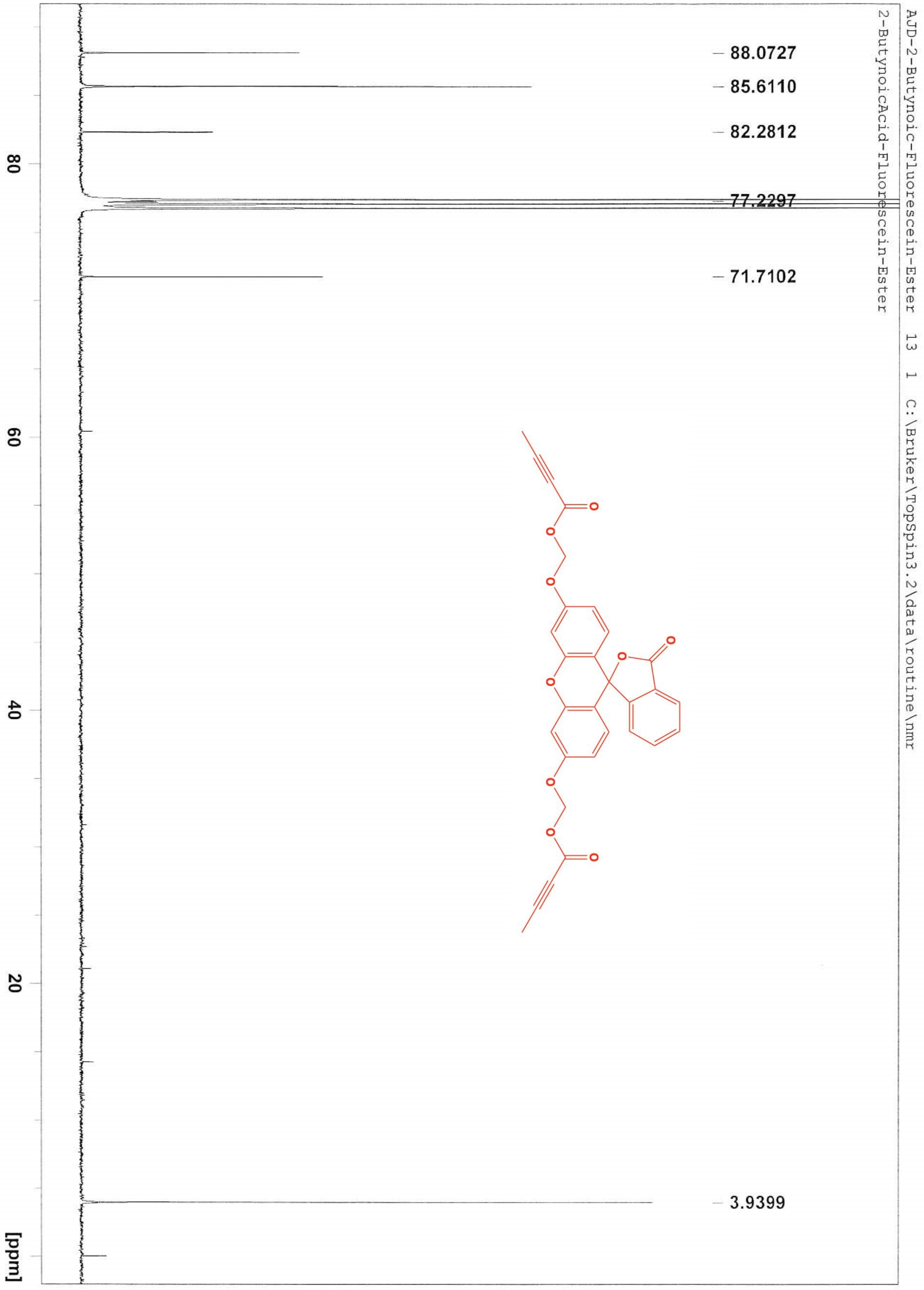
AJD-2-Butynoic-Fluorescein-Ester 13 1 C:\Bruker\TopSpin3.2\data\routine\nmr
2-ButynoicAcid-Fluorescein-Ester
88.0727
85.6110
82.2812
77.2297
71.7102
3.9399
80
60
40
20
[ppm]

Figure S31

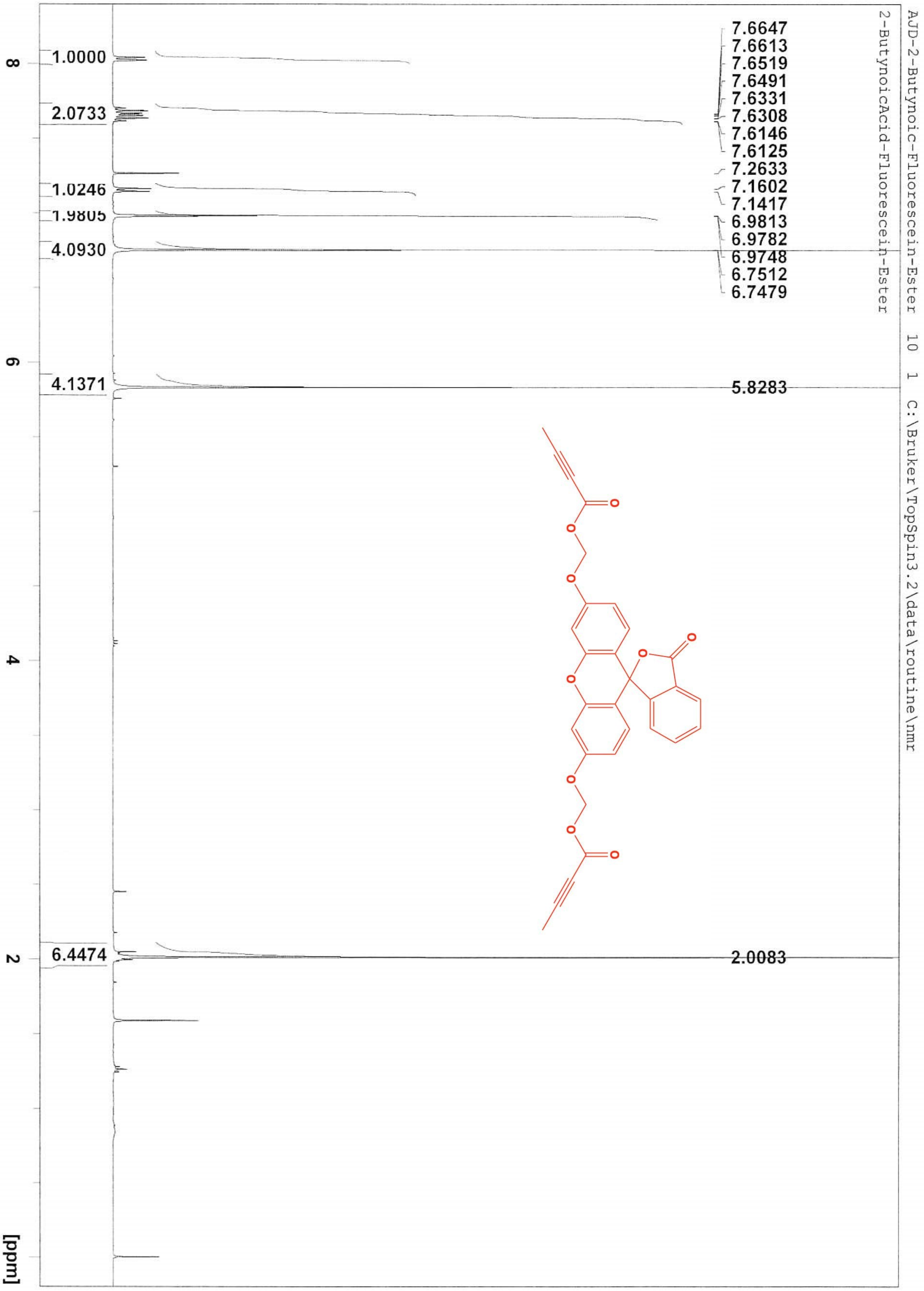

AJD-2-Butynoic-Fluorescein-Ester
2-ButynoicAcid-Fluorescein-Ester
10 1 C:\Bruker\TopSpin3.2\data\routine\nmr
7.6647
7.6613
7.6519
7.6491
7.6331
7.6308
7.6146
7.6125
7.2633
7.1602
7.1417
6.9813
6.9782
6.9748
6.7512
6.7479
5.8283
2.0083
1.0000
2.0733
1.0246
1.9805
4.0930
4.1371
6.4474
8
6
4
2
[ppm]

Figure S32

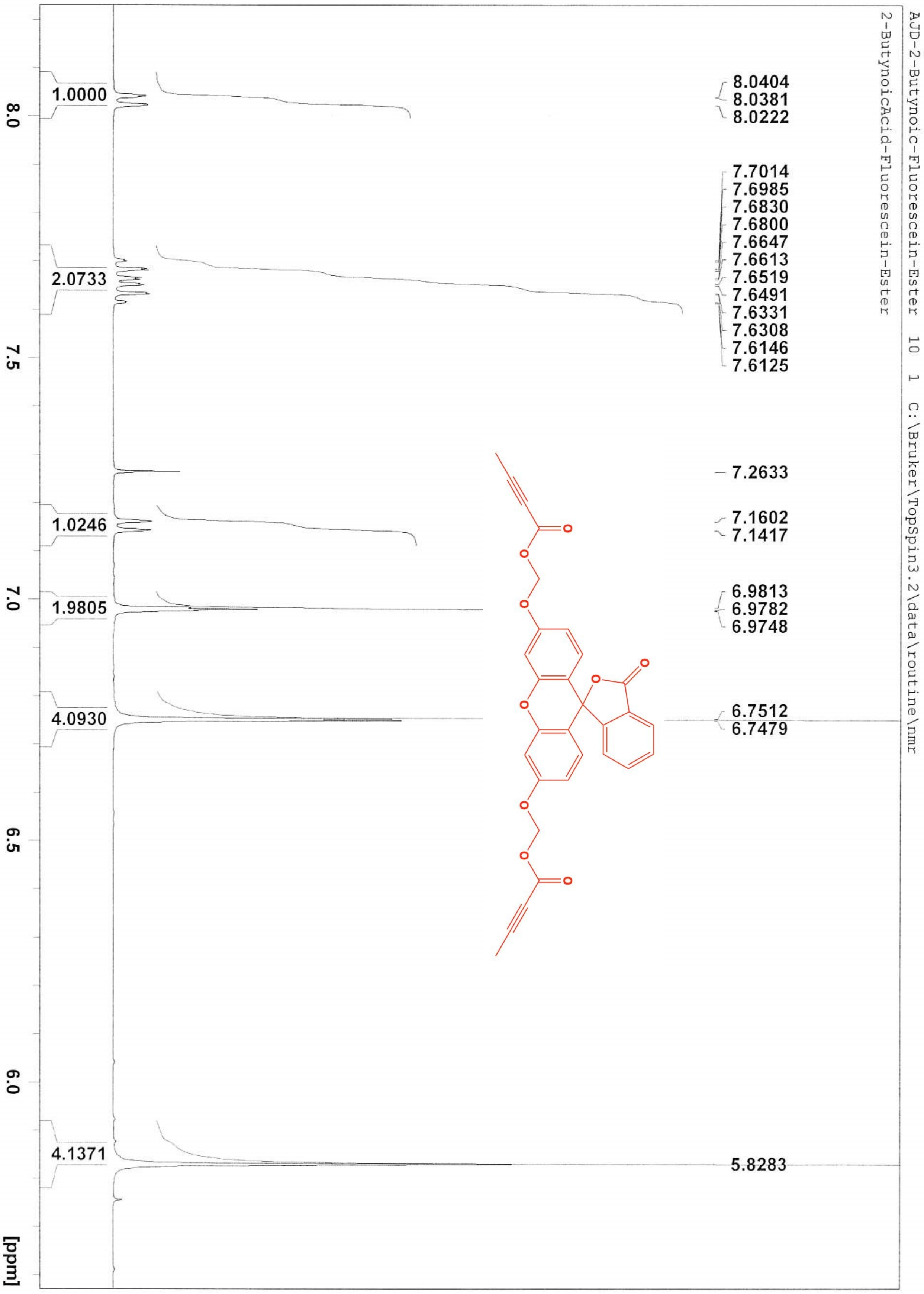
AJD-2-Butynoic-Fluorescein-Ester 10 1 C:\Bruker\TopSpin3.2\data\routine\nmr
2-ButynoicAcid-Fluorescein-Ester
8.0404
8.0381
8.0222
7.7014
7.6985
7.6830
7.6800
7.6647
7.6613
7.6519
7.6491
7.6331
7.6308
7.6146
7.6125
7.2633
7.1602
7.1417
6.9813
6.9782
6.9748
6.7512
6.7479
5.8283
1.0000
2.0733
1.0246
1.9805
4.0930
4.1371
8.0
7.5
7.0
6.5
6.0
[ppm]

Figure S33

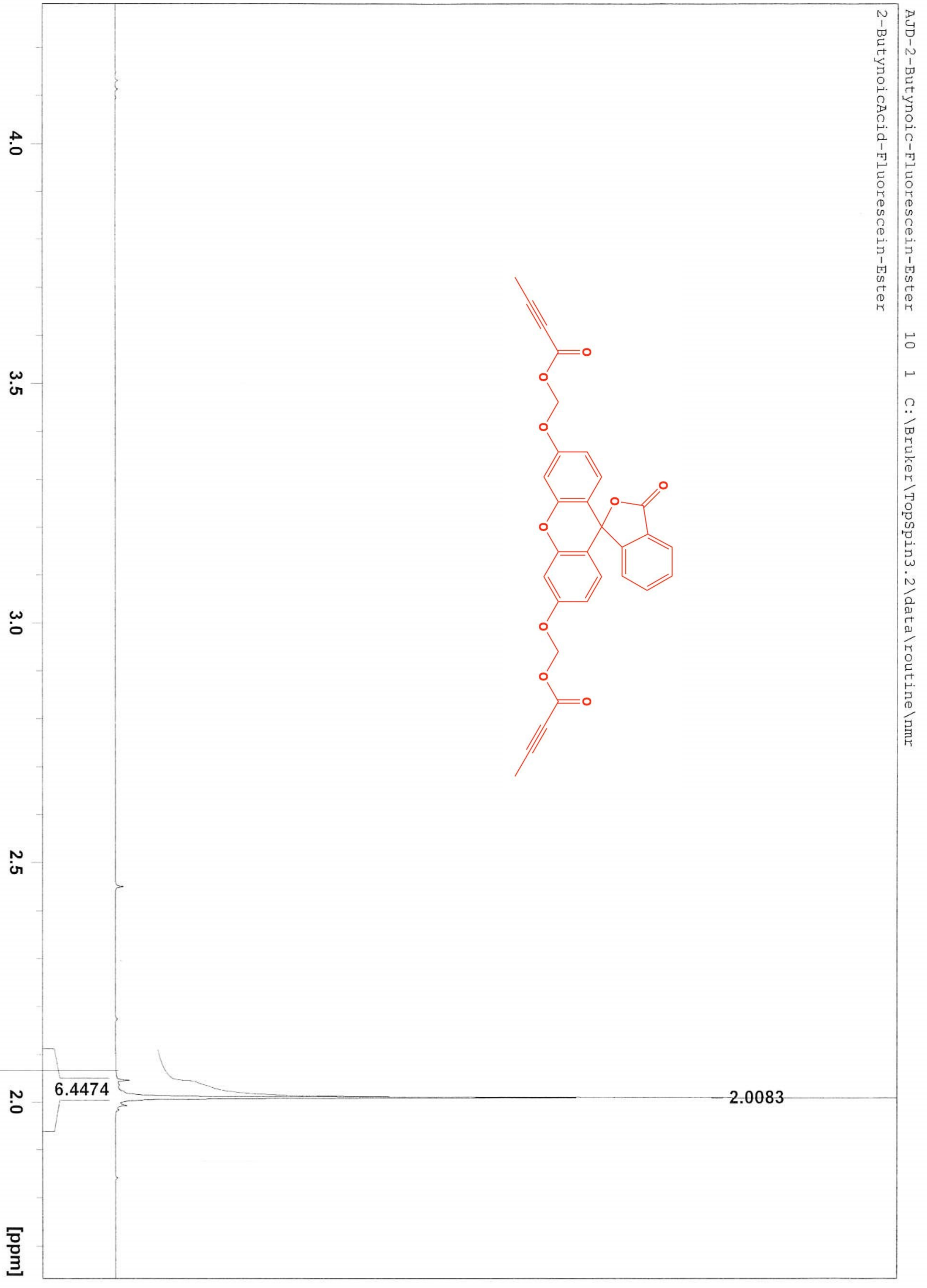
AJD-2-Butynoic-Fluorescein-Ester 10 1 C:\Bruker\TopSpin3.2\data\routine\nmr
2-ButynoicAcid-Fluorescein-Ester
4.0
3.5
3.0
2.5
2.0
[ppm]
6.4474
2.0083

Figure S34

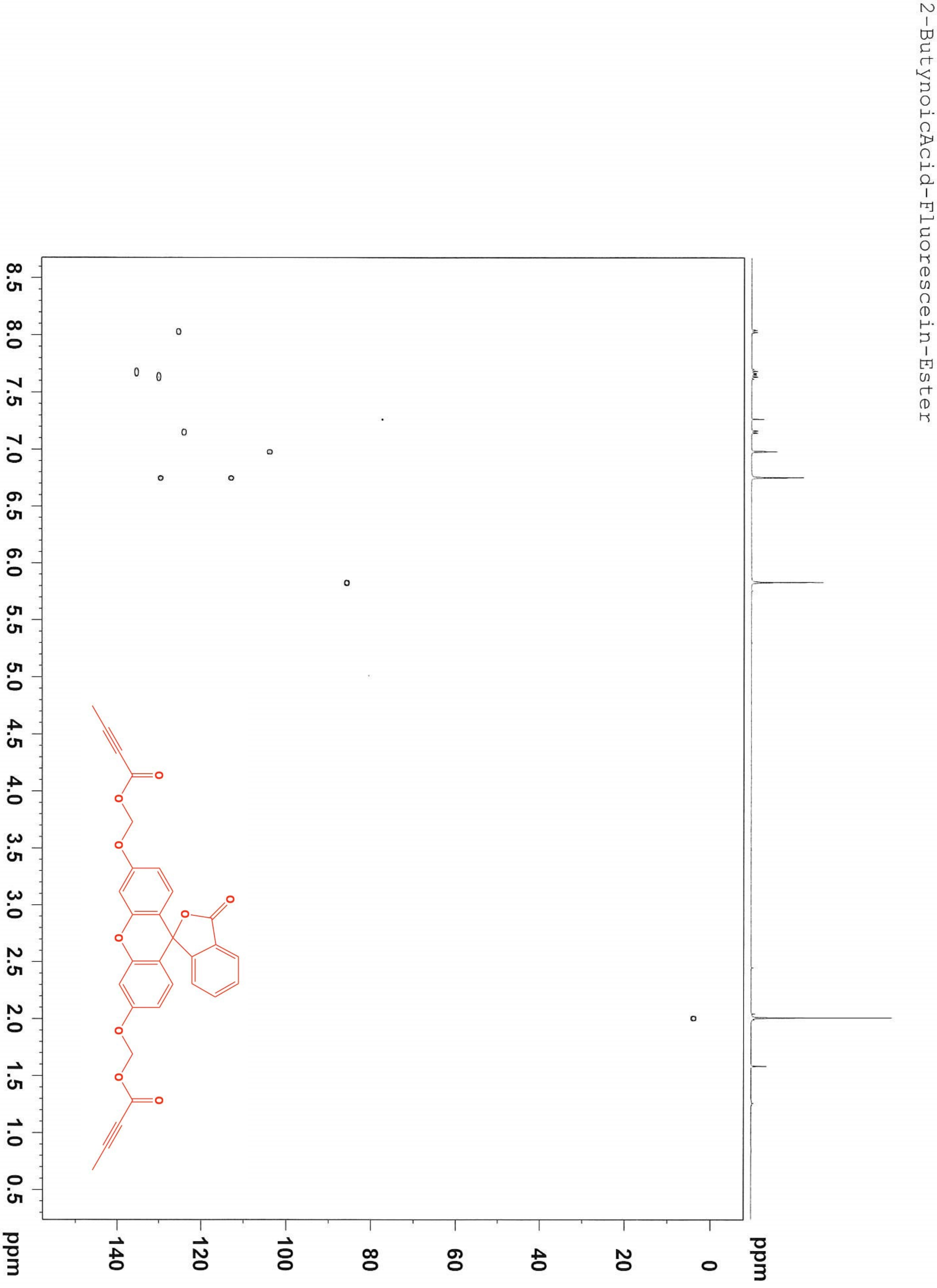
2-ButynoicAcid-Fluorescein-Ester
ppm
8.5
8.0
7.5
7.0
6.5
6.0
5.5
5.0
4.5
4.0
3.5
3.0
2.5
2.0
1.5
1.0
0.5
ppm
140
120
100
80
60
40
20
0

Figure S35

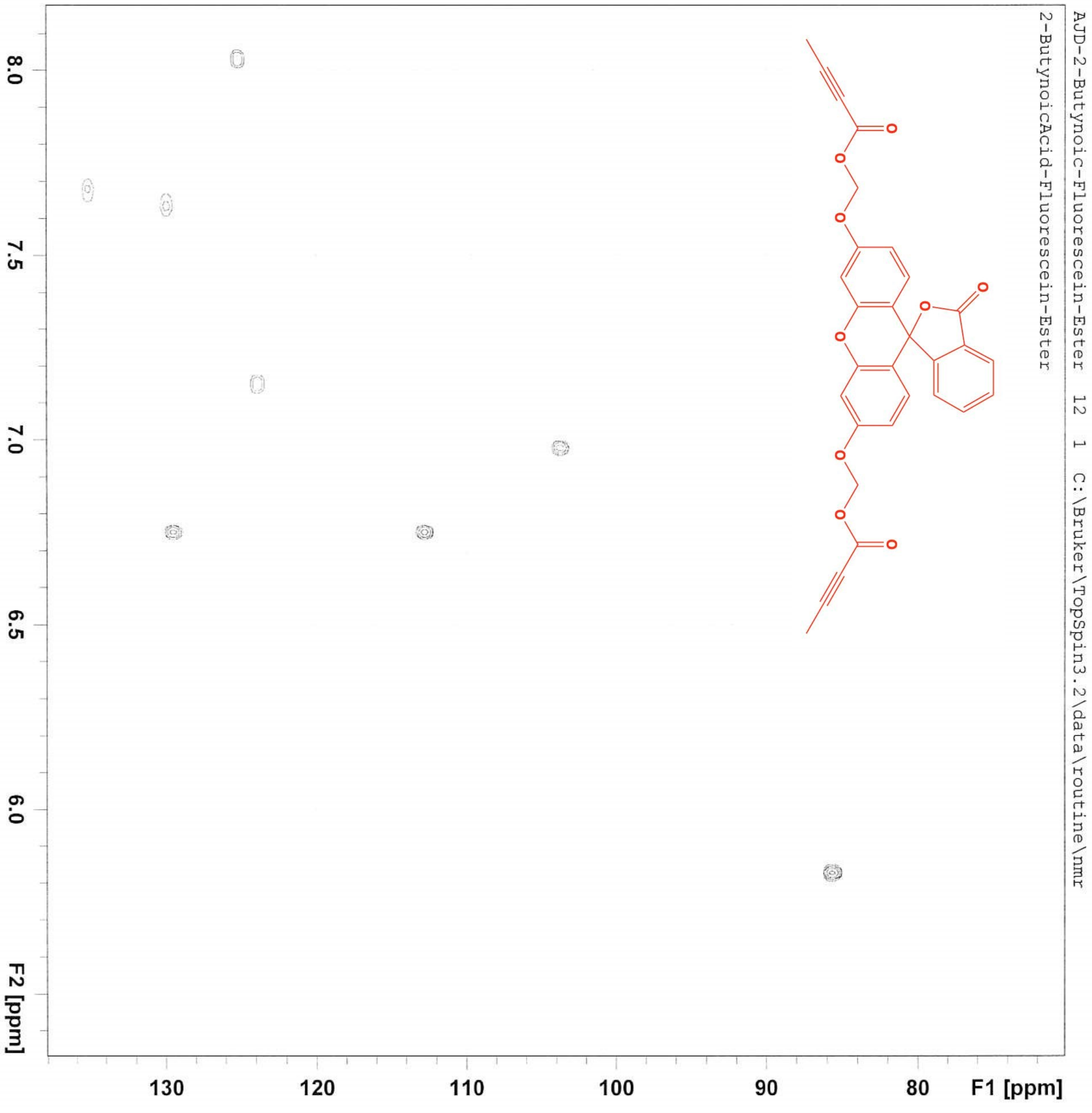

AJD-2-Butynoic-Fluorescein-Ester 12 1 C:\Bruker\TopSpin3.2\data\routine\nmr
2-ButynoicAcid-Fluorescein-Ester
F2 [ppm]
8.0
7.5
7.0
6.5
6.0
130
120
110
100
90
80
F1 [ppm]

Figure S36

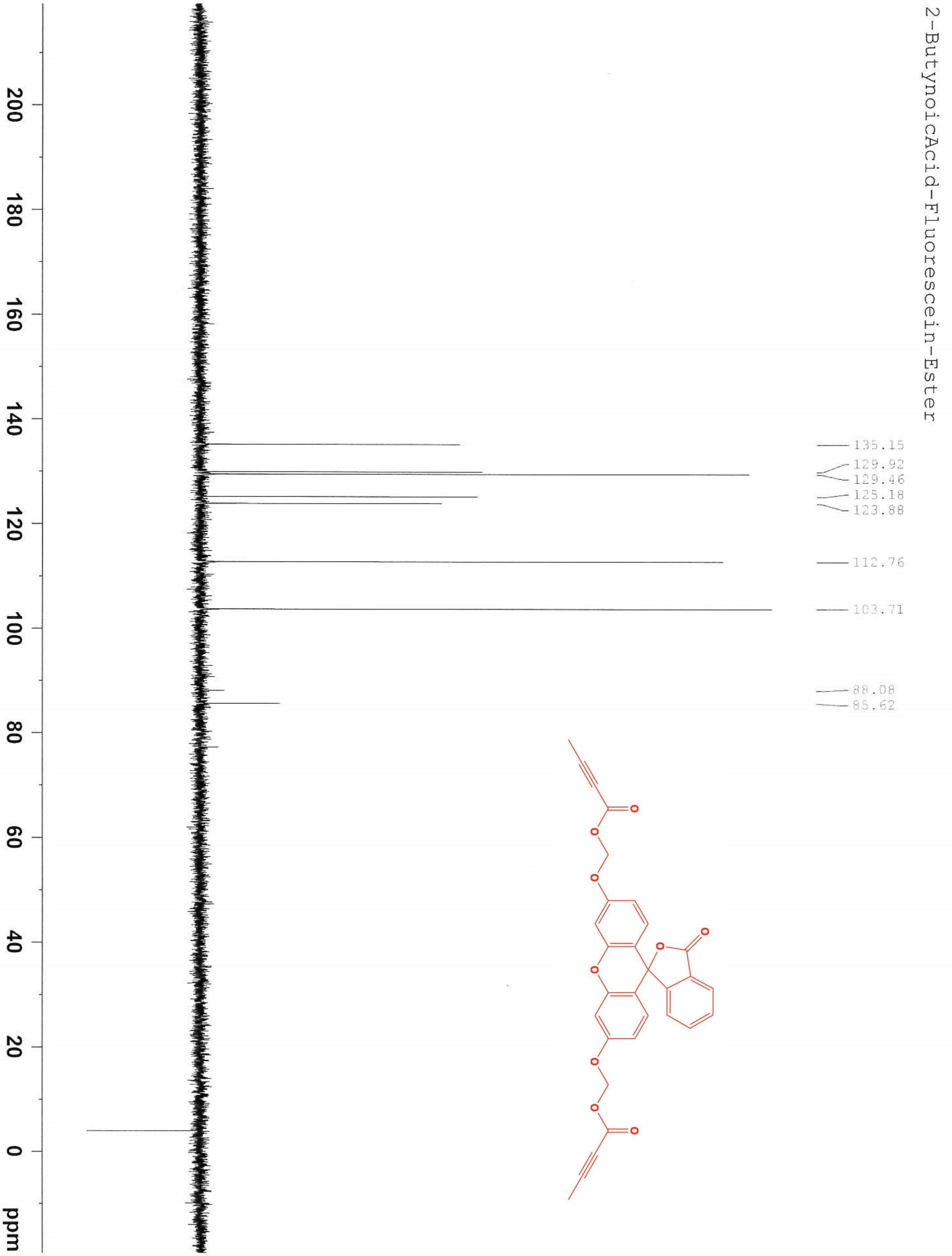

2-ButynoicAcid-Fluorescein-Ester
135.15
129.92
129.46
125.18
123.88
112.76
103.71
88.08
85.62
200
180
160
140
120
100
80
60
40
20
0
ppm

C:\Xcalibur\data\2022\January\JE-1 1/27/2022 1:45:58 PM

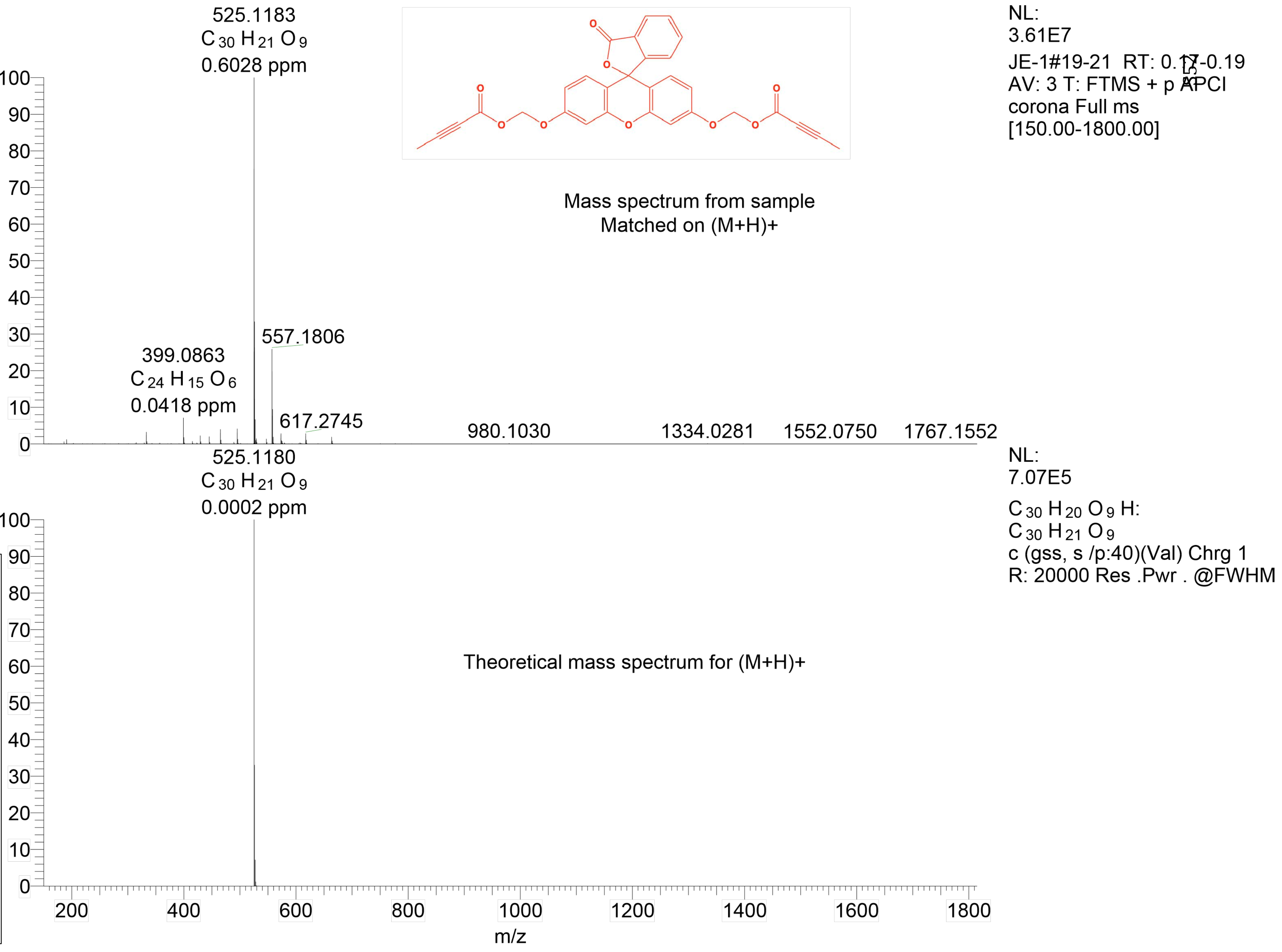

Figure S37

Figure S38

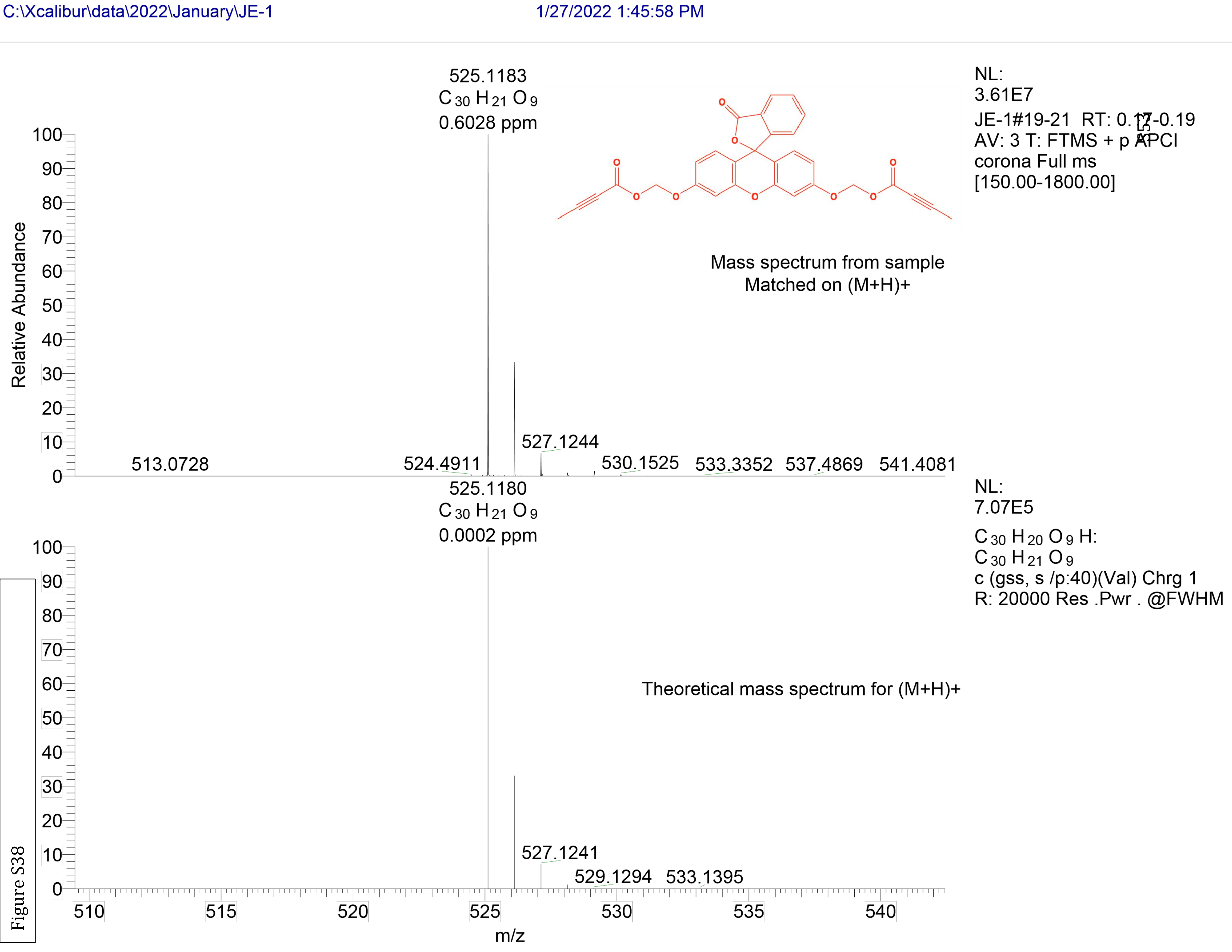
C:\Xcalibur\data\2022\January\JE-1
1/27/2022 1:45:58 PM
NL:
3.61E7
JE-1#19-21 RT: 0.17-0.19
AV: 3 T: FTMS + p APCI
corona Full ms
[150.00-1800.00]
Mass spectrum from sample
Matched on (M+H)+
525.1183
C 30 H 21 O 9
0.6028 ppm
513.0728
524.4911
527.1244
530.1525
533.3352
537.4869
541.4081
NL:
7.07E5
C 30 H 20 O 9 H:
C 30 H 21 O 9
c (gss, s /p:40)(Val) Chrg 1
R: 20000 Res .Pwr . @FWHM
Theoretical mass spectrum for (M+H)+
525.1180
C 30 H 21 O 9
0.0002 ppm
527.1241
529.1294
533.1395
Relative Abundance
m/z

Figure S39

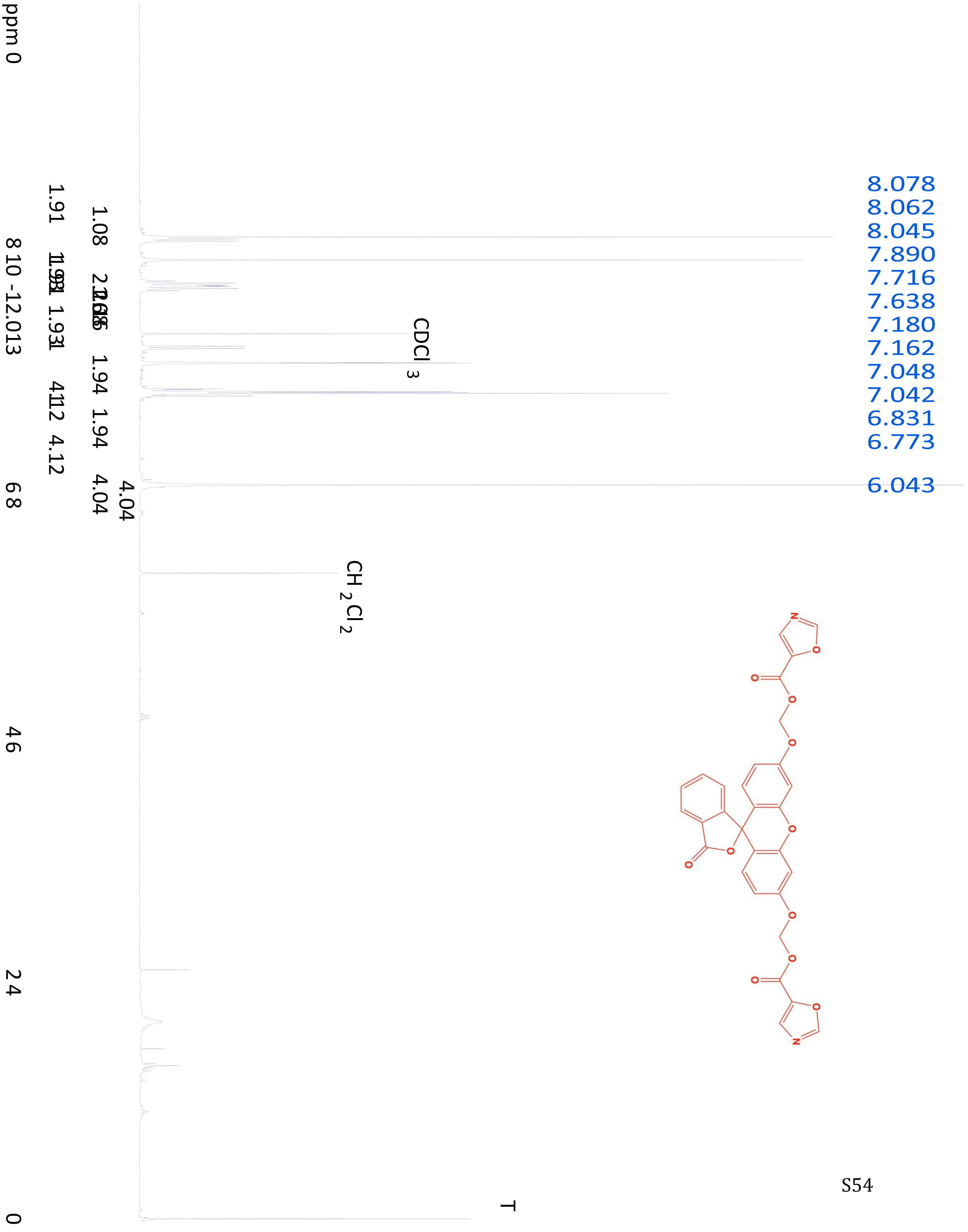
8.078
8.062
8.045
7.890
7.716
7.638
7.180
7.162
7.048
7.042
6.831
6.773
6.043
CDCl3
CH2Cl2

Figure S40

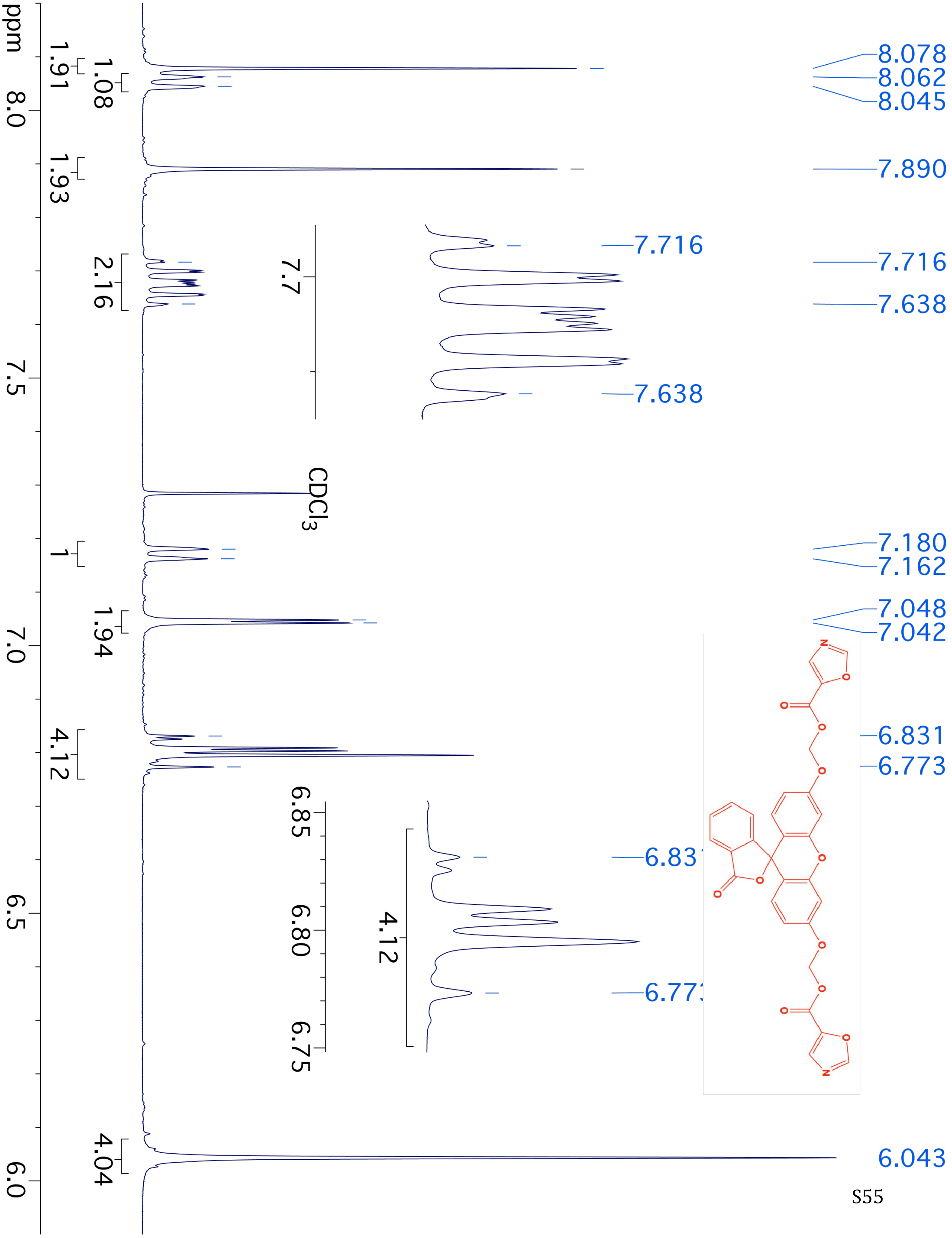
8.078
8.062
8.045
7.890
7.716
7.638
7.180
7.162
7.048
7.042
6.831
6.773
6.043
CDCl3
ppm
1.91
1.08
1.93
2.16
1
1.94
4.12
4.04

Figure S41

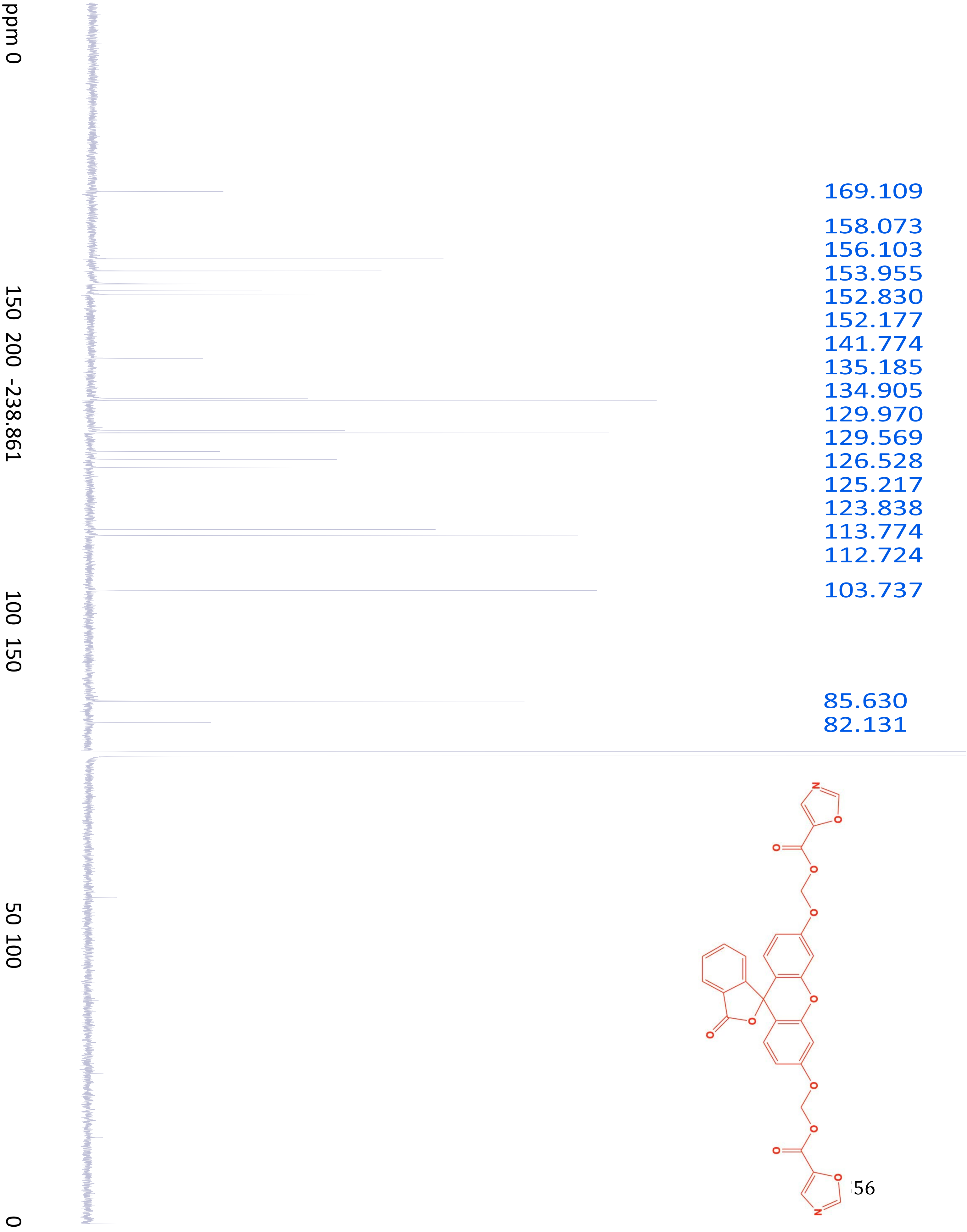
169.109
158.073
156.103
153.955
152.830
152.177
141.774
135.185
134.905
129.970
129.569
126.528
125.217
123.838
113.774
112.724
103.737
85.630
82.131

Figure S42

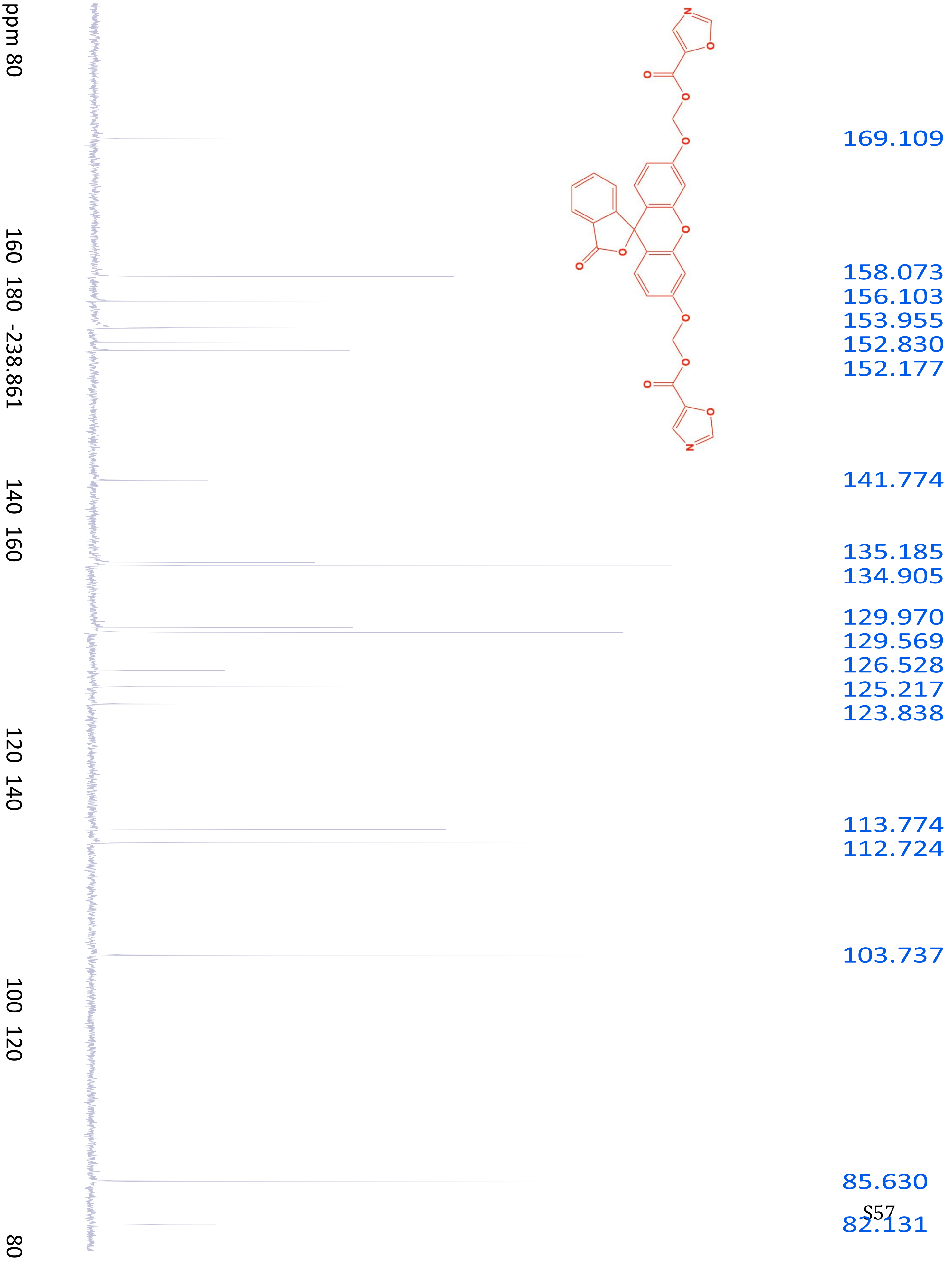
169.109
158.073
156.103
153.955
152.830
152.177
141.774
135.185
134.905
129.970
129.569
126.528
125.217
123.838
113.774
112.724
103.737
85.630
82.131

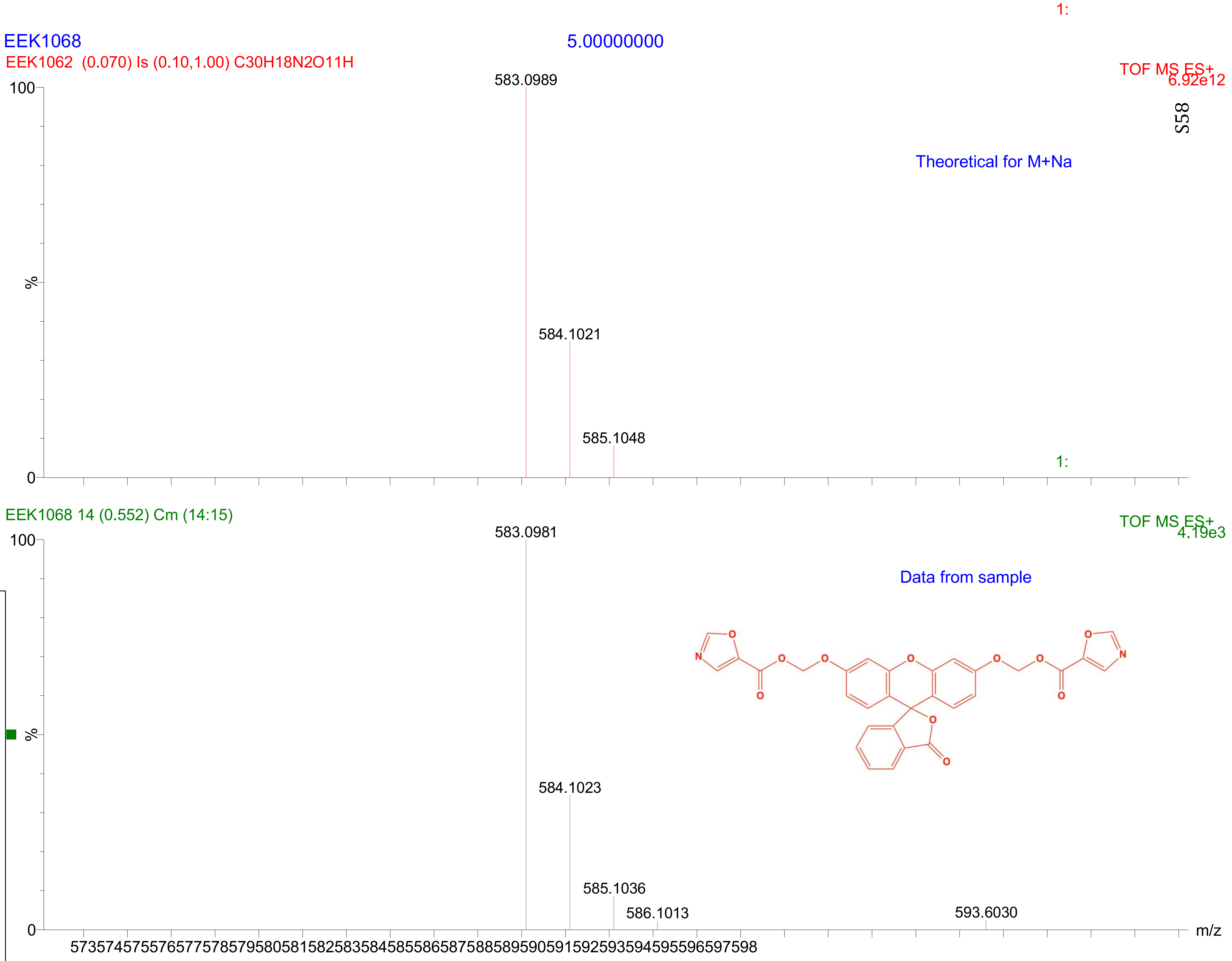

EEK1068
5.00000000
1:
EEK1062 (0.070) Is (0.10,1.00) C30H18N2O11H
TOF MS ES+
6.92e12
583.0989
584.1021
585.1048
Theoretical for M+Na
100
%
0
1:
EEK1068 14 (0.552) Cm (14:15)
TOF MS ES+
4.19e3
583.0981
584.1023
585.1036
586.1013
593.6030
Data from sample
573574575576577578579580581582583584585586587588589590591592593594595596597598
m/z

Figure S43

Figure S44

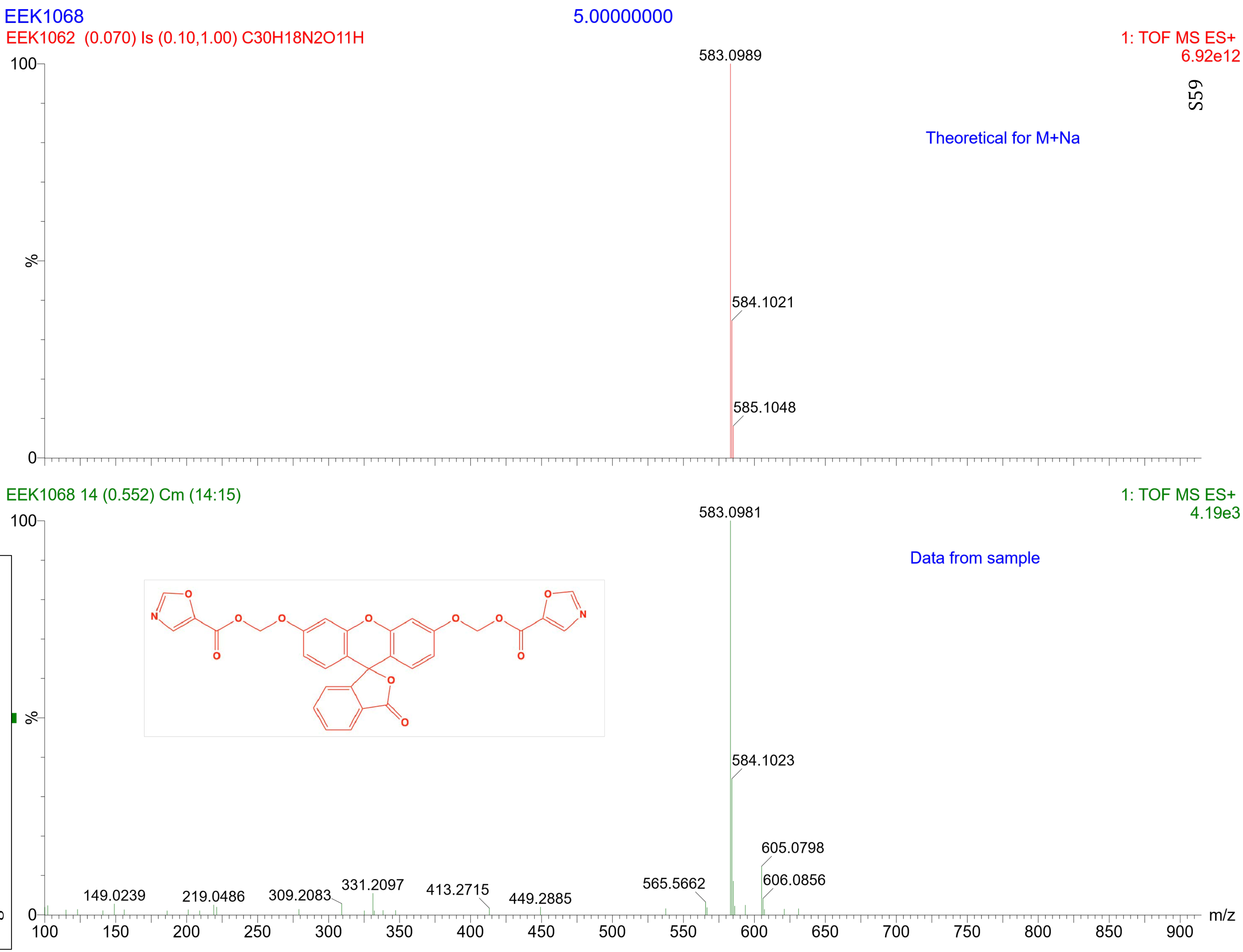

EEK1068
5.00000000
EEK1062 (0.070) Is (0.10,1.00) C30H18N2O11H
1: TOF MS ES+
6.92e12
583.0989
Theoretical for M+Na
584.1021
585.1048
EEK1068 14 (0.552) Cm (14:15)
1: TOF MS ES+
4.19e3
583.0981
Data from sample
584.1023
605.0798
606.0856
565.5662
449.2885
413.2715
331.2097
309.2083
219.0486
149.0239
100
%
0
m/z

Figure S45

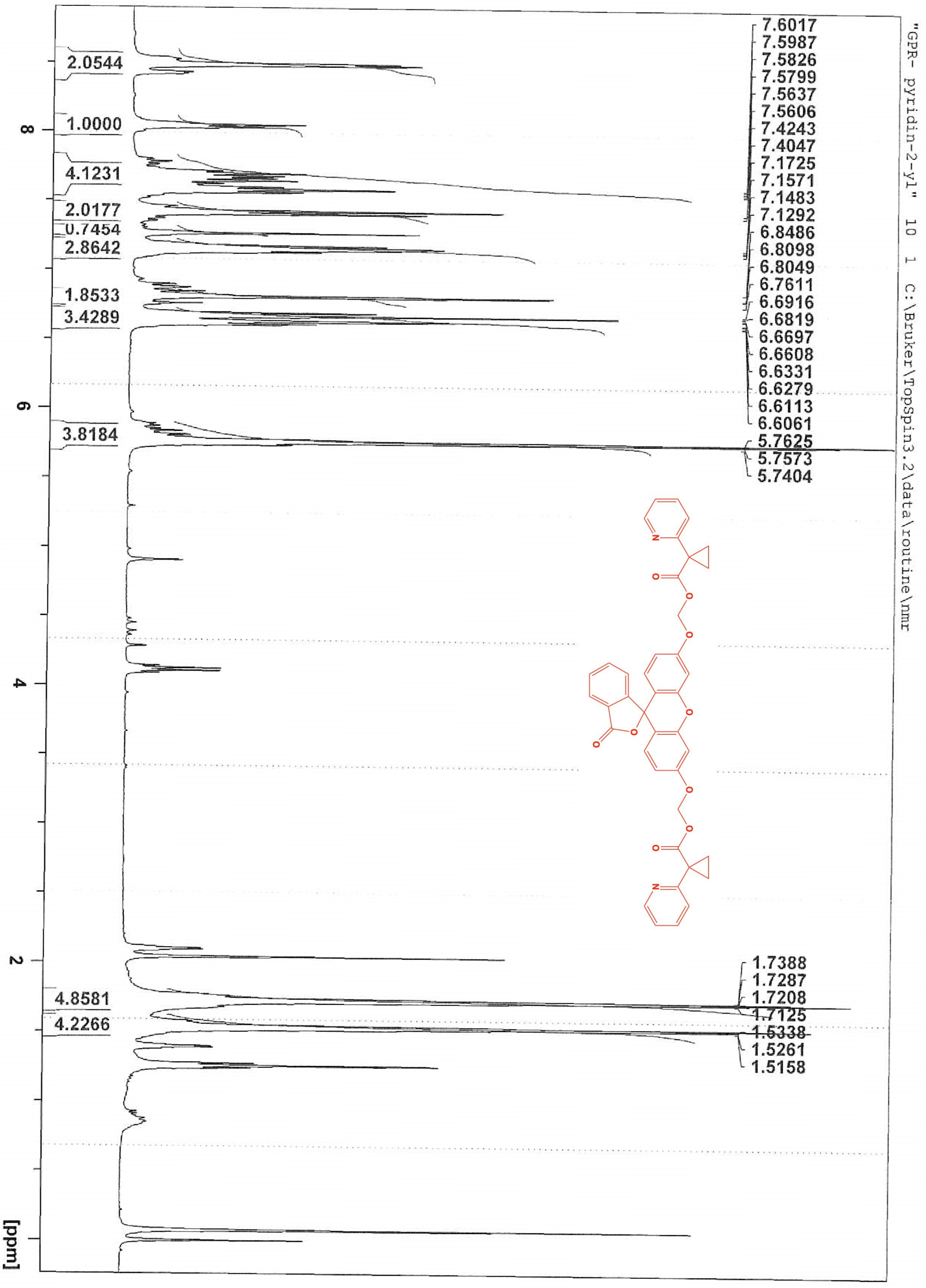
"GPR- pyridin-2-yl" 10 1 C:\Bruker\TopSpin3.2\data\routine\nmr
7.6017
7.5987
7.5826
7.5799
7.5637
7.5606
7.4243
7.4047
7.1725
7.1571
7.1483
7.1292
6.8486
6.8098
6.8049
6.7611
6.6916
6.6819
6.6697
6.6608
6.6331
6.6279
6.6113
6.6061
5.7625
5.7573
5.7404
1.7388
1.7287
1.7208
1.7125
1.5338
1.5261
1.5158
2.0544
1.0000
4.1231
2.0177
0.7454
2.8642
1.8533
3.4289
3.8184
4.8581
4.2266
8
6
4
2
[ppm]
N
O
O
O
O
O
O
O
O
O
N

Figure S46

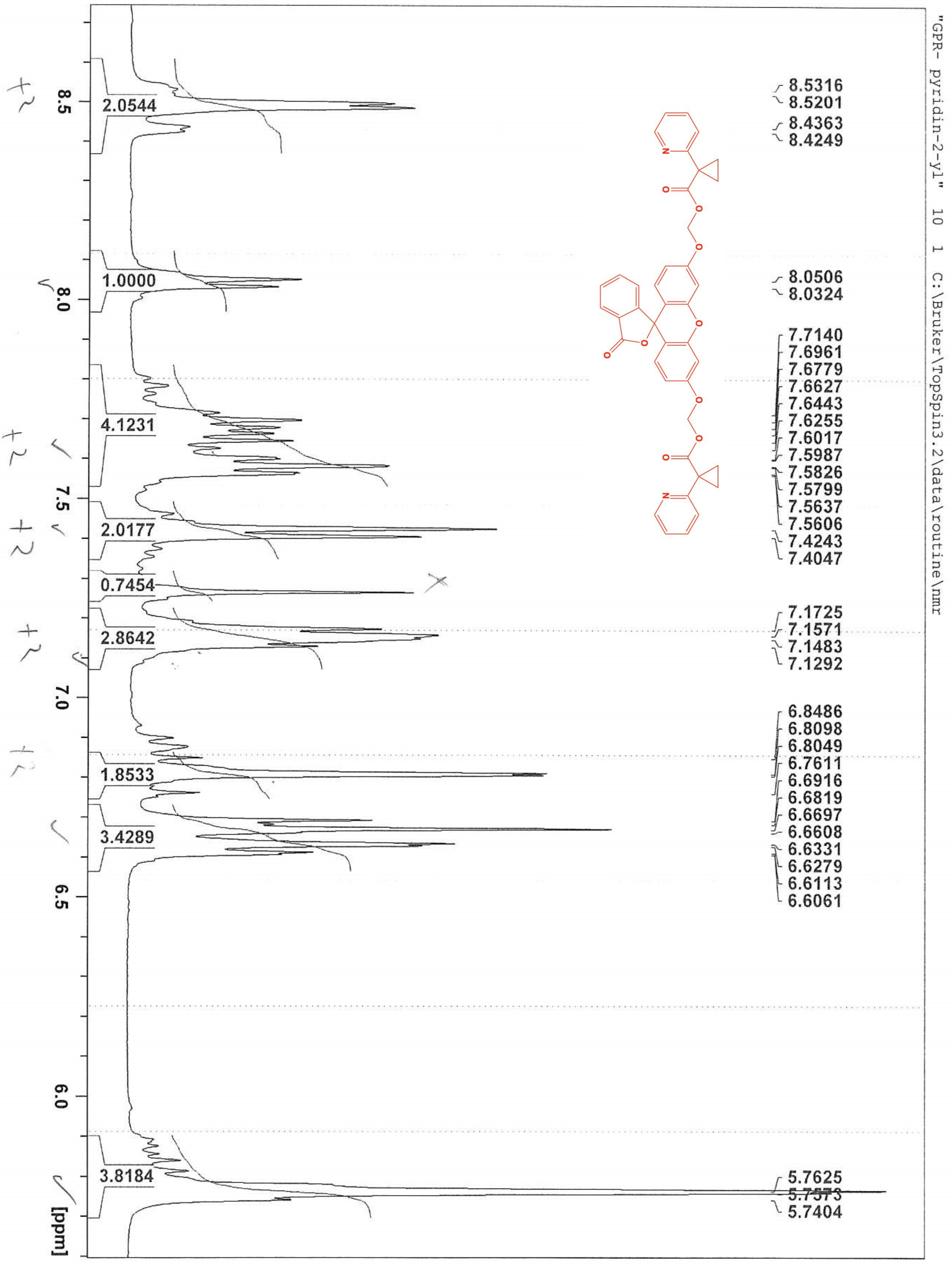
"GPR- pyridin-2-yl" 10 1 C:\Bruker\TopSpin3.2\data\routine\nmr
8.5316
8.5201
8.4363
8.4249
8.0506
8.0324
7.7140
7.6961
7.6779
7.6627
7.6443
7.6255
7.6017
7.5987
7.5826
7.5799
7.5637
7.5606
7.4243
7.4047
7.1725
7.1571
7.1483
7.1292
6.8486
6.8098
6.8049
6.7611
6.6916
6.6819
6.6697
6.6608
6.6331
6.6279
6.6113
6.6061
5.7625
5.7573
5.7404
2.0544
1.0000
4.1231
2.0177
0.7454
2.8642
1.8533
3.4289
3.8184
8.5
8.0
7.5
7.0
6.5
6.0
[ppm]

Figure S47

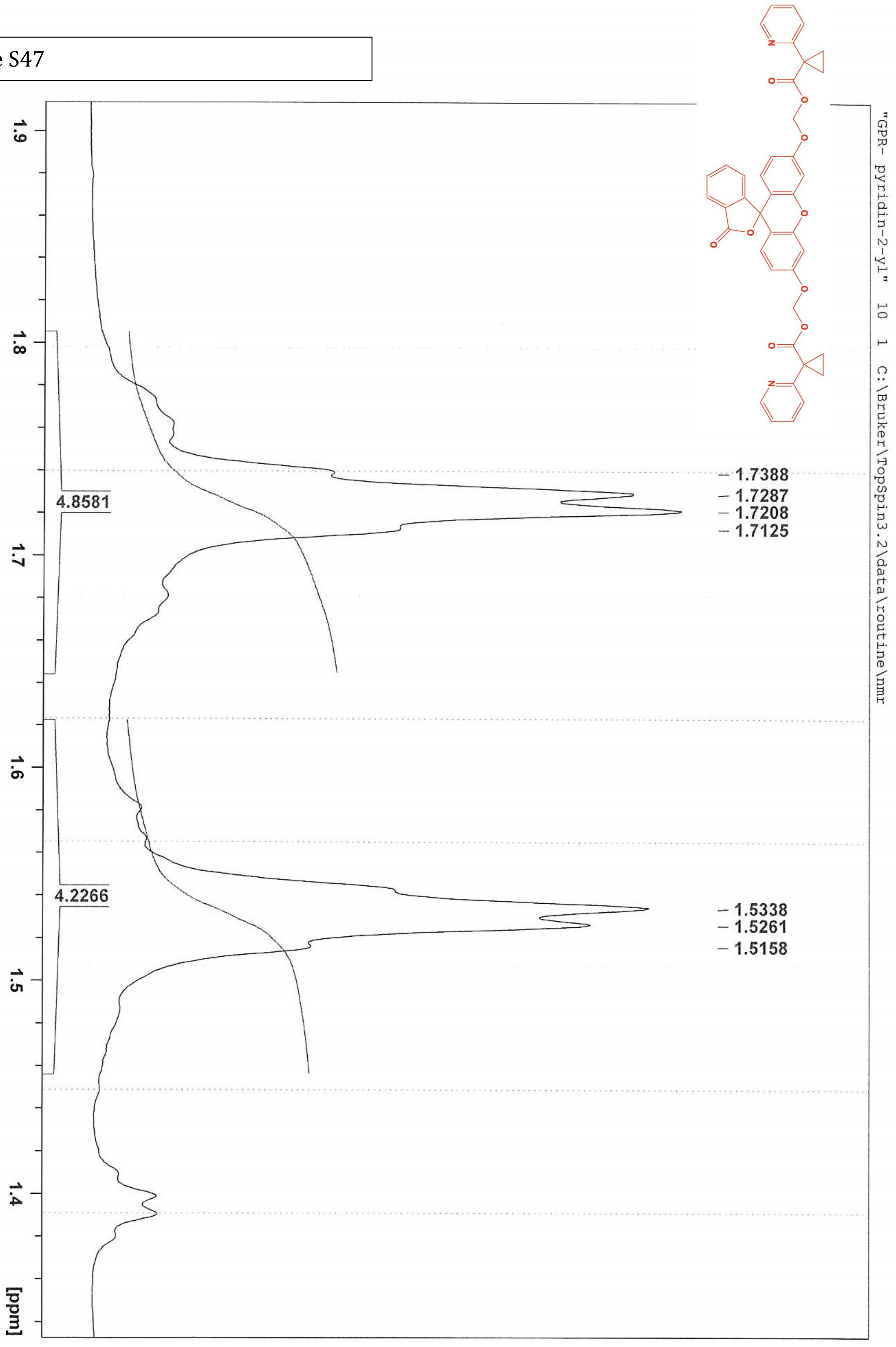
"GPR- pyridin-2-yl" 10 1 C:\Bruker\TopSpin3.2\data\routine\nmr
1.7388
1.7287
1.7208
1.7125
1.5338
1.5261
1.5158
4.8581
4.2266
1.9
1.8
1.7
1.6
1.5
1.4
[ppm]
N
O

Figure S48

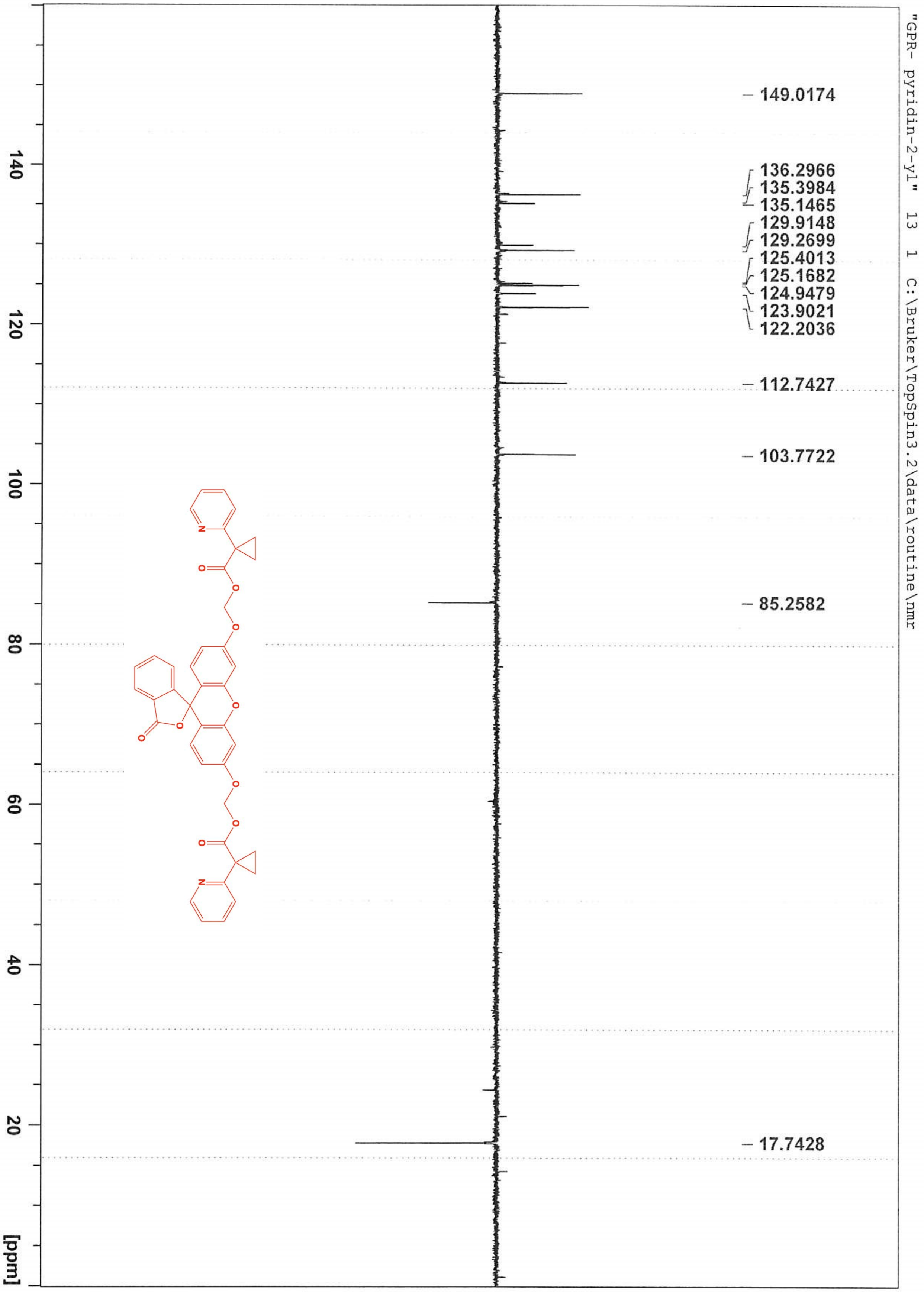
"GPR- pyridin-2-yl" 13 1 C:\Bruker\TopSpin3.2\data\routine\nmr
149.0174
136.2966
135.3984
135.1465
129.9148
129.2699
125.4013
125.1682
124.9479
123.9021
122.2036
112.7427
103.7722
85.2582
17.7428
140
120
100
80
60
40
20
[ppm]

Figure S49

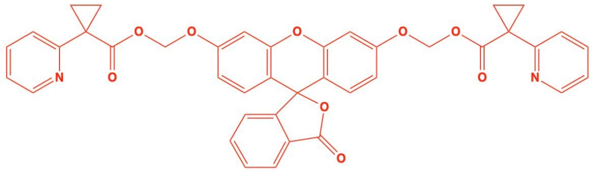

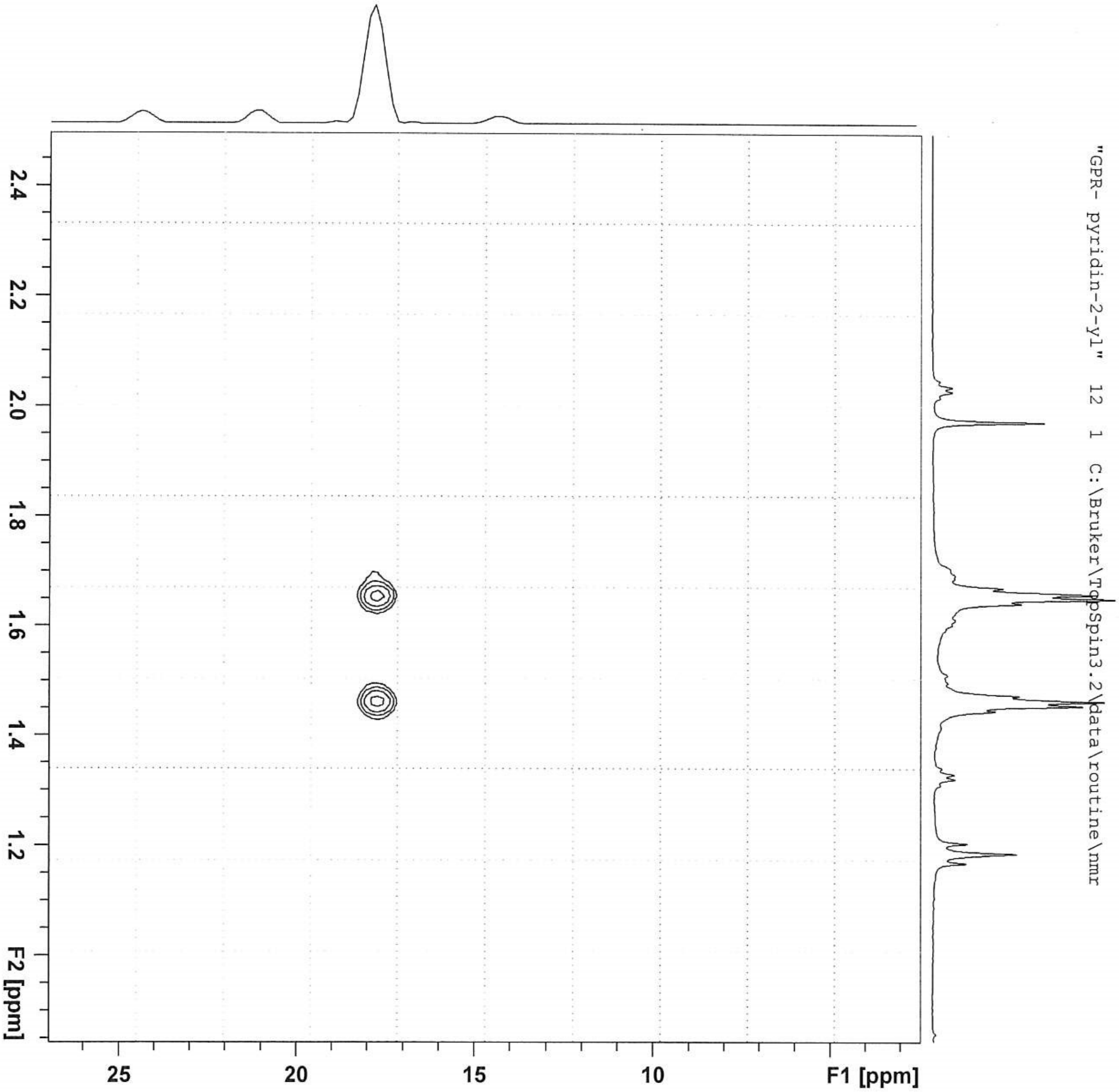
"GPR- pyridin-2-yl" 12 1 C:\Bruker\TopSpin3.2\data\routine\nmr
F2 [ppm]
F1 [ppm]
2.4
2.2
2.0
1.8
1.6
1.4
1.2
25
20
15
10

Figure S50

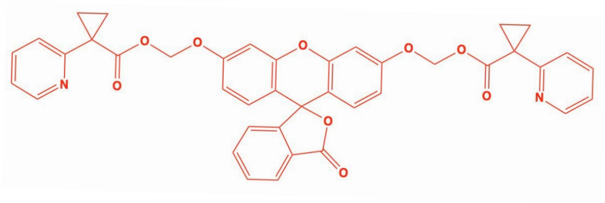

O
O
O
O
O
O
O
O
O
N
N

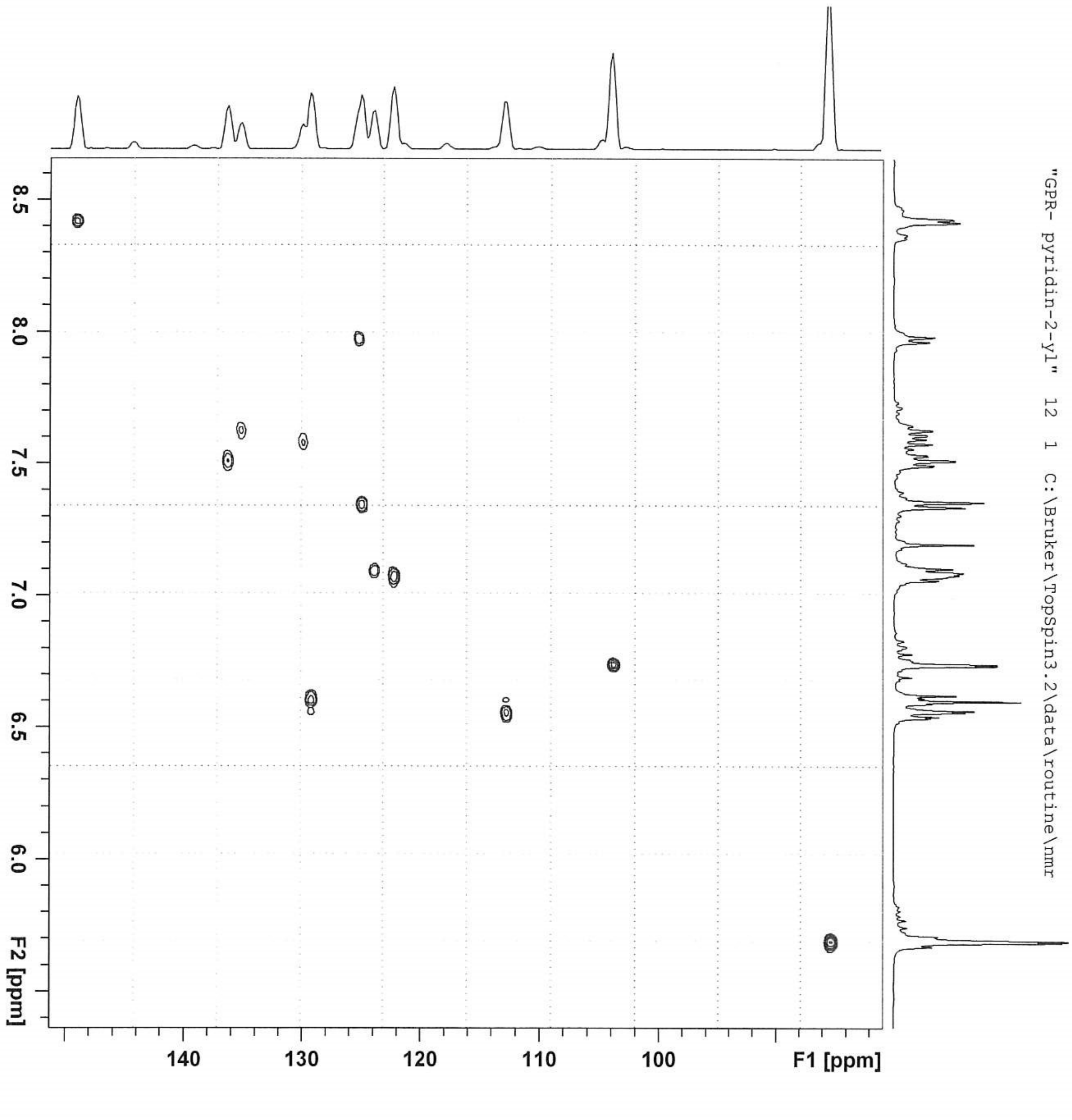

"GPR- pyridin-2-yl" 12 1 C:\Bruker\TopSpin3.2\data\routine\nmr
8.5
8.0
7.5
7.0
6.5
6.0
F2 [ppm]
140
130
120
110
100
F1 [ppm]

Figure S51

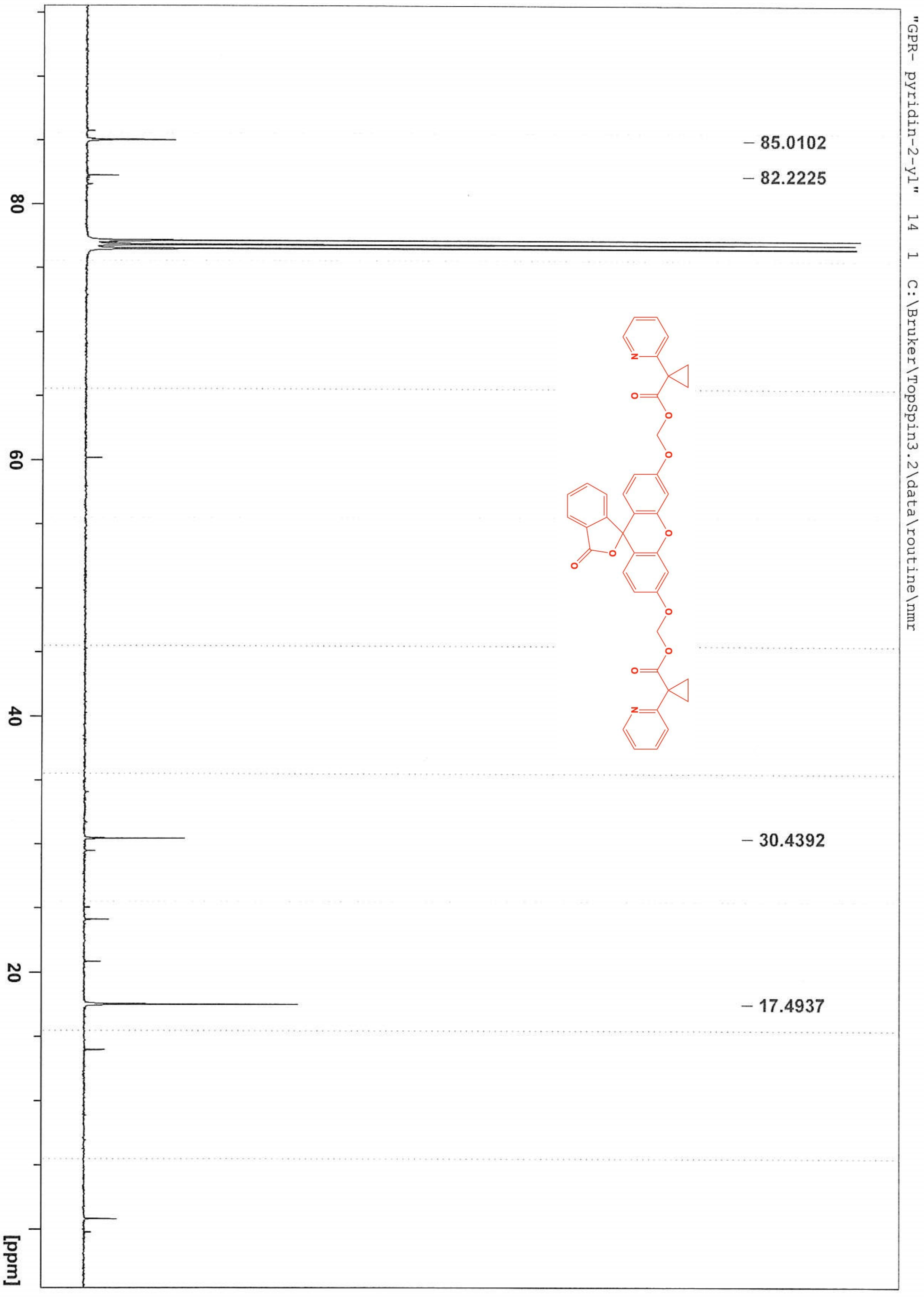
"GPR- pyridin-2-yl" 14 1 C:\Bruker\TopSpin3.2\data\routine\nmr
85.0102
82.2225
30.4392
17.4937
80
60
40
20
[ppm]

Figure S52

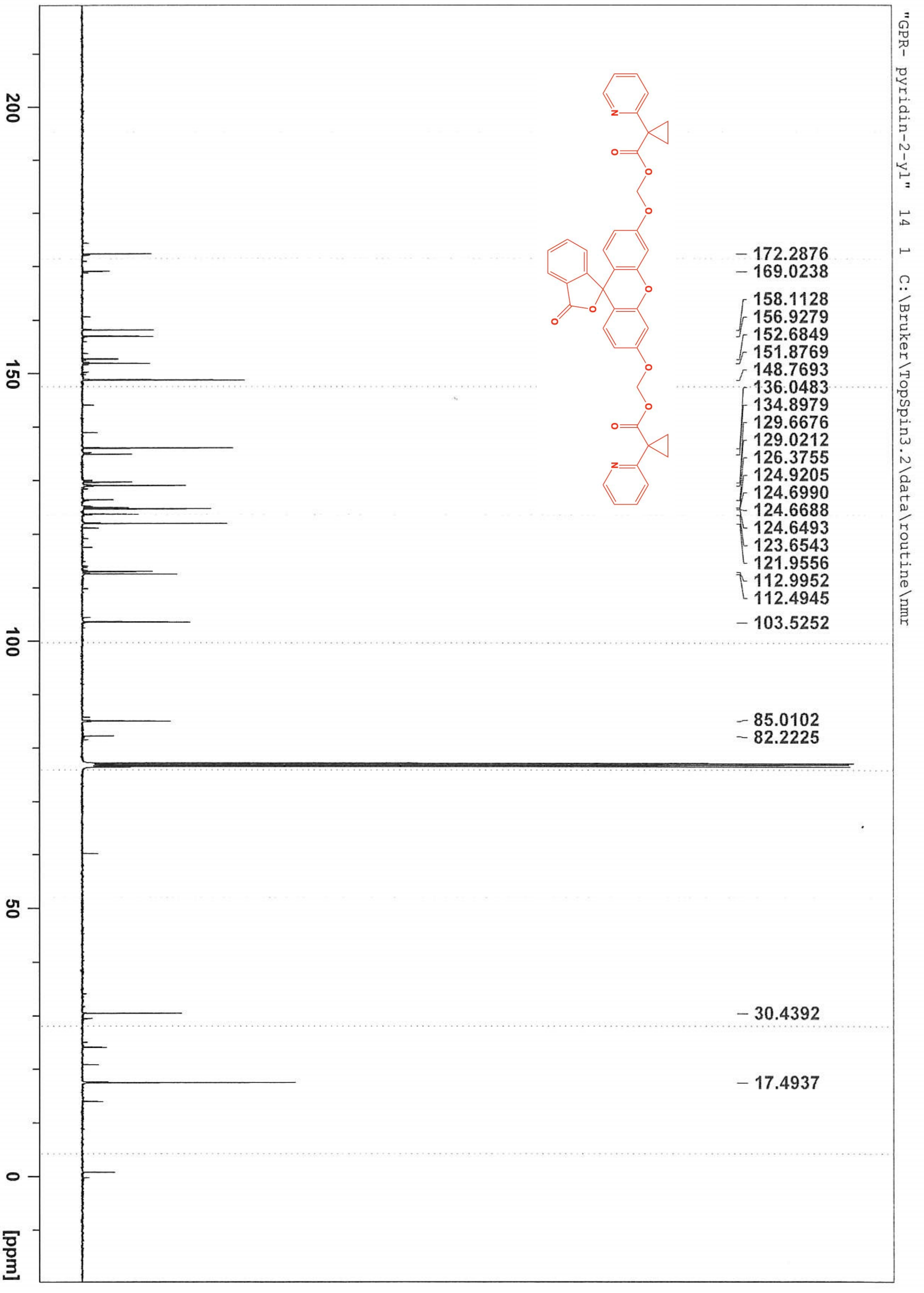
"GPR- pyridin-2-yl" 14 1 C:\Bruker\TopSpin3.2\data\routine\nmr
172.2876
169.0238
158.1128
156.9279
152.6849
151.8769
148.7693
136.0483
134.8979
129.6676
129.0212
126.3755
124.9205
124.6990
124.6688
124.6493
123.6543
121.9556
112.9952
112.4945
103.5252
85.0102
82.2225
30.4392
17.4937
200
150
100
50
0
[ppm]

Figure S53

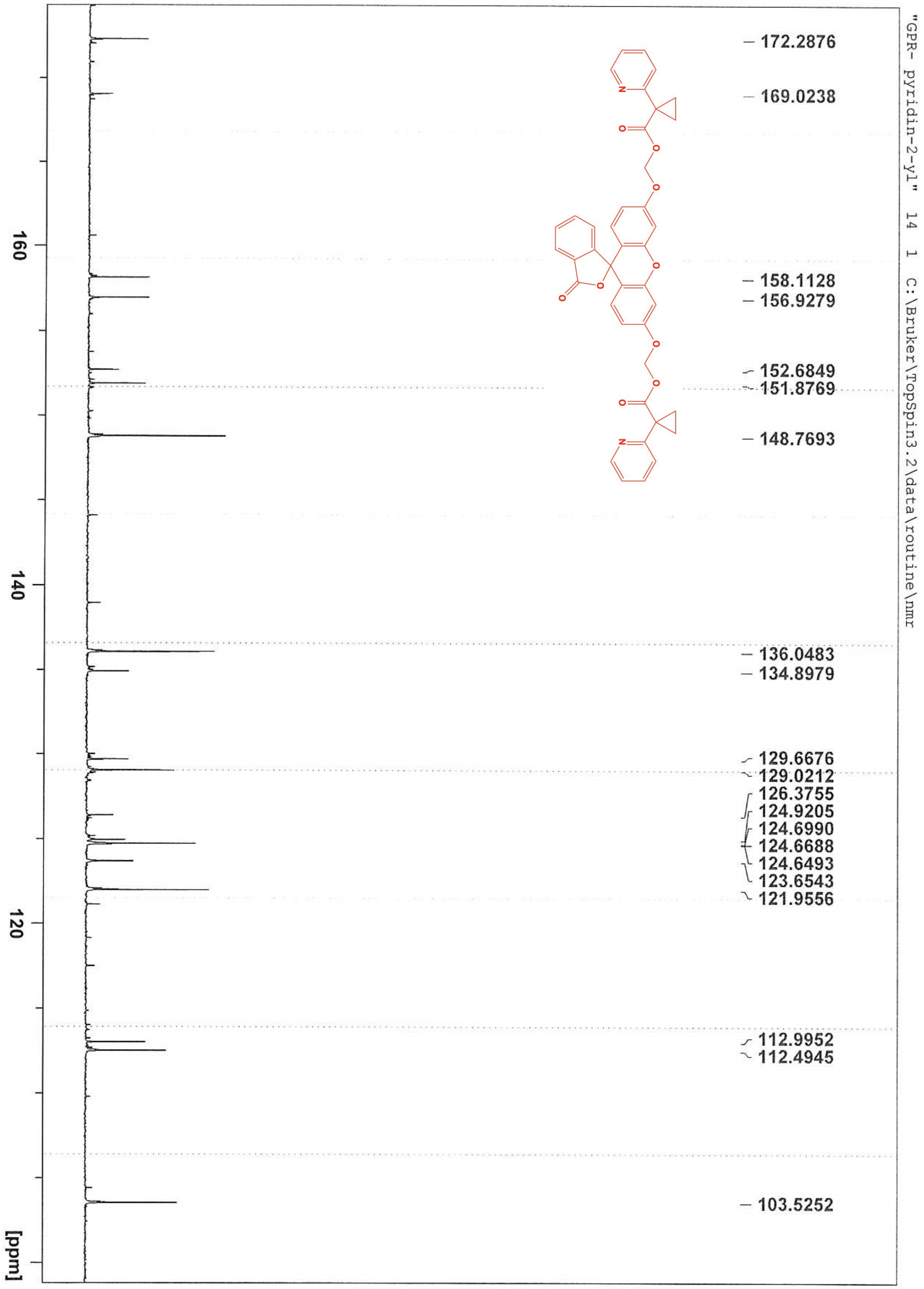

"GPR- pyridin-2-yl" 14 1 C:\Bruker\TopSpin3.2\data\routine\nmr
172.2876
169.0238
158.1128
156.9279
152.6849
151.8769
148.7693
136.0483
134.8979
129.6676
129.0212
126.3755
124.9205
124.6990
124.6688
124.6493
123.6543
121.9556
112.9952
112.4945
103.5252
160
140
120
[ppm]
N
O

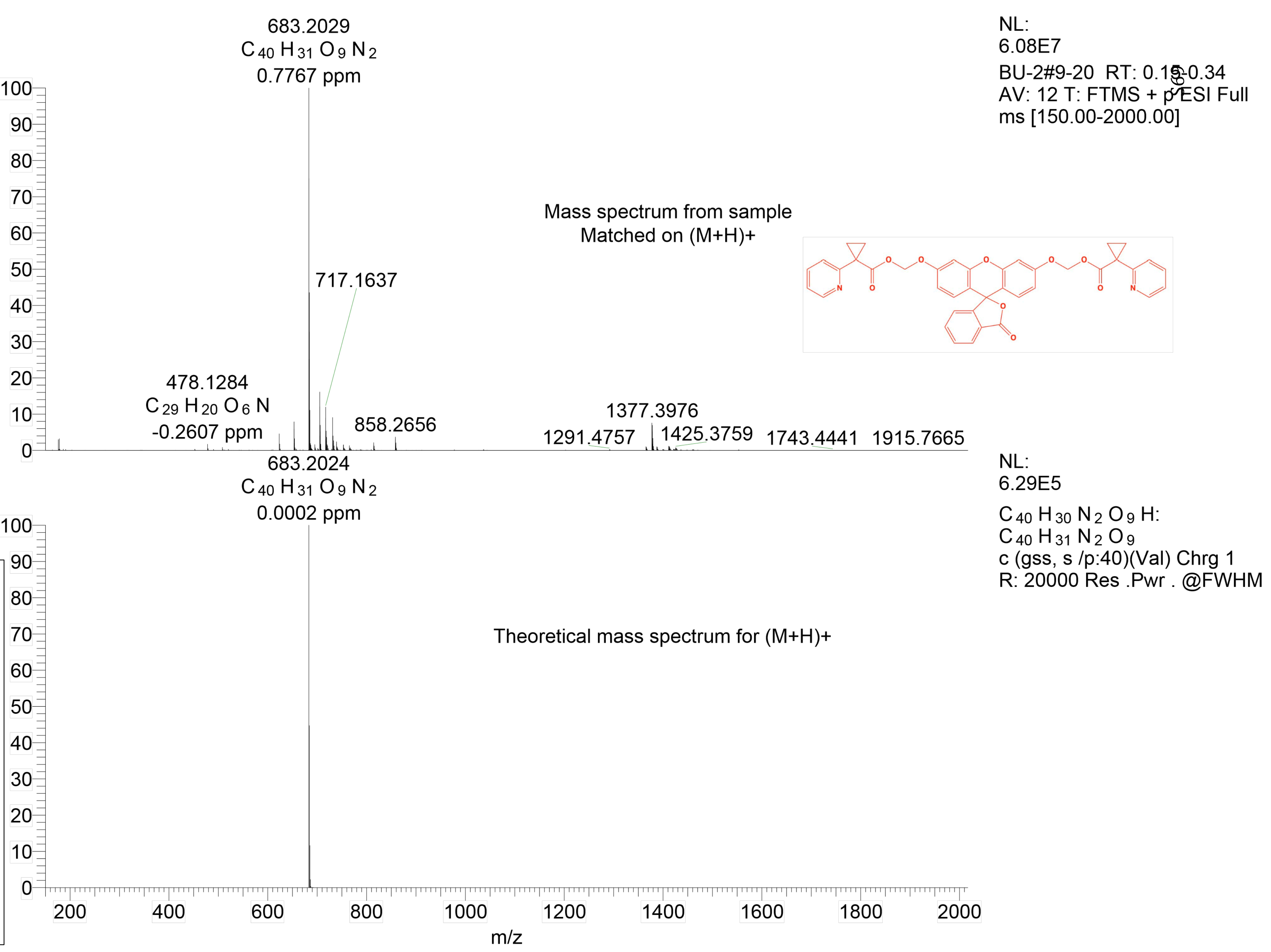
683.2029
C 40 H 31 O 9 N 2
0.7767 ppm
NL:
6.08E7
BU-2#9-20 RT: 0.15-0.34
AV: 12 T: FTMS + p ESI Full ms [150.00-2000.00]
Mass spectrum from sample
Matched on (M+H)+
717.1637
478.1284
C 29 H 20 O 6 N
-0.2607 ppm
858.2656
1377.3976
1291.4757
1425.3759
1743.4441
1915.7665
Relative Abundance
683.2024
C 40 H 31 O 9 N 2
0.0002 ppm
NL:
6.29E5
C 40 H 30 N 2 O 9 H:
C 40 H 31 N 2 O 9
c (gss, s /p:40)(Val) Chrg 1
R: 20000 Res .Pwr . @FWHM
Theoretical mass spectrum for (M+H)+
m/z

Figure S54

Figure S55

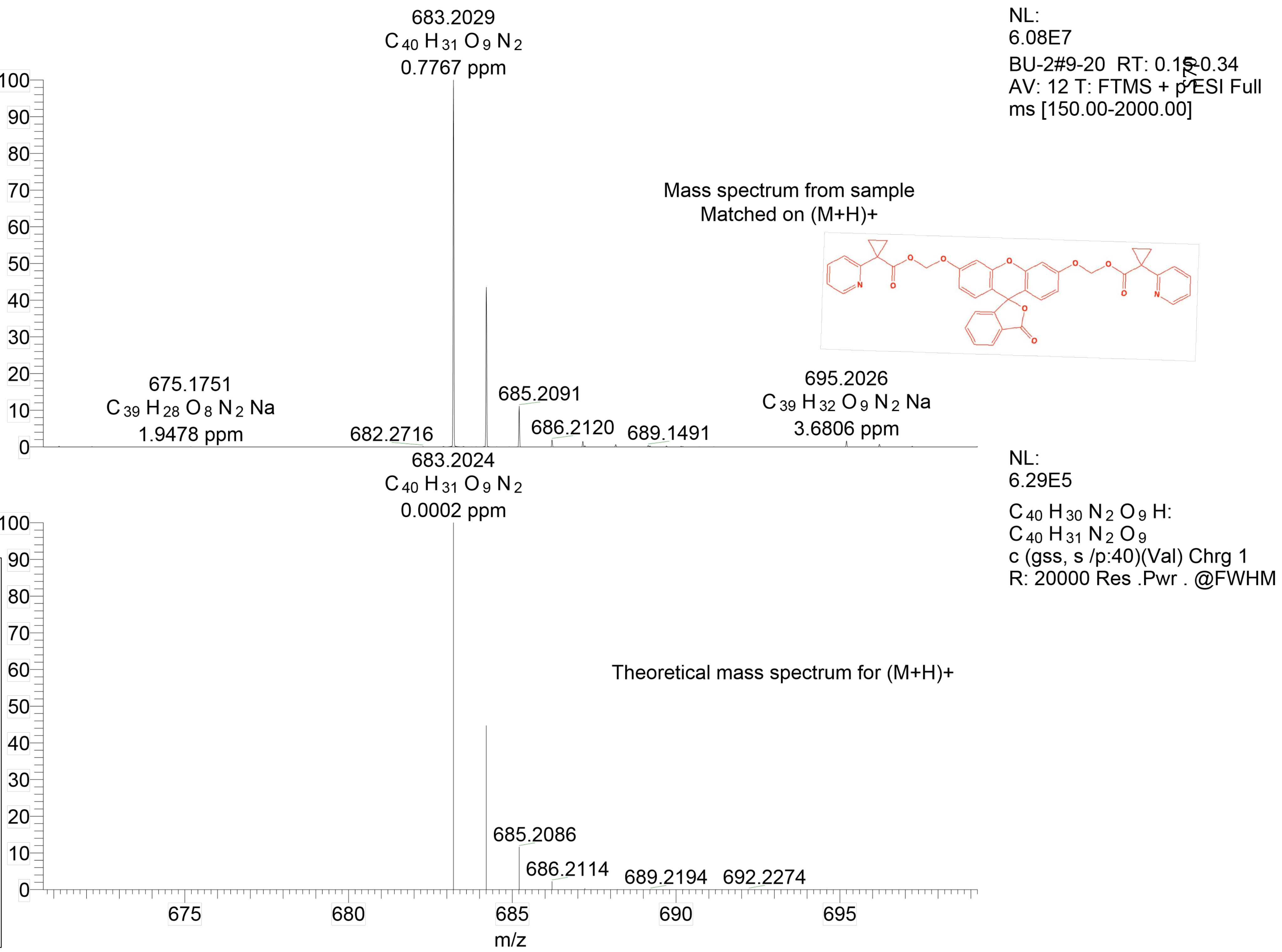
683.2029
C 40 H 31 O 9 N 2
0.7767 ppm
NL:
6.08E7
BU-2#9-20 RT: 0.15-0.34
AV: 12 T: FTMS + p ESI Full
ms [150.00-2000.00]
Mass spectrum from sample
Matched on (M+H)+
Relative Abundance
675.1751
C 39 H 28 O 8 N 2 Na
1.9478 ppm
682.2716
685.2091
686.2120
689.1491
695.2026
C 39 H 32 O 9 N 2 Na
3.6806 ppm
683.2024
C 40 H 31 O 9 N 2
0.0002 ppm
NL:
6.29E5
C 40 H 30 N 2 O 9 H:
C 40 H 31 N 2 O 9
c (gss, s /p:40)(Val) Chrg 1
R: 20000 Res .Pwr . @FWHM
Theoretical mass spectrum for (M+H)+
685.2086
686.2114
689.2194
692.2274
m/z

Figure S56

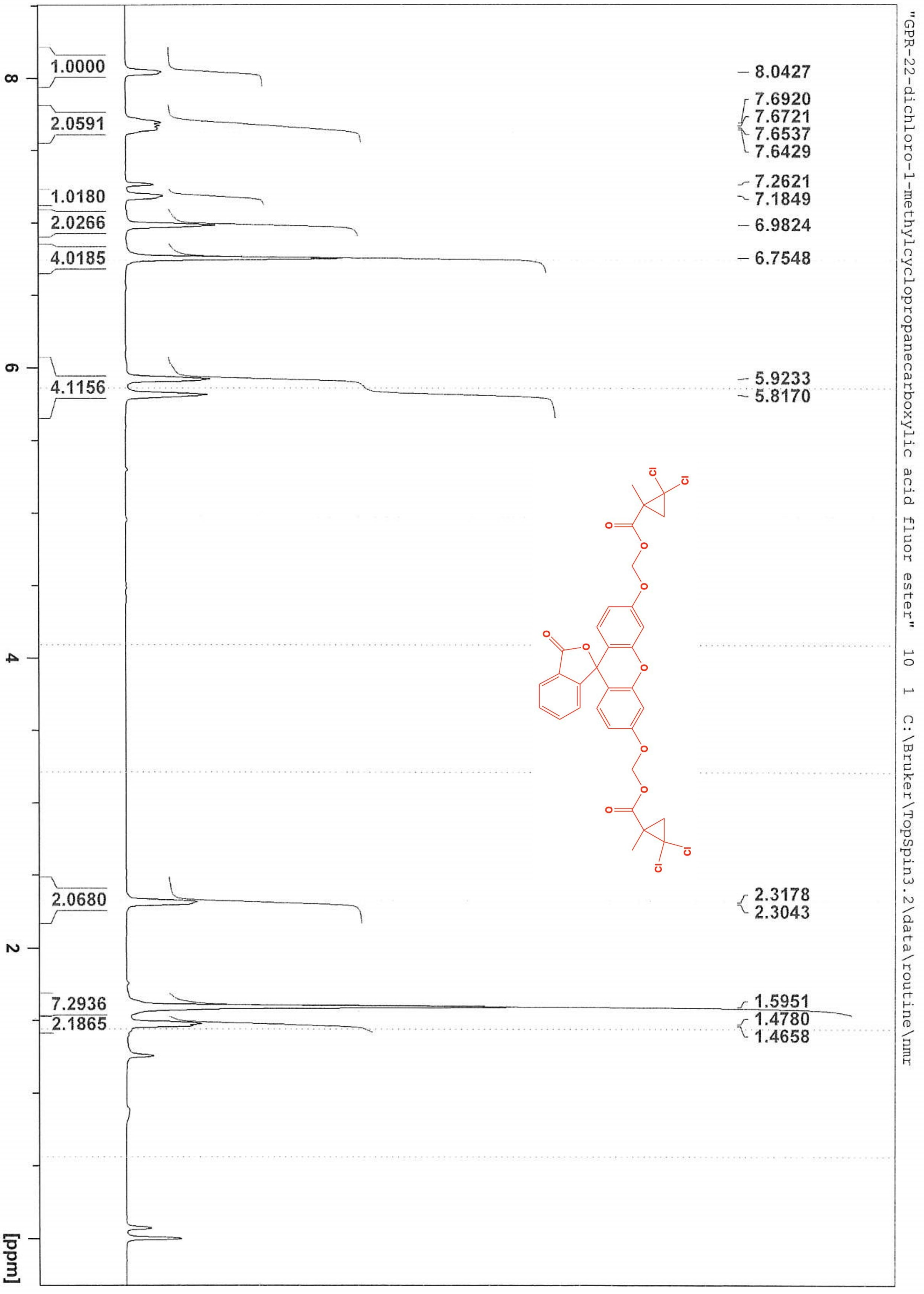

"GPR-22-dichloro-1-methylcyclopropanecarboxylic acid fluor ester" 10 1 C:\Bruker\TopSpin3.2\data\routine\nmr
8.0427
7.6920
7.6721
7.6537
7.6429
7.2621
7.1849
6.9824
6.7548
5.9233
5.8170
2.3178
2.3043
1.5951
1.4780
1.4658
1.0000
2.0591
1.0180
2.0266
4.0185
4.1156
2.0680
7.2936
2.1865
8
6
4
2
[ppm]

Figure S57

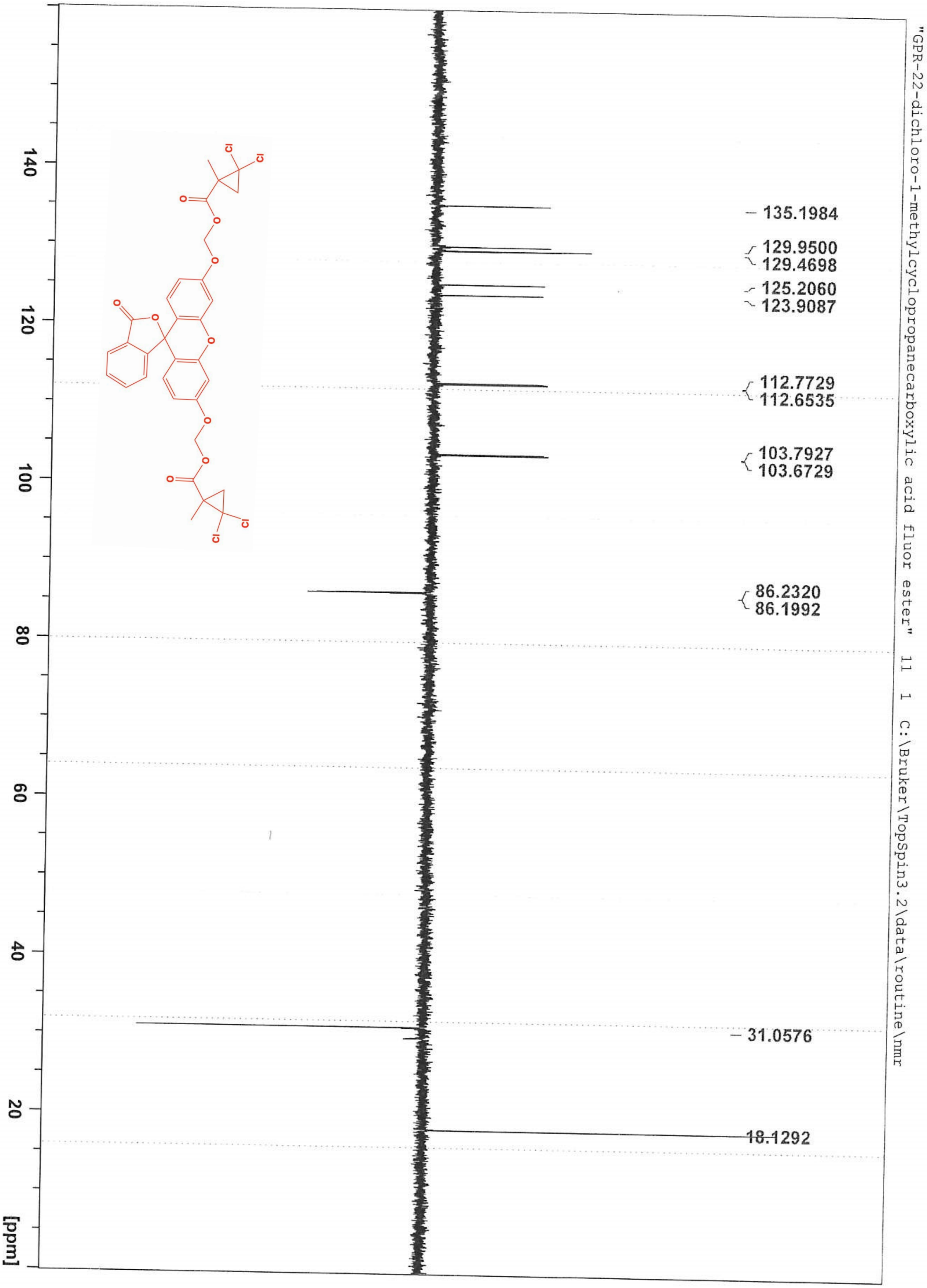
"GPR-22-dichloro-1-methylcyclopropanecarboxylic acid fluor ester" 11 1 C:\Bruker\TopSpin3.2\data\routine\nmr
135.1984
129.9500
129.4698
125.2060
123.9087
112.7729
112.6535
103.7927
103.6729
86.2320
86.1992
31.0576
18.1292
140
120
100
80
60
40
20
[ppm]
Cl
Cl
O
O
O
O
O
O
O
O
O
O
O
Cl
Cl

Figure S58

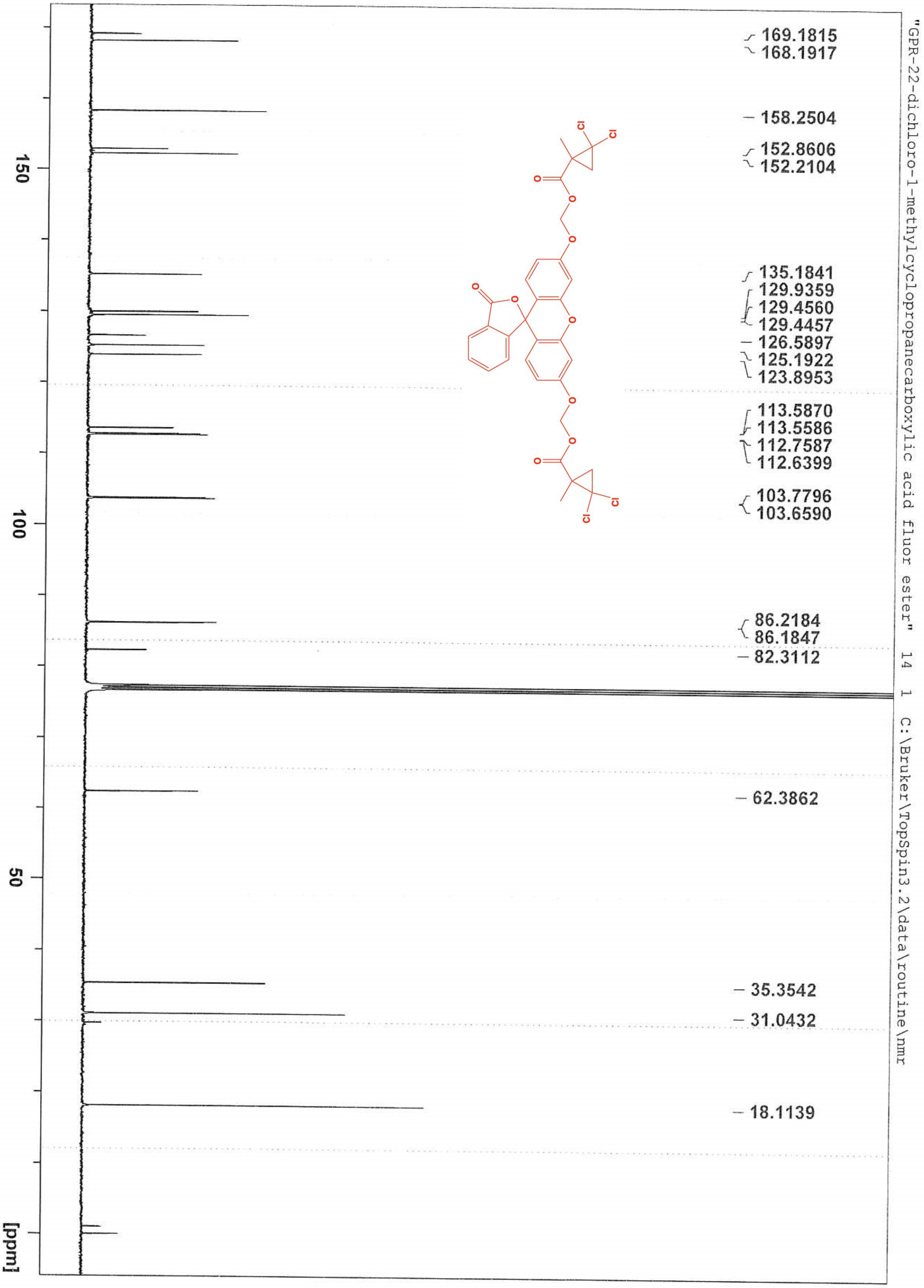
"GPR-22-dichloro-1-methylcyclopropanecarboxylic acid fluor ester" 14 1 C:\Bruker\TopSpin3.2\data\routine\nmr
169.1815
168.1917
158.2504
152.8606
152.2104
135.1841
129.9359
129.4560
129.4457
126.5897
125.1922
123.8953
113.5870
113.5586
112.7587
112.6399
103.7796
103.6590
86.2184
86.1847
82.3112
62.3862
35.3542
31.0432
18.1139
150
100
50
[ppm]
Cl
O

Figure S59

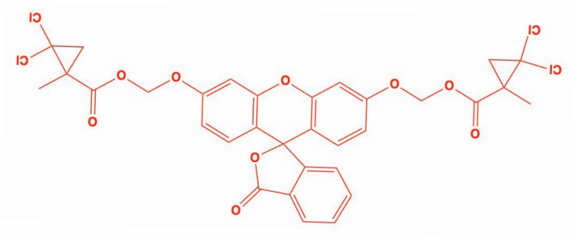

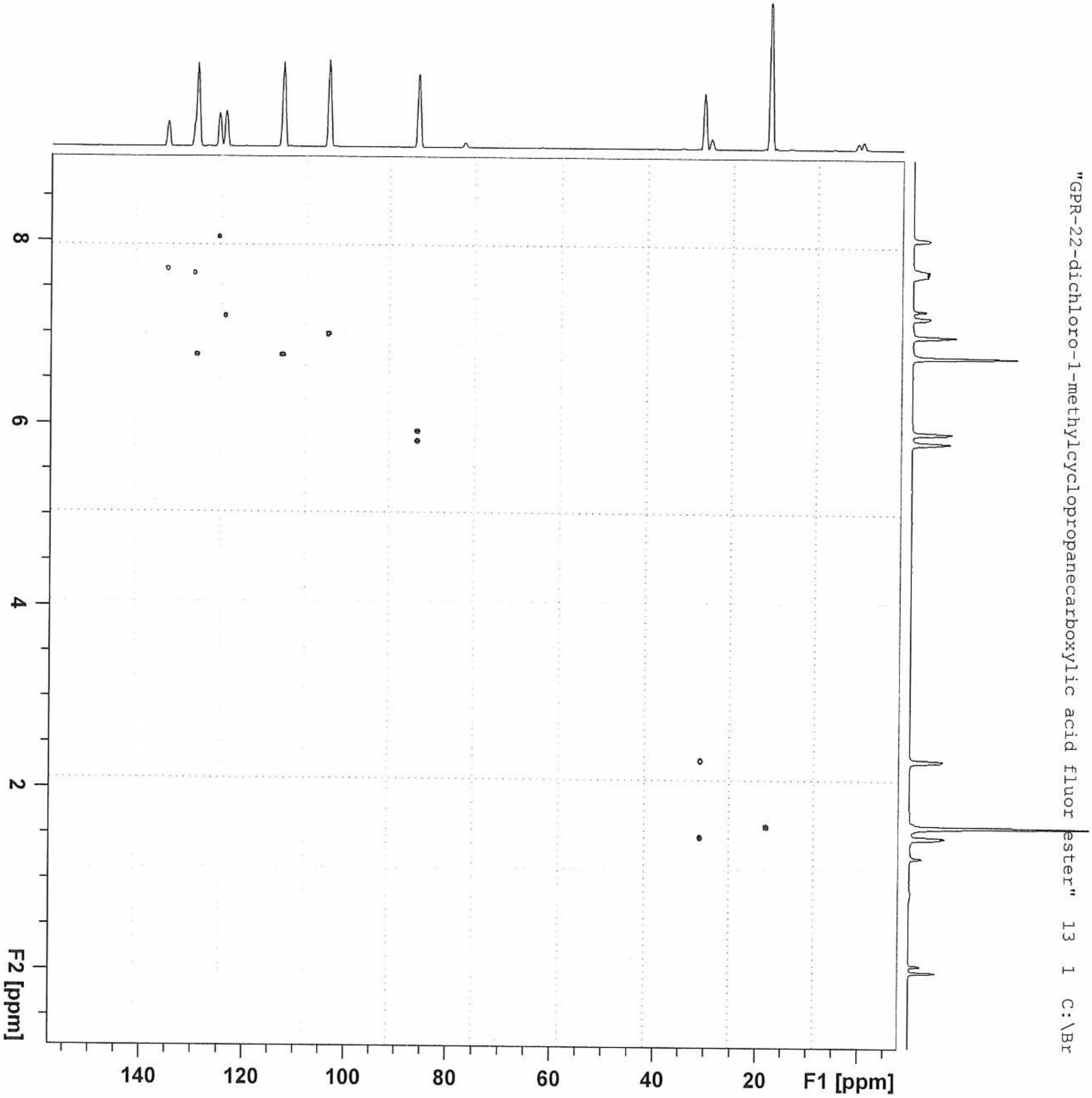
"GPR-22-dichloro-1-methylcyclopropanecarboxylic acid fluor ester" 13 1 C:\Br
F1 [ppm]
F2 [ppm]
140
120
100
80
60
40
20
8
6
4
2

Figure S60

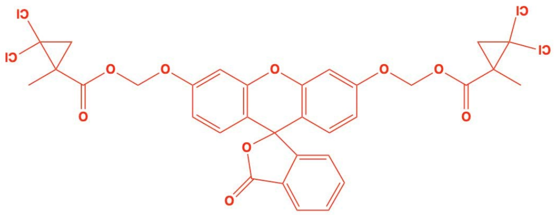
Cl
Cl
O
O
O
O
O
Cl
Cl
O
O
O
O

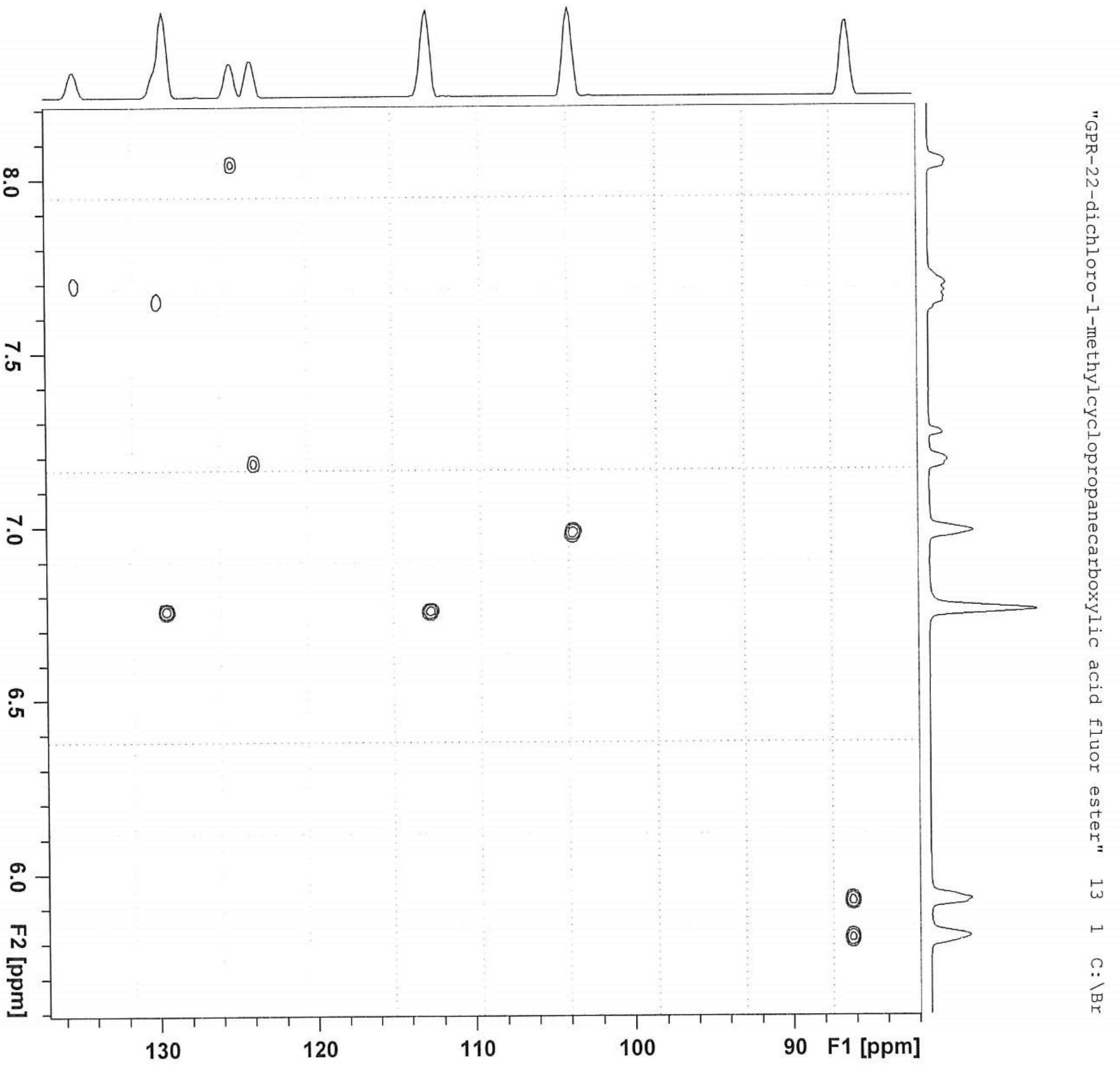
"GPR-22-dichloro-1-methylcyclopropanecarboxylic acid fluor ester" 13 1 C:\Br
F1 [ppm]
130
120
110
100
90
F2 [ppm]
8.0
7.5
7.0
6.5
6.0

Figure S61

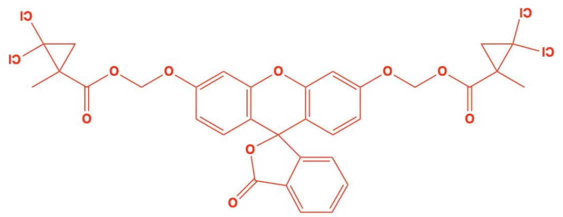
Cl
Cl
O
O
O
O
O
O
O
O
O
Cl
Cl
O
O

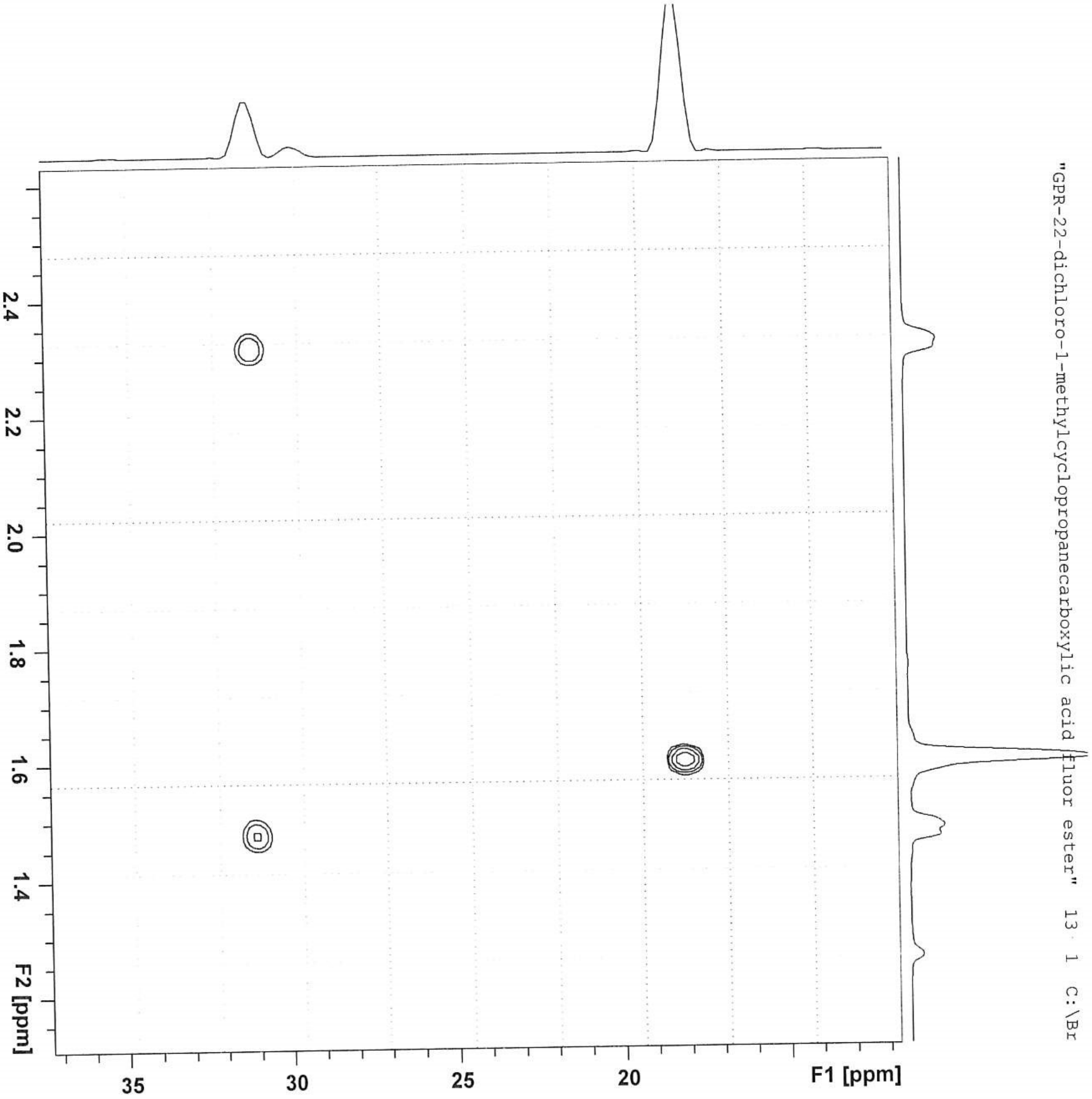
"GPR-22-dichloro-1-methylcyclopropanecarboxylic acid fluor ester" 13 1 C:\Br
F2 [ppm]
2.4
2.2
2.0
1.8
1.6
1.4
F1 [ppm]
35
30
25
20

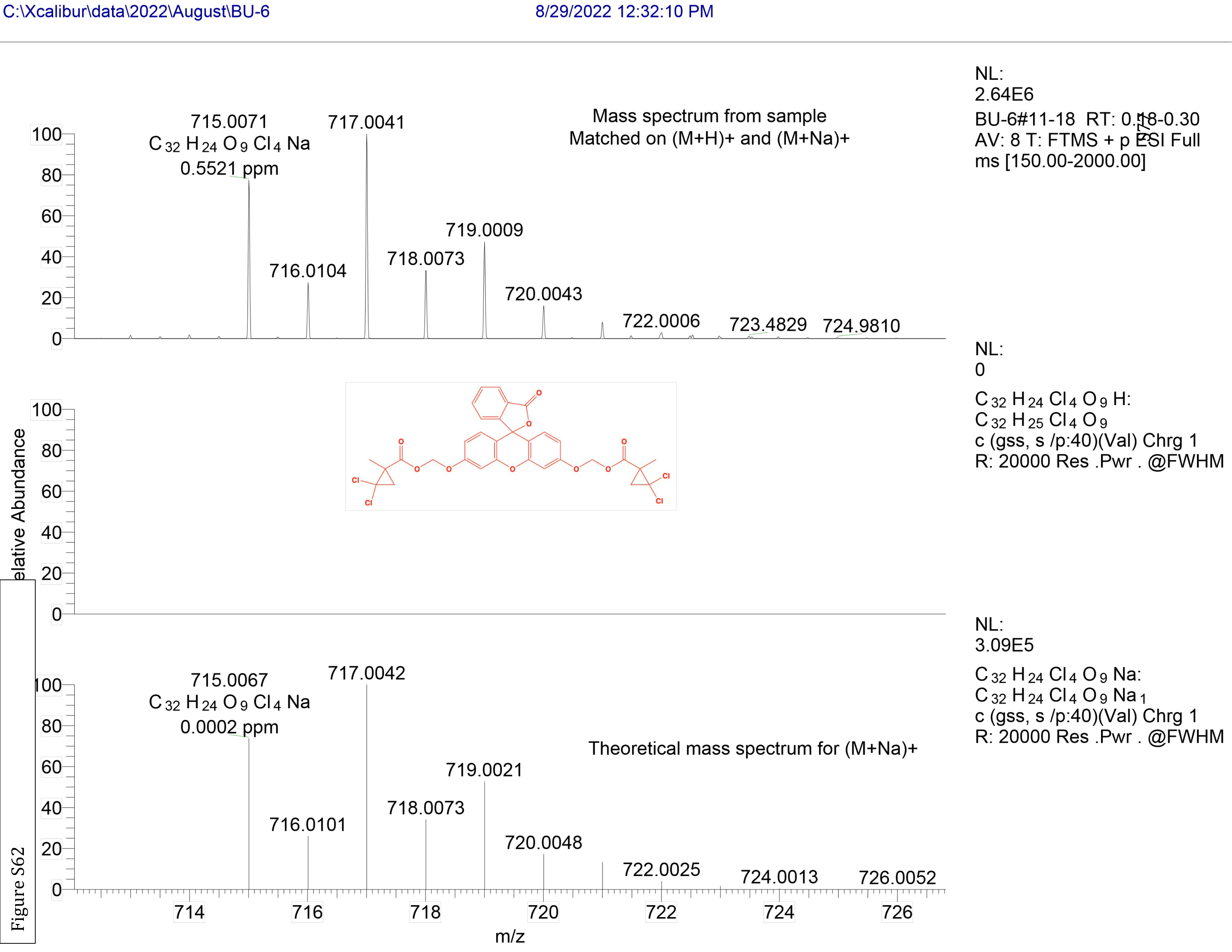
C:\Xcalibur\data\2022\August\BU-6
8/29/2022 12:32:10 PM
NL:
2.64E6
BU-6#11-18 RT: 0.18-0.30
AV: 8 T: FTMS + p ESI Full
ms [150.00-2000.00]
Mass spectrum from sample
Matched on (M+H)+ and (M+Na)+
715.0071
C 32 H 24 O 9 Cl 4 Na
0.5521 ppm
716.0104
717.0041
718.0073
719.0009
720.0043
722.0006
723.4829
724.9810
NL:
0
C 32 H 24 Cl 4 O 9 H:
C 32 H 25 Cl 4 O 9
c (gss, s /p:40)(Val) Chrg 1
R: 20000 Res .Pwr . @FWHM
O
Cl
NL:
3.09E5
C 32 H 24 Cl 4 O 9 Na:
C 32 H 24 Cl 4 O 9 Na 1
c (gss, s /p:40)(Val) Chrg 1
R: 20000 Res .Pwr . @FWHM
Theoretical mass spectrum for (M+Na)+
715.0067
C 32 H 24 O 9 Cl 4 Na
0.0002 ppm
716.0101
717.0042
718.0073
719.0021
720.0048
722.0025
724.0013
726.0052
Relative Abundance
m/z
0
20
40
60
80
100
714
716
718
720
722
724
726

Figure S62

Figure S63

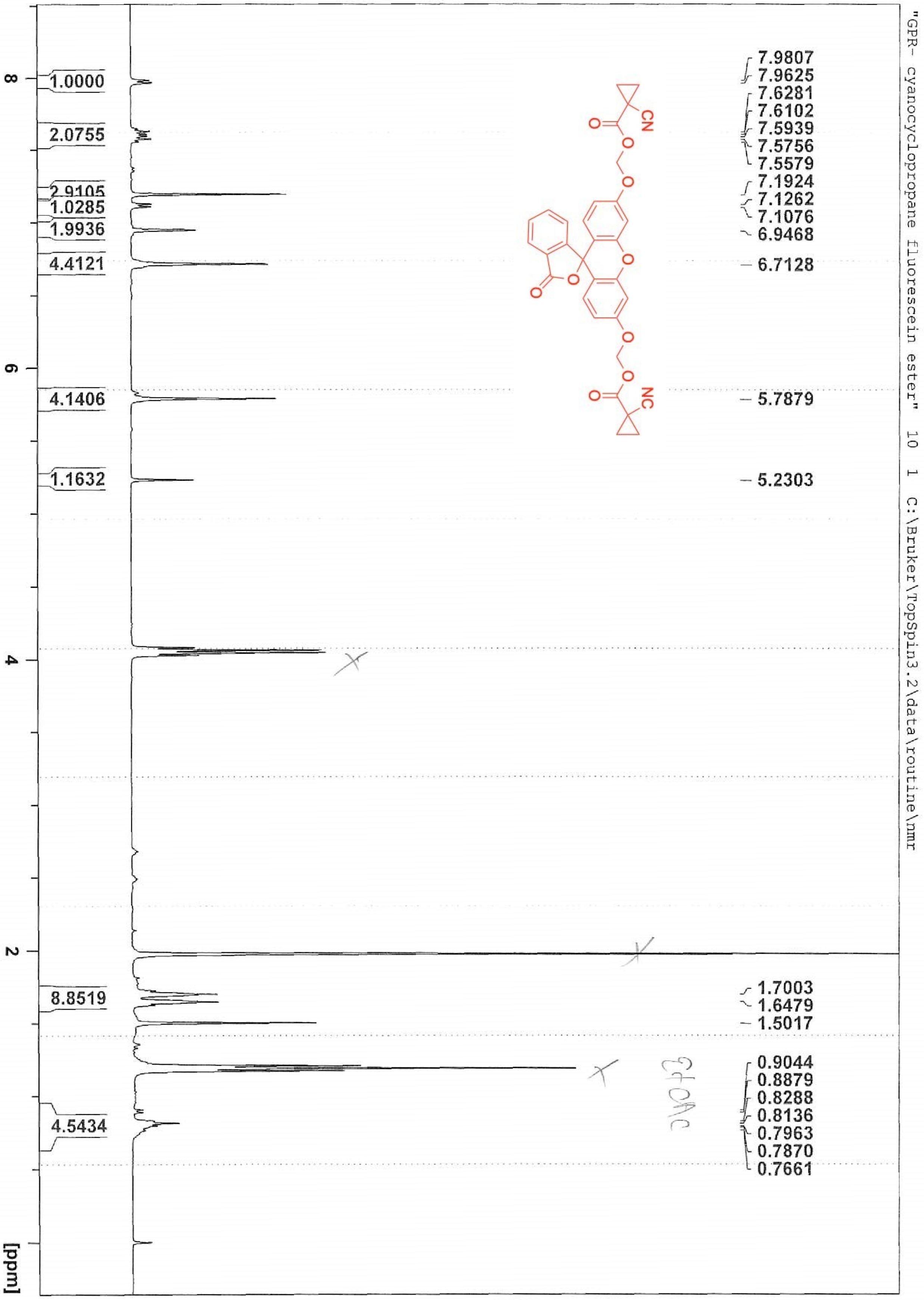

"GPR- cyanocyclopropane fluorescein ester" 10 1 C:\Bruker\TopSpin3.2\data\routine\nmr
7.9807
7.9625
7.6281
7.6102
7.5939
7.5756
7.5579
7.1924
7.1262
7.1076
6.9468
6.7128
5.7879
5.2303
1.7003
1.6479
1.5017
0.9044
0.8879
0.8288
0.8136
0.7963
0.7870
0.7661
1.0000
2.0755
2.9105
1.0285
1.9936
4.4121
4.1406
1.1632
8.8519
4.5434
[ppm]

Figure S64

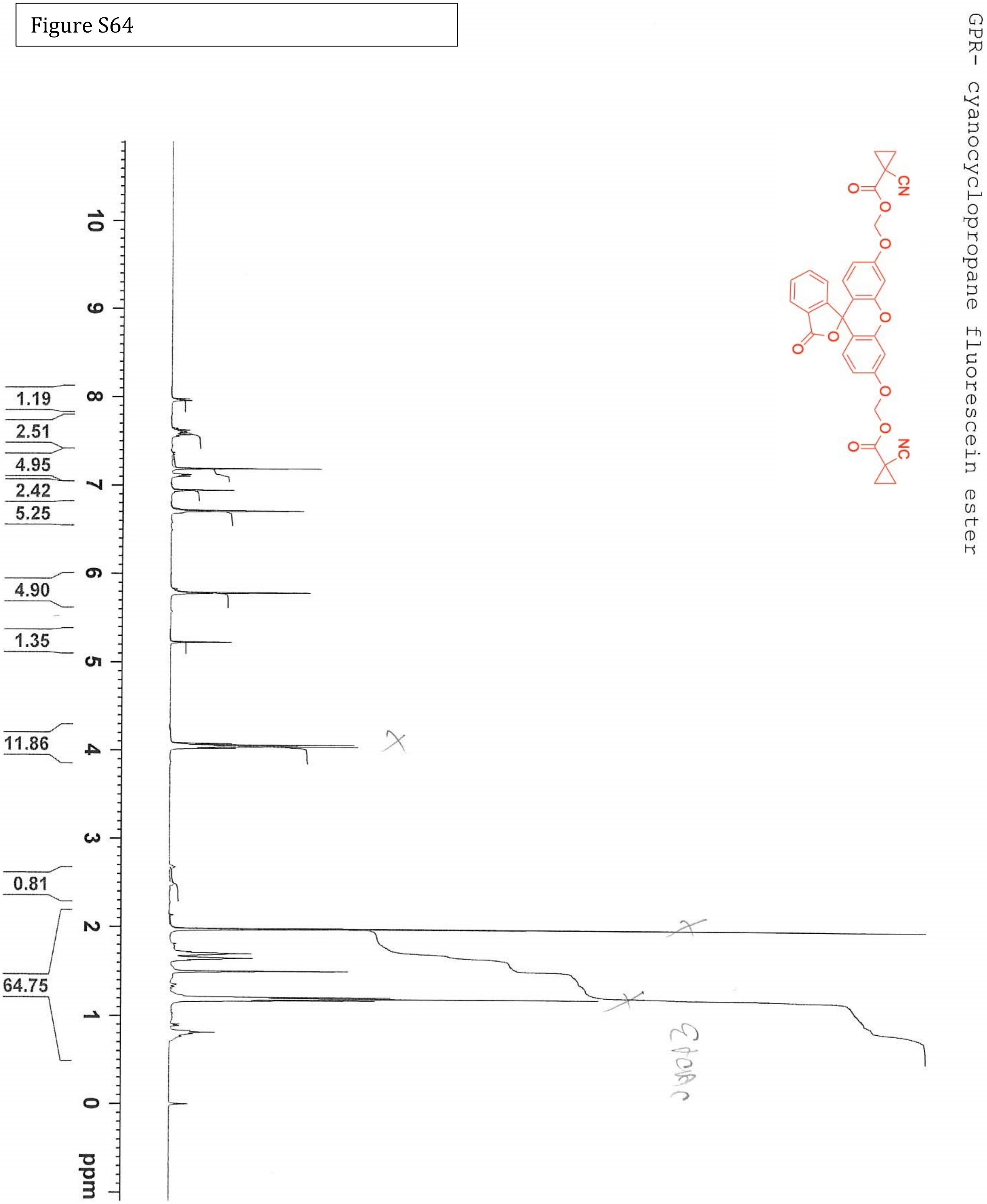

GPR- cyanocyclopropane fluorescein ester
CN
NC
O
1.19
2.51
4.95
2.42
5.25
4.90
1.35
11.86
0.81
64.75
10
9
8
7
6
5
4
3
2
1
0
ppm

Figure S65

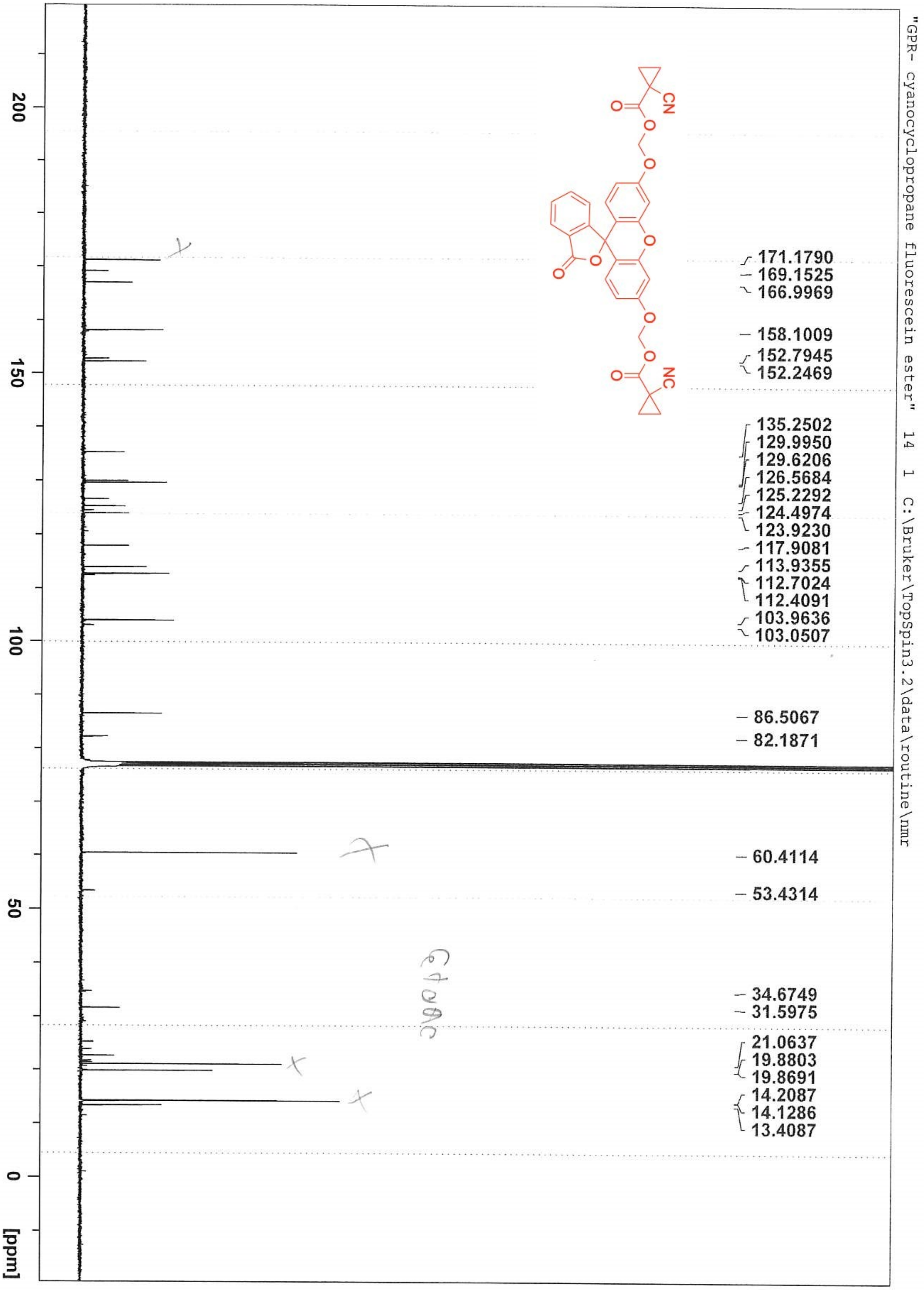

"GPR- cyanocyclopropane fluorescein ester" 14 1 C:\Bruker\TopSpin3.2\data\routine\nmr
CN
NC
171.1790
169.1525
166.9969
158.1009
152.7945
152.2469
135.2502
129.9950
129.6206
126.5684
125.2292
124.4974
123.9230
117.9081
113.9355
112.7024
112.4091
103.9636
103.0507
86.5067
82.1871
60.4114
53.4314
34.6749
31.5975
21.0637
19.8803
19.8691
14.2087
14.1286
13.4087
EtOAc
200
150
100
50
0
[ppm]

Figure S66

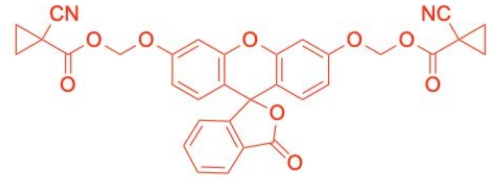
CN
NC

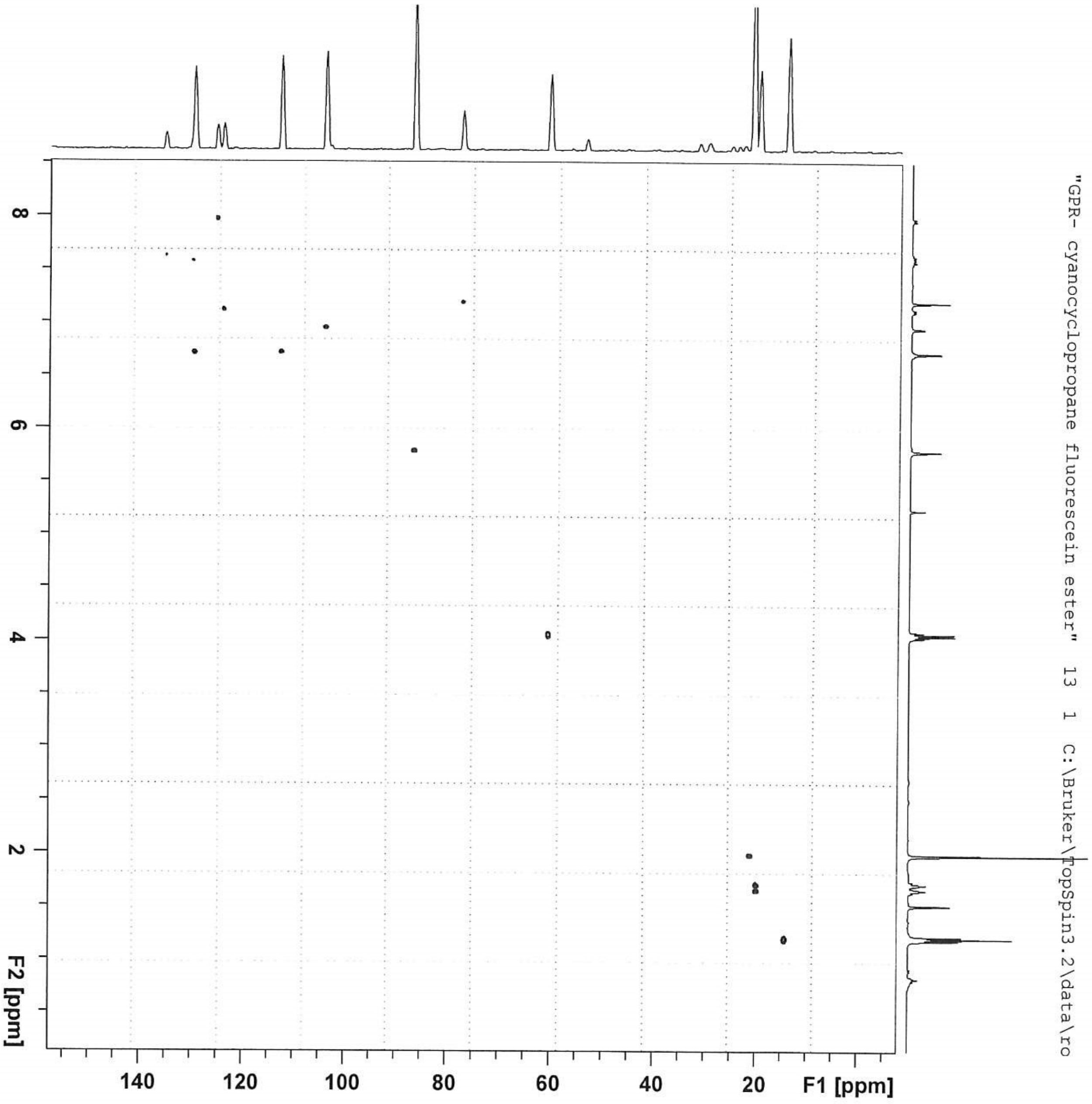
"GPR- cyanocyclopropane fluorescein ester" 13 1 C:\Bruker\TopSpin3.2\data\ro
F2 [ppm]
F1 [ppm]

Figure S67

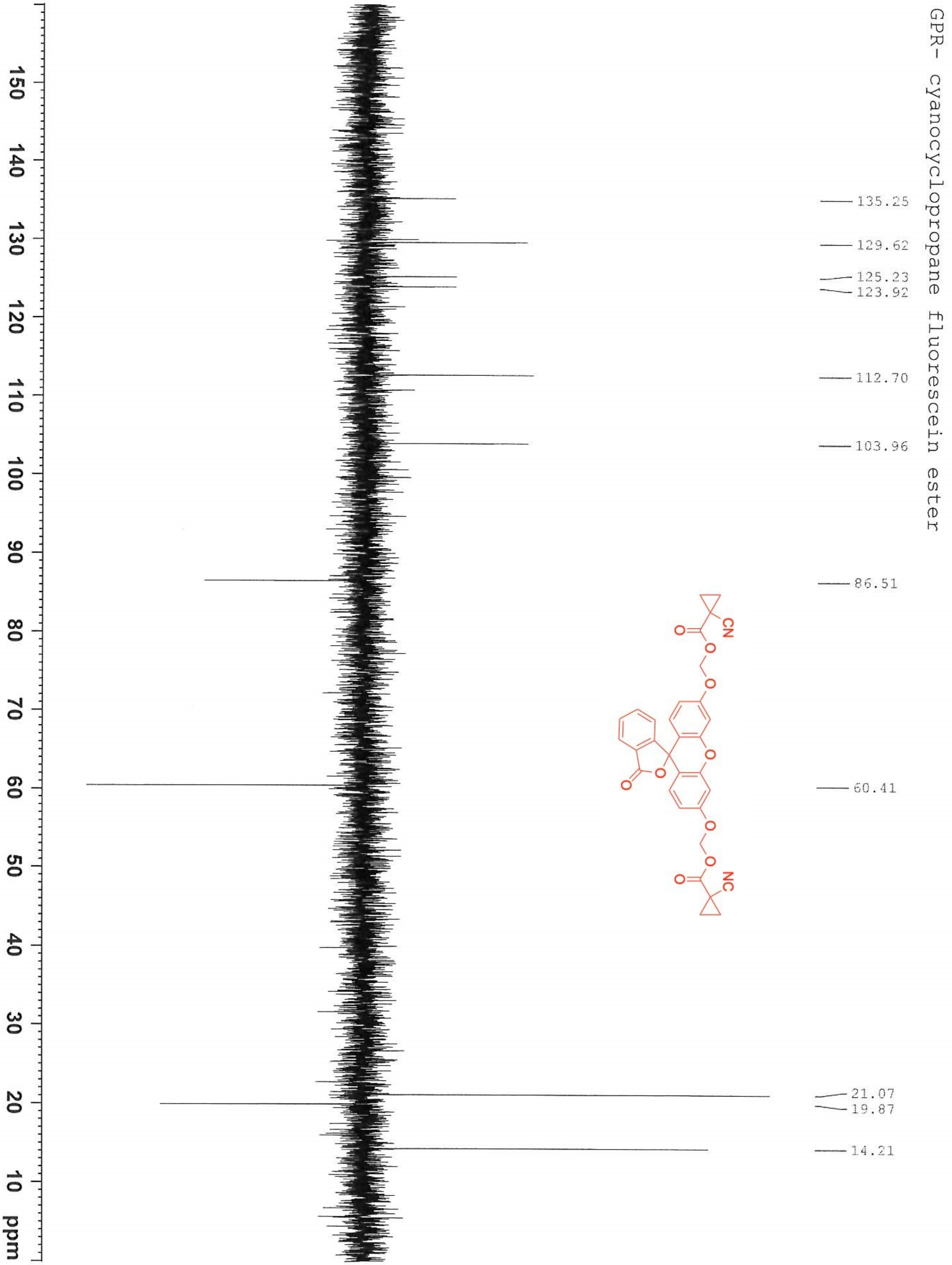
GPR- cyanocyclopropane fluorescein ester
135.25
129.62
125.23
123.92
112.70
103.96
86.51
60.41
21.07
19.87
14.21
CN
NC
ppm

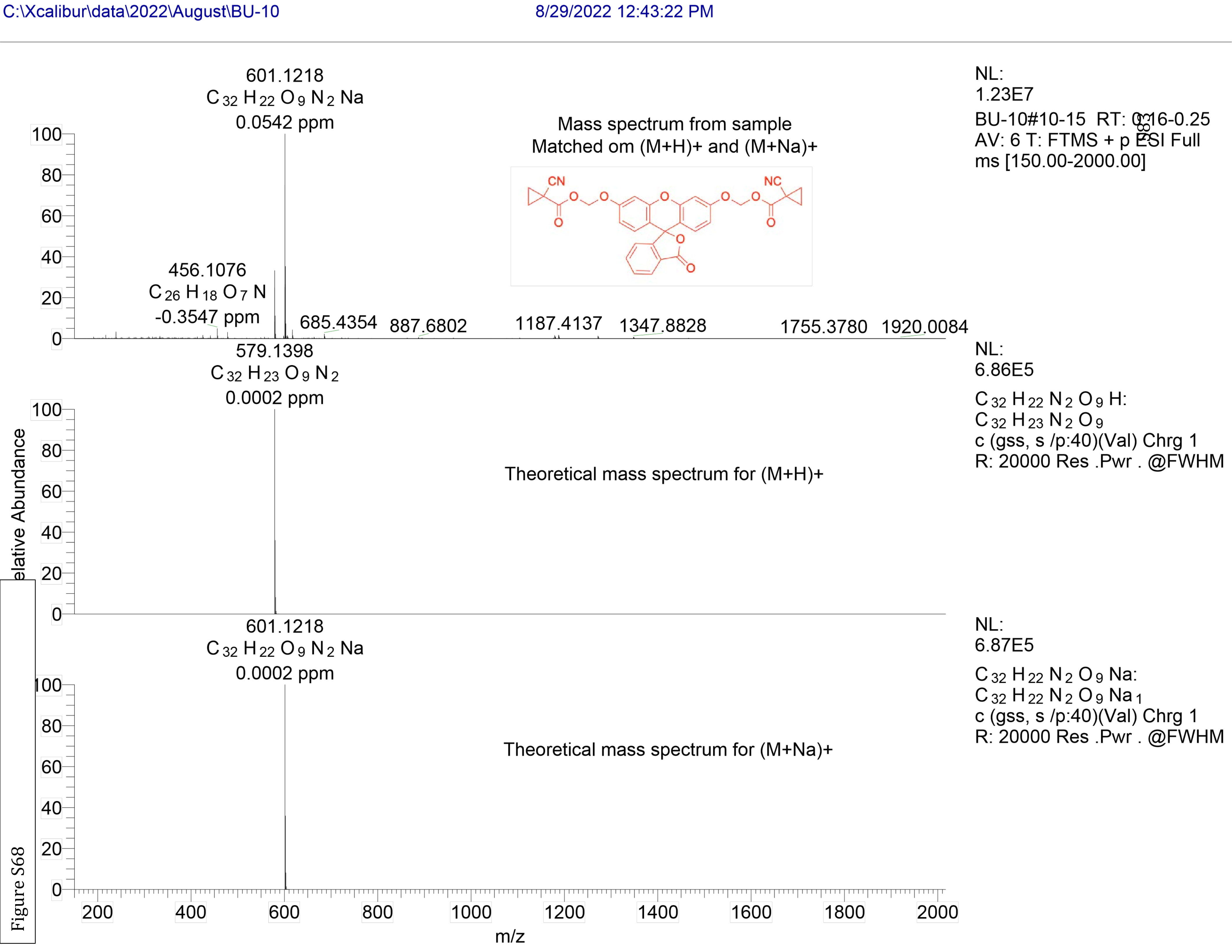

C:\Xcalibur\data\2022\August\BU-10
8/29/2022 12:43:22 PM
601.1218
C 32 H 22 O 9 N 2 Na
0.0542 ppm
Mass spectrum from sample
Matched om (M+H)+ and (M+Na)+
CN
NC
O
456.1076
C 26 H 18 O 7 N
-0.3547 ppm
685.4354
887.6802
1187.4137
1347.8828
1755.3780
1920.0084
NL:
1.23E7
BU-10#10-15 RT: 0.16-0.25
AV: 6 T: FTMS + p ESI Full
ms [150.00-2000.00]
579.1398
C 32 H 23 O 9 N 2
0.0002 ppm
Theoretical mass spectrum for (M+H)+
NL:
6.86E5
C 32 H 22 N 2 O 9 H:
C 32 H 23 N 2 O 9
c (gss, s /p:40)(Val) Chrg 1
R: 20000 Res .Pwr . @FWHM
601.1218
C 32 H 22 O 9 N 2 Na
0.0002 ppm
Theoretical mass spectrum for (M+Na)+
NL:
6.87E5
C 32 H 22 N 2 O 9 Na:
C 32 H 22 N 2 O 9 Na 1
c (gss, s /p:40)(Val) Chrg 1
R: 20000 Res .Pwr . @FWHM
Relative Abundance
m/z

Figure S68

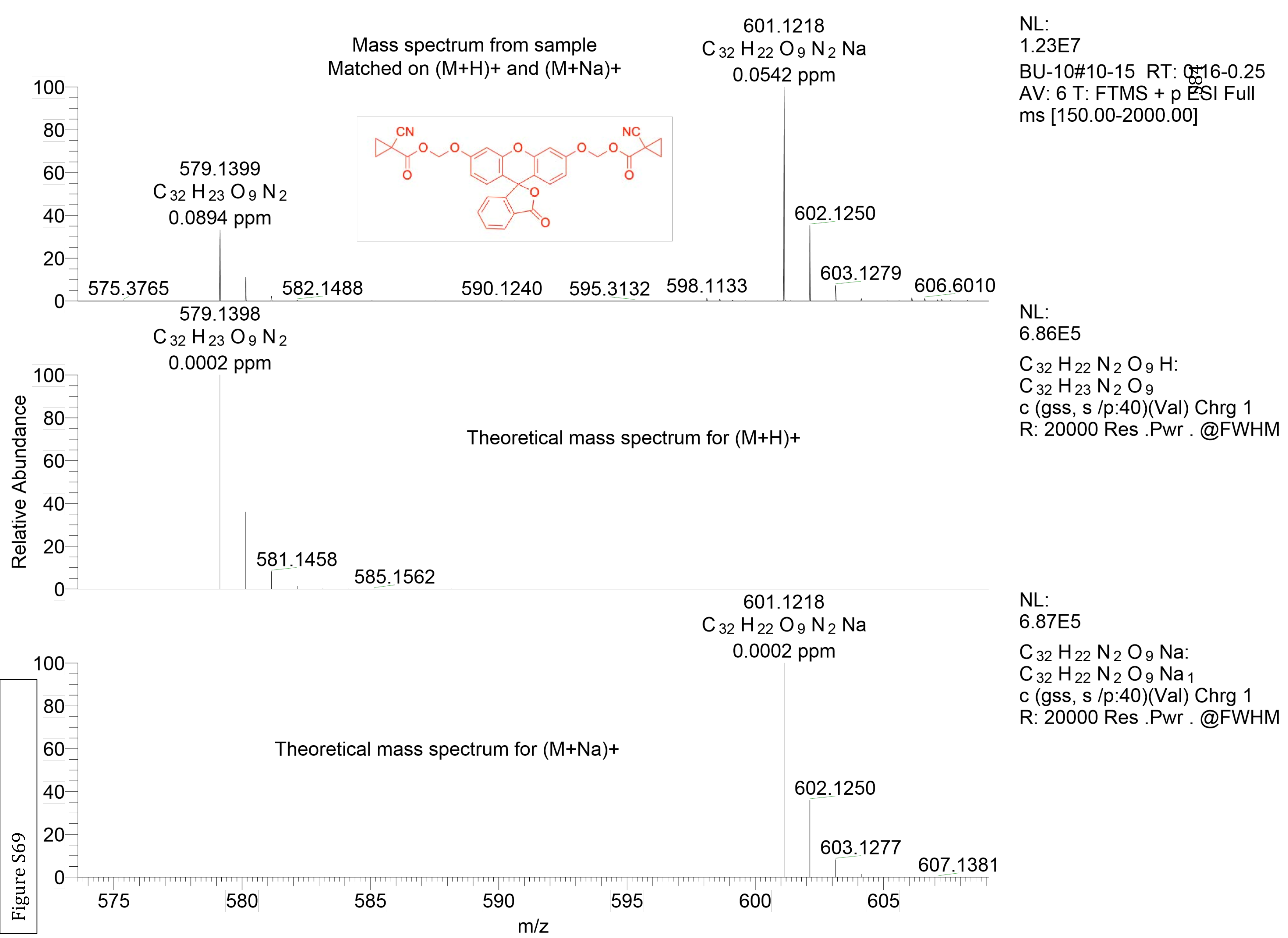
Mass spectrum from sample
Matched on (M+H)+ and (M+Na)+
601.1218
C 32 H 22 O 9 N 2 Na
0.0542 ppm
579.1399
C 32 H 23 O 9 N 2
0.0894 ppm
575.3765
582.1488
590.1240
595.3132
598.1133
602.1250
603.1279
606.6010
NL:
1.23E7
BU-10#10-15 RT: 0.16-0.25
AV: 6 T: FTMS + p ESI Full
ms [150.00-2000.00]
579.1398
C 32 H 23 O 9 N 2
0.0002 ppm
Theoretical mass spectrum for (M+H)+
581.1458
585.1562
NL:
6.86E5
C 32 H 22 N 2 O 9 H:
C 32 H 23 N 2 O 9
c (gss, s /p:40)(Val) Chrg 1
R: 20000 Res .Pwr . @FWHM
601.1218
C 32 H 22 O 9 N 2 Na
0.0002 ppm
Theoretical mass spectrum for (M+Na)+
602.1250
603.1277
607.1381
NL:
6.87E5
C 32 H 22 N 2 O 9 Na:
C 32 H 22 N 2 O 9 Na 1
c (gss, s /p:40)(Val) Chrg 1
R: 20000 Res .Pwr . @FWHM
Relative Abundance
m/z
CN
NC
O

Figure S69

Figure S70

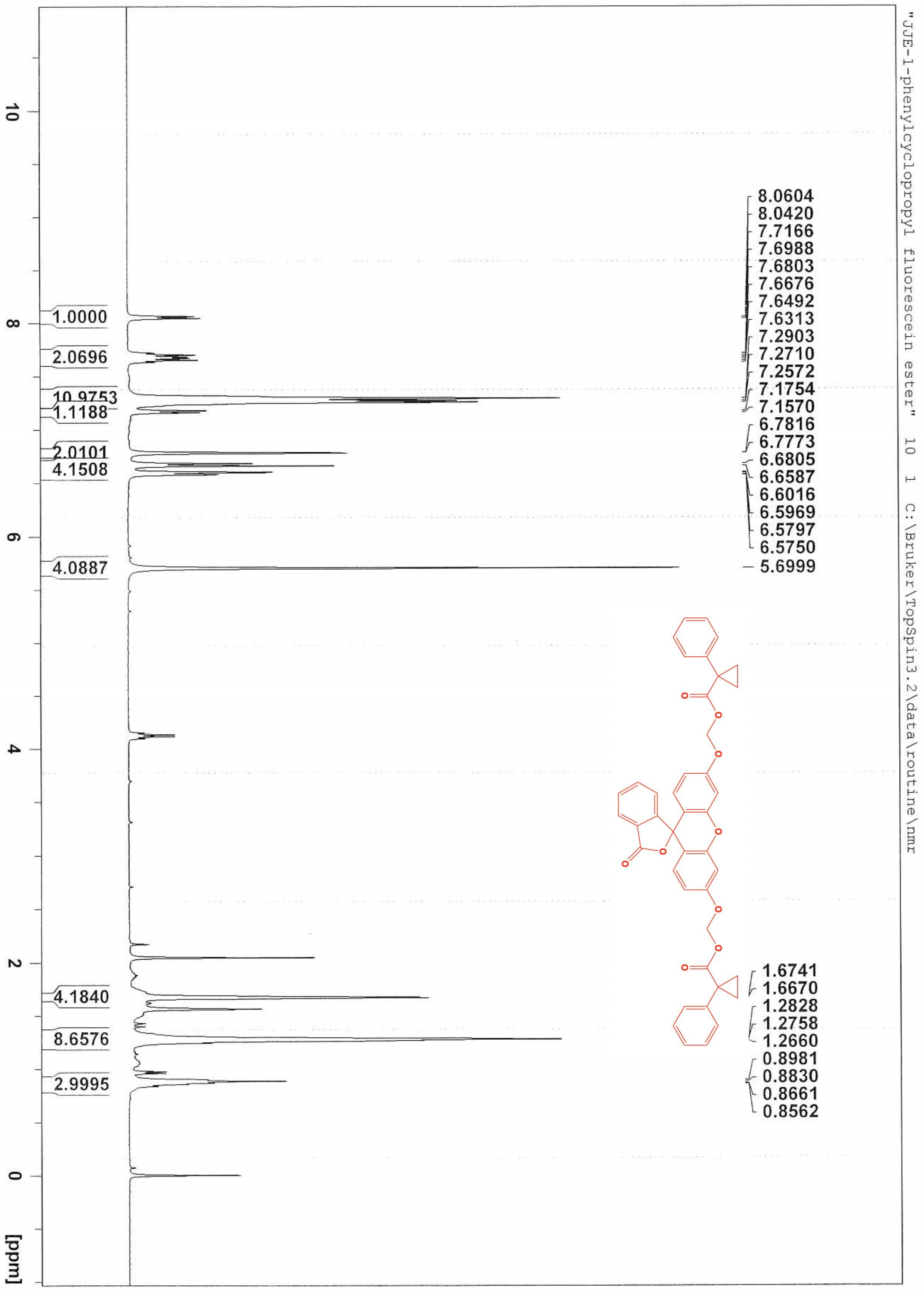
"JJE-1-phenylcyclopropyl fluorescein ester" 10 1 C:\Bruker\TopSpin3.2\data\routine\nmr
8.0604
8.0420
7.7166
7.6988
7.6803
7.6676
7.6492
7.6313
7.2903
7.2710
7.2572
7.1754
7.1570
6.7816
6.7773
6.6805
6.6587
6.6016
6.5969
6.5797
6.5750
5.6999
1.6741
1.6670
1.2828
1.2758
1.2660
0.8981
0.8830
0.8661
0.8562
1.0000
2.0696
10.9753
1.1188
2.0101
4.1508
4.0887
4.1840
8.6576
2.9995
10
8
6
4
2
0
[ppm]

Figure S71

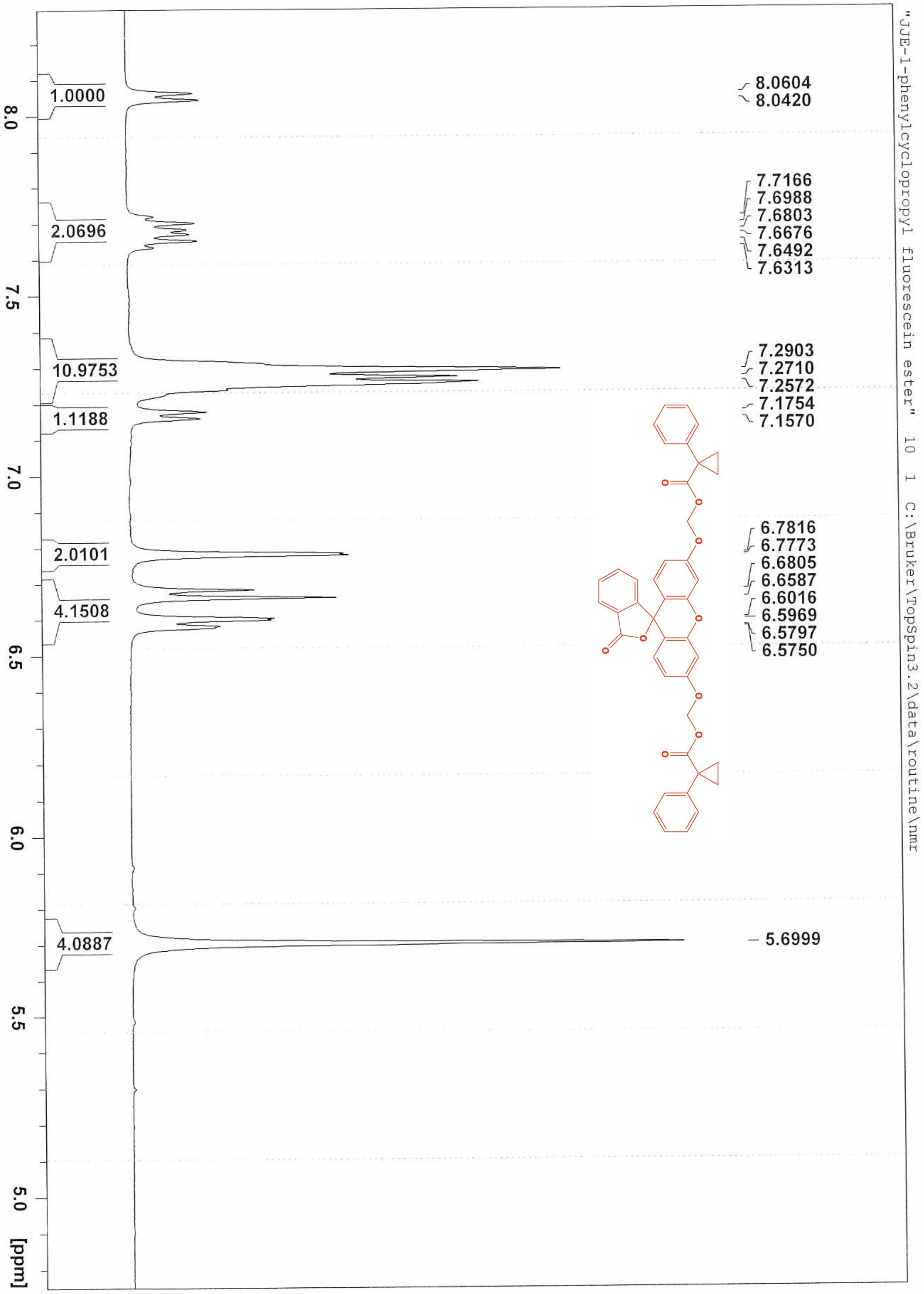
"JJE-1-phenylcyclopropyl fluorescein ester" 10 1 C:\Bruker\TopSpin3.2\data\routine\nmr
8.0604
8.0420
7.7166
7.6988
7.6803
7.6676
7.6492
7.6313
7.2903
7.2710
7.2572
7.1754
7.1570
6.7816
6.7773
6.6805
6.6587
6.6016
6.5969
6.5797
6.5750
5.6999
1.0000
2.0696
10.9753
1.1188
2.0101
4.1508
4.0887
8.0
7.5
7.0
6.5
6.0
5.5
5.0
[ppm]

Figure S72

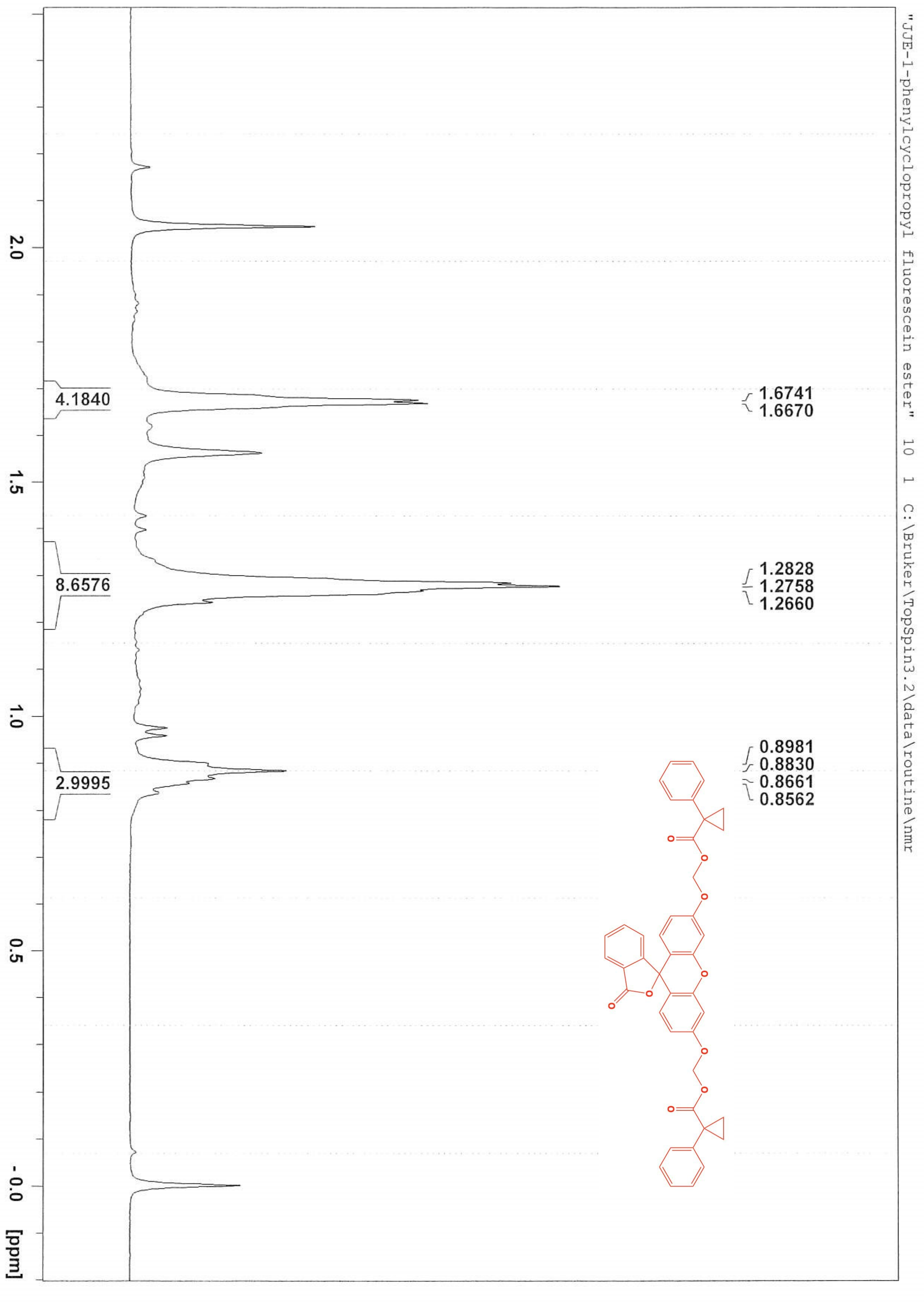

"JJE-1-phenylcyclopropyl fluorescein ester" 10 1 C:\Bruker\TopSpin3.2\data\routine\nmr
1.6741
1.6670
1.2828
1.2758
1.2660
0.8981
0.8830
0.8661
0.8562
4.1840
8.6576
2.9995
2.0
1.5
1.0
0.5
-0.0
[ppm]

Figure S73

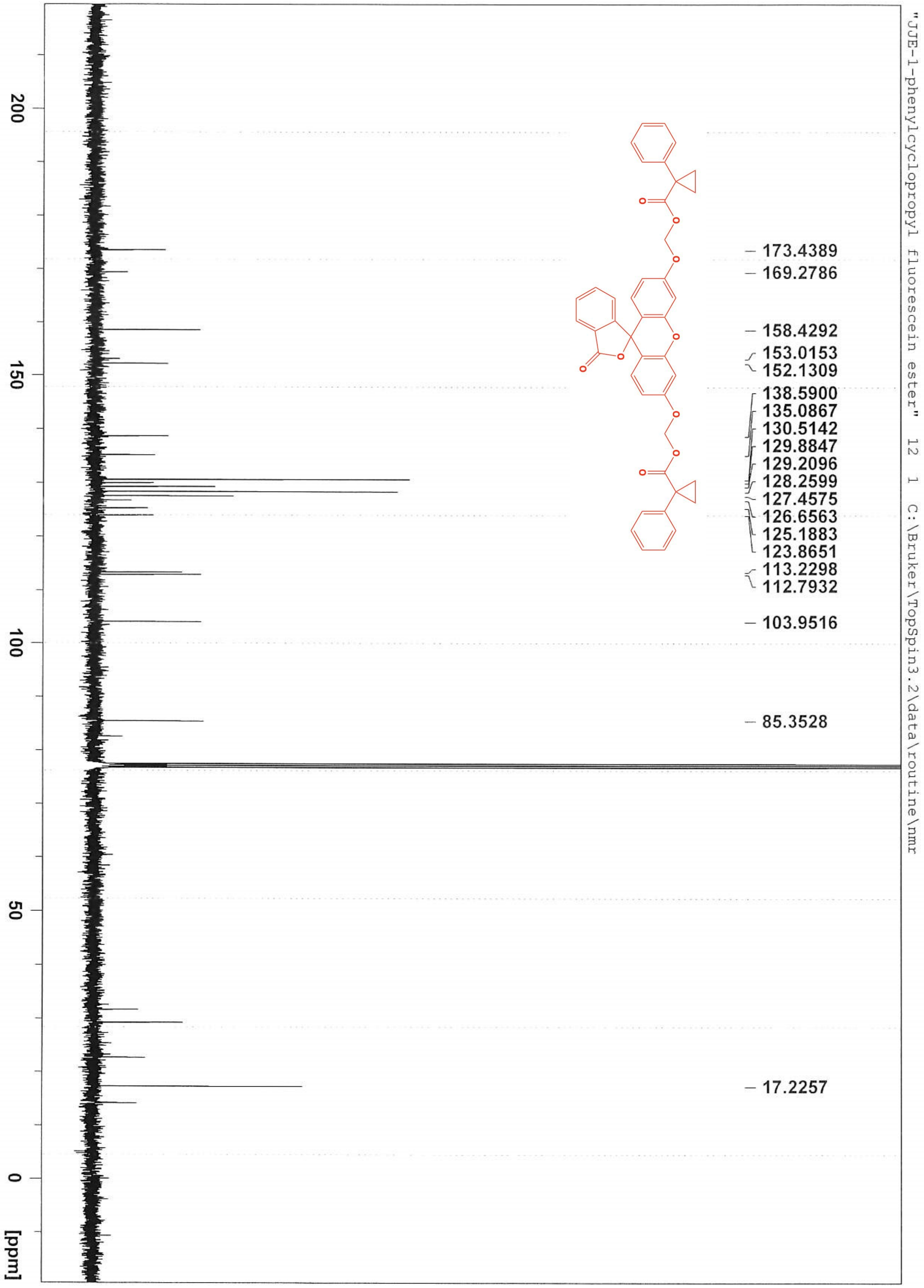
"JJE-1-phenylcyclopropyl fluorescein ester" 12 1 C:\Bruker\TopSpin3.2\data\routine\nmr
173.4389
169.2786
158.4292
153.0153
152.1309
138.5900
135.0867
130.5142
129.8847
129.2096
128.2599
127.4575
126.6563
125.1883
123.8651
113.2298
112.7932
103.9516
85.3528
17.2257
200
150
100
50
0
[ppm]

Figure S74

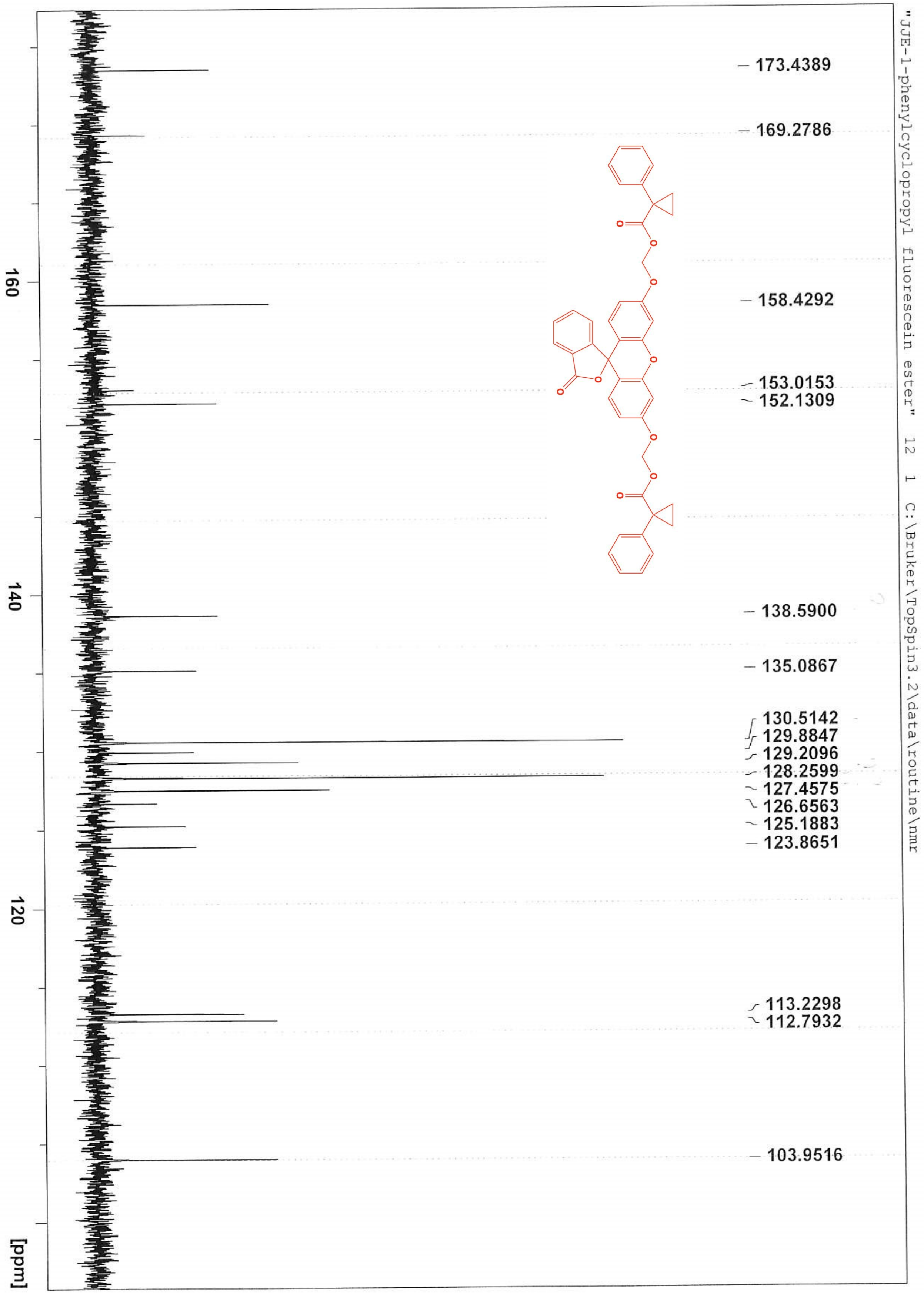
"JJE-1-phenylcyclopropyl fluorescein ester" 12 1 C:\Bruker\TopSpin3.2\data\routine\nmr
173.4389
169.2786
158.4292
153.0153
152.1309
138.5900
135.0867
130.5142
129.8847
129.2096
128.2599
127.4575
126.6563
125.1883
123.8651
113.2298
112.7932
103.9516
160
140
120
[ppm]

Figure S75

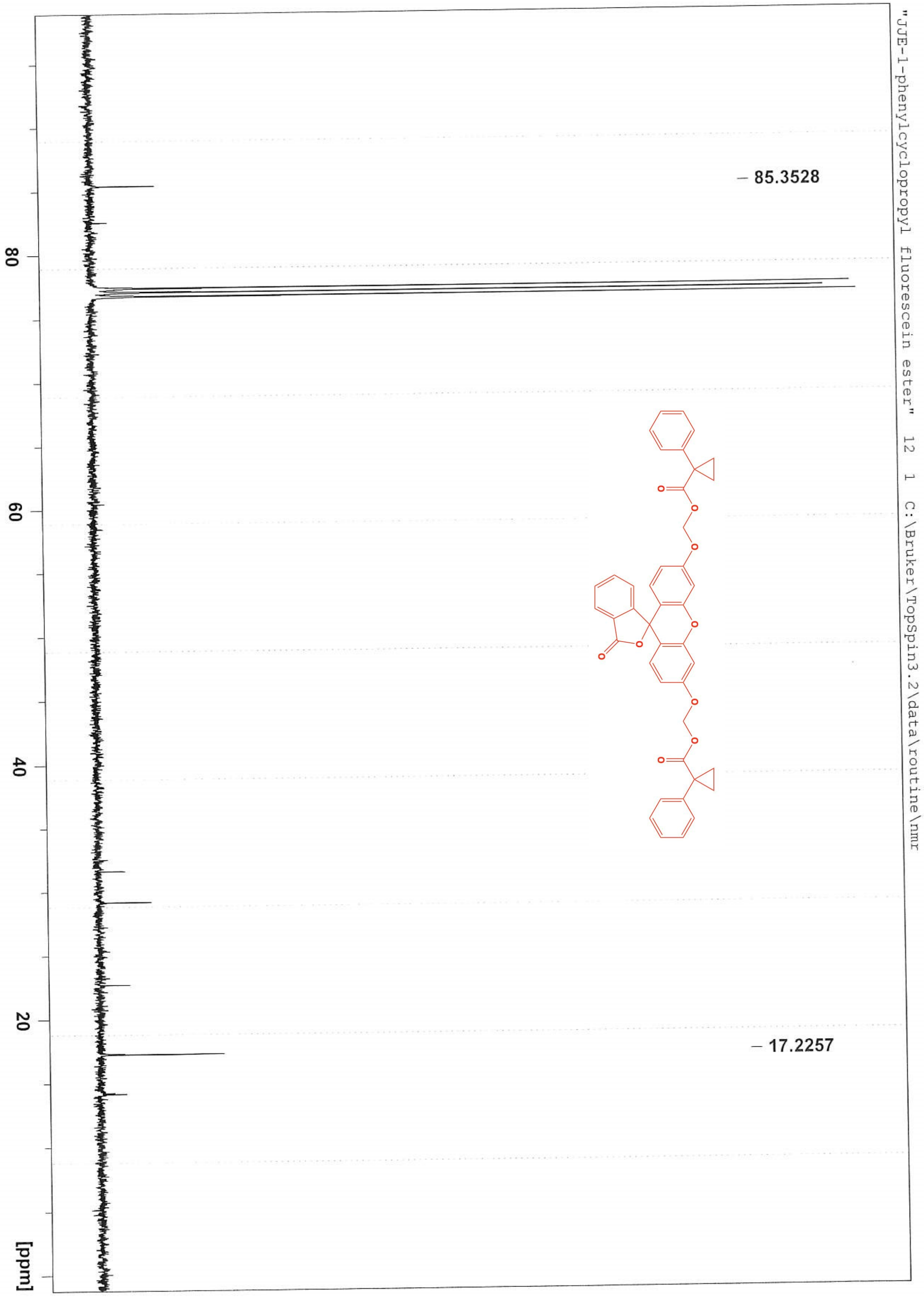

"JJE-1-phenylcyclopropyl fluorescein ester" 12 1 C:\Bruker\TopSpin3.2\data\routine\nmr
85.3528
17.2257
80
60
40
20
[ppm]

Figure S76

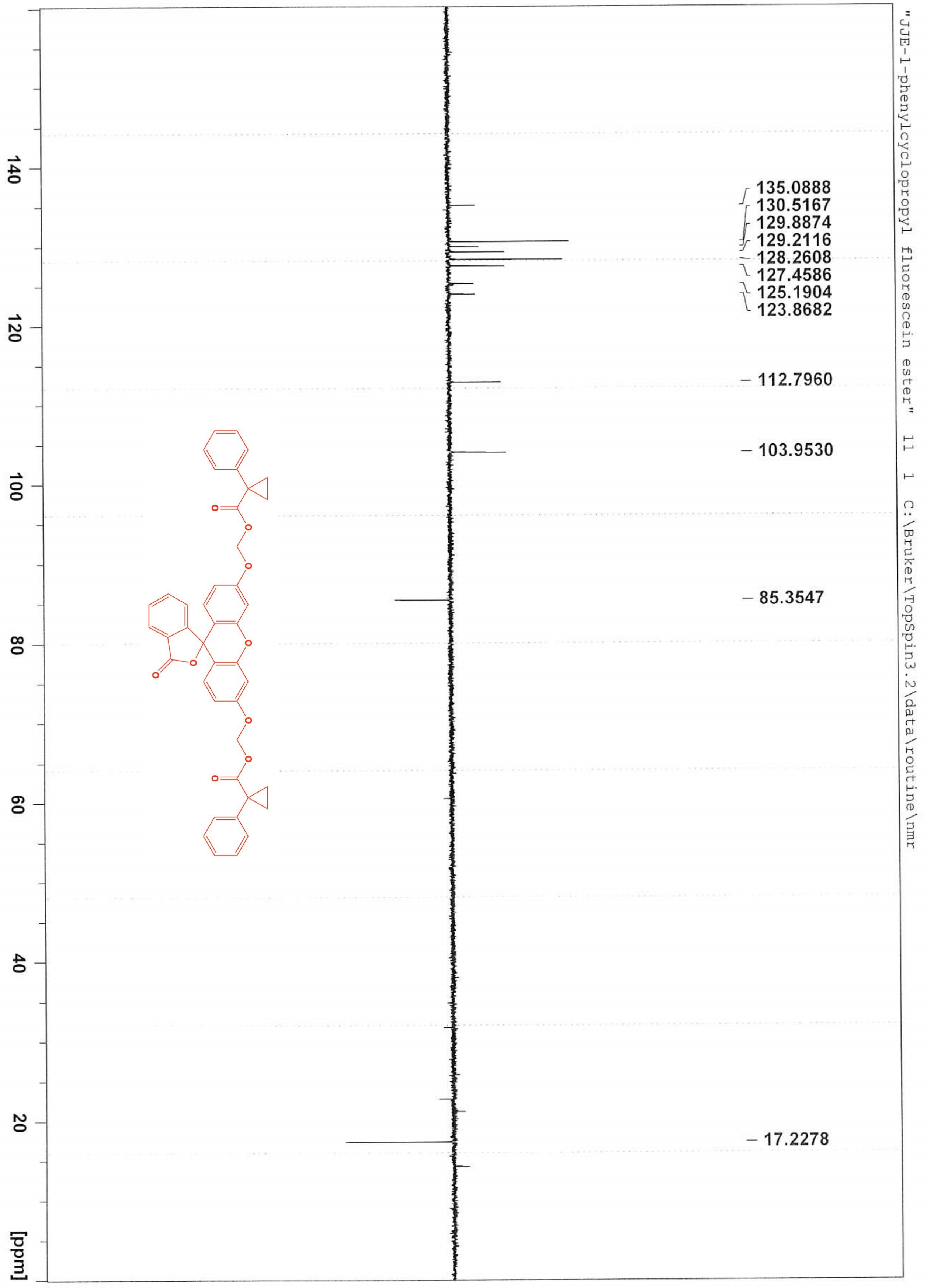
"JJE-1-phenylcyclopropyl fluorescein ester" 11 1 C:\Bruker\TopSpin3.2\data\routine\nmr
135.0888
130.5167
129.8874
129.2116
128.2608
127.4586
125.1904
123.8682
112.7960
103.9530
85.3547
17.2278
140
120
100
80
60
40
20
[ppm]

Figure S77

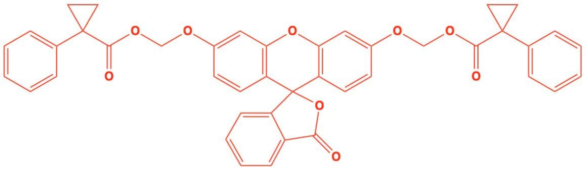

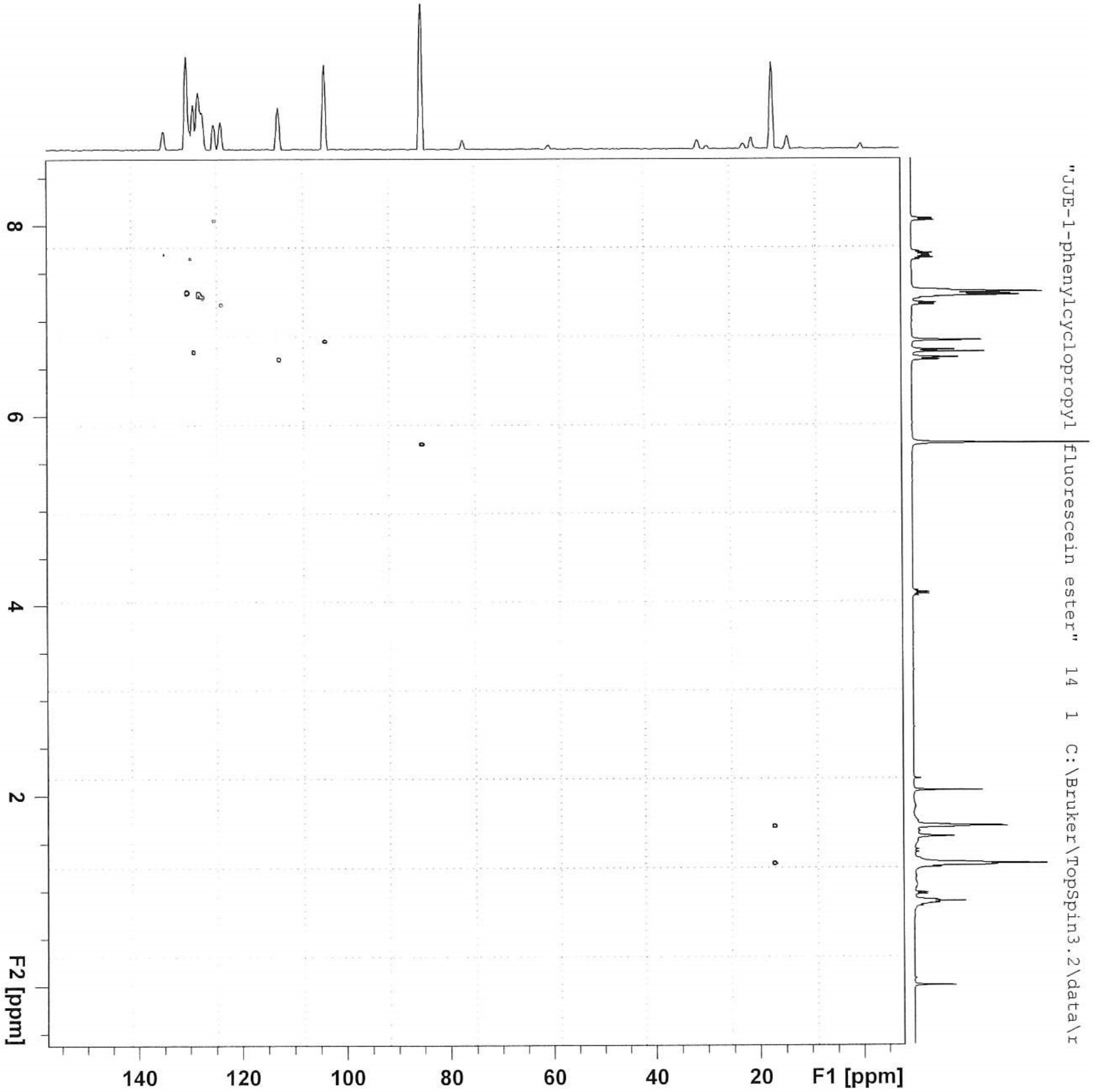

"JJE-1-phenylcyclopropyl fluorescein ester" 14 1 C:\Bruker\TopSpin3.2\data\r
F2 [ppm]
8
6
4
2
140
120
100
80
60
40
20
F1 [ppm]

Figure S78

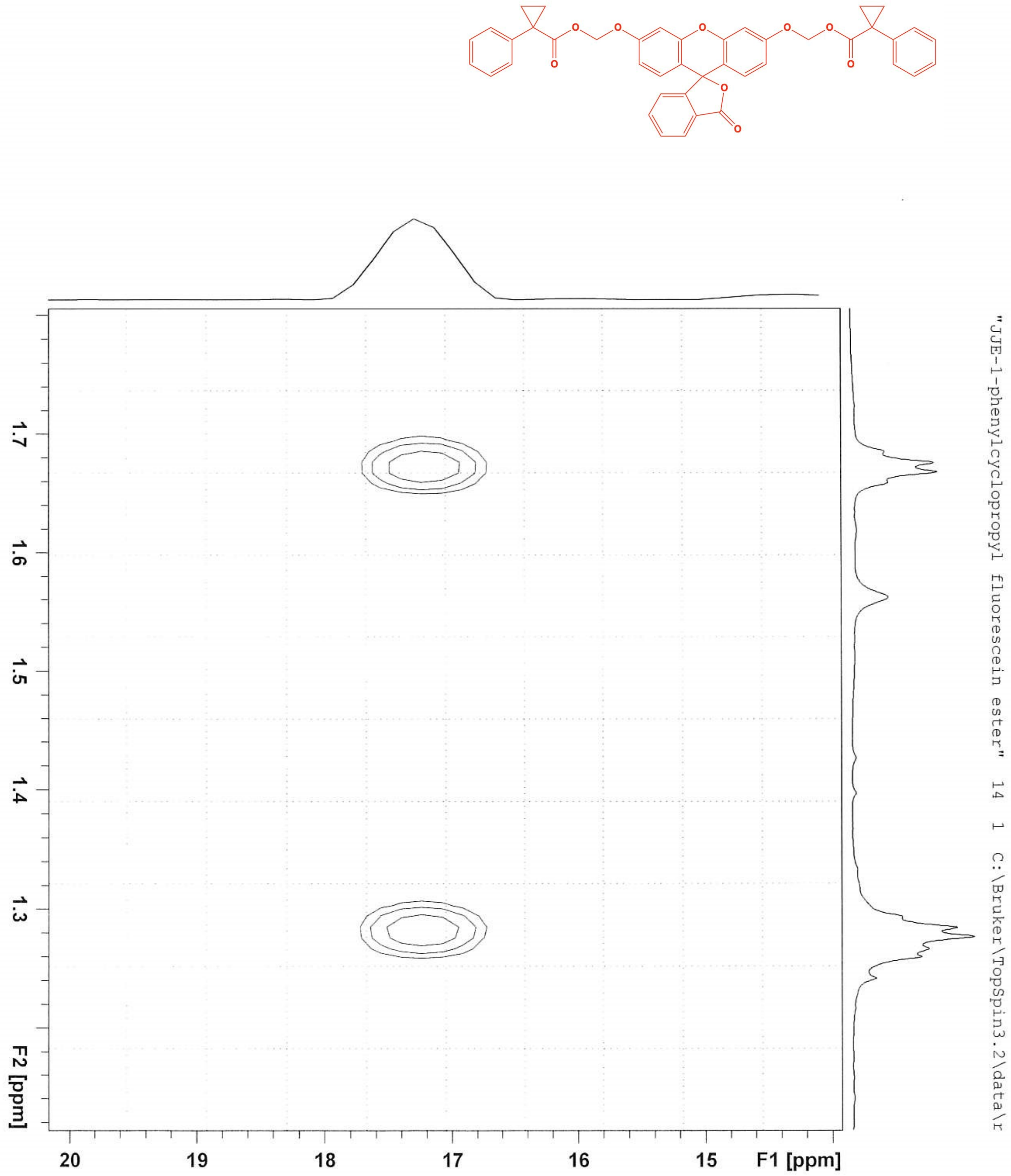

"JJE-1-phenylcyclopropyl fluorescein ester" 14 1 C:\Bruker\TopSpin3.2\data\r
1.7
1.6
1.5
1.4
1.3
F2 [ppm]
20
19
18
17
16
15
F1 [ppm]

Figure S79

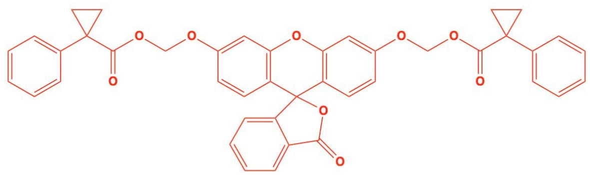

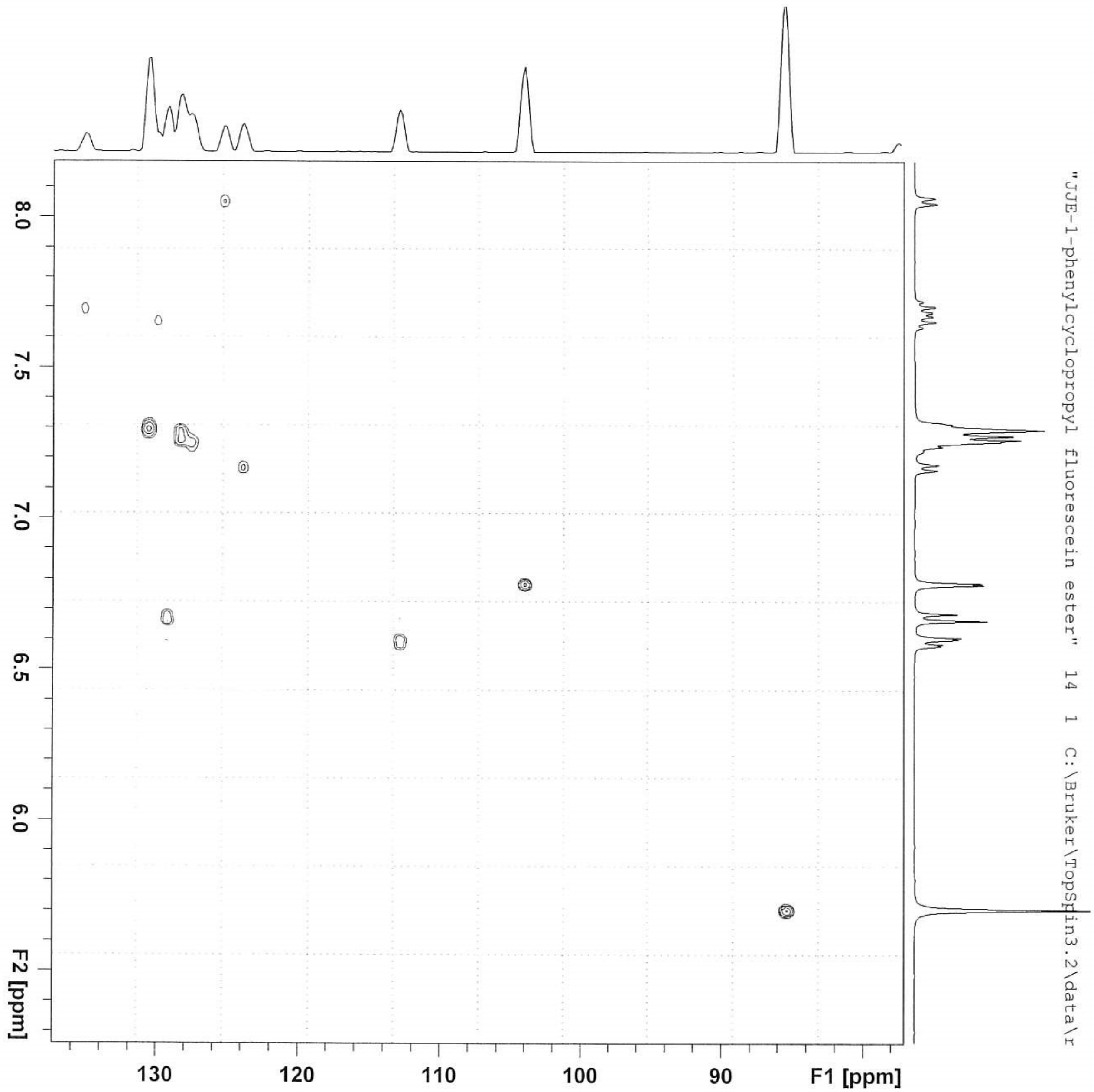
"JJE-1-phenylcyclopropyl fluorescein ester" 14 1 C:\Bruker\TopSpin3.2\data\r
8.0
7.5
7.0
6.5
6.0
F2 [ppm]
130
120
110
100
90
F1 [ppm]

Figure S80

1-phenylcyclopropane carboxylic acid

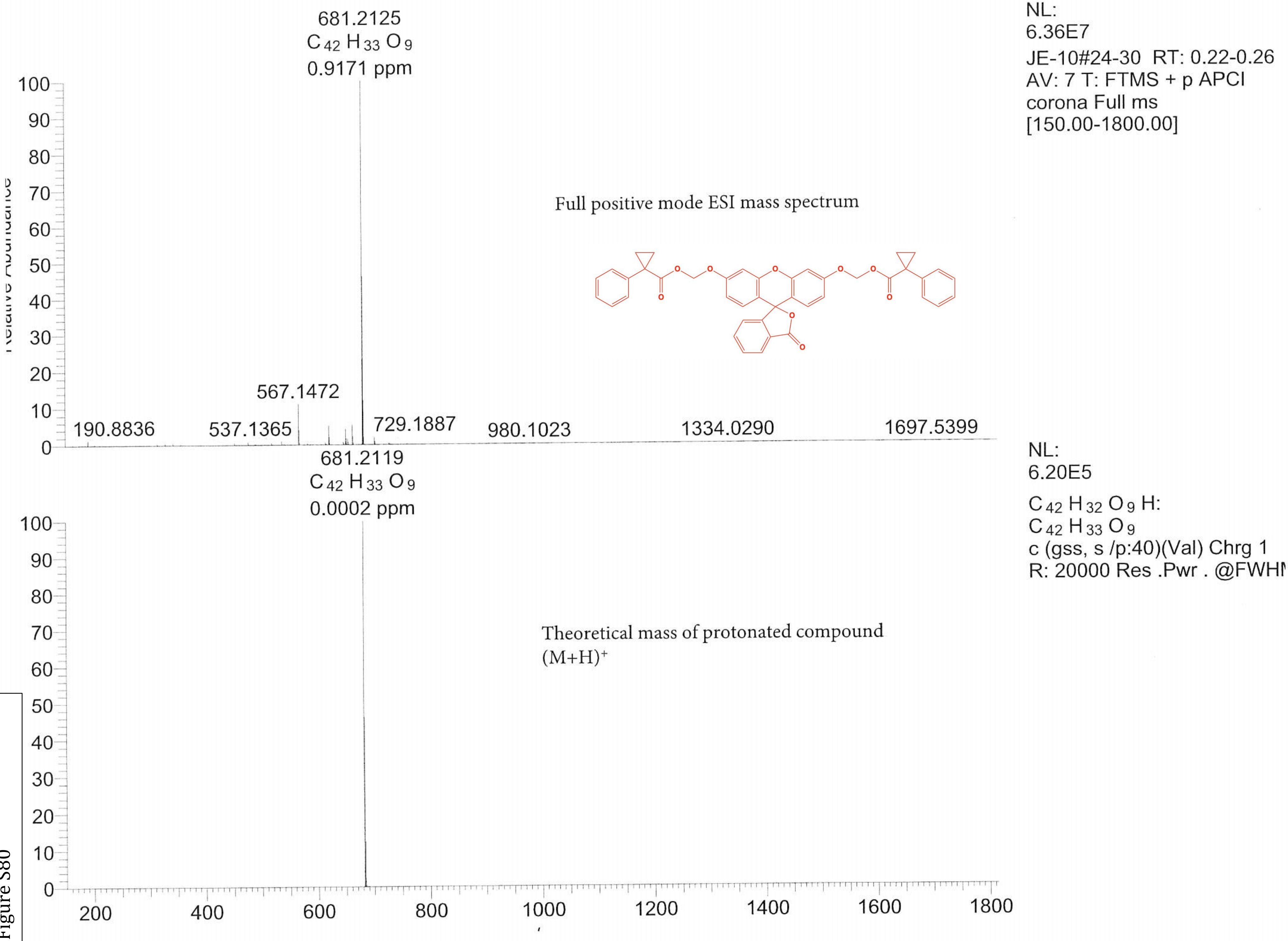

Figure S81

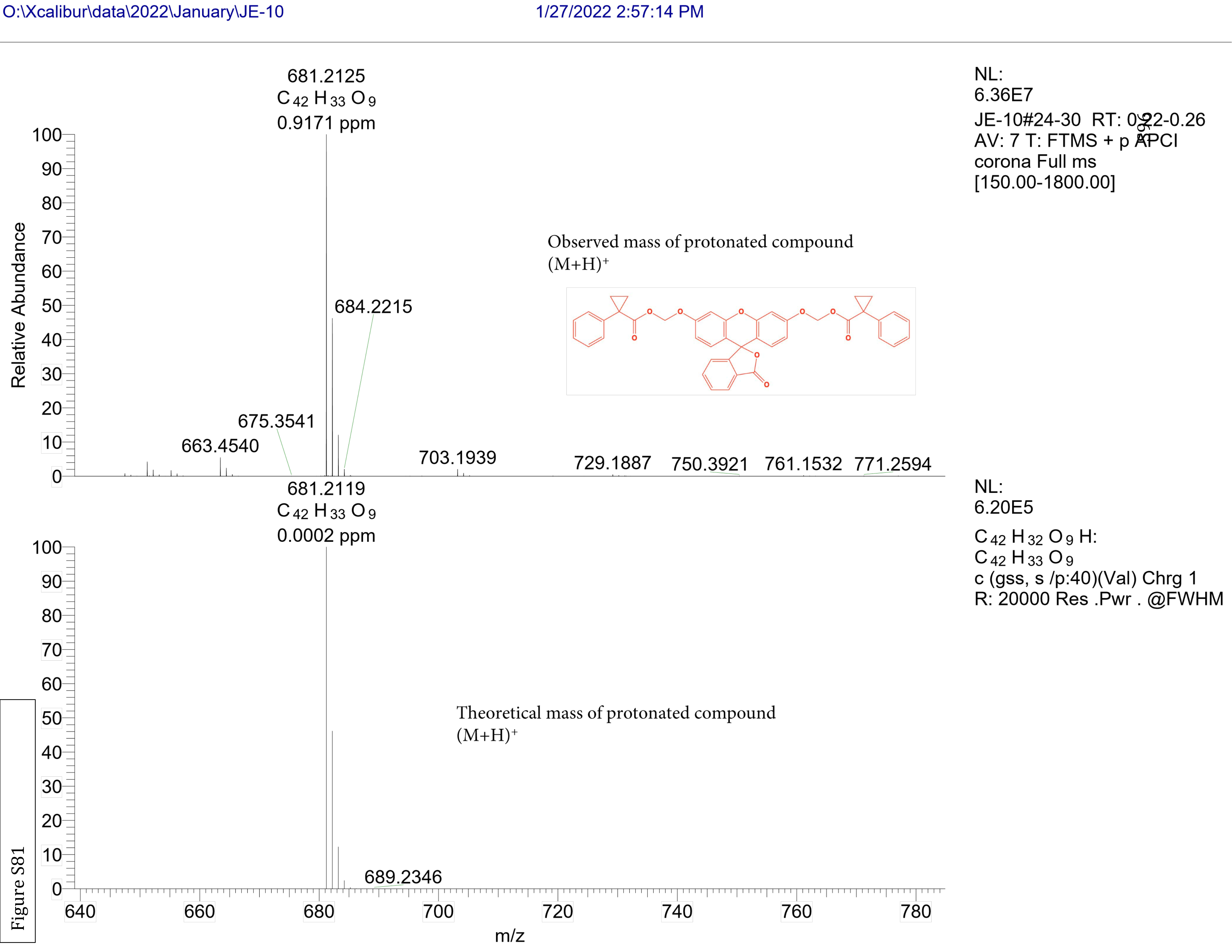

O:\Xcalibur\data\2022\January\JE-10
1/27/2022 2:57:14 PM
681.2125
C 42 H 33 O 9
0.9171 ppm
NL:
6.36E7
JE-10#24-30 RT: 0.22-0.26
AV: 7 T: FTMS + p APCI
corona Full ms
[150.00-1800.00]
Observed mass of protonated compound
(M+H)+
684.2215
675.3541
663.4540
703.1939
729.1887
750.3921
761.1532
771.2594
681.2119
C 42 H 33 O 9
0.0002 ppm
NL:
6.20E5
C 42 H 32 O 9 H:
C 42 H 33 O 9
c (gss, s /p:40)(Val) Chrg 1
R: 20000 Res .Pwr . @FWHM
Theoretical mass of protonated compound
(M+H)+
689.2346
Relative Abundance
m/z

Figure S82

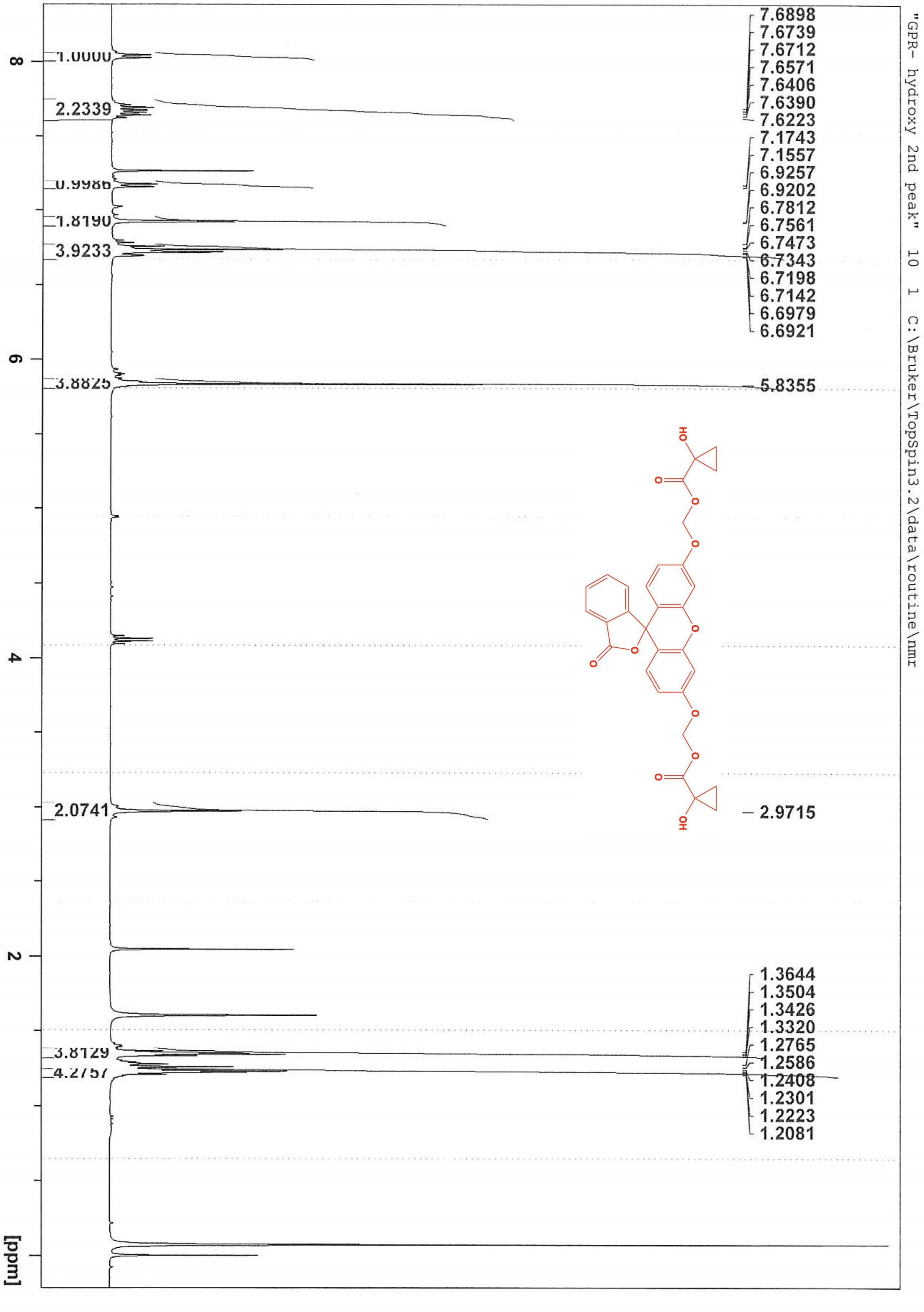

"GPR- hydroxy 2nd peak" 10 1 C:\Bruker\TopSpin3.2\data\routine\nmr
7.6898
7.6739
7.6712
7.6571
7.6406
7.6390
7.6223
7.1743
7.1557
6.9257
6.9202
6.7812
6.7561
6.7473
6.7343
6.7198
6.7142
6.6979
6.6921
5.8355
2.9715
1.3644
1.3504
1.3426
1.3320
1.2765
1.2586
1.2408
1.2301
1.2223
1.2081
1.0000
2.2339
0.9986
1.8190
3.9233
3.8825
2.0741
3.8129
4.2757
8
6
4
2
[ppm]
HO
O
OH

Figure S83

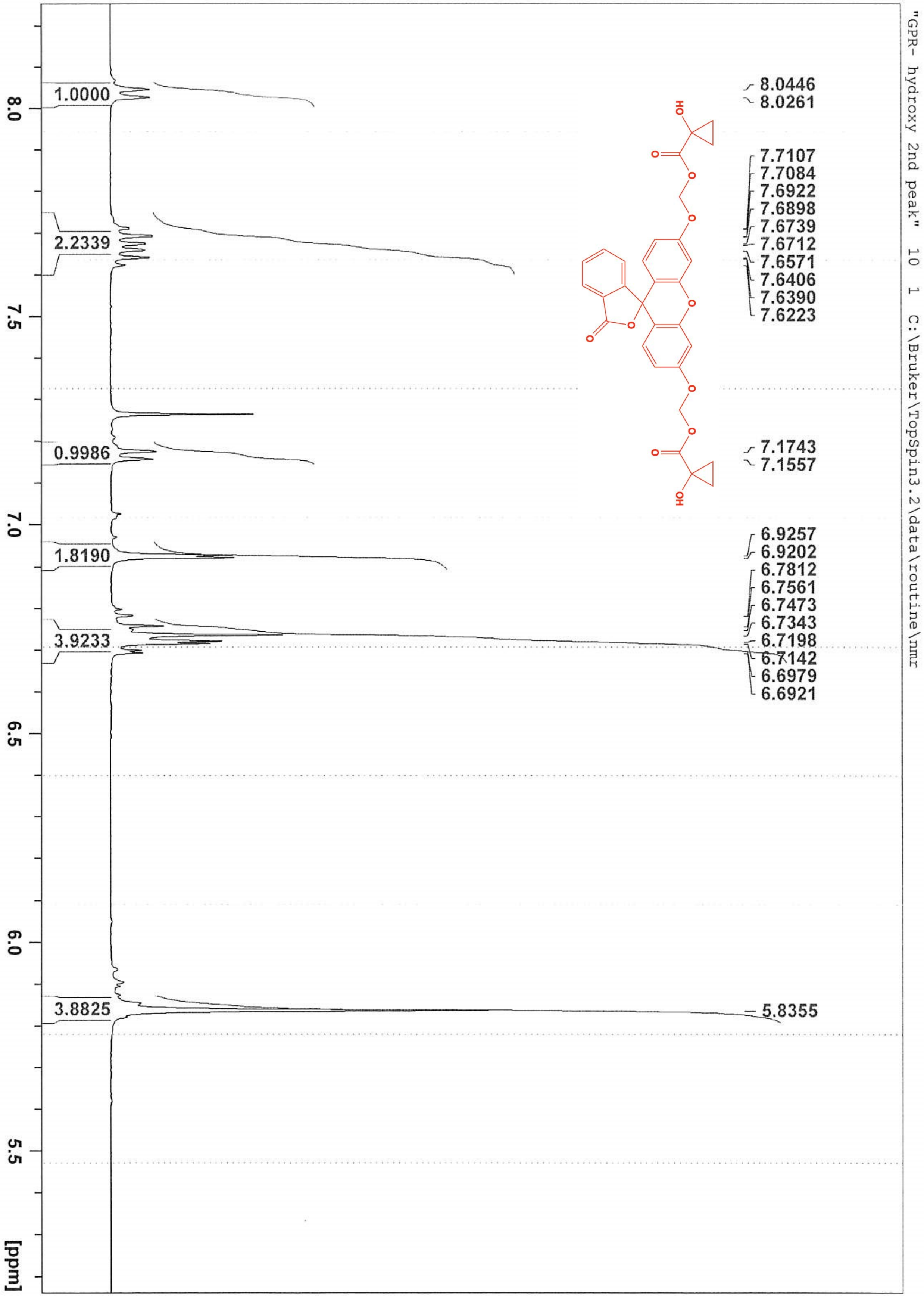

"GPR- hydroxy 2nd peak" 10 1 C:\Bruker\TopSpin3.2\data\routine\nmr
8.0446
8.0261
7.7107
7.7084
7.6922
7.6898
7.6739
7.6712
7.6571
7.6406
7.6390
7.6223
7.1743
7.1557
6.9257
6.9202
6.7812
6.7561
6.7473
6.7343
6.7198
6.7142
6.6979
6.6921
5.8355
1.0000
2.2339
0.9986
1.8190
3.9233
3.8825
8.0
7.5
7.0
6.5
6.0
5.5
[ppm]

Figure S84

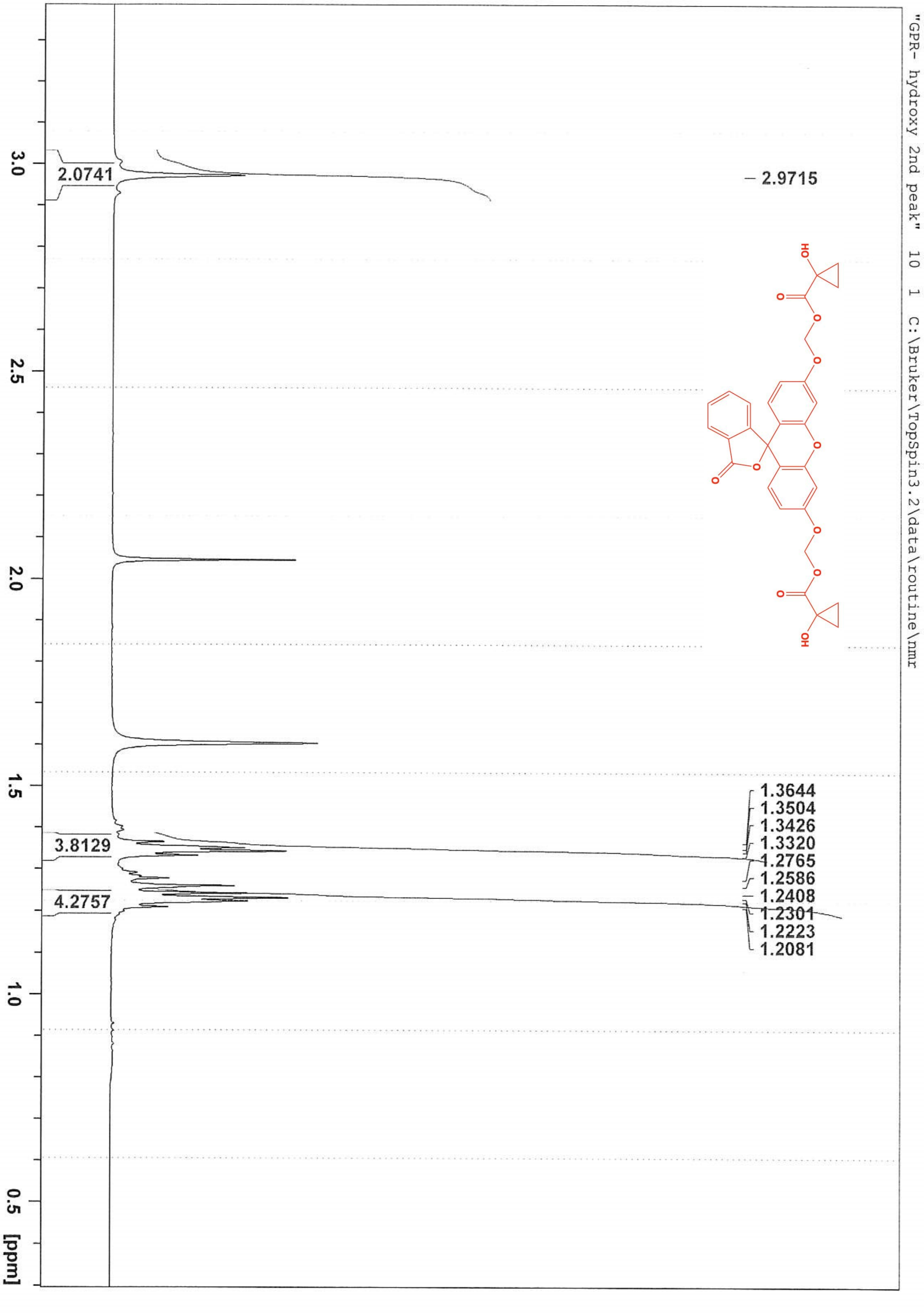
"GPR- hydroxy 2nd peak" 10 1 C:\Bruker\TopSpin3.2\data\routine\nmr
2.9715
1.3644
1.3504
1.3426
1.3320
1.2765
1.2586
1.2408
1.2301
1.2223
1.2081
2.0741
3.8129
4.2757
3.0
2.5
2.0
1.5
1.0
0.5
[ppm]

Figure S85

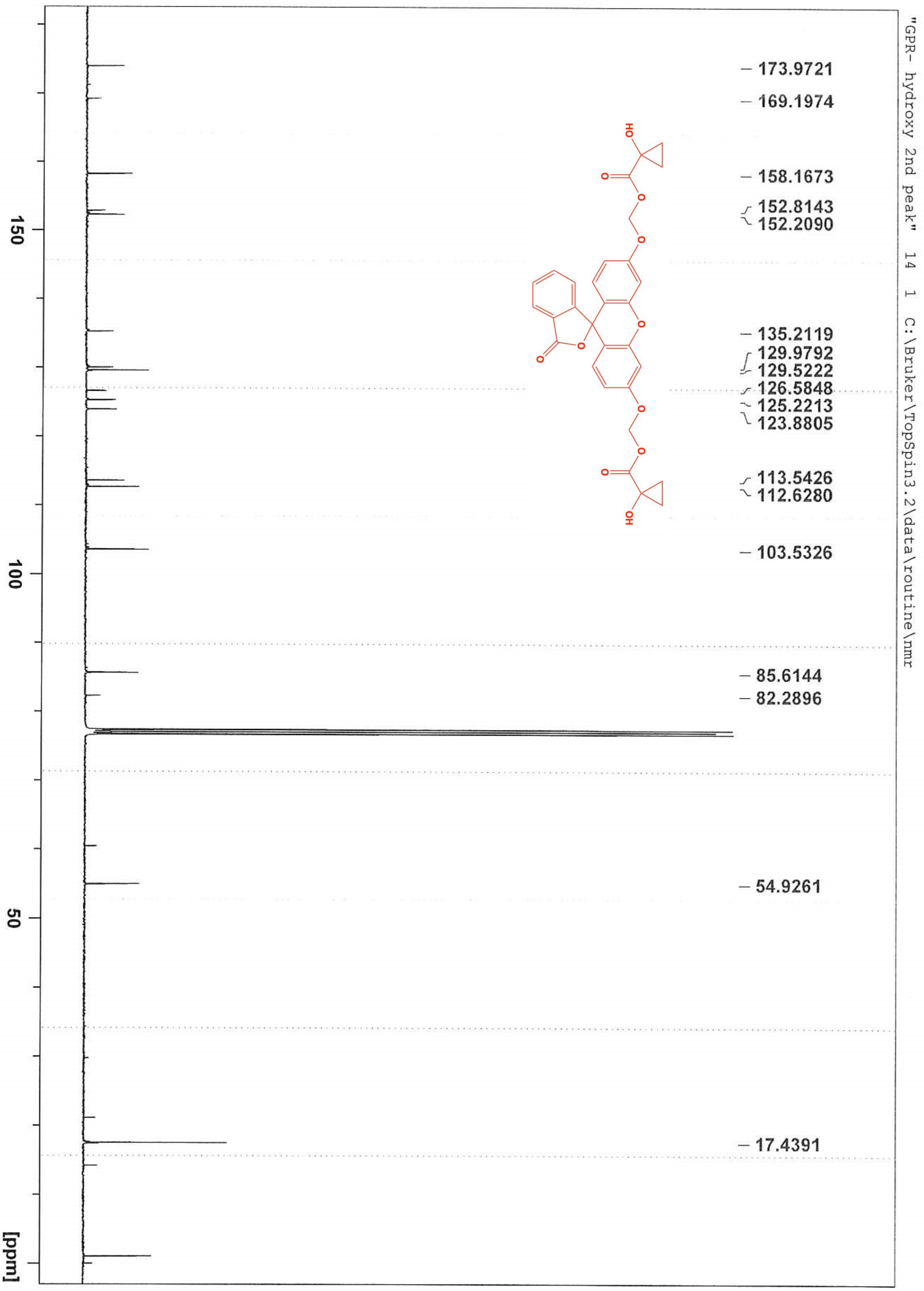
"GPR- hydroxy 2nd peak" 14 1 C:\Bruker\TopSpin3.2\data\routine\nmr
173.9721
169.1974
158.1673
152.8143
152.2090
135.2119
129.9792
129.5222
126.5848
125.2213
123.8805
113.5426
112.6280
103.5326
85.6144
82.2896
54.9261
17.4391
HO
O
OH
150
100
50
[ppm]

Figure S86

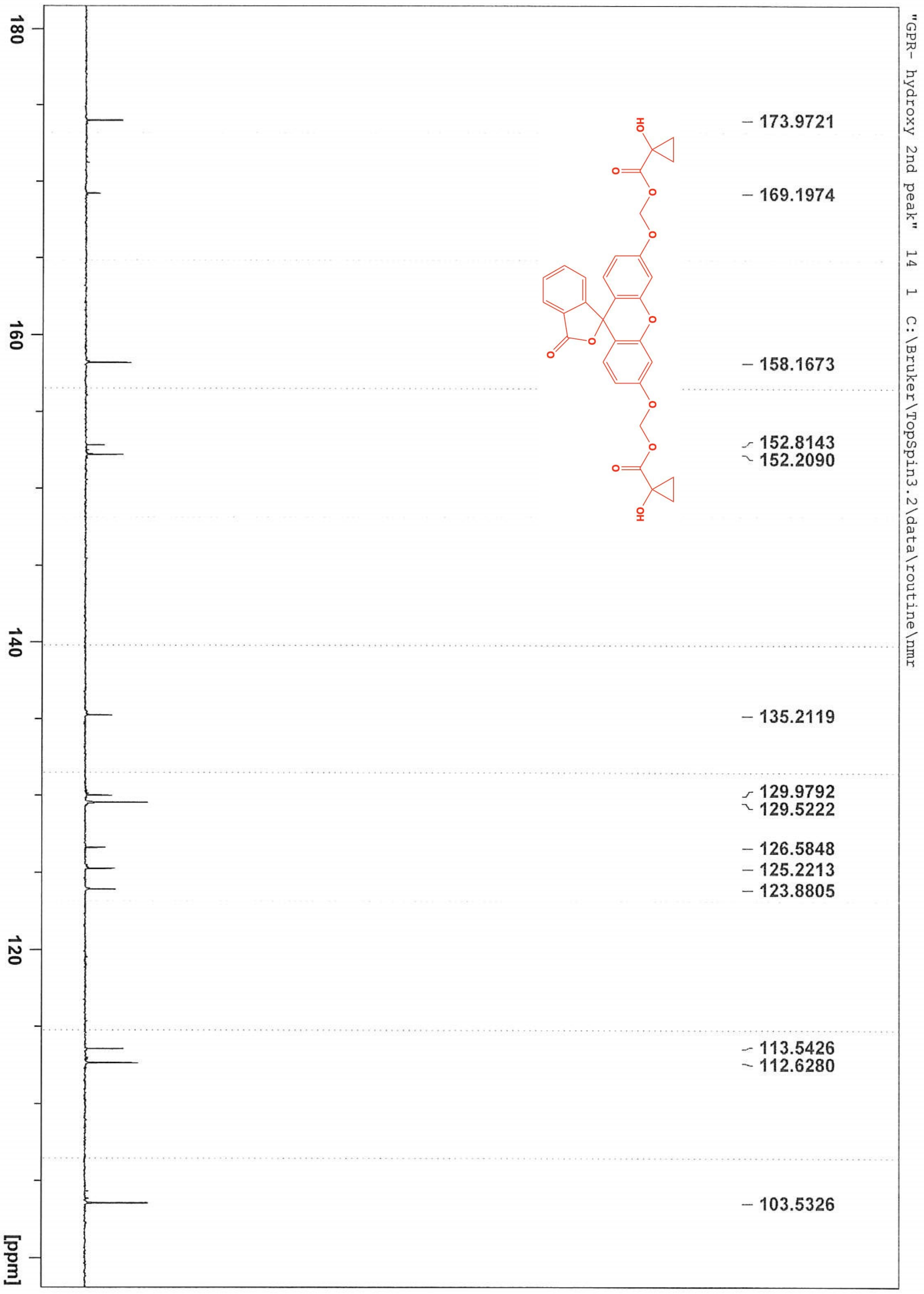
"GPR- hydroxy 2nd peak" 14 1 C:\Bruker\TopSpin3.2\data\routine\nmr
173.9721
169.1974
158.1673
152.8143
152.2090
135.2119
129.9792
129.5222
126.5848
125.2213
123.8805
113.5426
112.6280
103.5326
180
160
140
120
[ppm]
HO
O
OH

Figure S87

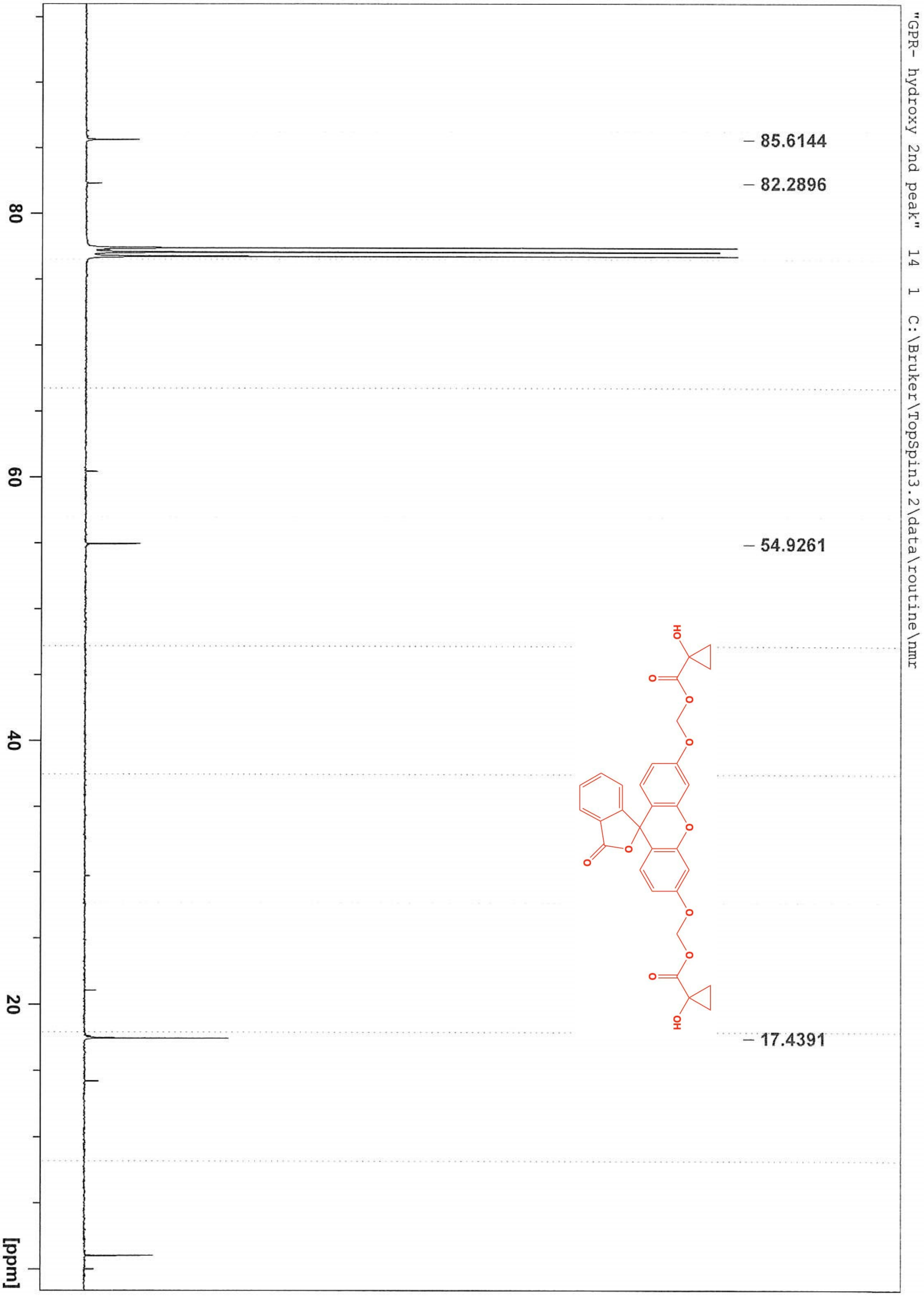

"GPR- hydroxy 2nd peak" 14 1 C:\Bruker\TopSpin3.2\data\routine\nmr
85.6144
82.2896
54.9261
17.4391
80
60
40
20
[ppm]
HO
OH

Figure S88

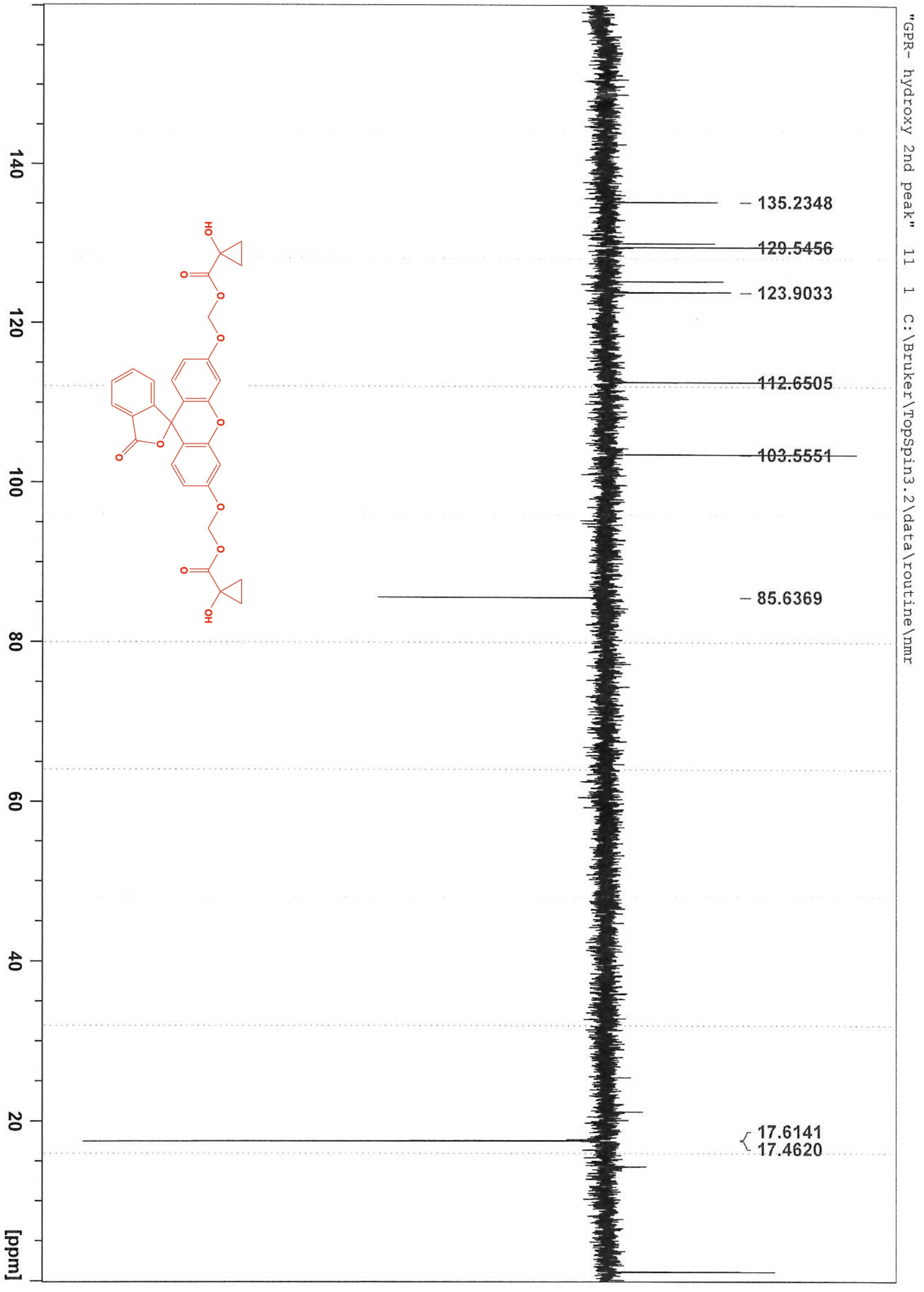
"GPR- hydroxy 2nd peak" 11 1 C:\Bruker\TopSpin3.2\data\routine\nmr
135.2348
129.5456
123.9033
112.6505
103.5551
85.6369
17.6141
17.4620
140
120
100
80
60
40
20
[ppm]

Figure S89

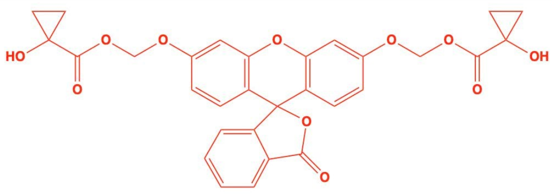
HO
O
O
O
O
O
OH
O
O
O

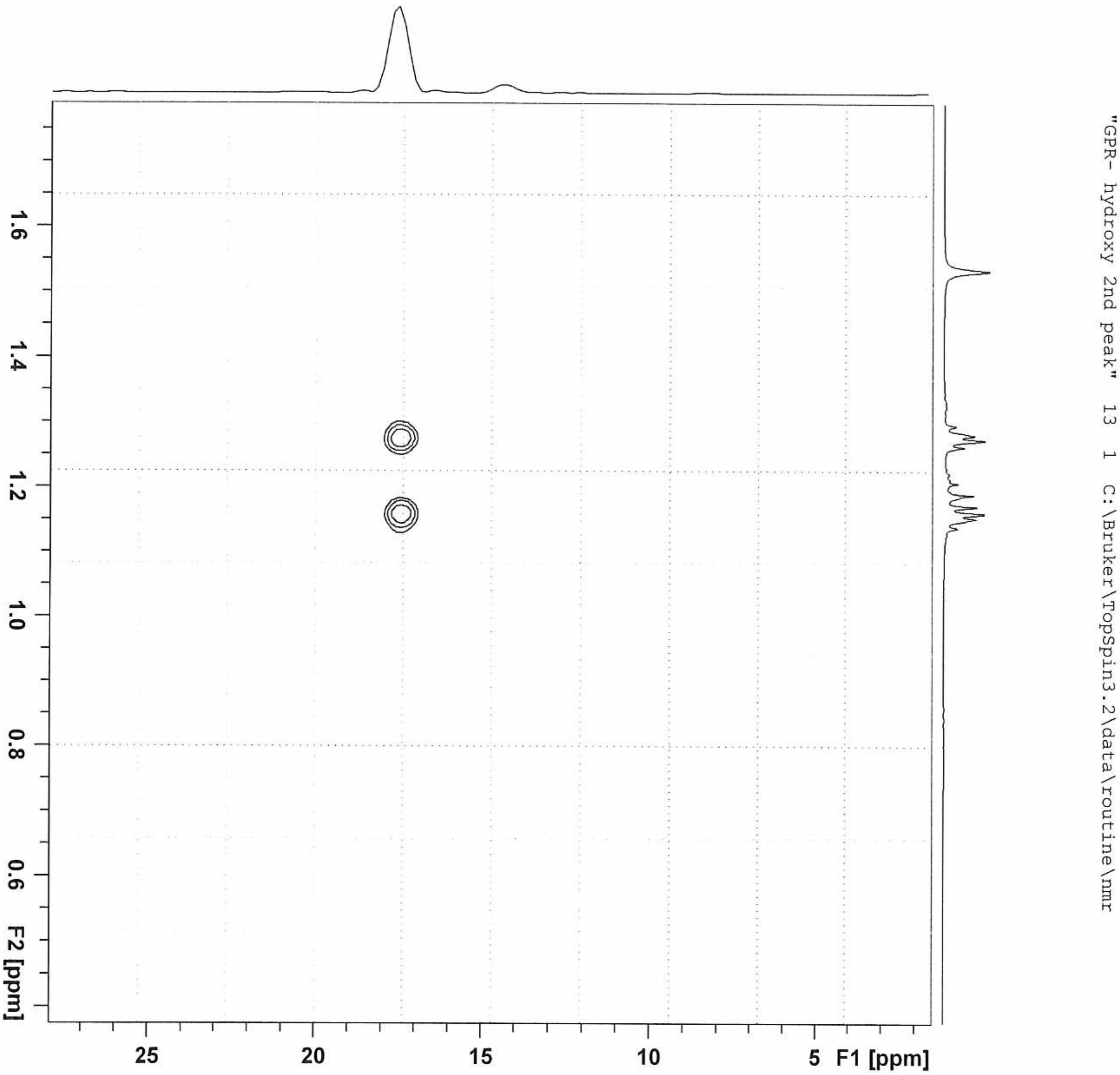
"GPR- hydroxy 2nd peak" 13 1 C:\Bruker\TopSpin3.2\data\routine\nmr
F2 [ppm]
0.6
0.8
1.0
1.2
1.4
1.6
25
20
15
10
5
F1 [ppm]

Figure S90

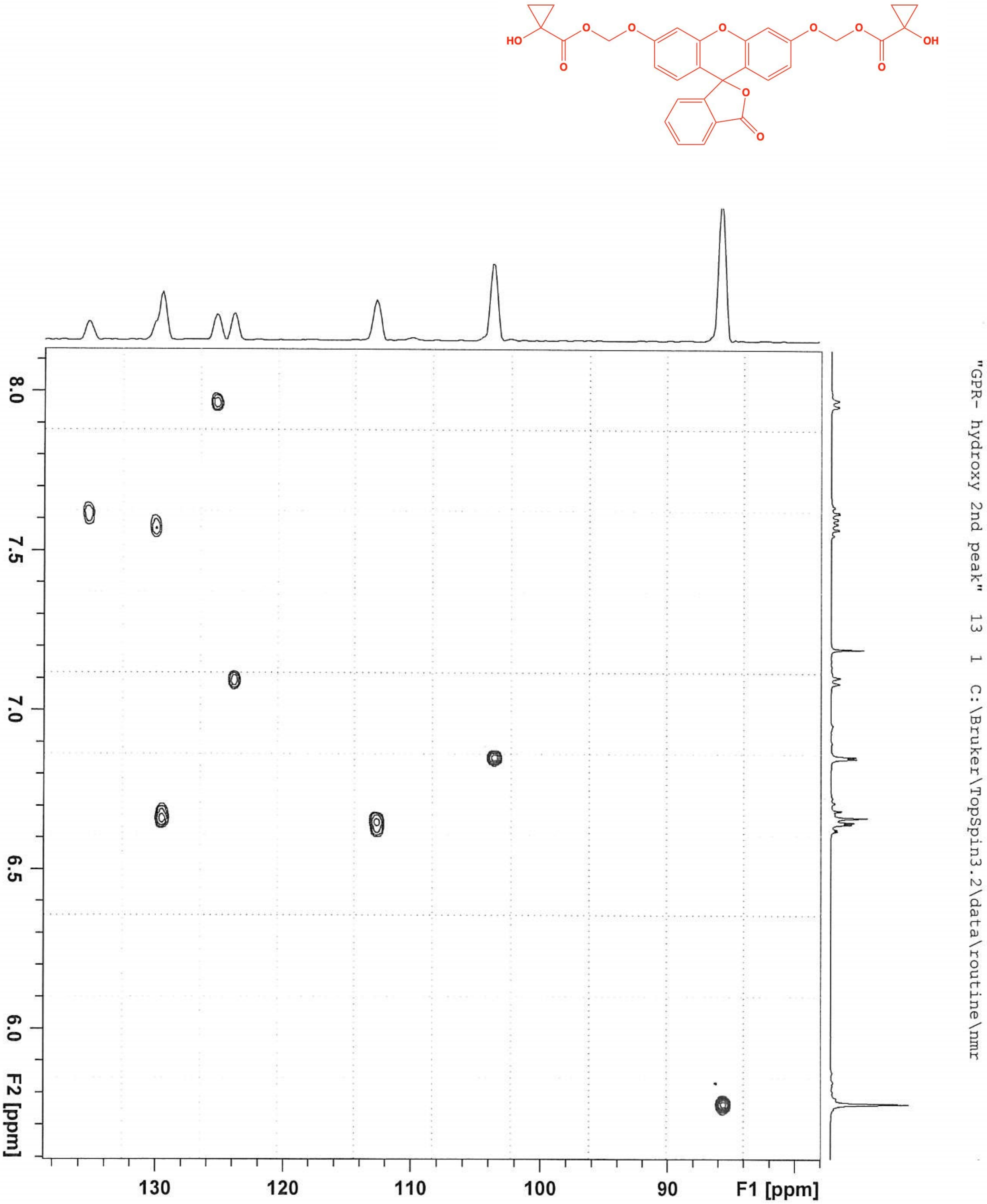
HO
O
O
O
O
O
O
OH
O
O
O
O
8.0
7.5
7.0
6.5
6.0
F2 [ppm]
130
120
110
100
90
F1 [ppm]
"GPR- hydroxy 2nd peak" 13 1 C:\Bruker\TopSpin3.2\data\routine\nmr

Figure S91

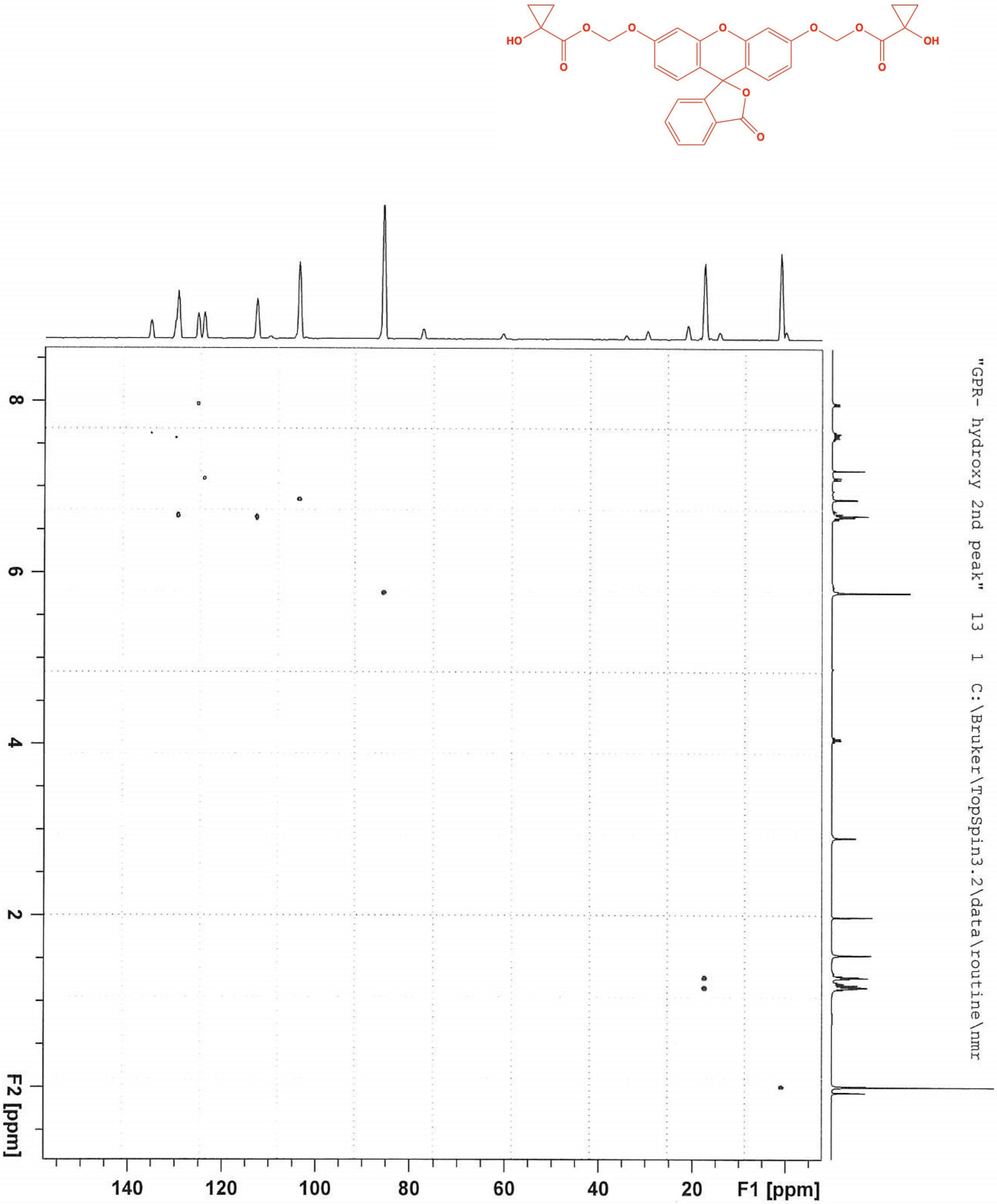

"GPR- hydroxy 2nd peak" 13 1 C:\Bruker\TopSpin3.2\data\routine\nmr
F2 [ppm]
F1 [ppm]

Figure S92

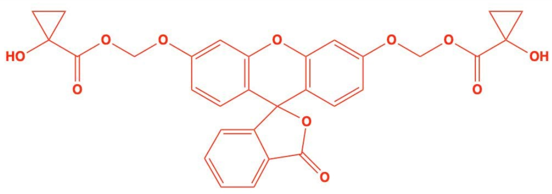
HO
OH

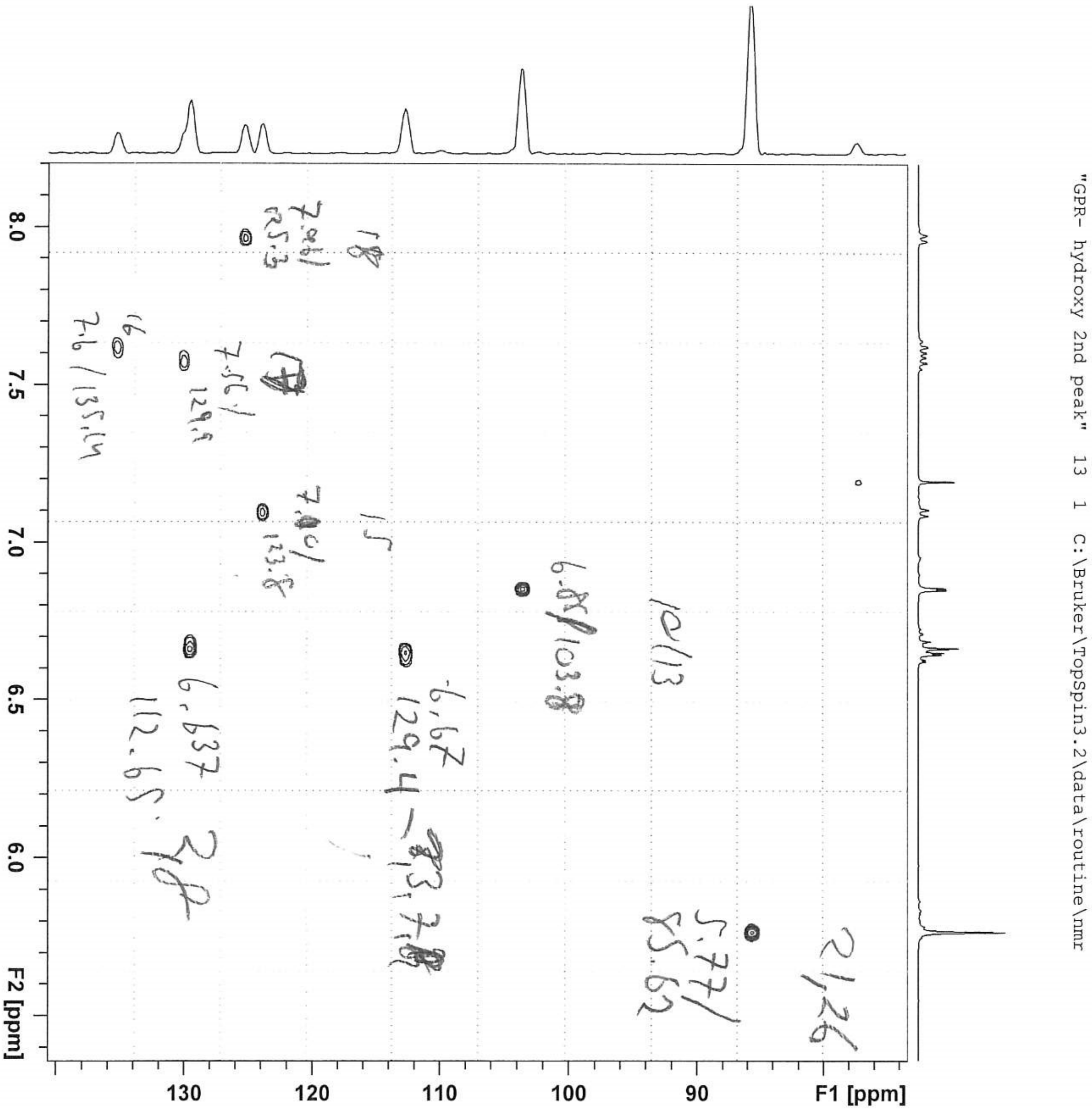
"GPR- hydroxy 2nd peak" 13 1 C:\Bruker\TopSpin3.2\data\routine\nmr
F1 [ppm]
F2 [ppm]

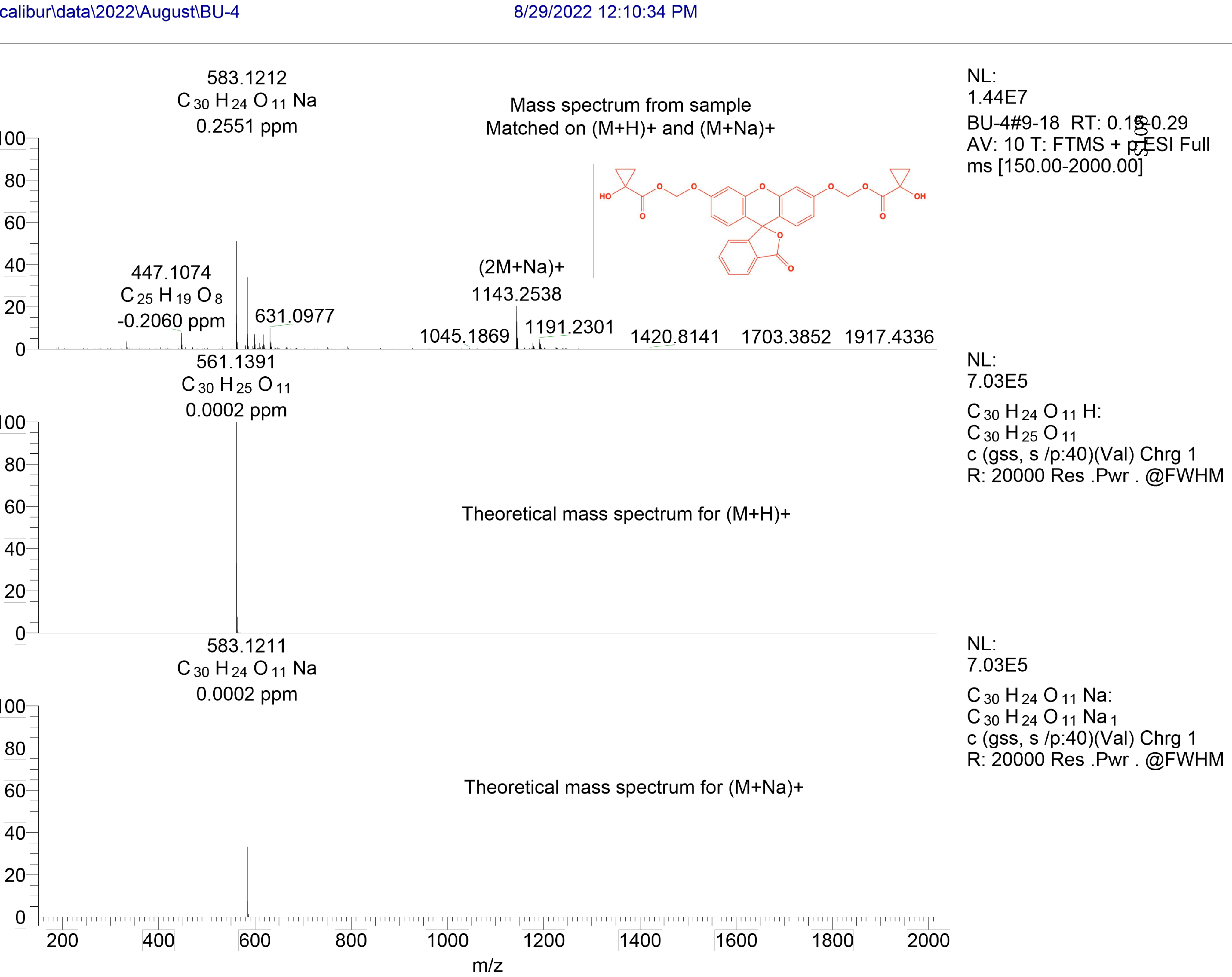

C:\Xcalibur\data\2022\August\BU-4
8/29/2022 12:10:34 PM
Mass spectrum from sample
Matched on (M+H)+ and (M+Na)+
583.1212
C 30 H 24 O 11 Na
0.2551 ppm
447.1074
C 25 H 19 O 8
-0.2060 ppm
631.0977
(2M+Na)+
1143.2538
1045.1869
1191.2301
1420.8141
1703.3852
1917.4336
NL:
1.44E7
BU-4#9-18 RT: 0.15-0.29
AV: 10 T: FTMS + p ESI Full
ms [150.00-2000.00]
561.1391
C 30 H 25 O 11
0.0002 ppm
Theoretical mass spectrum for (M+H)+
NL:
7.03E5
C 30 H 24 O 11 H:
C 30 H 25 O 11
c (gss, s /p:40)(Val) Chrg 1
R: 20000 Res .Pwr . @FWHM
583.1211
C 30 H 24 O 11 Na
0.0002 ppm
Theoretical mass spectrum for (M+Na)+
NL:
7.03E5
C 30 H 24 O 11 Na:
C 30 H 24 O 11 Na 1
c (gss, s /p:40)(Val) Chrg 1
R: 20000 Res .Pwr . @FWHM
Relative Abundance
m/z
HO
OH

Figure S93

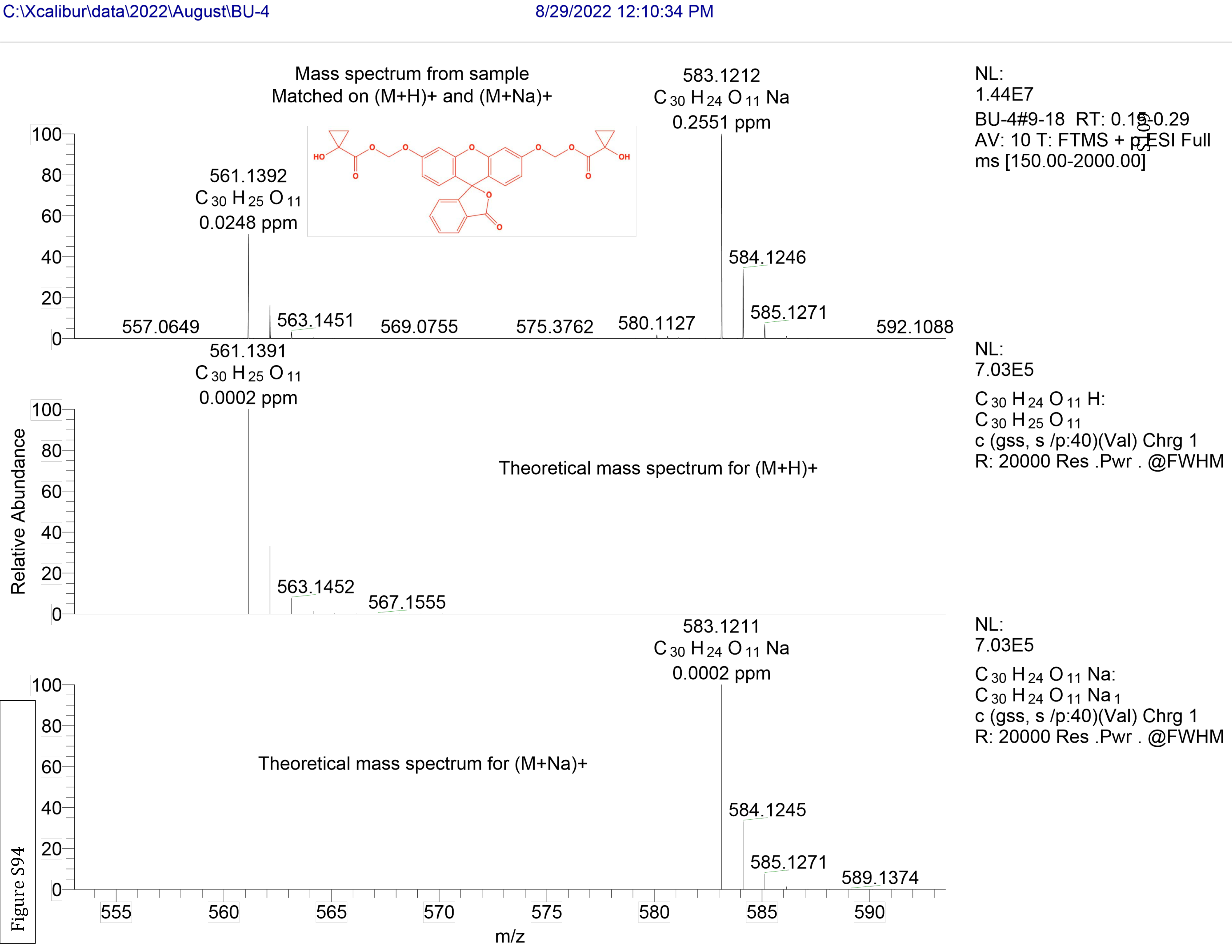

C:\Xcalibur\data\2022\August\BU-4
8/29/2022 12:10:34 PM
Mass spectrum from sample
Matched on (M+H)+ and (M+Na)+
561.1392
C 30 H 25 O 11
0.0248 ppm
583.1212
C 30 H 24 O 11 Na
0.2551 ppm
557.0649
563.1451
569.0755
575.3762
580.1127
584.1246
585.1271
592.1088
NL:
1.44E7
BU-4#9-18 RT: 0.15-0.29
AV: 10 T: FTMS + p ESI Full
ms [150.00-2000.00]
561.1391
C 30 H 25 O 11
0.0002 ppm
563.1452
567.1555
Theoretical mass spectrum for (M+H)+
NL:
7.03E5
C 30 H 24 O 11 H:
C 30 H 25 O 11
c (gss, s /p:40)(Val) Chrg 1
R: 20000 Res .Pwr . @FWHM
583.1211
C 30 H 24 O 11 Na
0.0002 ppm
584.1245
585.1271
589.1374
Theoretical mass spectrum for (M+Na)+
NL:
7.03E5
C 30 H 24 O 11 Na:
C 30 H 24 O 11 Na 1
c (gss, s /p:40)(Val) Chrg 1
R: 20000 Res .Pwr . @FWHM
Relative Abundance
m/z

Figure S94

Figure S95

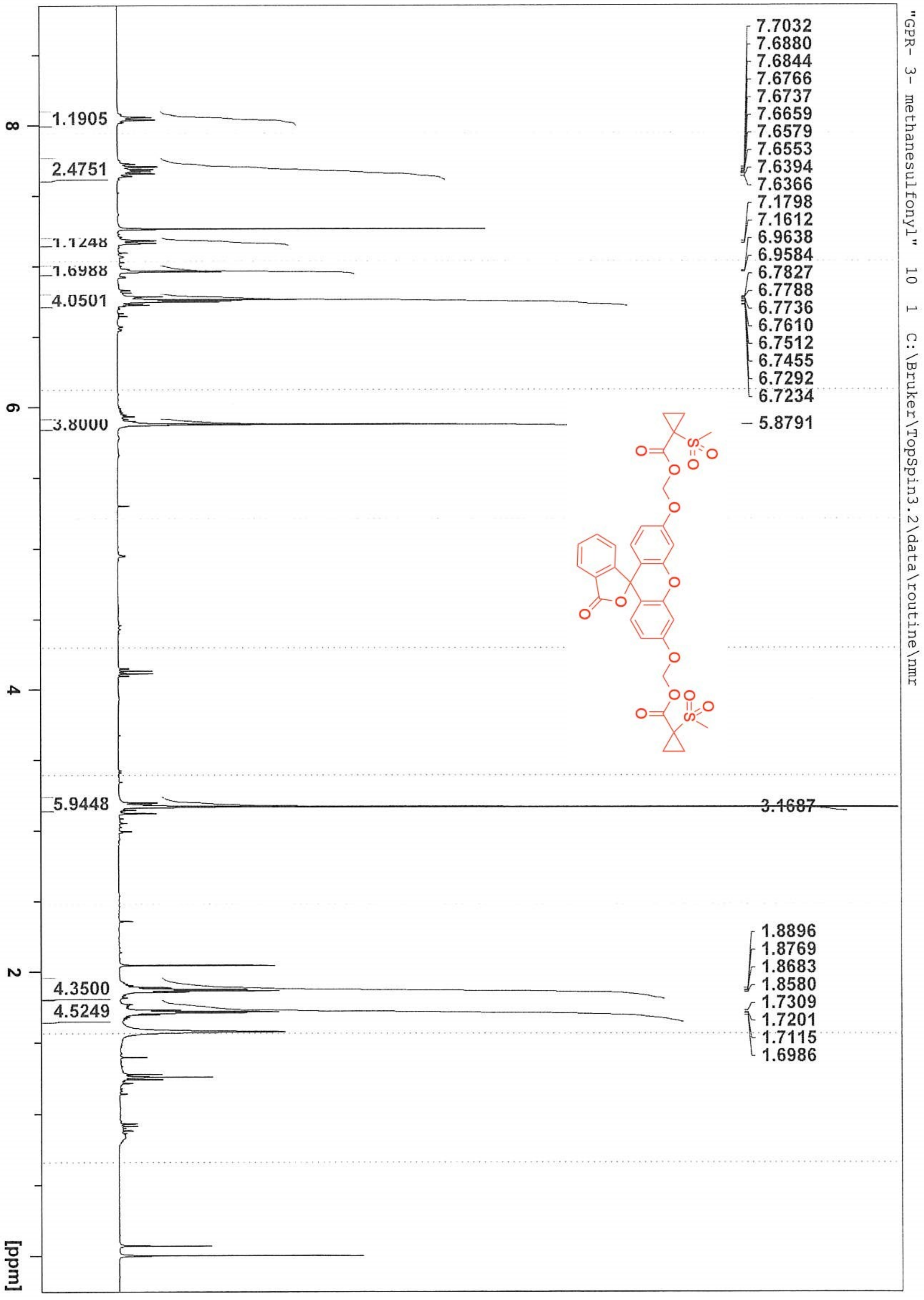
"GPR- 3- methanesulfonyl" 10 1 C:\Bruker\TopSpin3.2\data\routine\nmr
7.7032
7.6880
7.6844
7.6766
7.6737
7.6659
7.6579
7.6553
7.6394
7.6366
7.1798
7.1612
6.9638
6.9584
6.7827
6.7788
6.7736
6.7610
6.7512
6.7455
6.7292
6.7234
5.8791
3.1687
1.8896
1.8769
1.8683
1.8580
1.7309
1.7201
1.7115
1.6986
1.1905
2.4751
1.1248
1.6988
4.0501
3.8000
5.9448
4.3500
4.5249
8
6
4
2
[ppm]

Figure S96

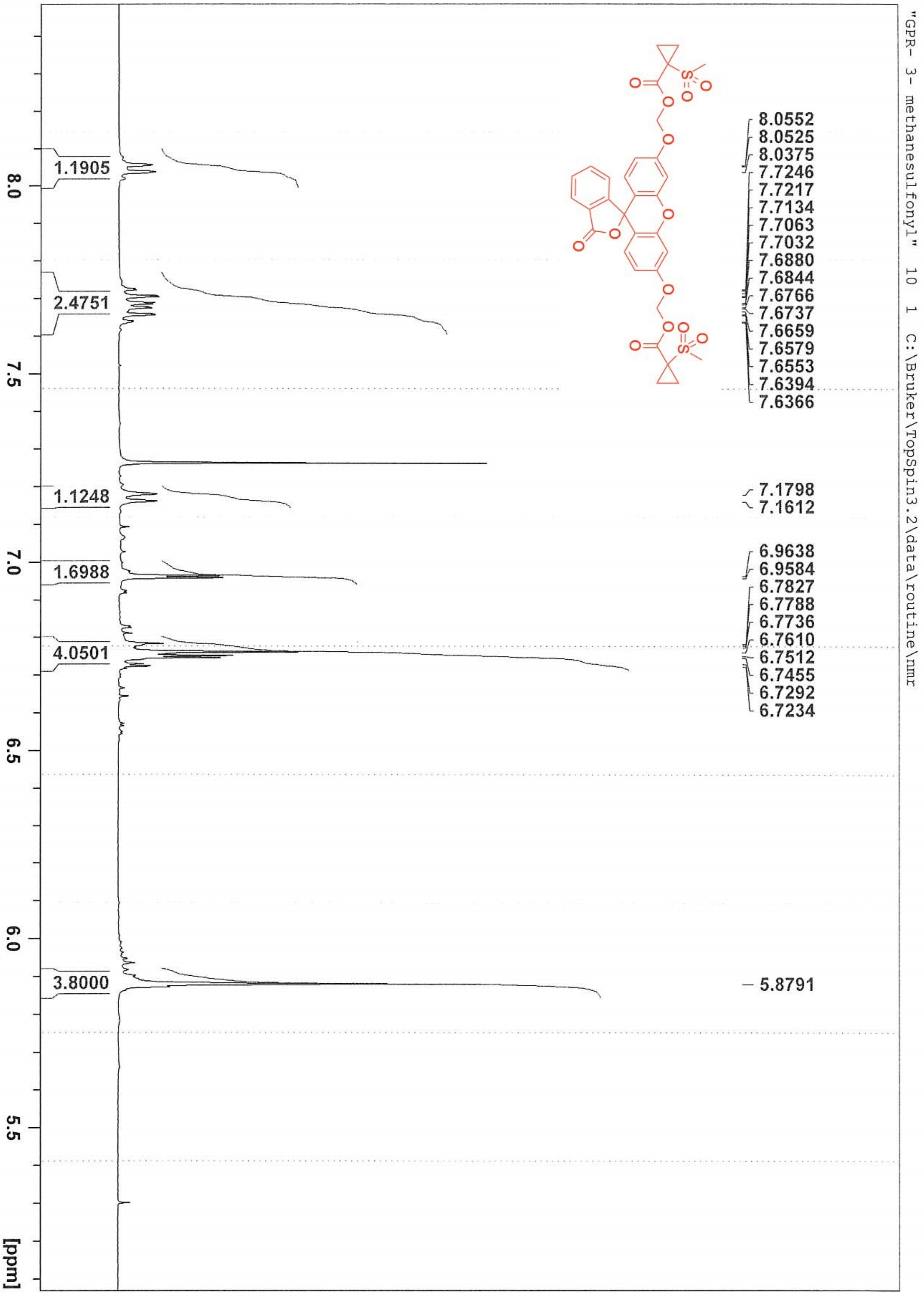
"GPR- 3- methanesulfonyl" 10 1 C:\Bruker\TopSpin3.2\data\routine\nmr
8.0552
8.0525
8.0375
7.7246
7.7217
7.7134
7.7063
7.7032
7.6880
7.6844
7.6766
7.6737
7.6659
7.6579
7.6553
7.6394
7.6366
7.1798
7.1612
6.9638
6.9584
6.7827
6.7788
6.7736
6.7610
6.7512
6.7455
6.7292
6.7234
5.8791
1.1905
2.4751
1.1248
1.6988
4.0501
3.8000
8.0
7.5
7.0
6.5
6.0
5.5
[ppm]

Figure S97

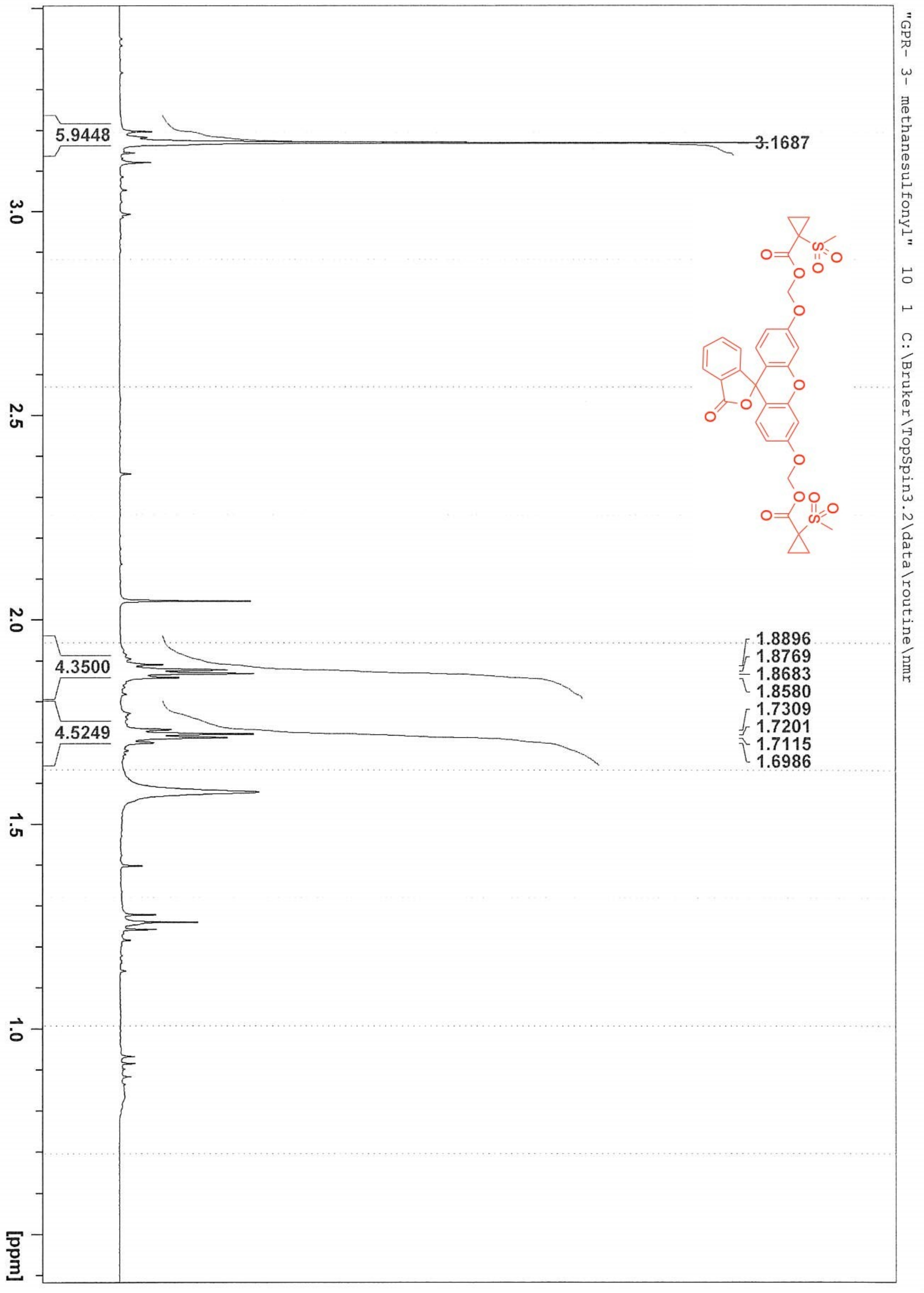

"GPR- 3- methanesulfonyl" 10 1 C:\Bruker\TopSpin3.2\data\routine\nmr
3.1687
1.8896
1.8769
1.8683
1.8580
1.7309
1.7201
1.7115
1.6986
5.9448
4.3500
4.5249
3.0
2.5
2.0
1.5
1.0
[ppm]

Figure S98

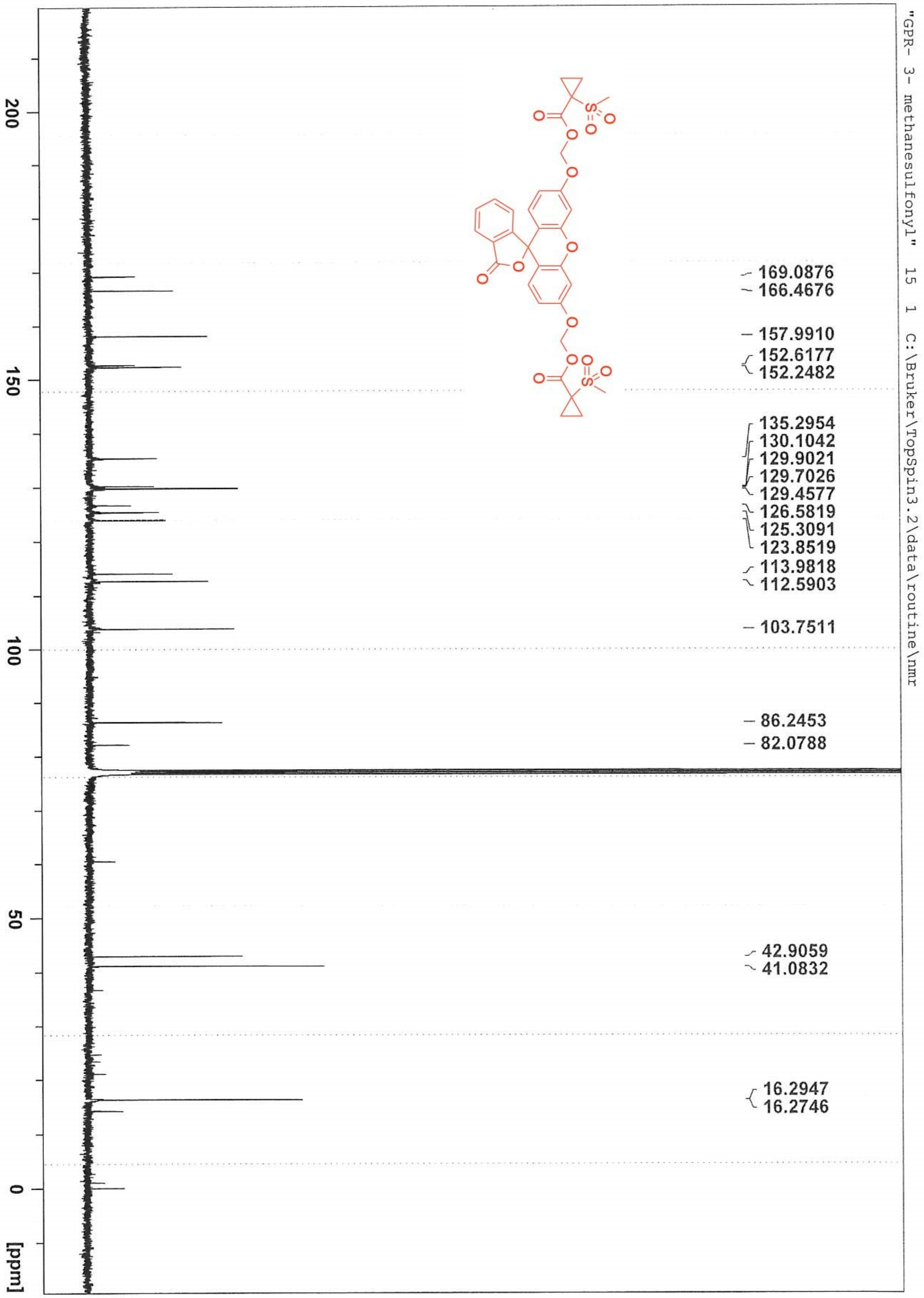

"GPR- 3- methanesulfonyl" 15 1 C:\Bruker\TopSpin3.2\data\routine\nmr
169.0876
166.4676
157.9910
152.6177
152.2482
135.2954
130.1042
129.9021
129.7026
129.4577
126.5819
125.3091
123.8519
113.9818
112.5903
103.7511
86.2453
82.0788
42.9059
41.0832
16.2947
16.2746
200
150
100
50
0
[ppm]

Figure S99

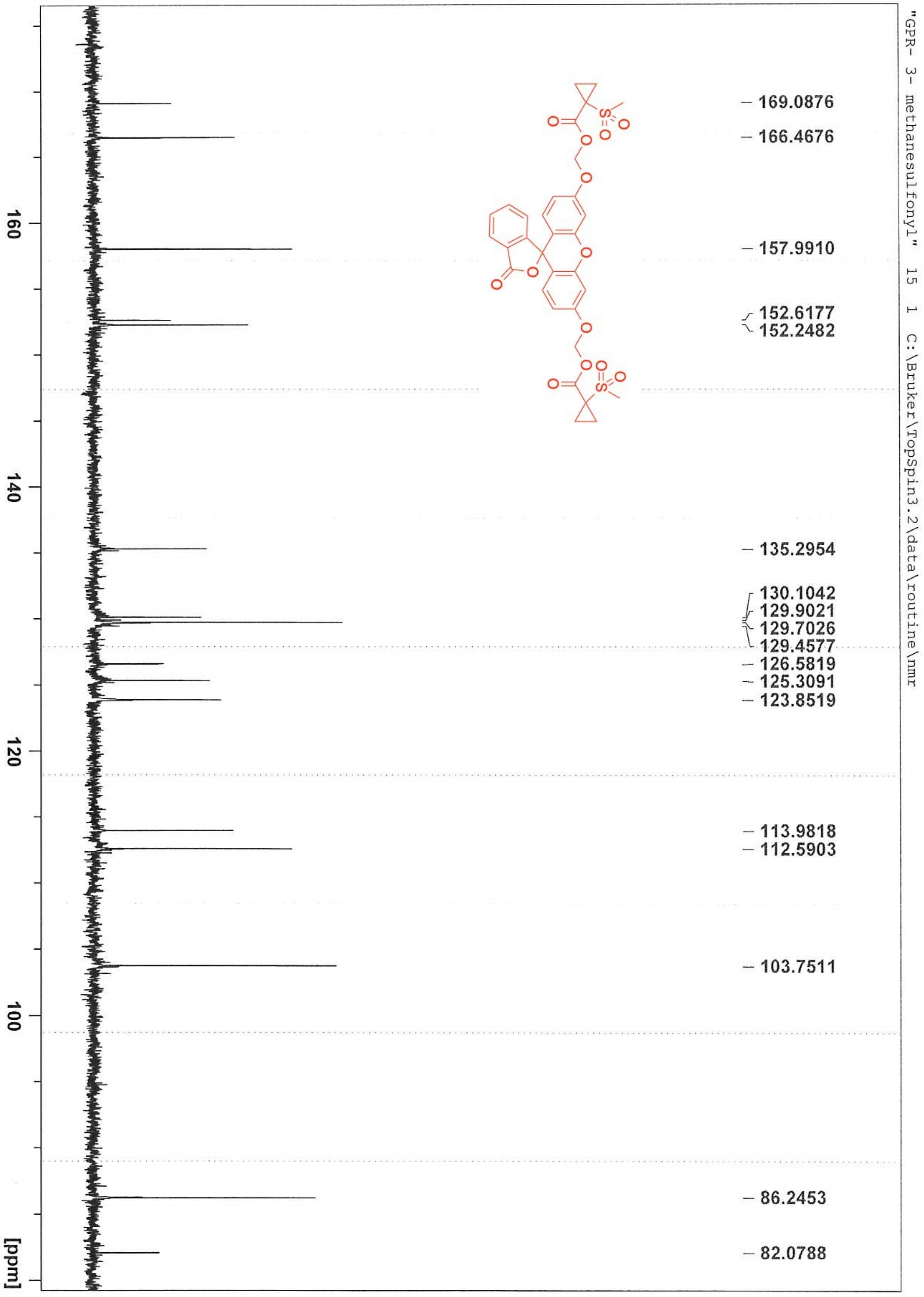
"GPR- 3- methanesulfonyl" 15 1 C:\Bruker\TopSpin3.2\data\routine\nmr
169.0876
166.4676
157.9910
152.6177
152.2482
135.2954
130.1042
129.9021
129.7026
129.4577
126.5819
125.3091
123.8519
113.9818
112.5903
103.7511
86.2453
82.0788
160
140
120
100
[ppm]

Figure S100

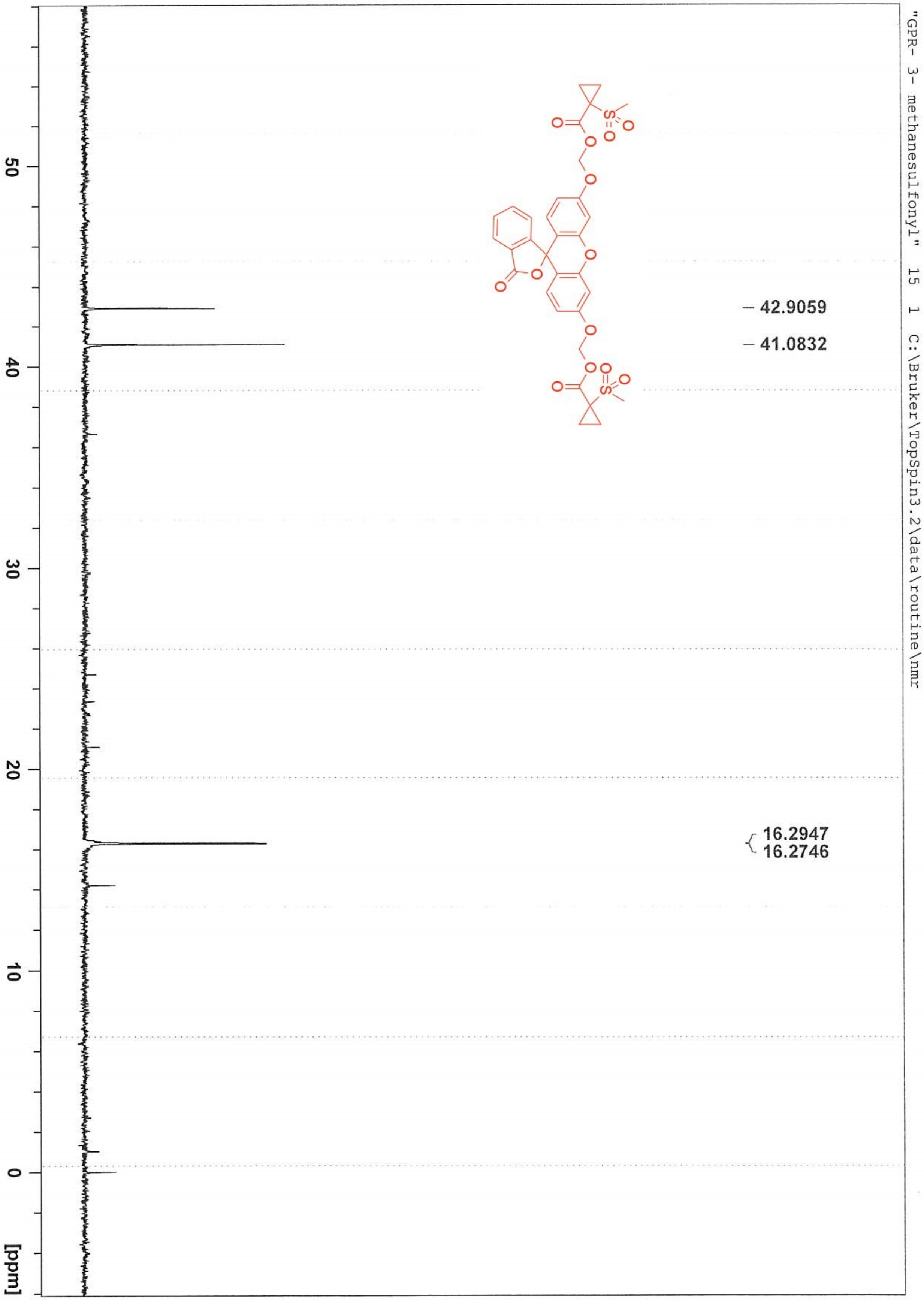

"GPR- 3- methanesulfonyl" 15 1 C:\Bruker\TopSpin3.2\data\routine\nmr
42.9059
41.0832
16.2947
16.2746
50
40
30
20
10
0
[ppm]

Figure S101

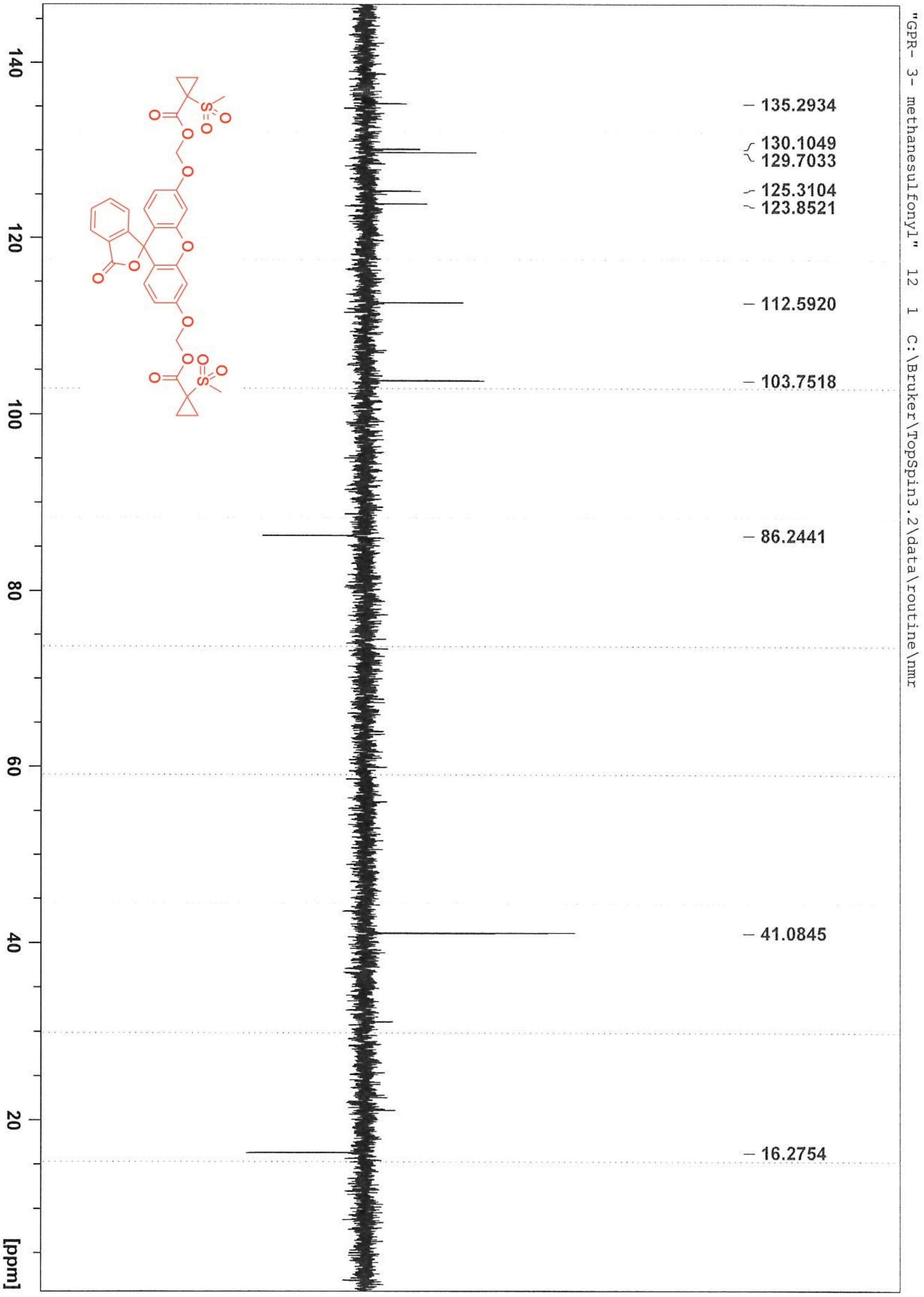

"GPR- 3- methanesulfonyl" 12 1 C:\Bruker\TopSpin3.2\data\routine\nmr
135.2934
130.1049
129.7033
125.3104
123.8521
112.5920
103.7518
86.2441
41.0845
16.2754
140
120
100
80
60
40
20
[ppm]

Figure S102

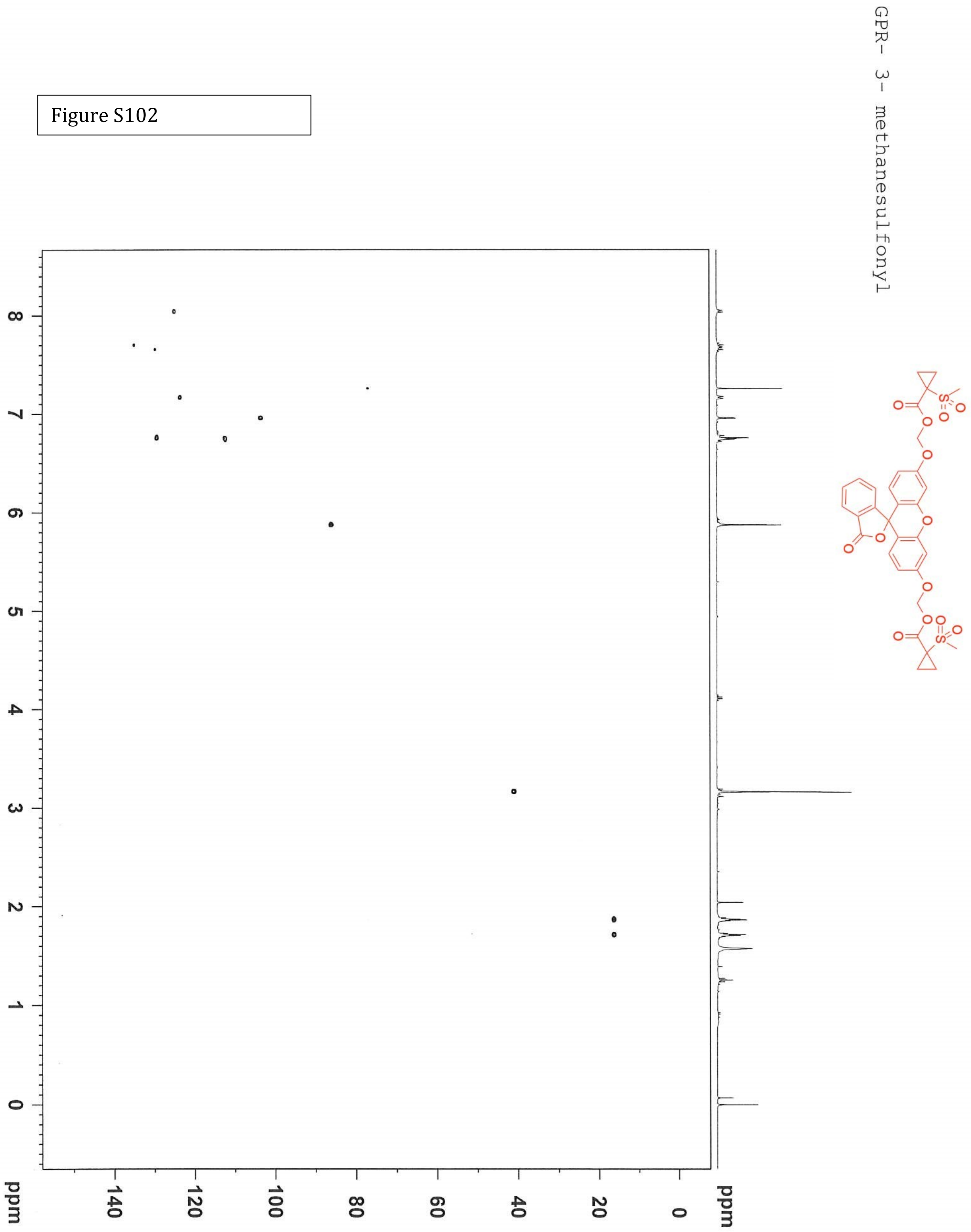
GPR- 3- methanesulfonyl
ppm
8
7
6
5
4
3
2
1
0
ppm
140
120
100
80
60
40
20
0

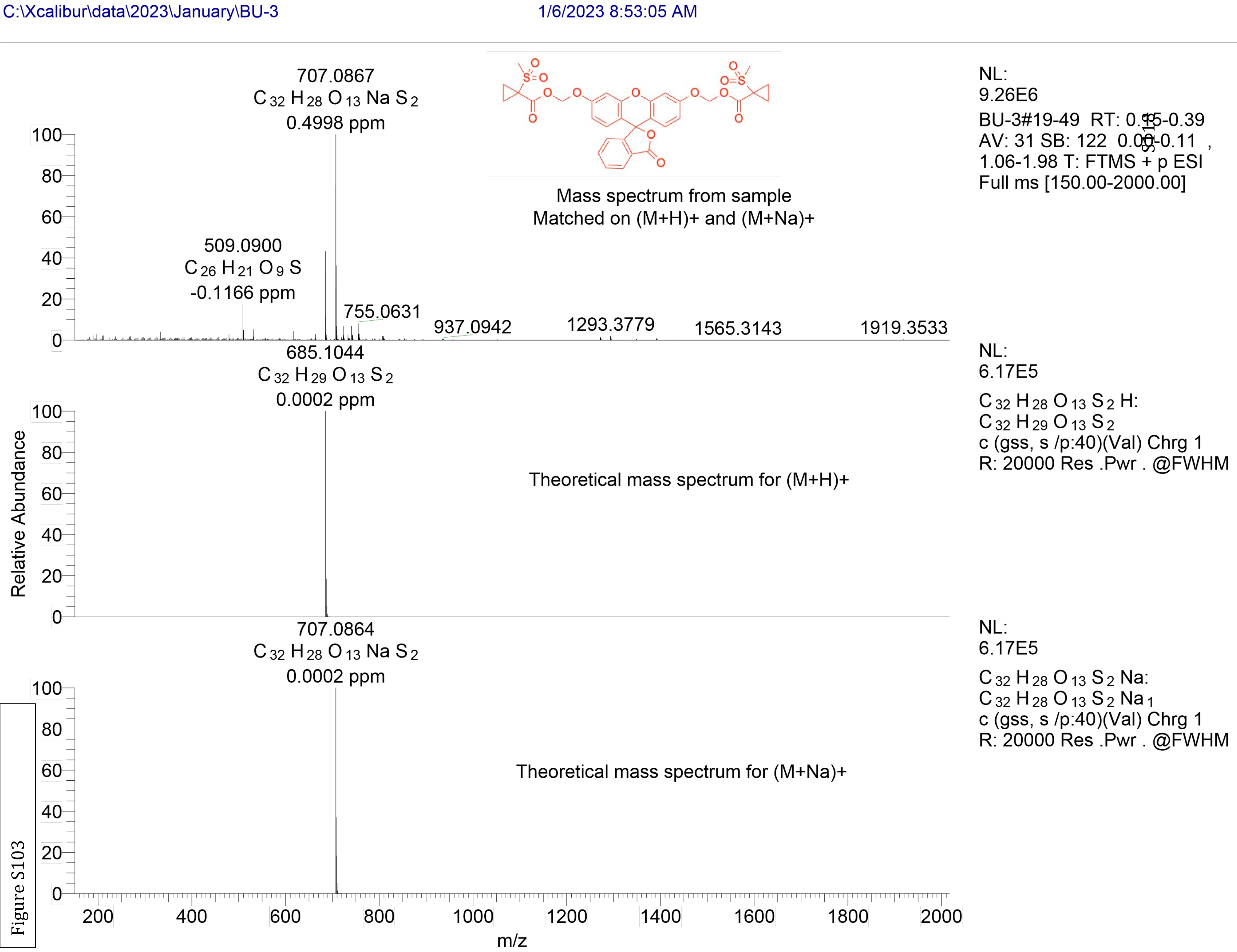
C:\Xcalibur\data\2023\January\BU-3
1/6/2023 8:53:05 AM
707.0867
C 32 H 28 O 13 Na S 2
0.4998 ppm
509.0900
C 26 H 21 O 9 S
-0.1166 ppm
755.0631
937.0942
1293.3779
1565.3143
1919.3533
Mass spectrum from sample
Matched on (M+H)+ and (M+Na)+
NL:
9.26E6
BU-3#19-49 RT: 0.15-0.39
AV: 31 SB: 122 0.00-0.11 ,
1.06-1.98 T: FTMS + p ESI
Full ms [150.00-2000.00]
685.1044
C 32 H 29 O 13 S 2
0.0002 ppm
Theoretical mass spectrum for (M+H)+
NL:
6.17E5
C 32 H 28 O 13 S 2 H:
C 32 H 29 O 13 S 2
c (gss, s /p:40)(Val) Chrg 1
R: 20000 Res .Pwr . @FWHM
707.0864
C 32 H 28 O 13 Na S 2
0.0002 ppm
Theoretical mass spectrum for (M+Na)+
NL:
6.17E5
C 32 H 28 O 13 S 2 Na:
C 32 H 28 O 13 S 2 Na 1
c (gss, s /p:40)(Val) Chrg 1
R: 20000 Res .Pwr . @FWHM
Relative Abundance
m/z

Figure S103

C:\Xcalibur\data\2023\January\BU-3 1/6/2023 8:53:05 AM

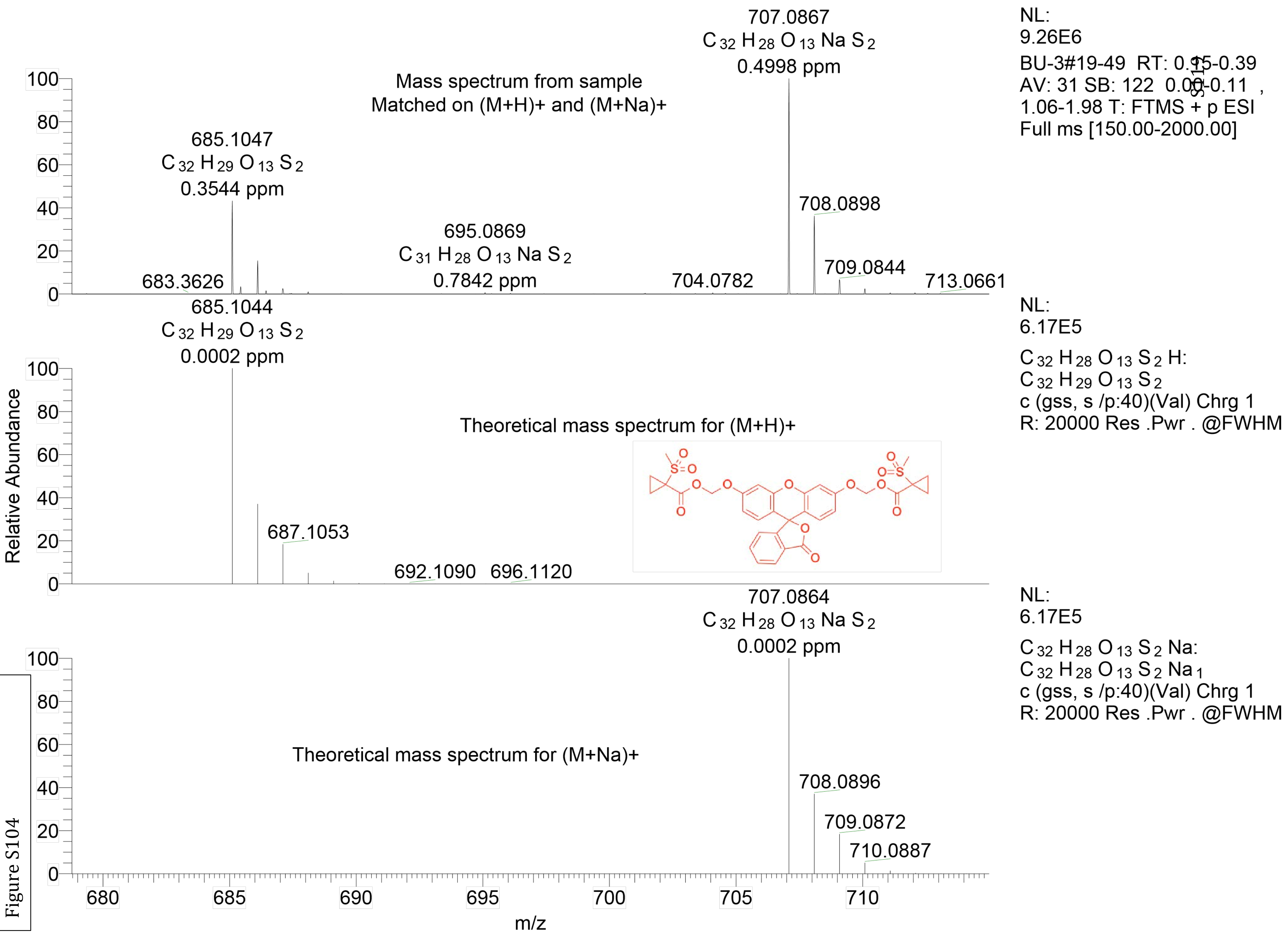

Figure S104

Figure S105

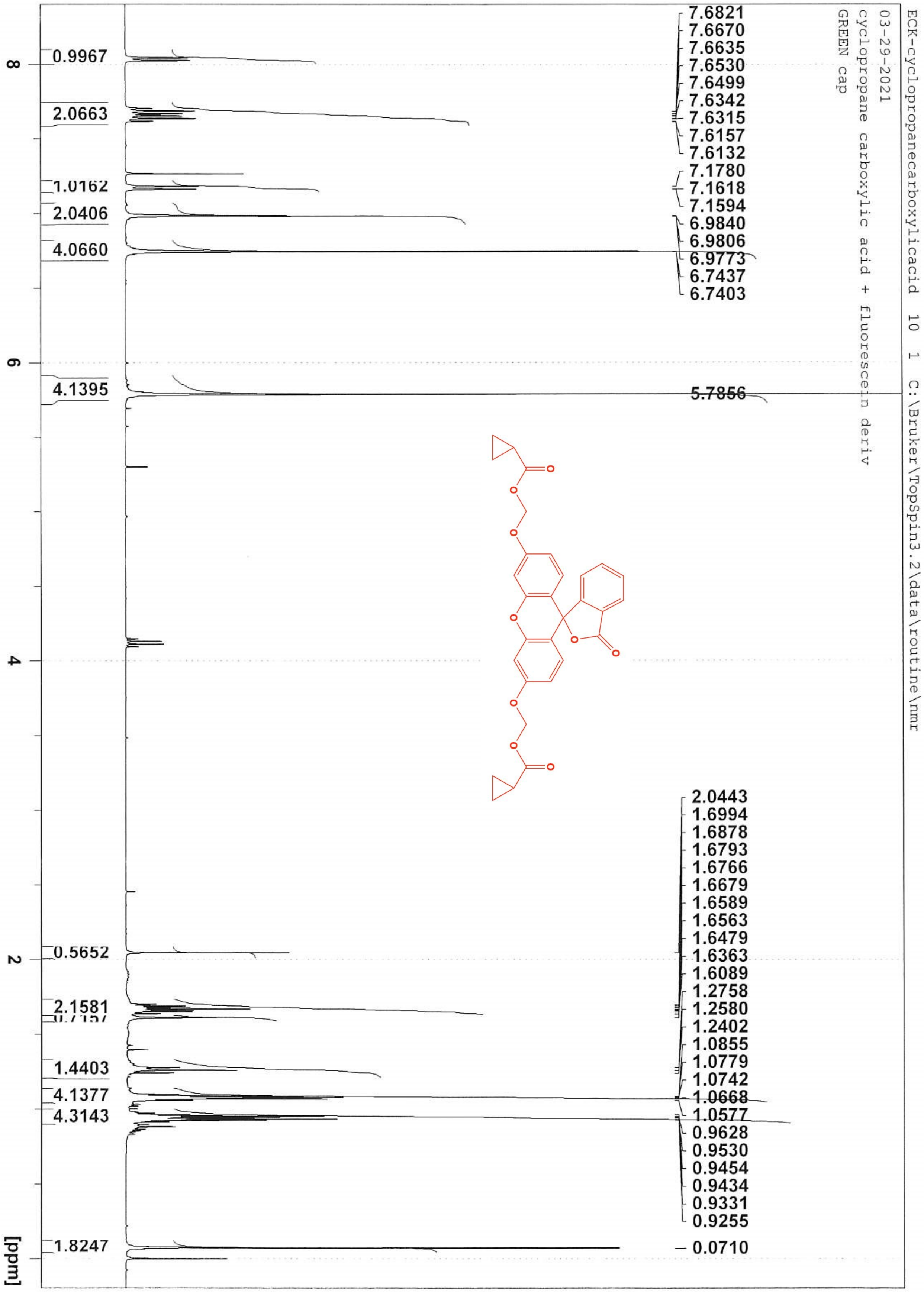

ECK-cyclopropanecarboxylicacid 10 1 C:\Bruker\TopSpin3.2\data\routine\nmr
03-29-2021
cyclopropane carboxylic acid + fluorescein deriv
GREEN cap
7.6821
7.6670
7.6635
7.6530
7.6499
7.6342
7.6315
7.6157
7.6132
7.1780
7.1618
7.1594
6.9840
6.9806
6.9773
6.7437
6.7403
5.7856
2.0443
1.6994
1.6878
1.6793
1.6766
1.6679
1.6589
1.6563
1.6479
1.6363
1.6089
1.2758
1.2580
1.2402
1.0855
1.0779
1.0742
1.0668
1.0577
0.9628
0.9530
0.9454
0.9434
0.9331
0.9255
0.0710
0.9967
2.0663
1.0162
2.0406
4.0660
4.1395
0.5652
2.1581
1.4403
4.1377
4.3143
1.8247
8
6
4
2
[ppm]

Figure S106

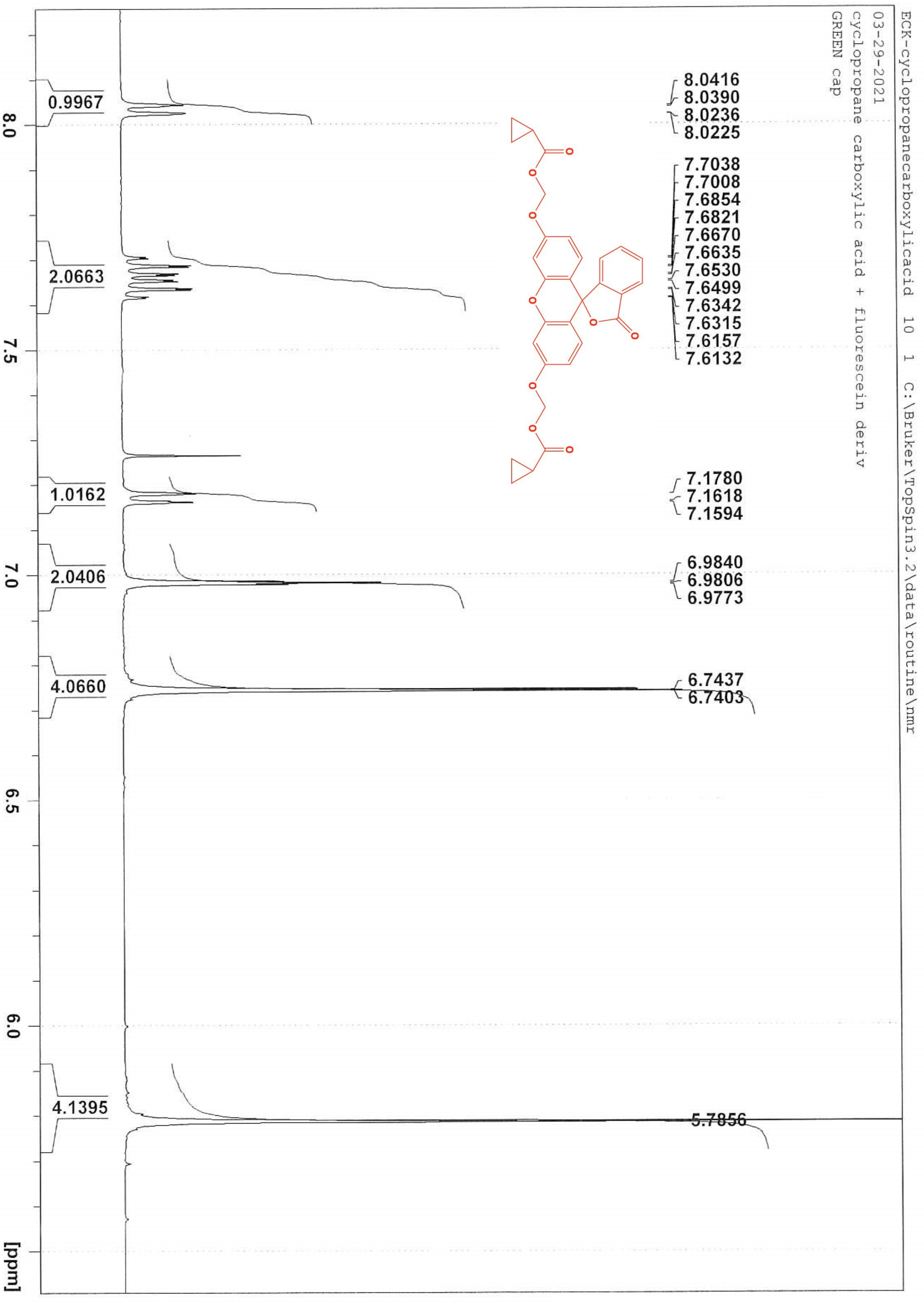

ECK-cyclopropanecarboxylicacid 10 1 C:\Bruker\TopSpin3.2\data\routine\nmr
03-29-2021
cyclopropane carboxylic acid + fluorescein deriv
GREEN cap
8.0416
8.0390
8.0236
8.0225
7.7038
7.7008
7.6854
7.6821
7.6670
7.6635
7.6530
7.6499
7.6342
7.6315
7.6157
7.6132
7.1780
7.1618
7.1594
6.9840
6.9806
6.9773
6.7437
6.7403
5.7856
0.9967
2.0663
1.0162
2.0406
4.0660
4.1395
8.0
7.5
7.0
6.5
6.0
[ppm]

Figure S107

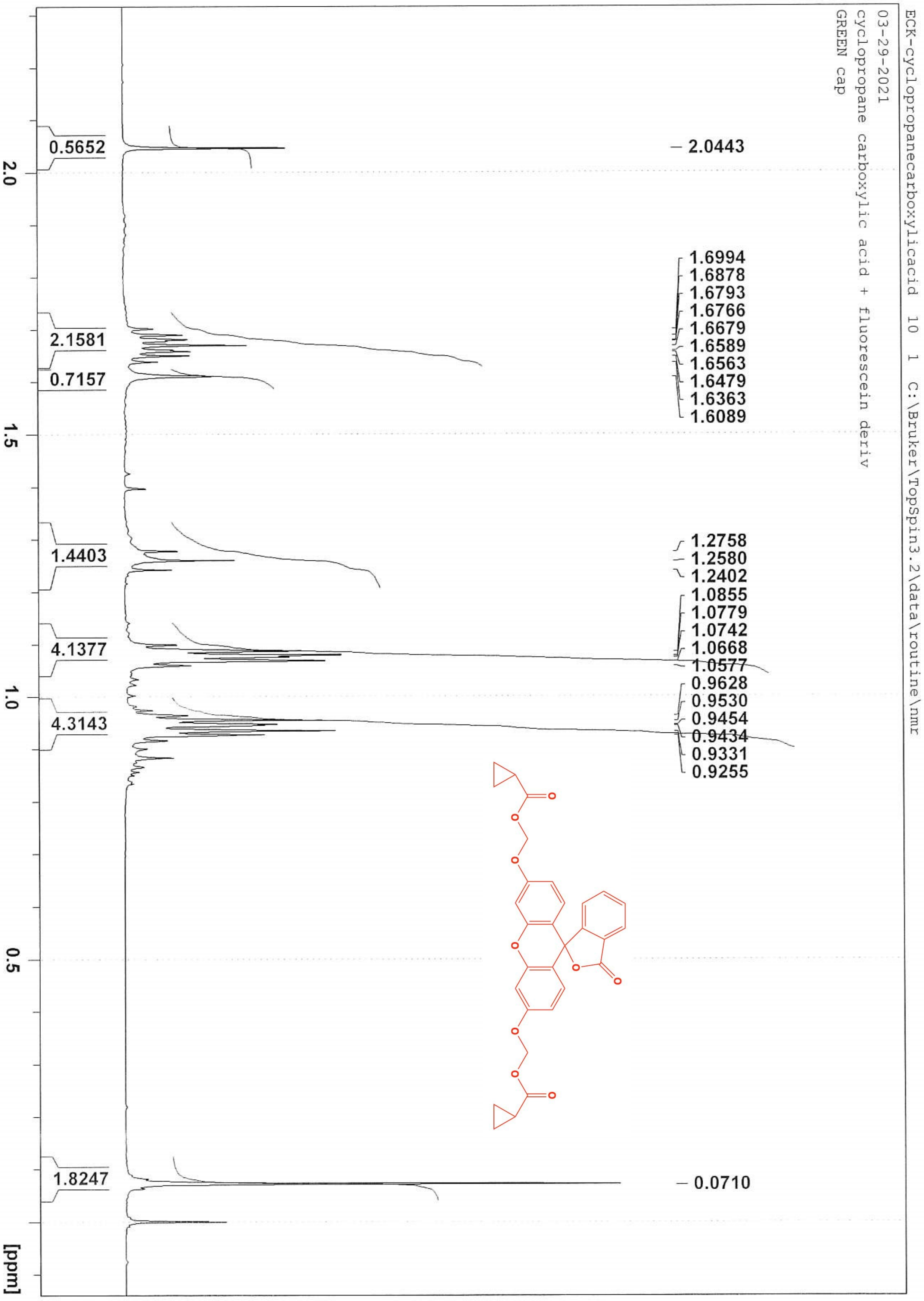

ECK-cyclopropanecarboxylicacid 10 1 C:\Bruker\TopSpin3.2\data\routine\nmr
03-29-2021
cyclopropane carboxylic acid + fluorescein deriv
GREEN cap
2.0443
1.6994
1.6878
1.6793
1.6766
1.6679
1.6589
1.6563
1.6479
1.6363
1.6089
1.2758
1.2580
1.2402
1.0855
1.0779
1.0742
1.0668
1.0577
0.9628
0.9530
0.9454
0.9434
0.9331
0.9255
0.0710
0.5652
2.1581
0.7157
1.4403
4.1377
4.3143
1.8247
2.0
1.5
1.0
0.5
[ppm]

Figure S108

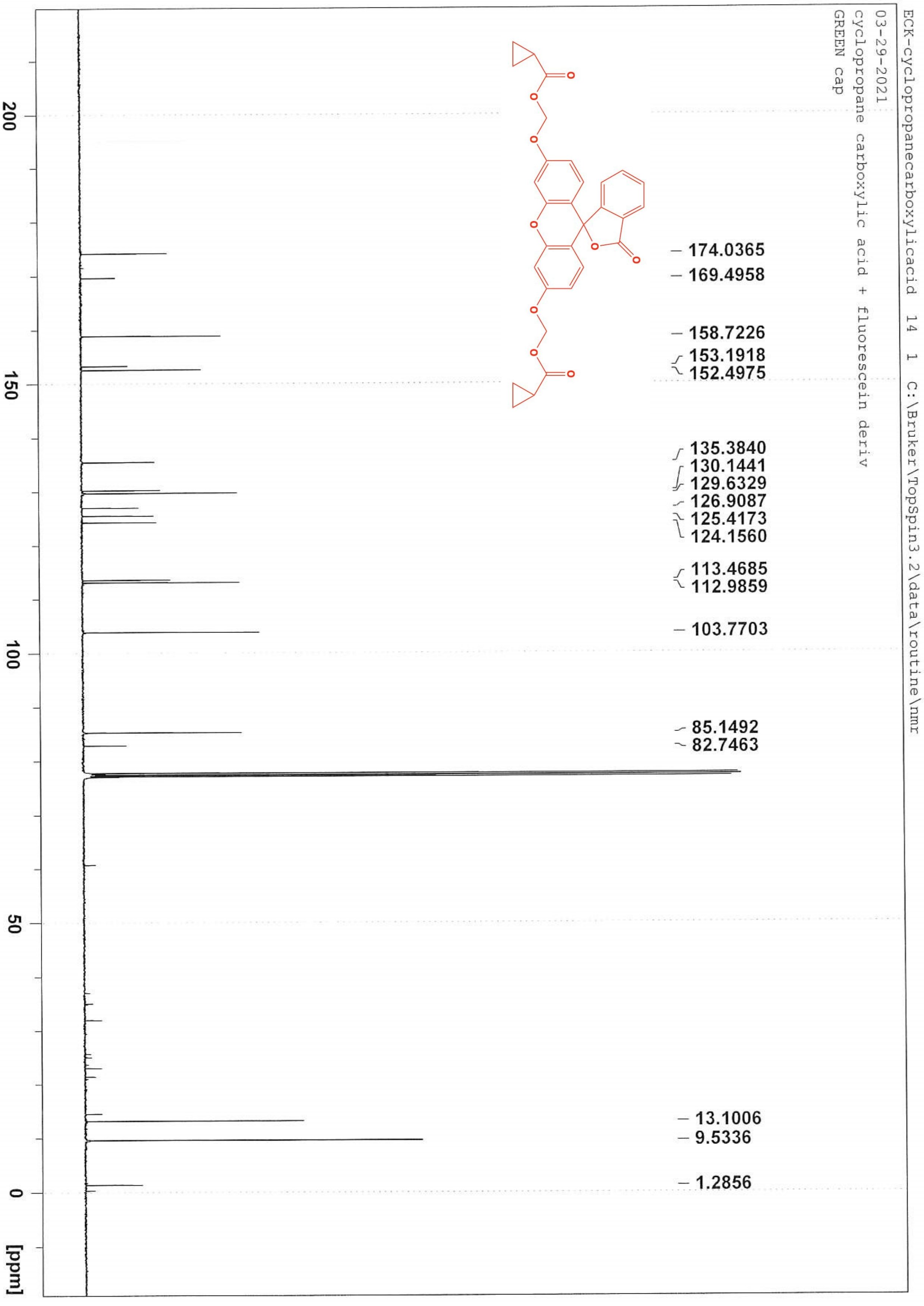
ECK-cyclopropanecarboxylicacid 14 1 C:\Bruker\TopSpin3.2\data\routine\nmr
03-29-2021
cyclopropane carboxylic acid + fluorescein deriv
GREEN cap
174.0365
169.4958
158.7226
153.1918
152.4975
135.3840
130.1441
129.6329
126.9087
125.4173
124.1560
113.4685
112.9859
103.7703
85.1492
82.7463
13.1006
9.5336
1.2856
200
150
100
50
0
[ppm]

Figure S109

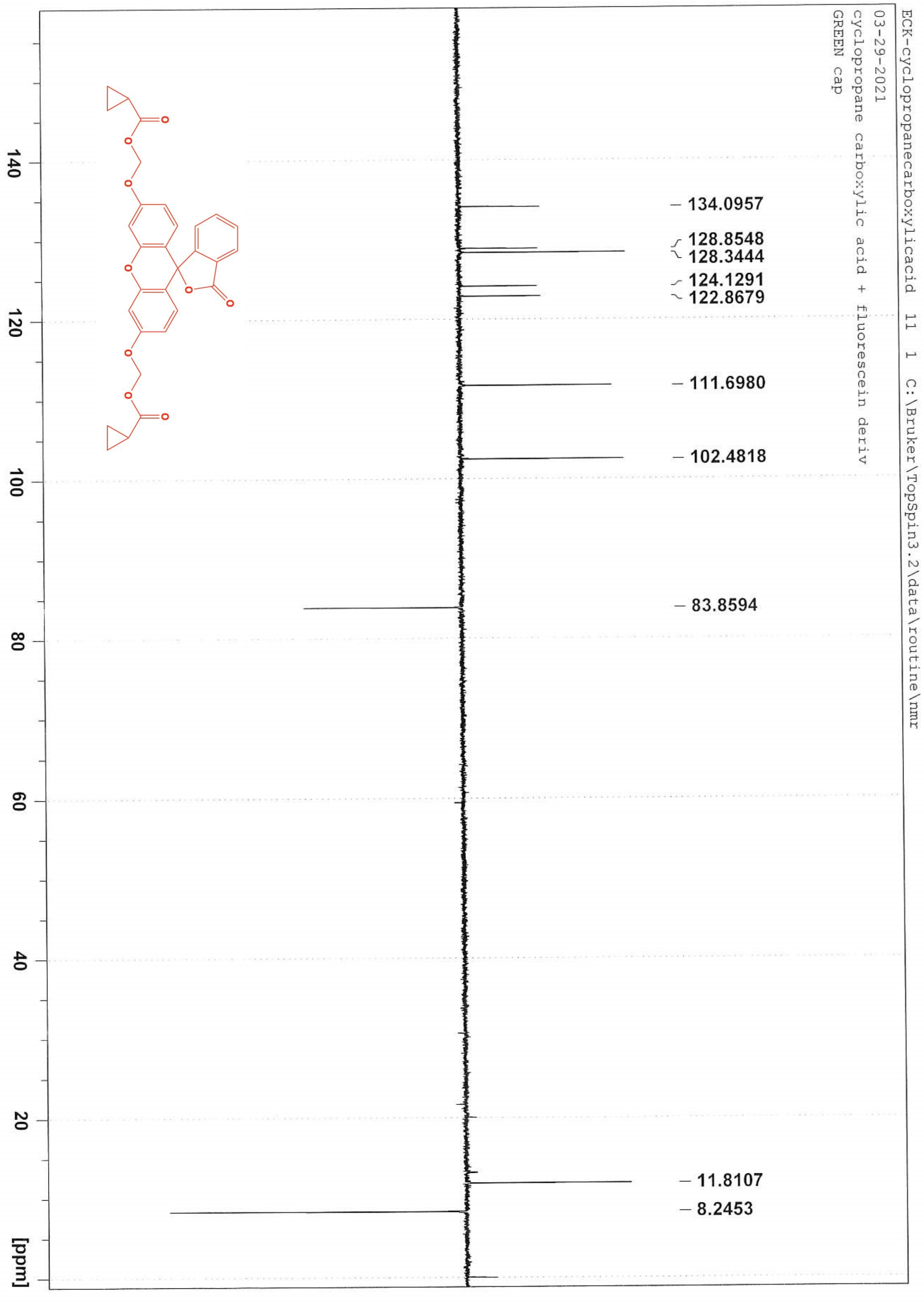

ECK-cyclopropanecarboxylicacid 11 1 C:\Bruker\TopSpin3.2\data\routine\nmr
03-29-2021
cyclopropane carboxylic acid + fluorescein deriv
GREEN cap
134.0957
128.8548
128.3444
124.1291
122.8679
111.6980
102.4818
83.8594
11.8107
8.2453
140
120
100
80
60
40
20
[ppm]

Figure S110

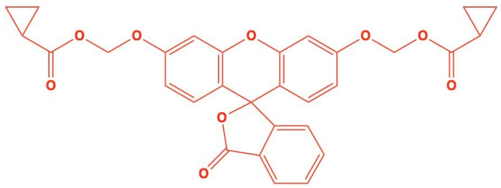

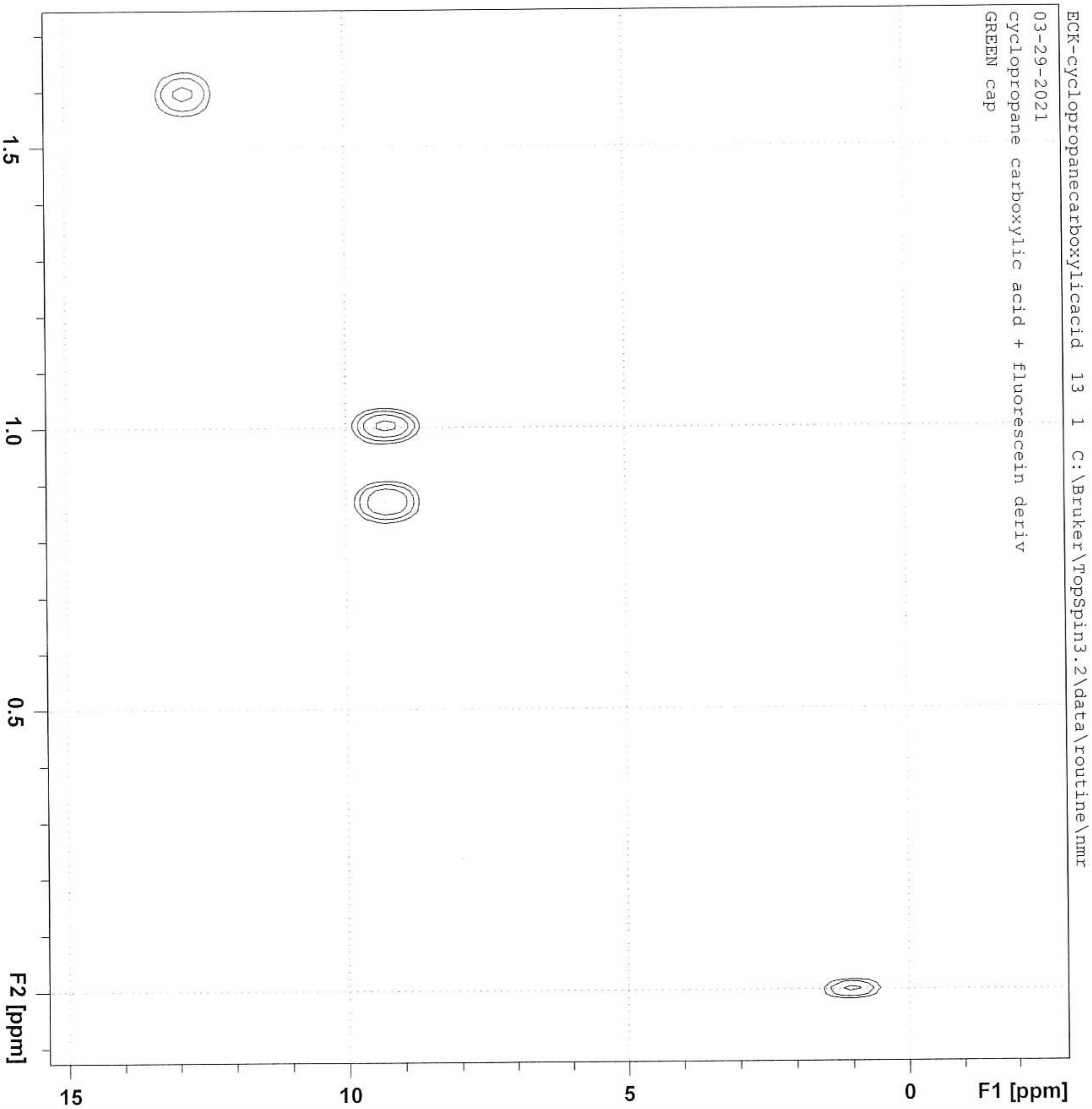
ECK-cyclopropanecarboxylicacid 13 1 C:\Bruker\TopSpin3.2\data\routine\nmr
03-29-2021
cyclopropane carboxylic acid + fluorescein deriv
GREEN cap
F2 [ppm]
F1 [ppm]

Figure S111

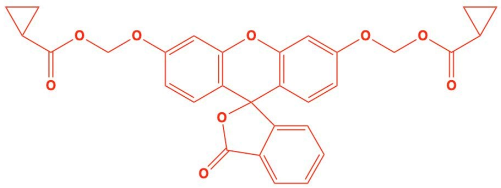

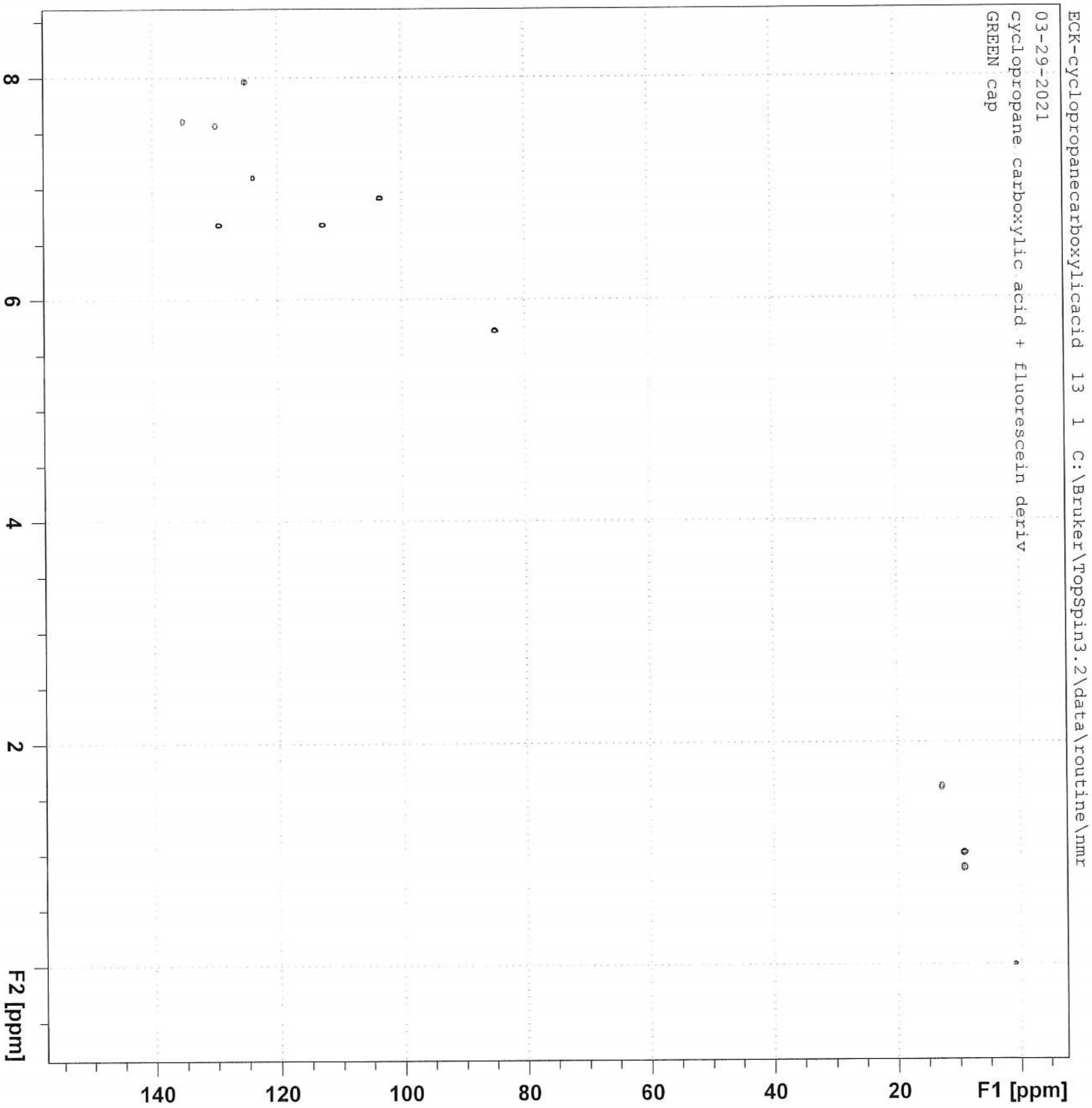

ECK-cyclopropanecarboxylicacid 13 1 C:\Bruker\TopSpin3.2\data\routine\nmr
03-29-2021
cyclopropane carboxylic acid + fluorescein deriv
GREEN cap
F2 [ppm]
F1 [ppm]

Figure S112

Cyclopropene carboxylic acid

1 of 1

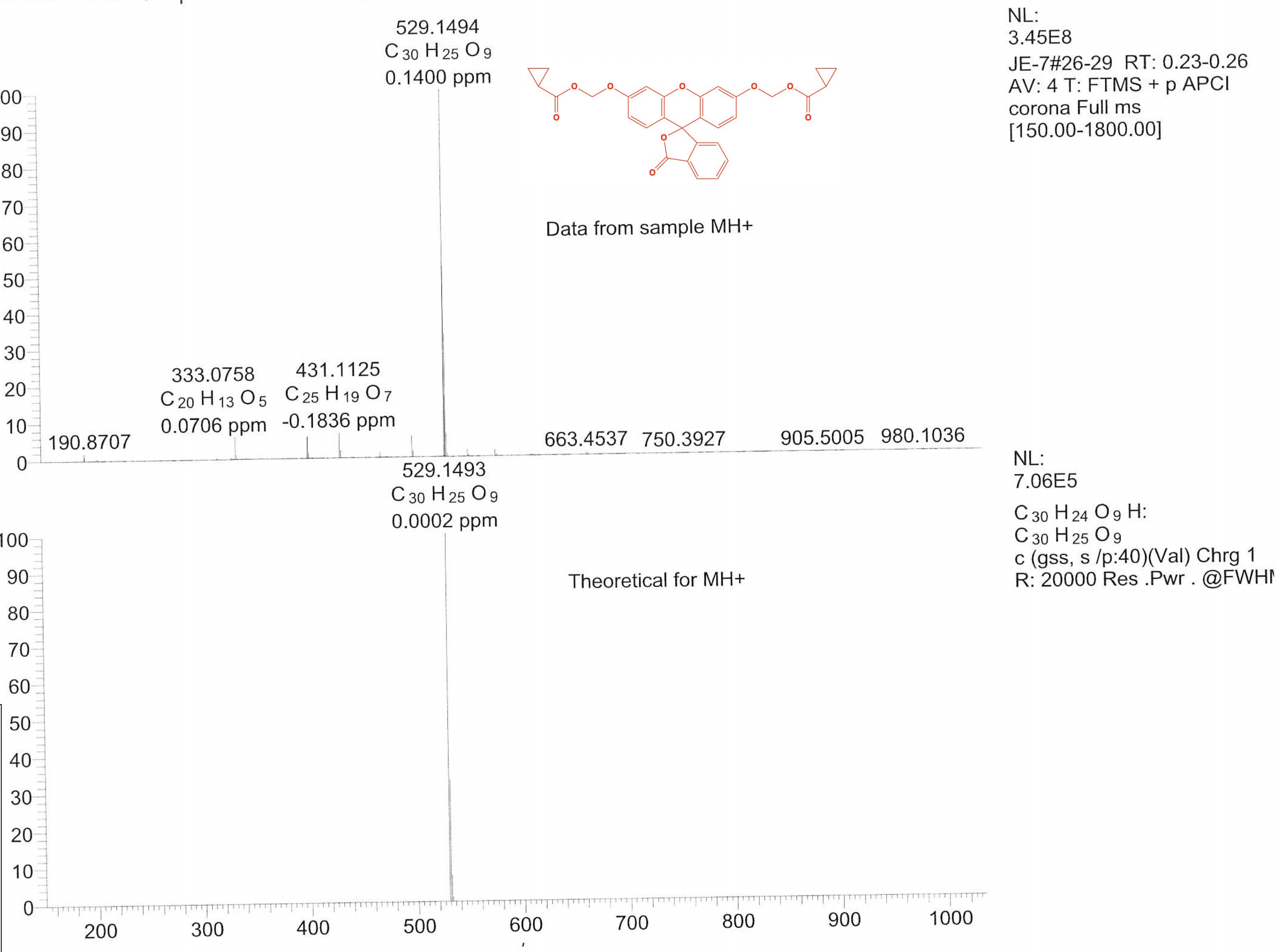

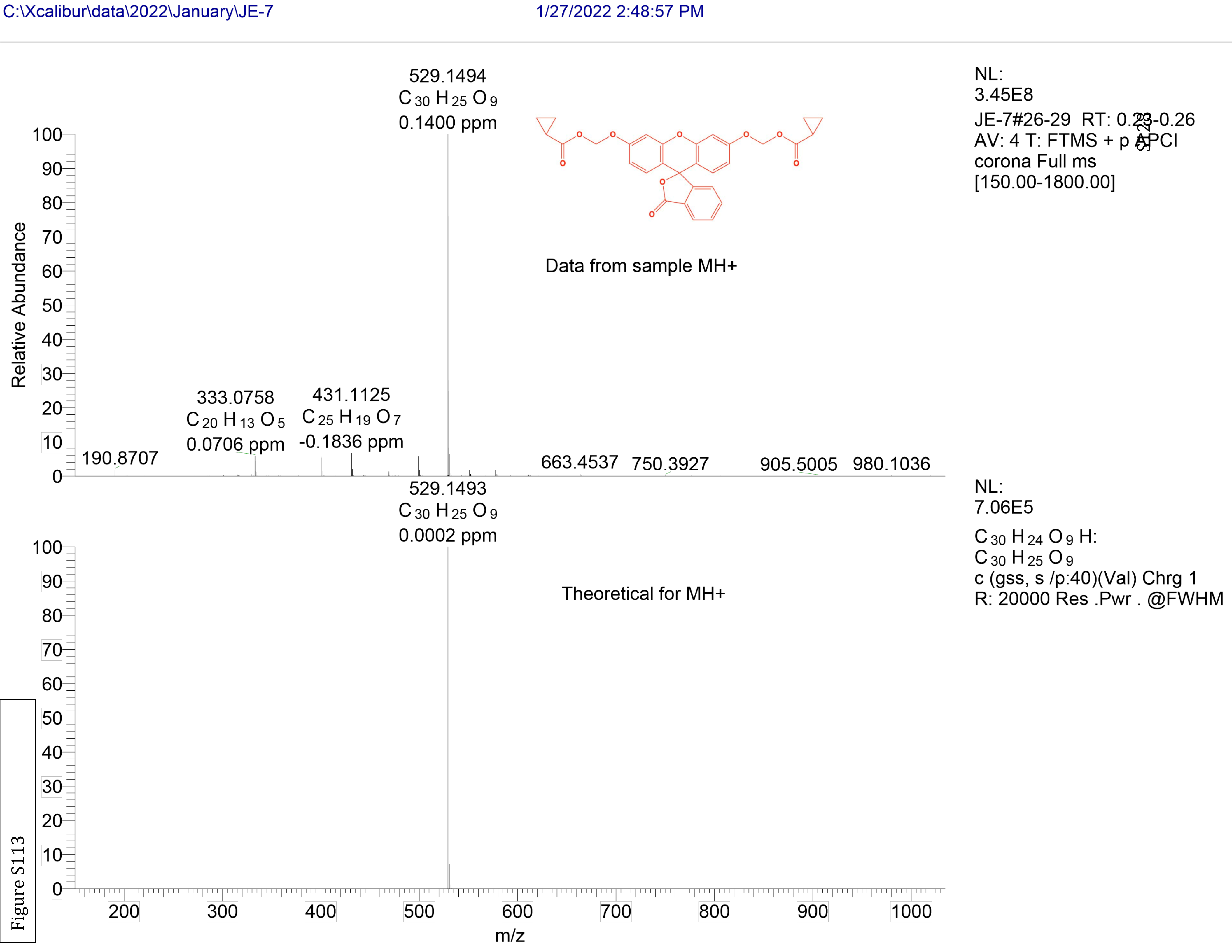
C:\Xcalibur\data\2022\January\JE-7
1/27/2022 2:48:57 PM
529.1494
$C_{30}H_{25}O_9$
0.1400 ppm
Data from sample MH+
NL:
3.45E8
JE-7#26-29 RT: 0.23-0.26
AV: 4 T: FTMS + p APCI
corona Full ms
[150.00-1800.00]
Relative Abundance
190.8707
333.0758
$C_{20}H_{13}O_5$
0.0706 ppm
431.1125
$C_{25}H_{19}O_7$
-0.1836 ppm
663.4537
750.3927
905.5005
980.1036
529.1493
$C_{30}H_{25}O_9$
0.0002 ppm
Theoretical for MH+
NL:
7.06E5
$C_{30}H_{24}O_9$ H:
$C_{30}H_{25}O_9$
c (gss, s /p:40)(Val) Chrg 1
R: 20000 Res .Pwr . @FWHM
m/z

Figure S113

Figure S114

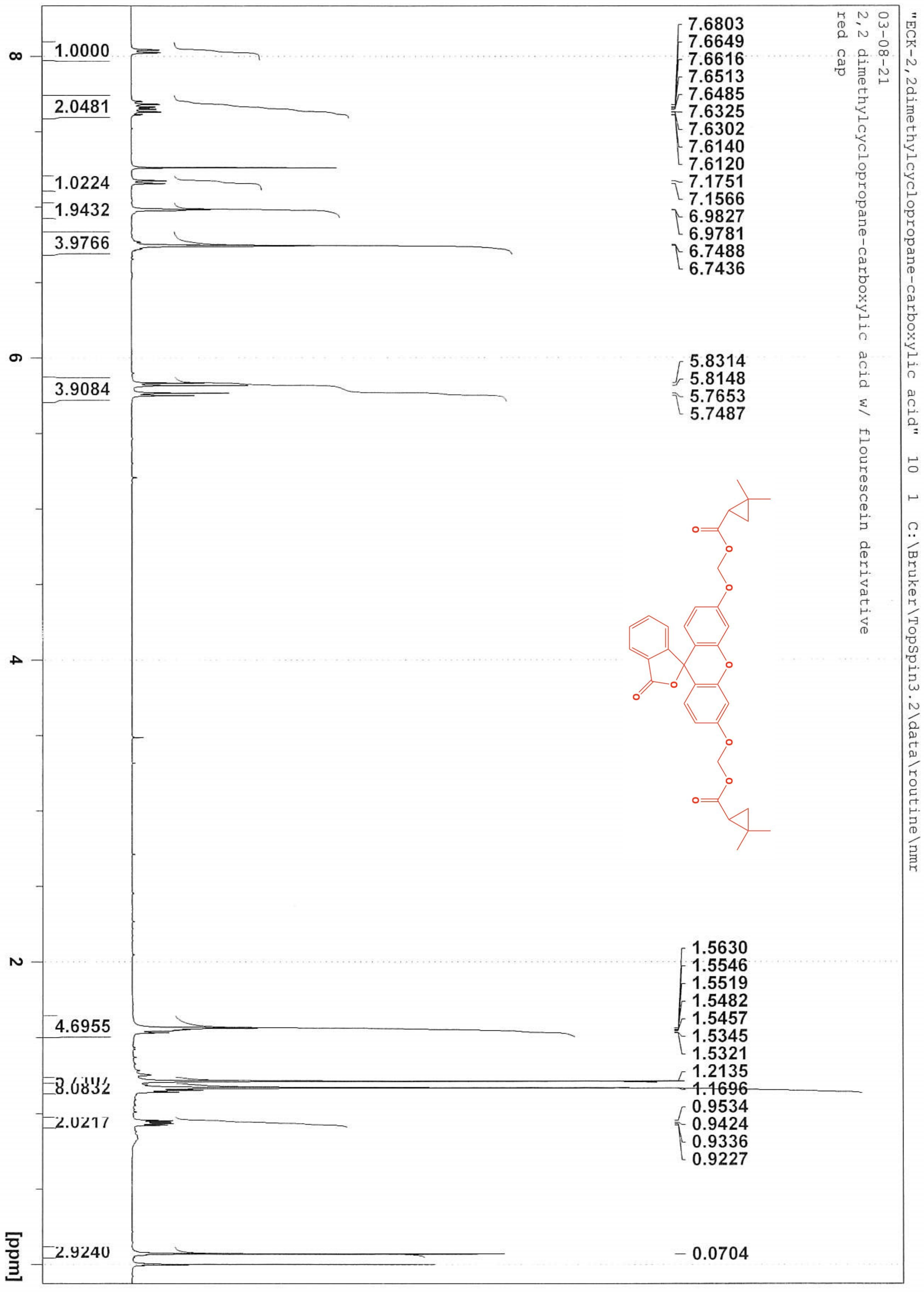

"ECK-2,2dimethylcyclopropane-carboxylic acid" 10 1 C:\Bruker\TopSpin3.2\data\routine\nmr
03-08-21
2,2 dimethylcyclopropane-carboxylic acid w/ flourescein derivative
red cap
7.6803
7.6649
7.6616
7.6513
7.6485
7.6325
7.6302
7.6140
7.6120
7.1751
7.1566
6.9827
6.9781
6.7488
6.7436
5.8314
5.8148
5.7653
5.7487
1.5630
1.5546
1.5519
1.5482
1.5457
1.5345
1.5321
1.2135
1.1696
0.9534
0.9424
0.9336
0.9227
0.0704
1.0000
2.0481
1.0224
1.9432
3.9766
3.9084
4.6955
8.0832
2.0217
2.9240
8
6
4
2
[ppm]

Figure S115

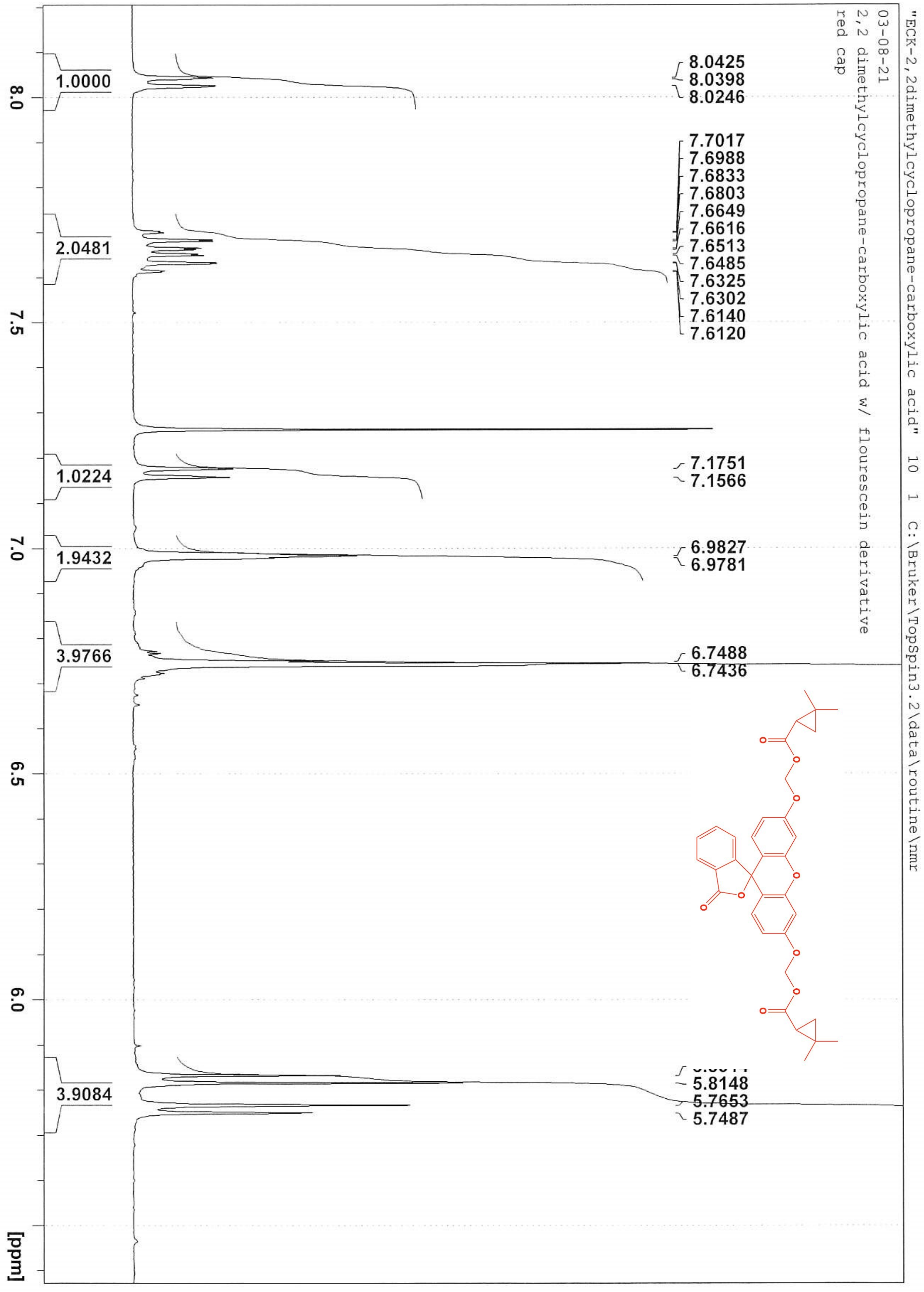

"ECK-2,2dimethylcyclopropane-carboxylic acid" 10 1 C:\Bruker\TopSpin3.2\data\routine\nmr
03-08-21
2,2 dimethylcyclopropane-carboxylic acid w/ flourescein derivative
red cap
8.0425
8.0398
8.0246
7.7017
7.6988
7.6833
7.6803
7.6649
7.6616
7.6513
7.6485
7.6325
7.6302
7.6140
7.6120
7.1751
7.1566
6.9827
6.9781
6.7488
6.7436
5.8148
5.7653
5.7487
1.0000
2.0481
1.0224
1.9432
3.9766
3.9084
8.0
7.5
7.0
6.5
6.0
[ppm]

Figure S116

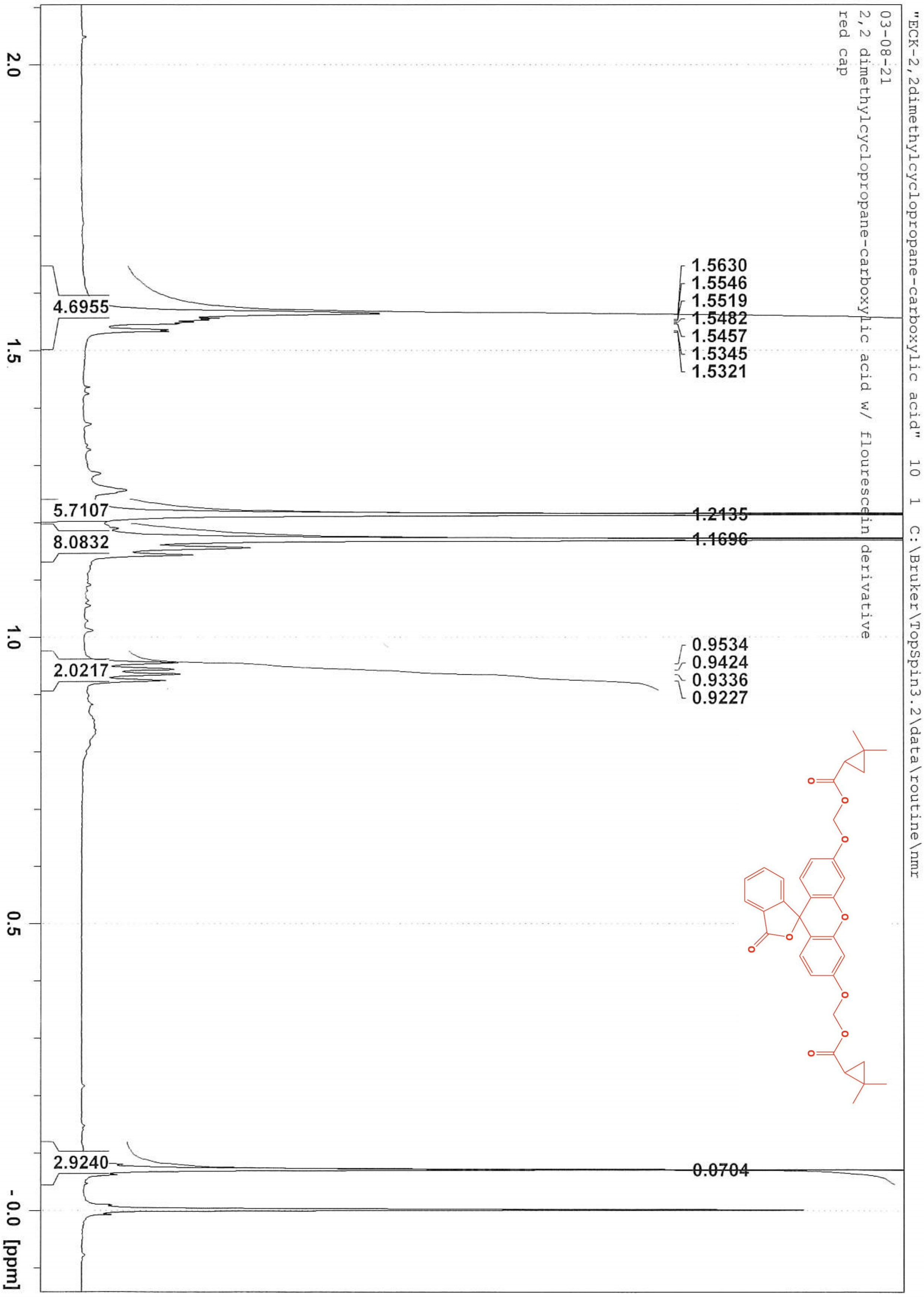
"ECK-2,2dimethylcyclopropane-carboxylic acid" 10 1 C:\Bruker\TopSpin3.2\data\routine\nmr
03-08-21
2,2 dimethylcyclopropane-carboxylic acid w/ flourescein derivative
red cap
1.5630
1.5546
1.5519
1.5482
1.5457
1.5345
1.5321
1.2135
1.1696
0.9534
0.9424
0.9336
0.9227
0.0704
4.6955
5.7107
8.0832
2.0217
2.9240
2.0
1.5
1.0
0.5
- 0.0
[ppm]

Figure S117

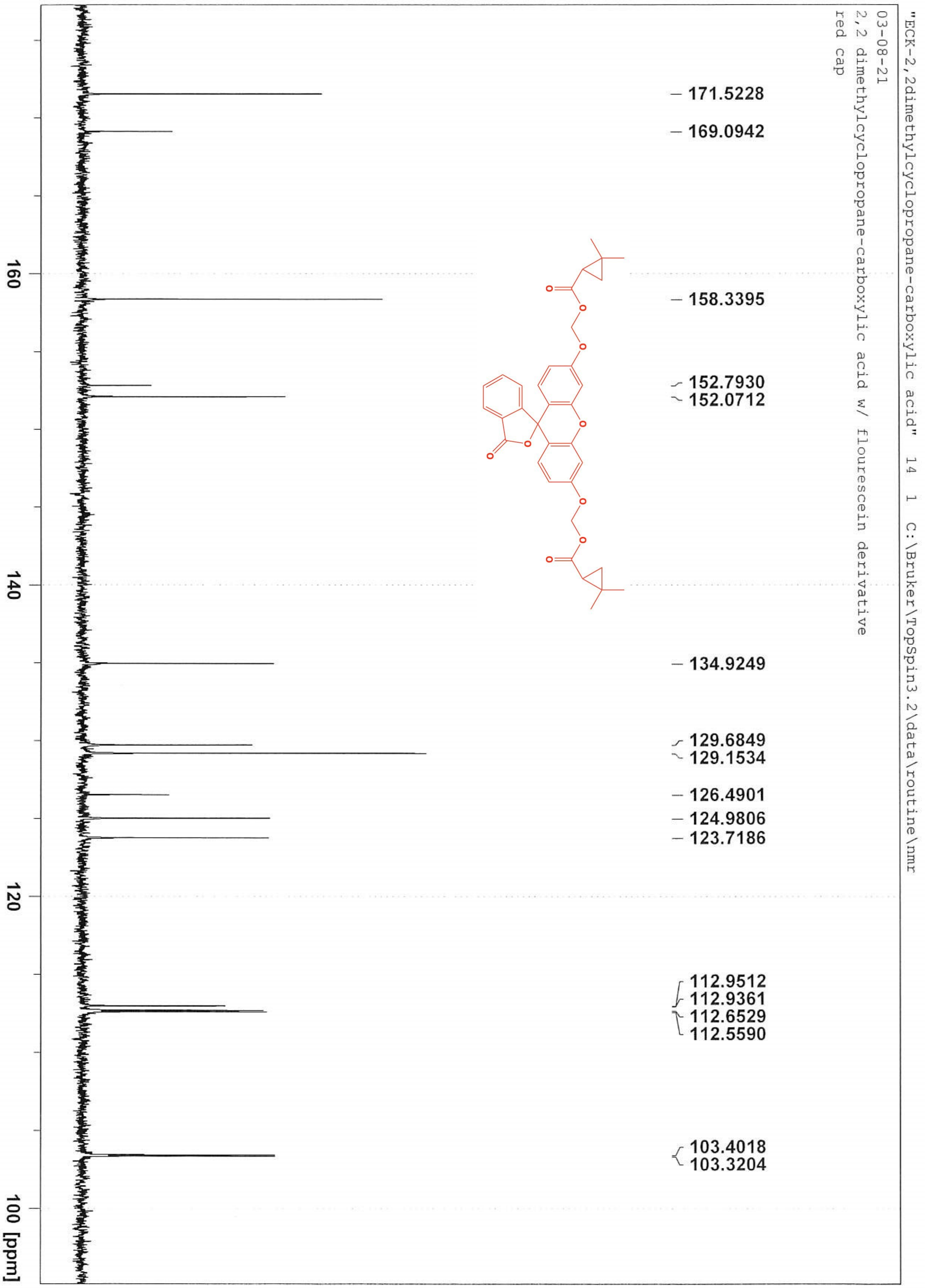
"ECK-2,2dimethylcyclopropane-carboxylic acid" 14 1 C:\Bruker\TopSpin3.2\data\routine\nmr
03-08-21
2,2 dimethylcyclopropane-carboxylic acid w/ flourescein derivative
red cap
171.5228
169.0942
158.3395
152.7930
152.0712
134.9249
129.6849
129.1534
126.4901
124.9806
123.7186
112.9512
112.9361
112.6529
112.5590
103.4018
103.3204
160
140
120
100 [ppm]

Figure S118

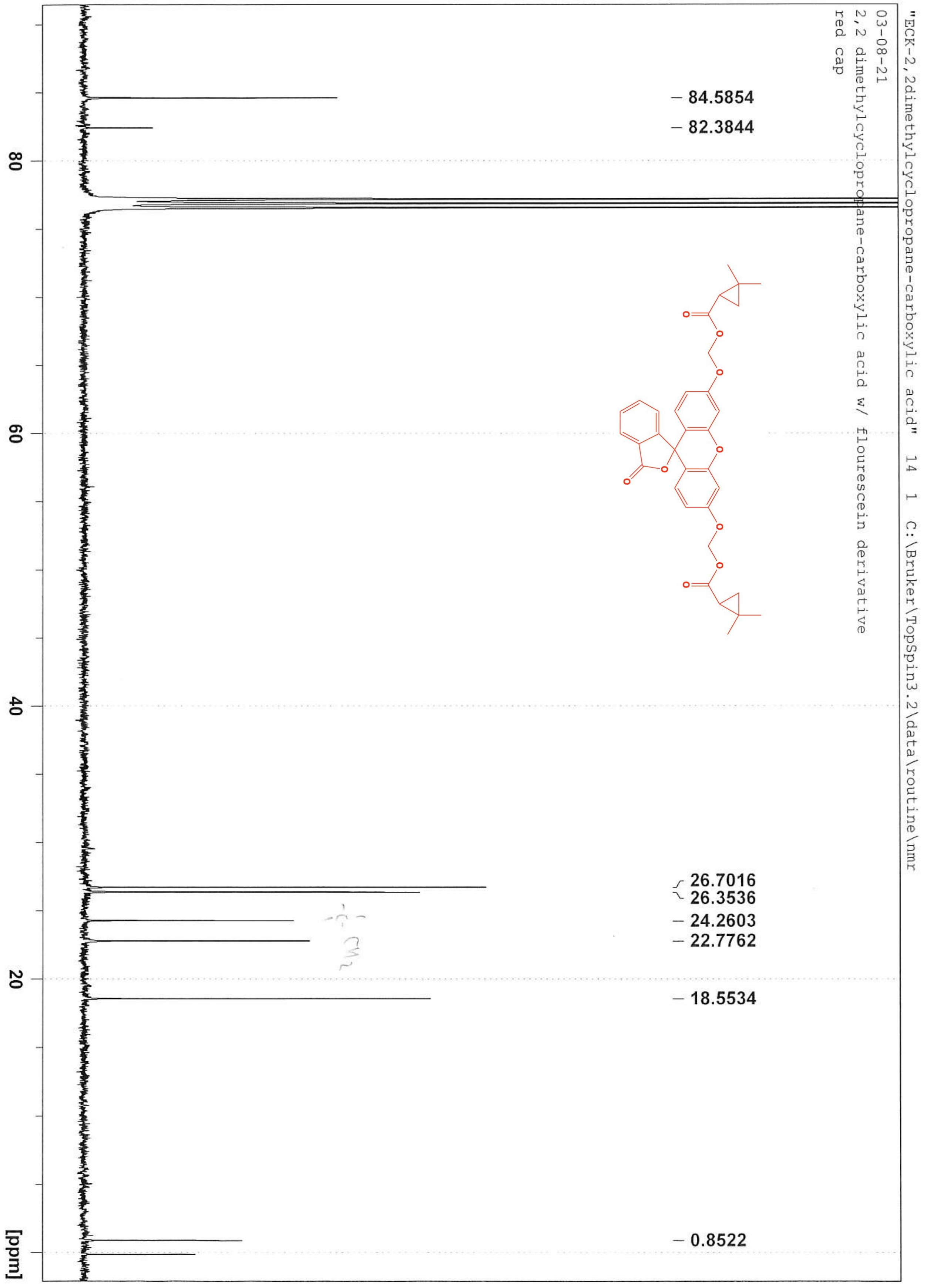

"ECK-2,2dimethylcyclopropane-carboxylic acid" 14 1 C:\Bruker\TopSpin3.2\data\routine\nmr
03-08-21
2,2 dimethylcyclopropane-carboxylic acid w/ flourescein derivative
red cap
84.5854
82.3844
26.7016
26.3536
24.2603
22.7762
18.5534
0.8522
80
60
40
20
[ppm]

Figure S119

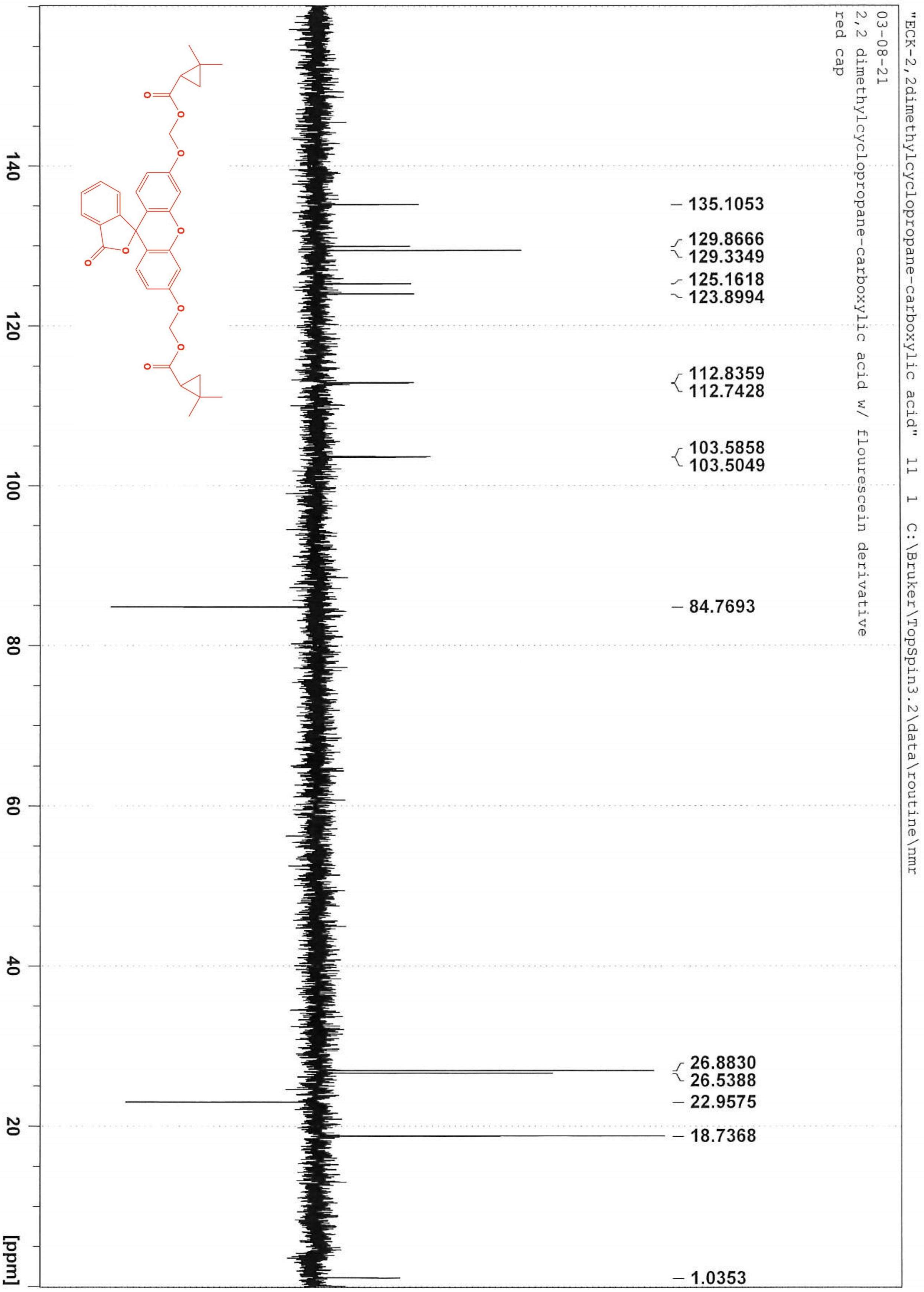
"ECK-2,2dimethylcyclopropane-carboxylic acid" 11 1 C:\Bruker\TopSpin3.2\data\routine\nmr
03-08-21
2,2 dimethylcyclopropane-carboxylic acid w/ flourescein derivative
red cap
135.1053
129.8666
129.3349
125.1618
123.8994
112.8359
112.7428
103.5858
103.5049
84.7693
26.8830
26.5388
22.9575
18.7368
1.0353
140
120
100
80
60
40
20
[ppm]

Figure S120

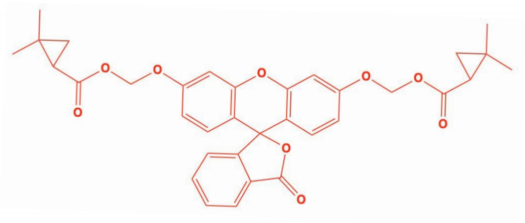

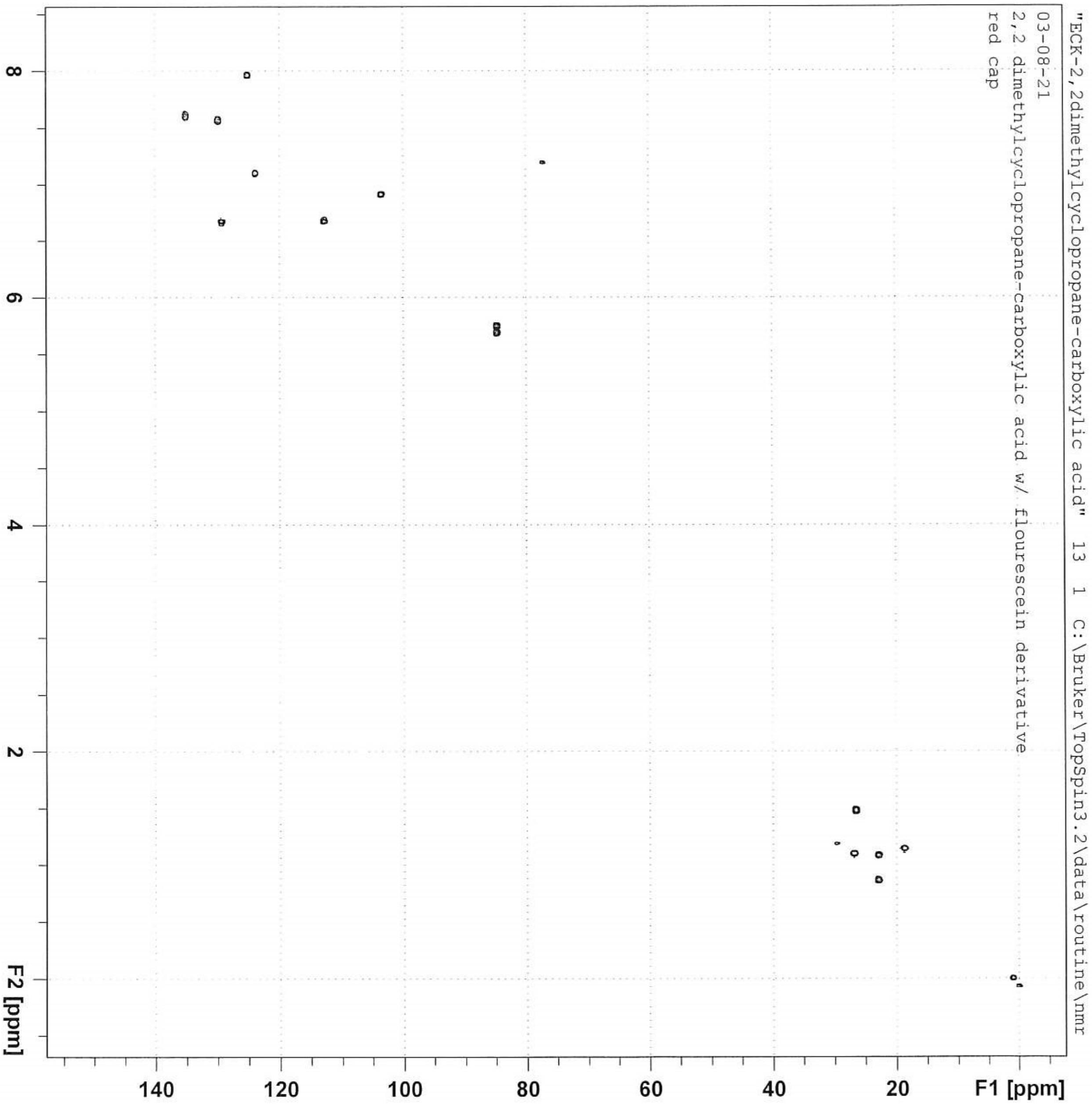
"ECK-2,2dimethylcyclopropane-carboxylic acid" 13 1 C:\Bruker\TopSpin3.2\data\routine\nmr
03-08-21
2,2 dimethylcyclopropane-carboxylic acid w/ flourescein derivative
red cap
F1 [ppm]
F2 [ppm]

Figure S121

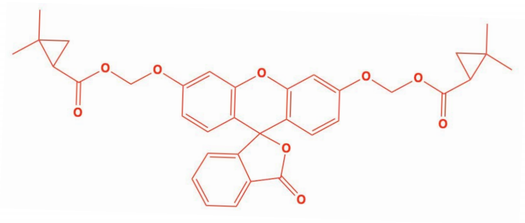

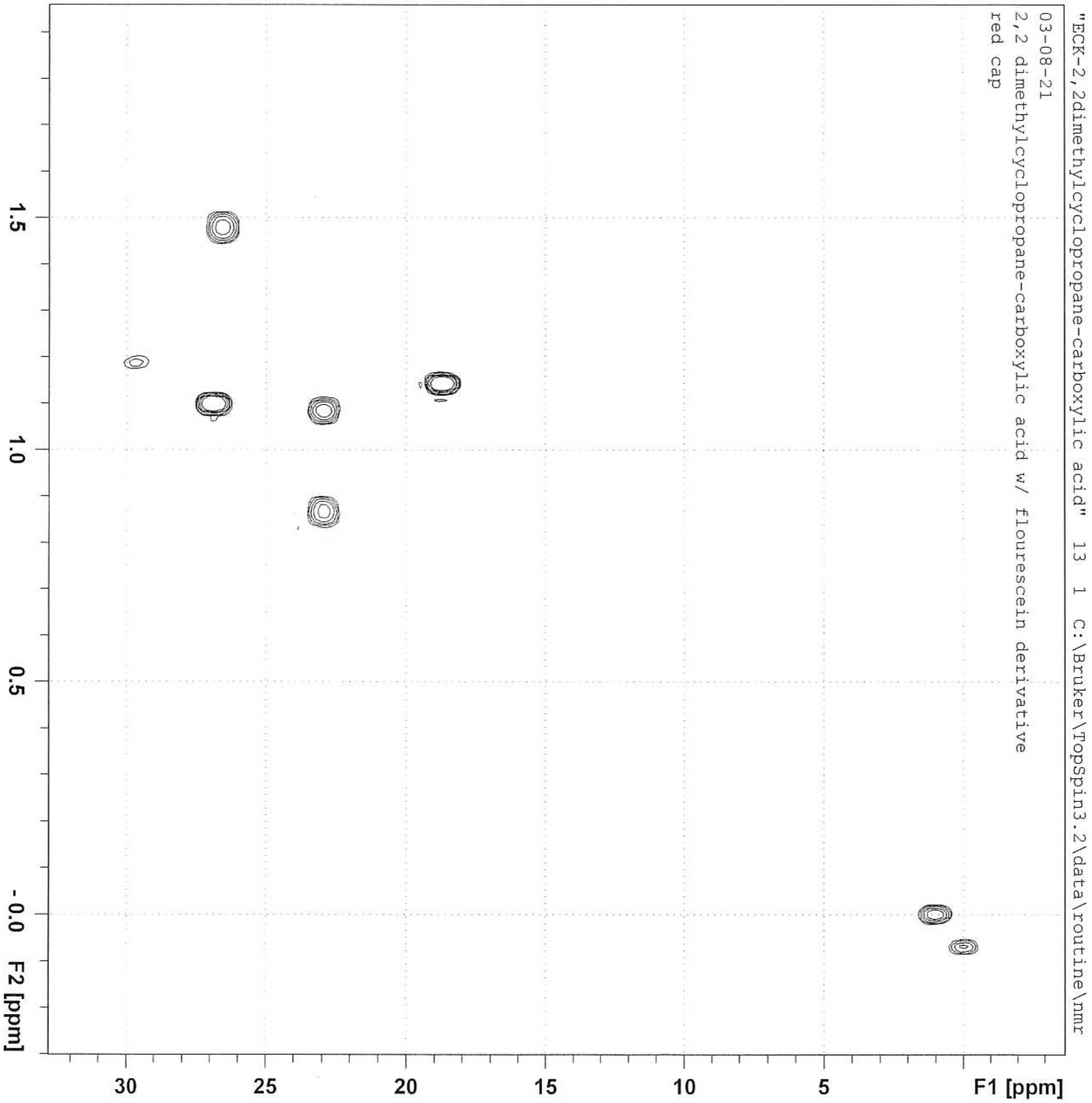
"ECK-2,2dimethylcyclopropane-carboxylic acid" 13 1 C:\Bruker\TopSpin3.2\data\routine\nmr
03-08-21
2,2 dimethylcyclopropane-carboxylic acid w/ flourescein derivative
red cap
F2 [ppm]
F1 [ppm]

Figure S122

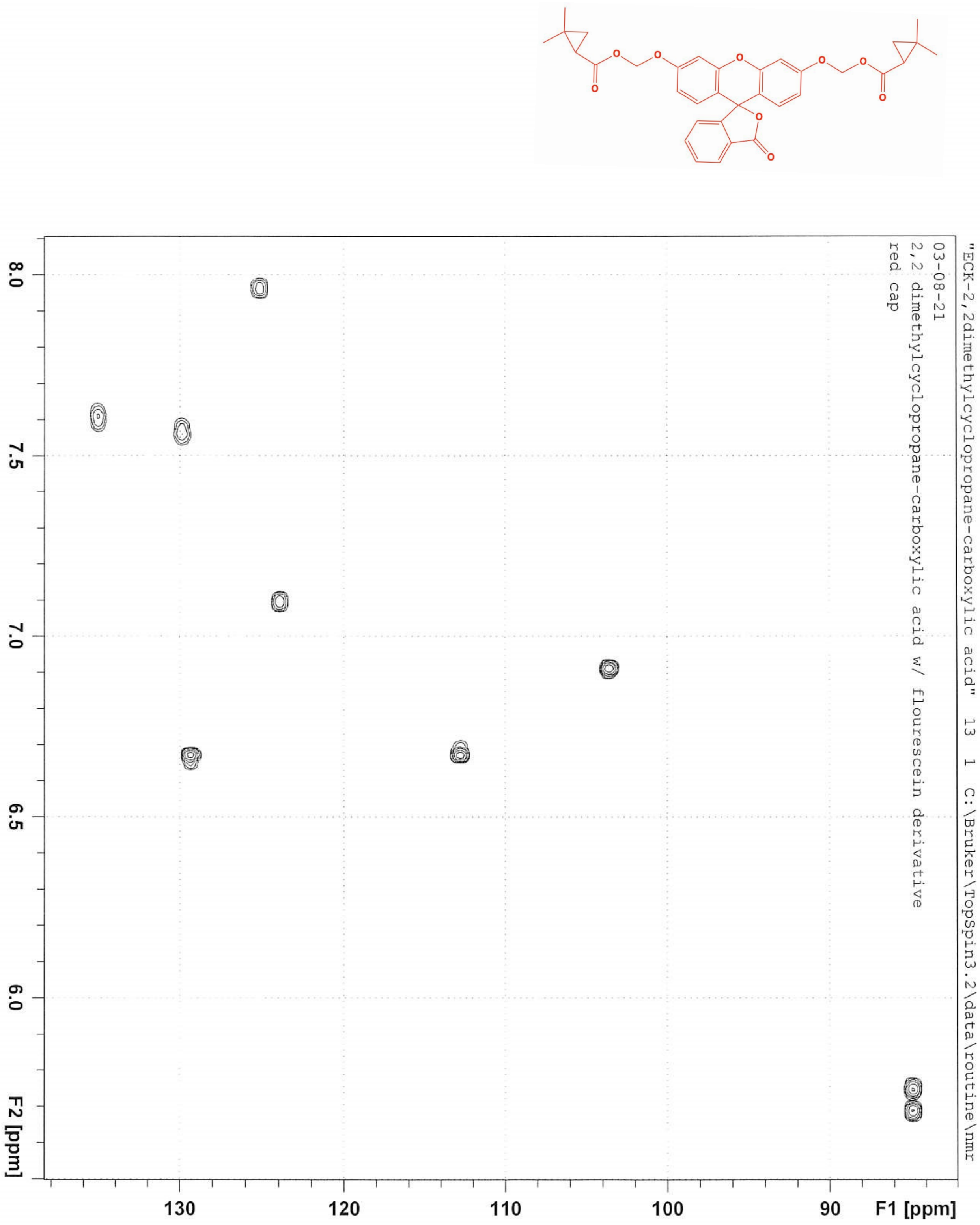
"ECK-2,2dimethylcyclopropane-carboxylic acid" 13 1 C:\Bruker\TopSpin3.2\data\routine\nmr
03-08-21
2,2 dimethylcyclopropane-carboxylic acid w/ flourescein derivative
red cap
F2 [ppm]
8.0
7.5
7.0
6.5
6.0
F1 [ppm]
130
120
110
100
90

Figure S123

2.2-dimethylcyclopropane carboxylic acid

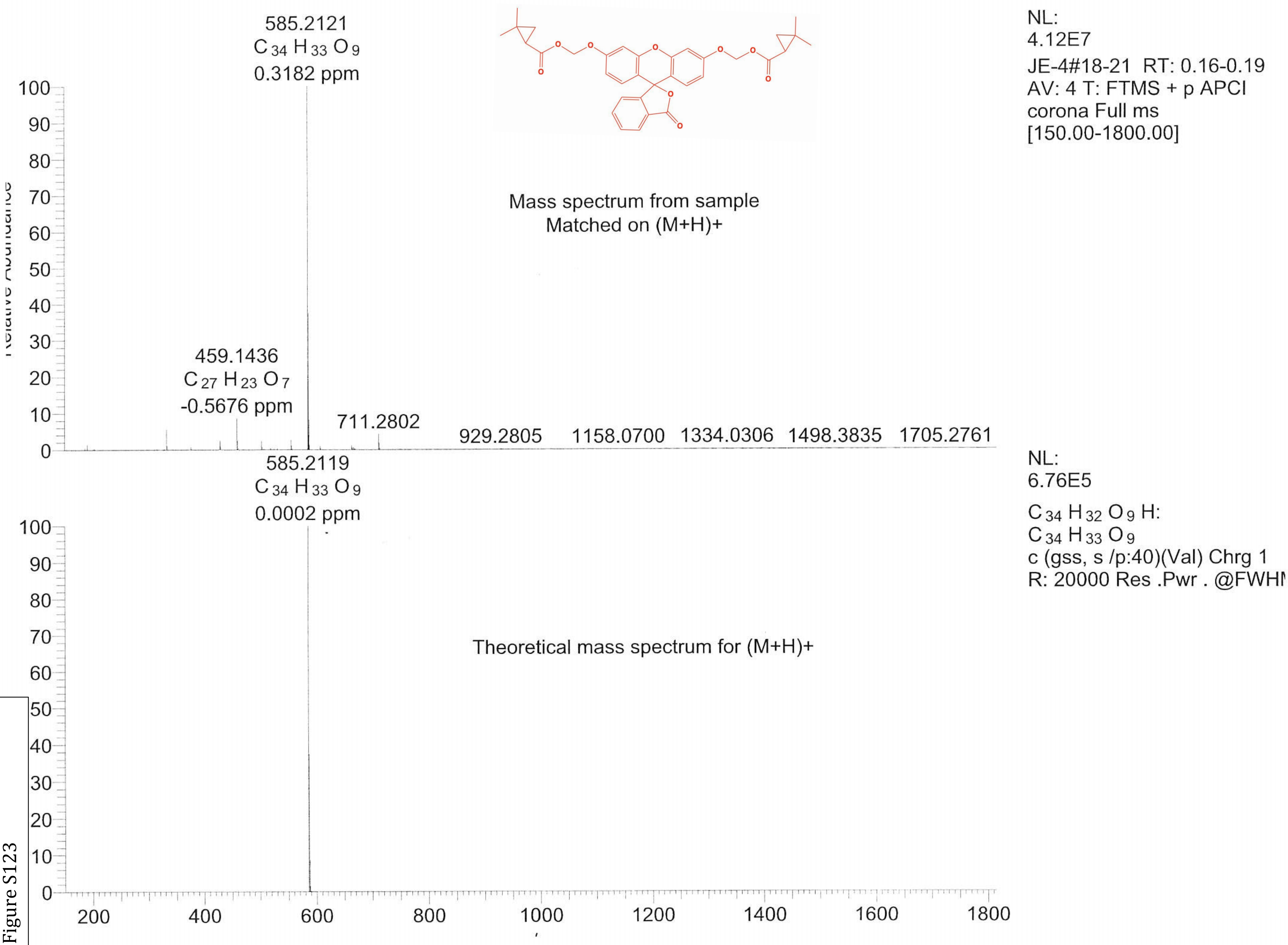

2,2-dimethyl cyclopropane carbotylic acid

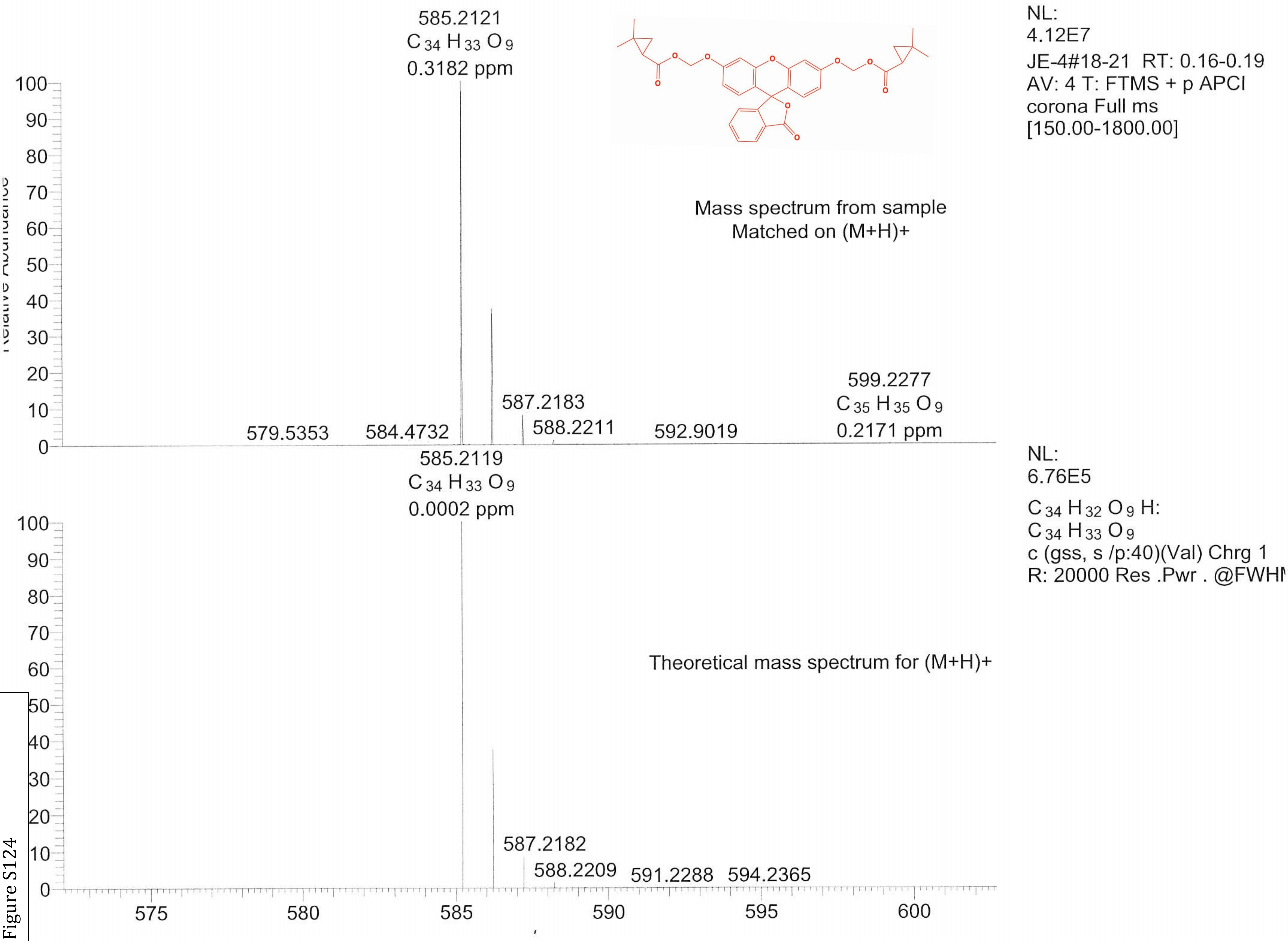

Figure S124

C:\Xcalibur\data\2022\January\JE-4 1/27/2022 2:39:50 PM

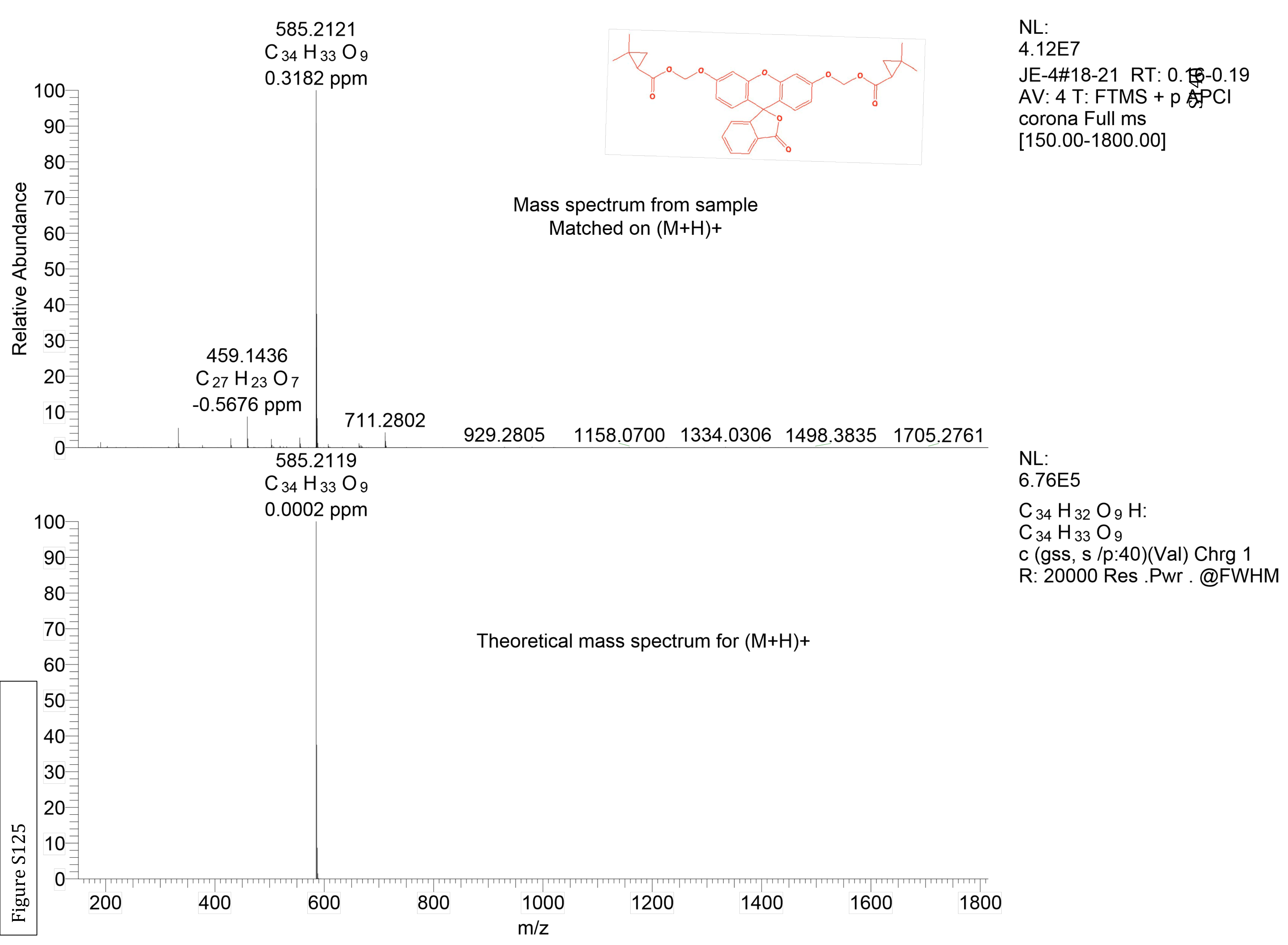

Figure S125

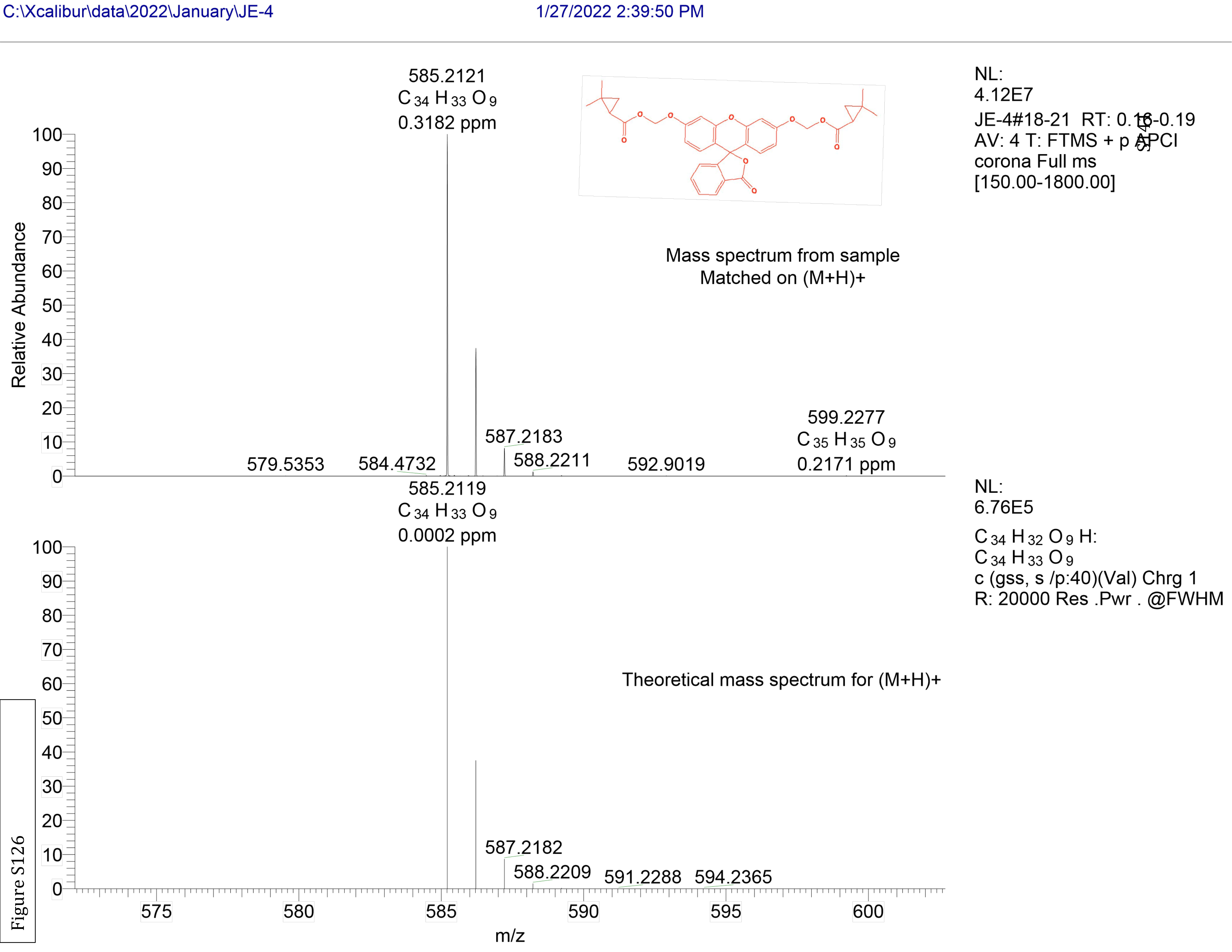
C:\Xcalibur\data\2022\January\JE-4
1/27/2022 2:39:50 PM
585.2121
$C_{34}H_{33}O_9$
0.3182 ppm
NL:
4.12E7
JE-4#18-21 RT: 0.16-0.19
AV: 4 T: FTMS + p APCI
corona Full ms
[150.00-1800.00]
Mass spectrum from sample
Matched on (M+H)+
Relative Abundance
579.5353
584.4732
587.2183
588.2211
592.9019
599.2277
$C_{35}H_{35}O_9$
0.2171 ppm
585.2119
$C_{34}H_{33}O_9$
0.0002 ppm
NL:
6.76E5
$C_{34}H_{32}O_9$ H:
$C_{34}H_{33}O_9$
c (gss, s /p:40)(Val) Chrg 1
R: 20000 Res .Pwr . @FWHM
Theoretical mass spectrum for (M+H)+
587.2182
588.2209
591.2288
594.2365
m/z

Figure S126

Figure S127

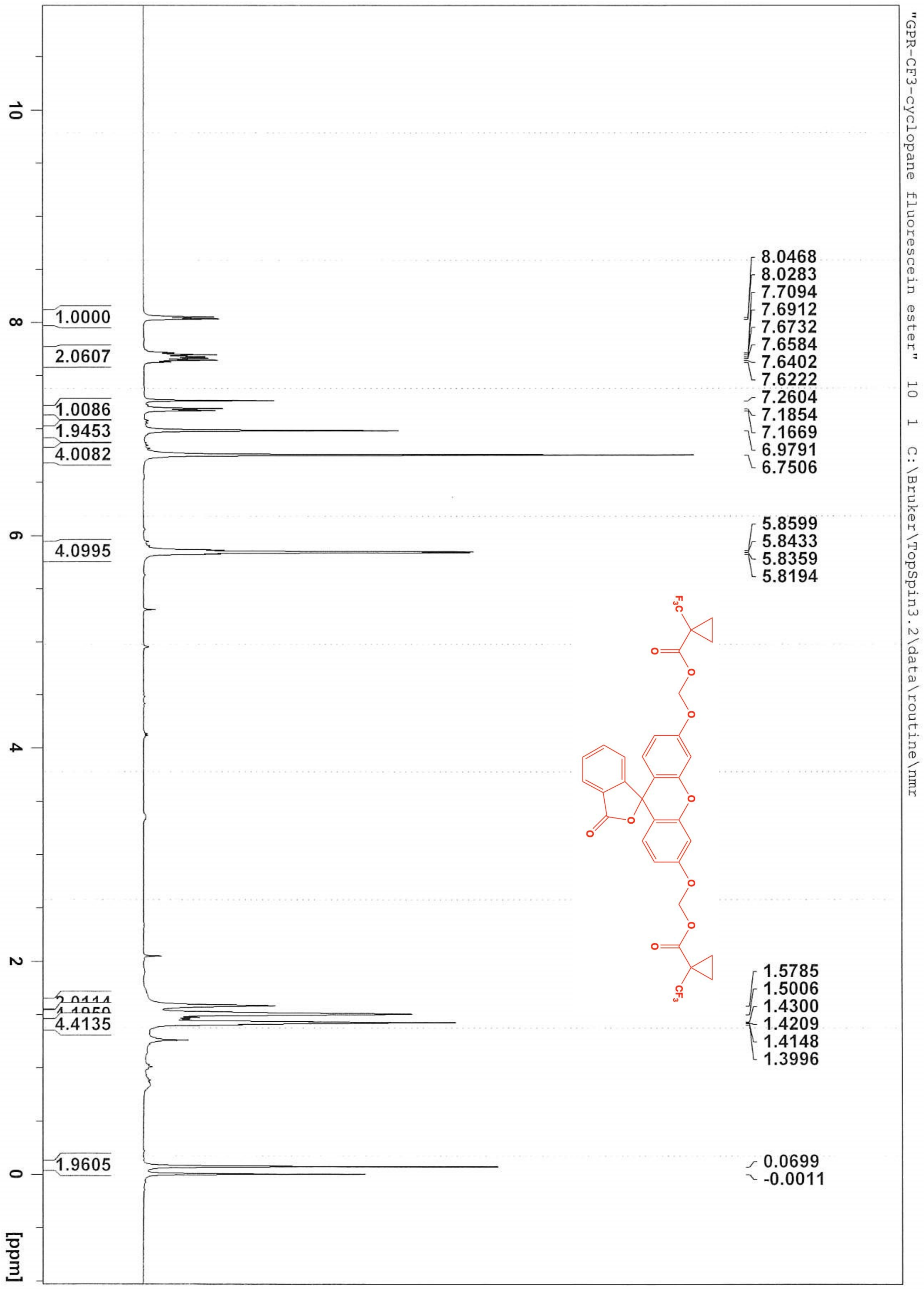

"GPR-CF3-cyclopane fluorescein ester" 10 1 C:\Bruker\TopSpin3.2\data\routine\nmr
8.0468
8.0283
7.7094
7.6912
7.6732
7.6584
7.6402
7.6222
7.2604
7.1854
7.1669
6.9791
6.7506
5.8599
5.8433
5.8359
5.8194
1.5785
1.5006
1.4300
1.4209
1.4148
1.3996
0.0699
-0.0011
1.0000
2.0607
1.0086
1.9453
4.0082
4.0995
4.4135
1.9605
10
8
6
4
2
0
[ppm]

Figure S128

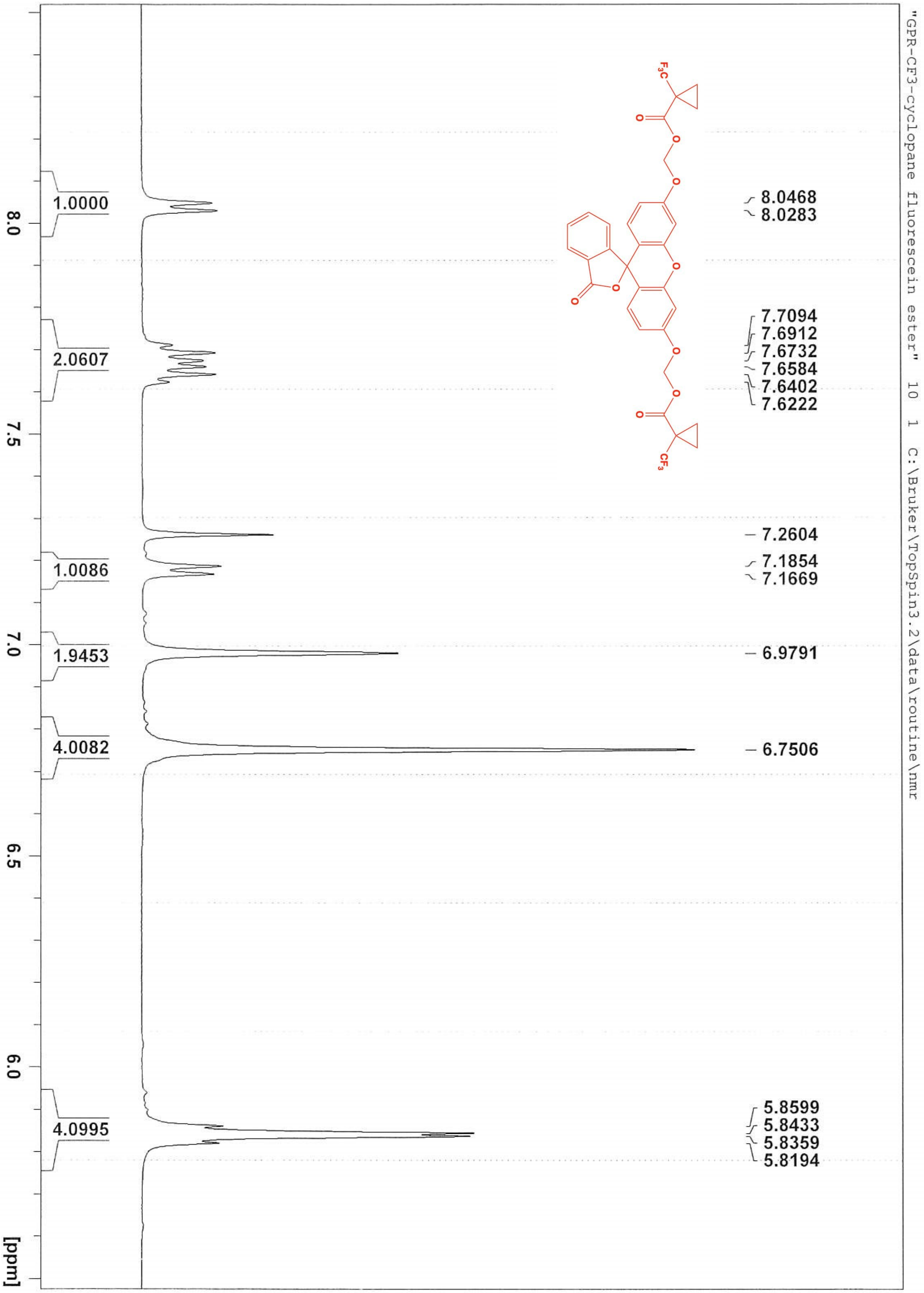

"GPR-CF3-cyclopane fluorescein ester" 10 1 C:\Bruker\TopSpin3.2\data\routine\nmr
8.0468
8.0283
7.7094
7.6912
7.6732
7.6584
7.6402
7.6222
7.2604
7.1854
7.1669
6.9791
6.7506
5.8599
5.8433
5.8359
5.8194
1.0000
2.0607
1.0086
1.9453
4.0082
4.0995
8.0
7.5
7.0
6.5
6.0
[ppm]

Figure S129

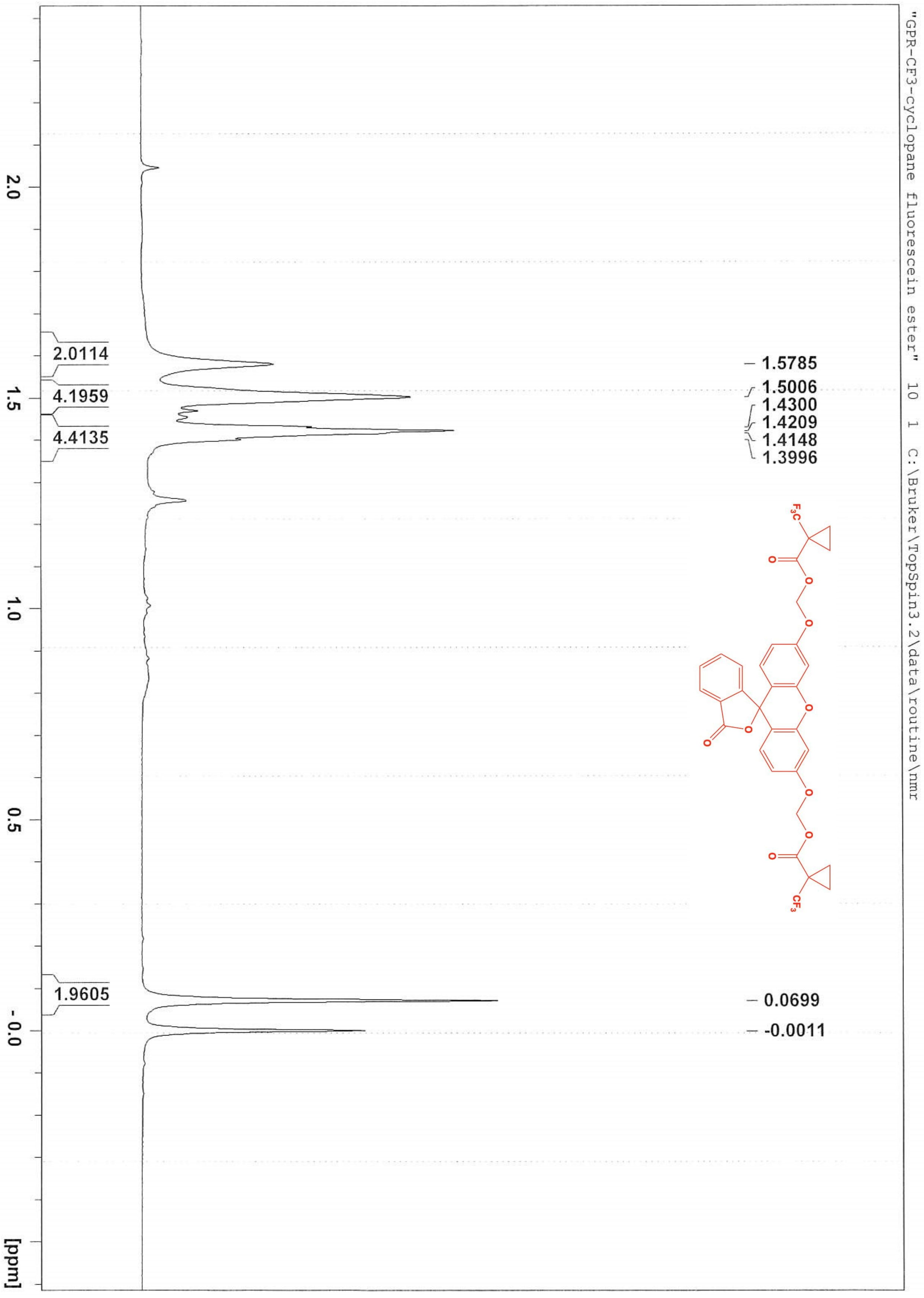

"GPR-CF3-cyclopane fluorescein ester" 10 1 C:\Bruker\TopSpin3.2\data\routine\nmr
1.5785
1.5006
1.4300
1.4209
1.4148
1.3996
0.0699
-0.0011
2.0114
4.1959
4.4135
1.9605
2.0
1.5
1.0
0.5
-0.0
[ppm]

Figure S130

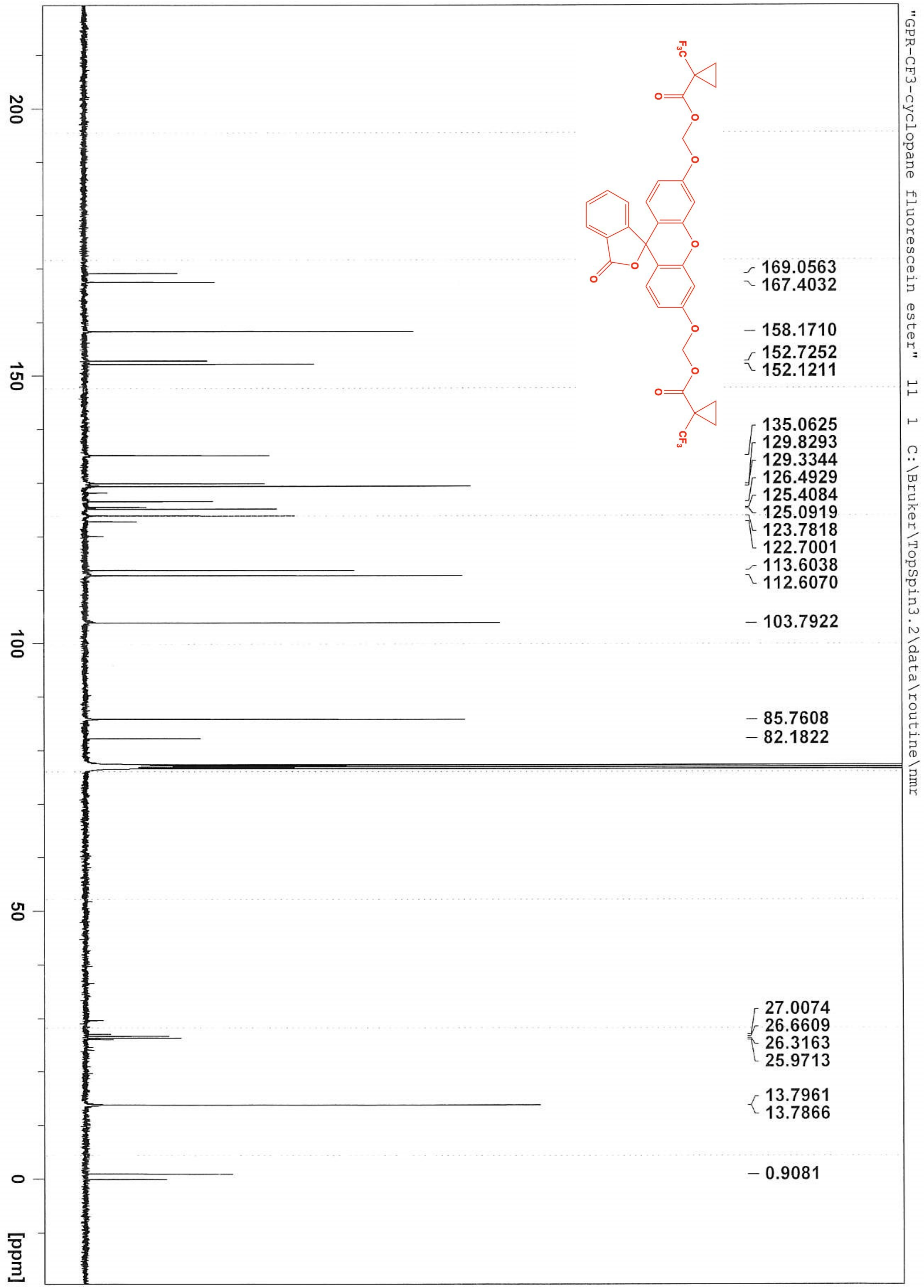
"GPR-CF3-cyclopane fluorescein ester" 11 1 C:\Bruker\TopSpin3.2\data\routine\nmr
169.0563
167.4032
158.1710
152.7252
152.1211
135.0625
129.8293
129.3344
126.4929
125.4084
125.0919
123.7818
122.7001
113.6038
112.6070
103.7922
85.7608
82.1822
27.0074
26.6609
26.3163
25.9713
13.7961
13.7866
0.9081
200
150
100
50
0
[ppm]

Figure S131

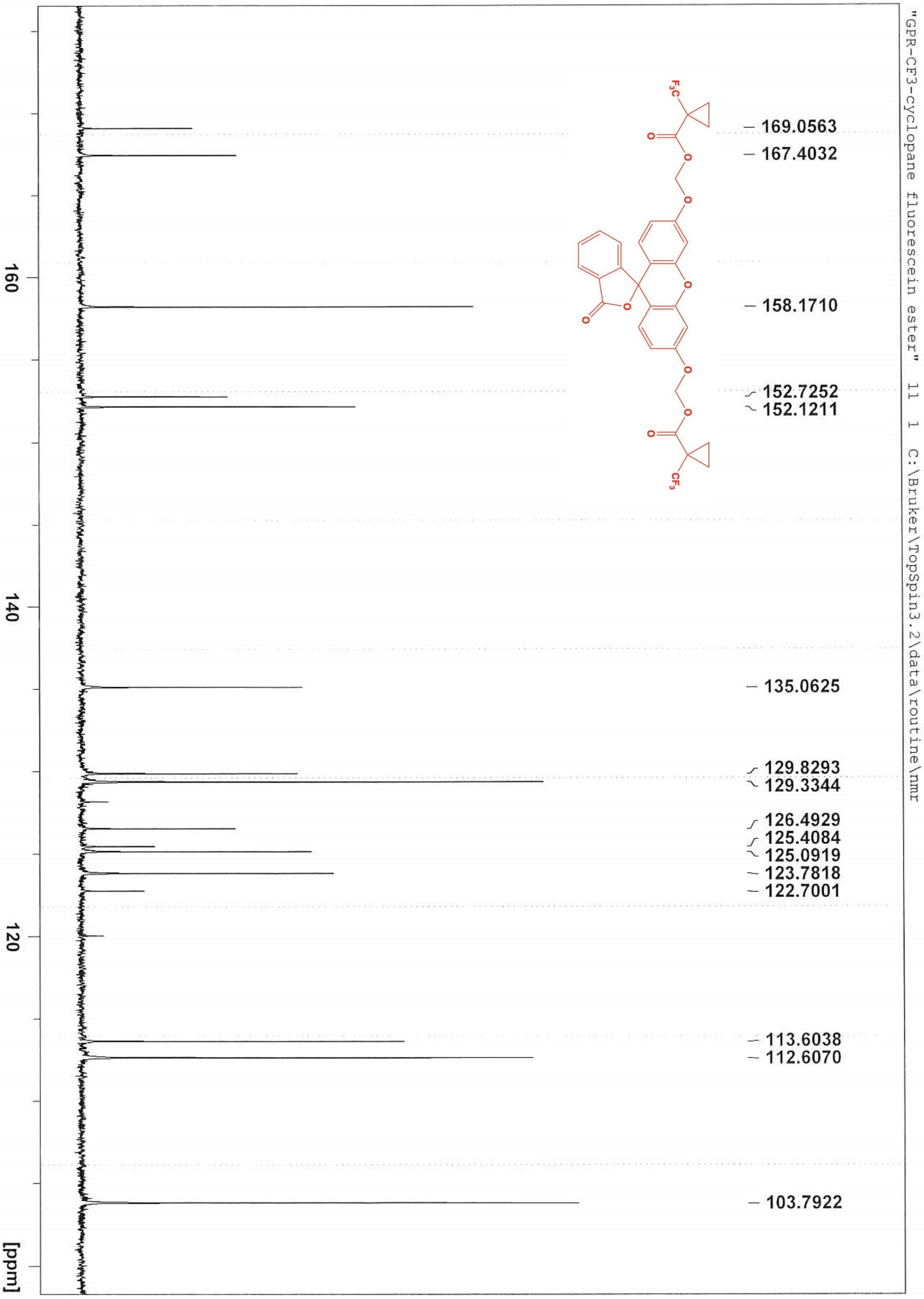
"GPR-CF3-cyclopane fluorescein ester" 11 1 C:\Bruker\TopSpin3.2\data\routine\nmr
169.0563
167.4032
158.1710
152.7252
152.1211
135.0625
129.8293
129.3344
126.4929
125.4084
125.0919
123.7818
122.7001
113.6038
112.6070
103.7922
160
140
120
[ppm]
F3C
CF3
O

Figure S132

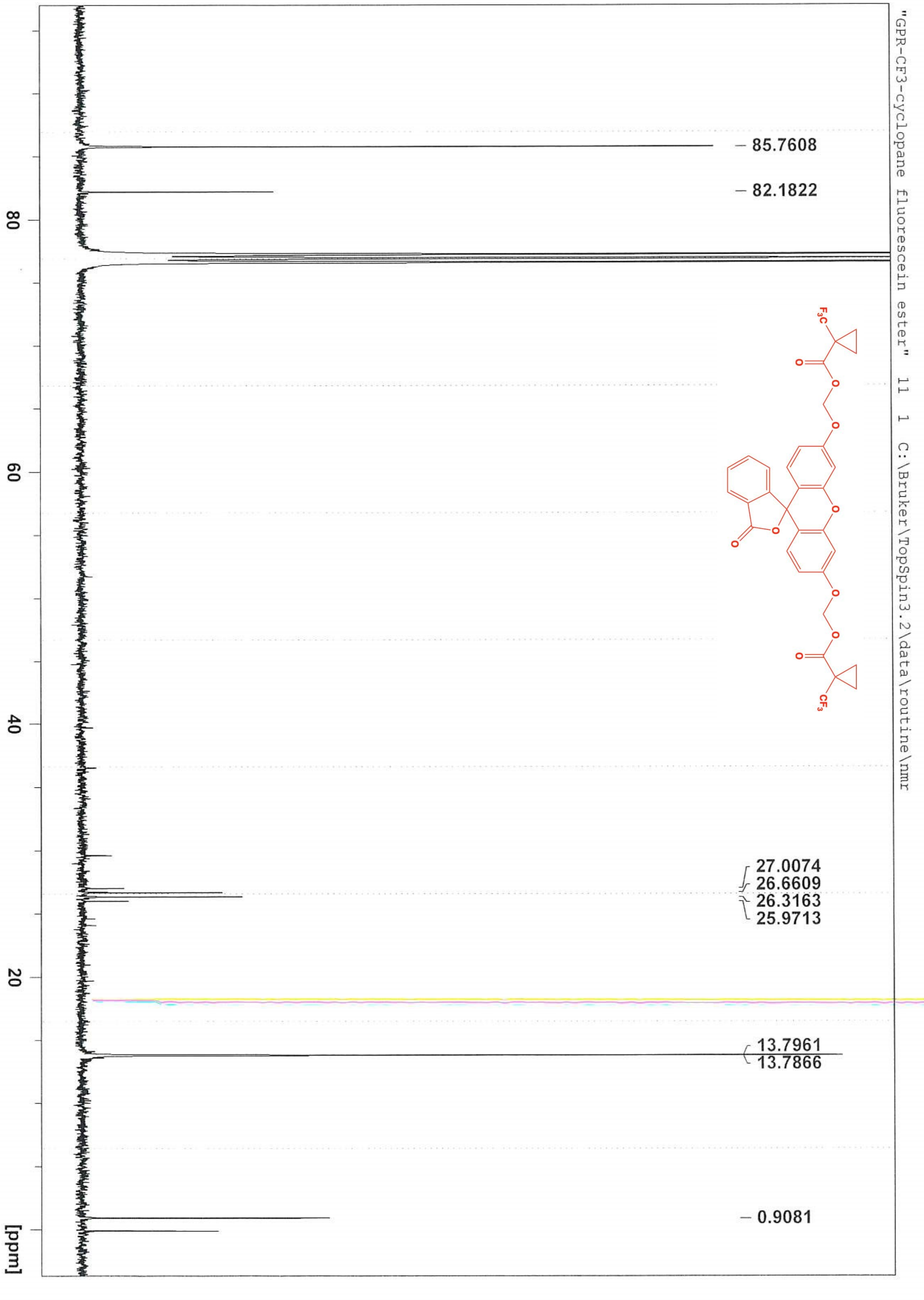
"GPR-CF3-cyclopane fluorescein ester" 11 1 C:\Bruker\TopSpin3.2\data\routine\nmr
85.7608
82.1822
27.0074
26.6609
26.3163
25.9713
13.7961
13.7866
0.9081
80
60
40
20
[ppm]
F_3C
CF_3

Figure S133

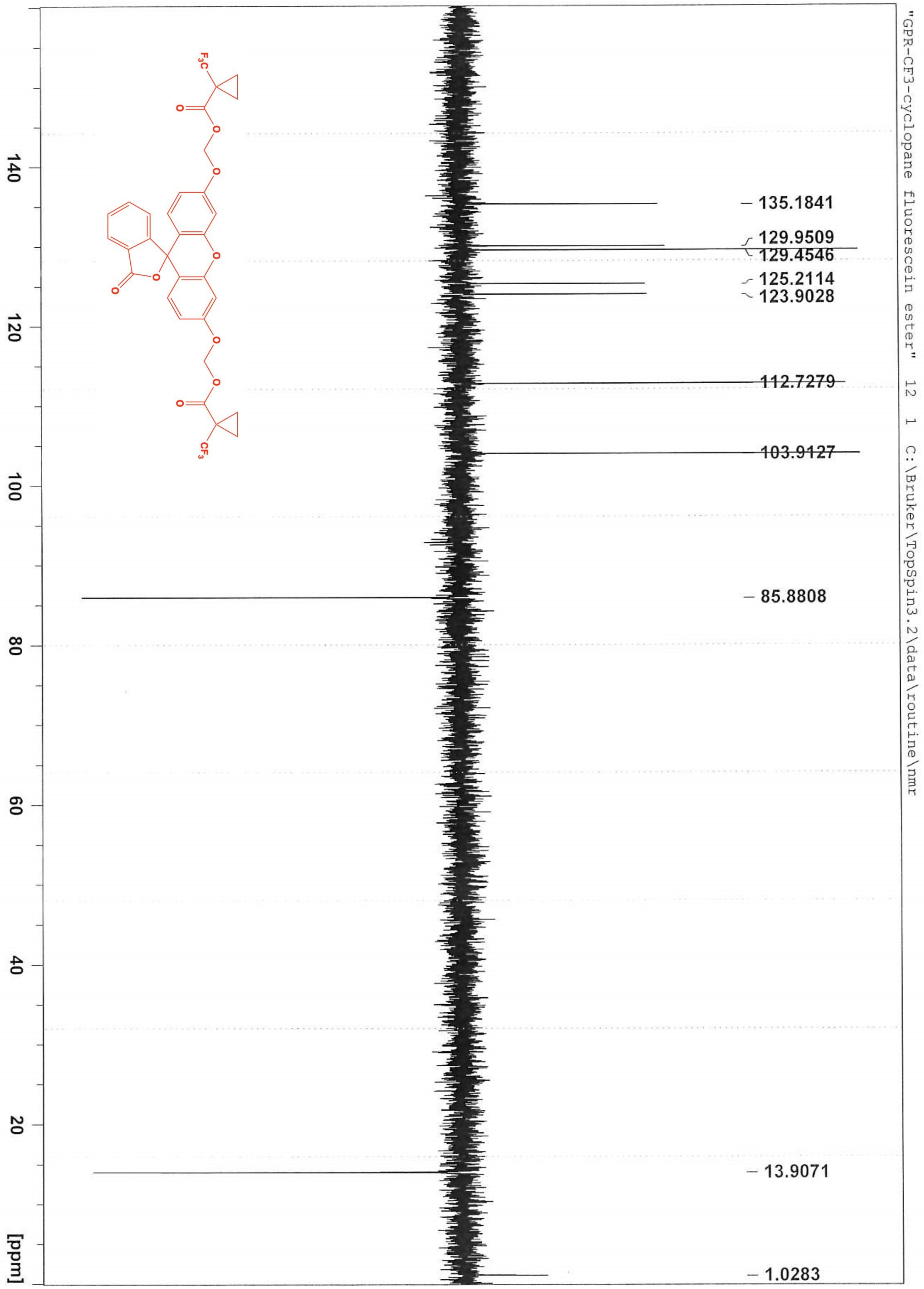
"GPR-CF3-cyclopane fluorescein ester" 12 1 C:\Bruker\TopSpin3.2\data\routine\nmr
135.1841
129.9509
129.4546
125.2114
123.9028
112.7279
103.9127
85.8808
13.9071
1.0283
140
120
100
80
60
40
20
[ppm]
F_3C
CF_3
O

Figure S134

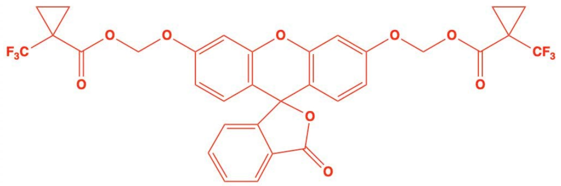

GPR-CF3-cyclopane fluorescein ester

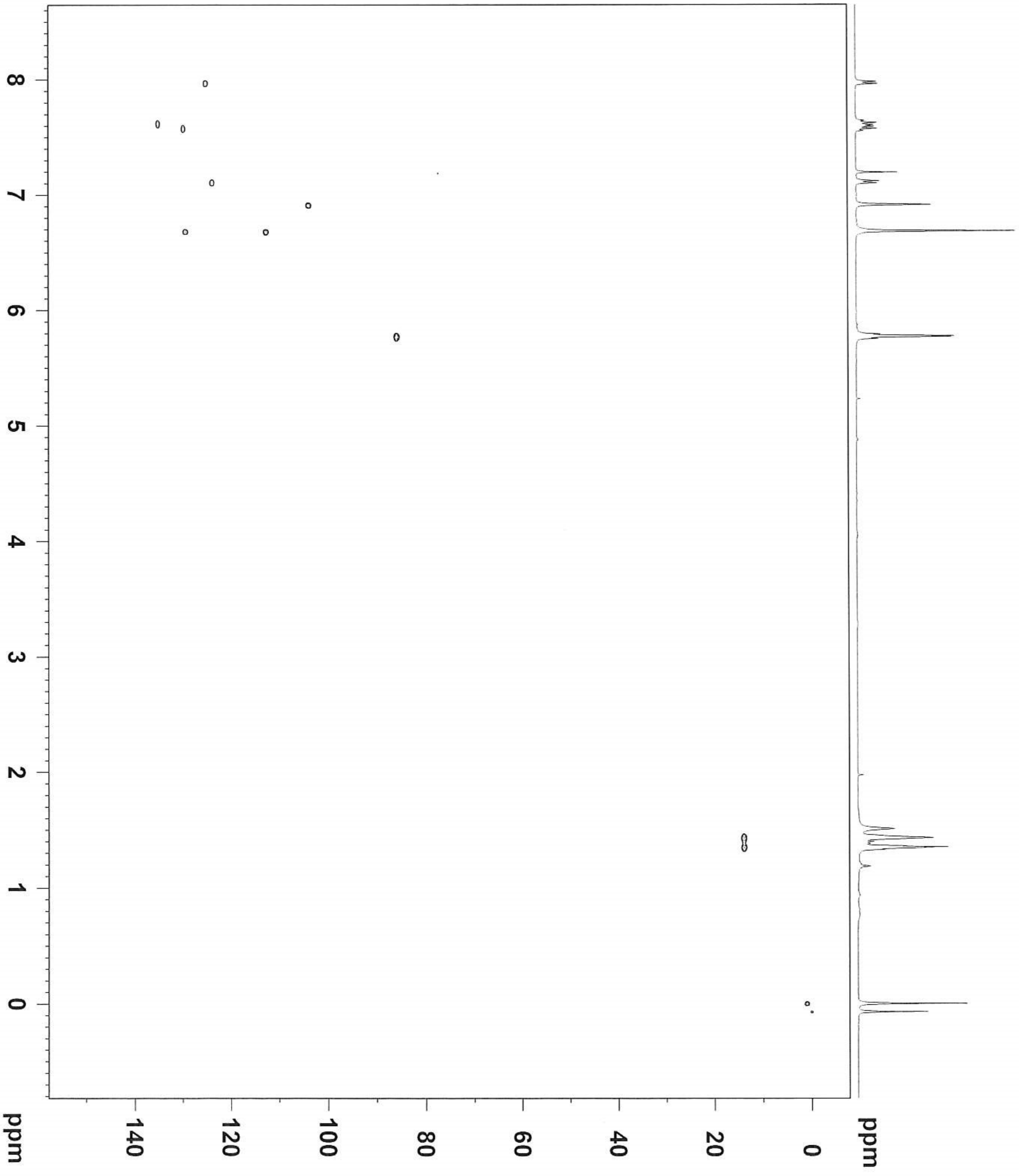

Figure S135

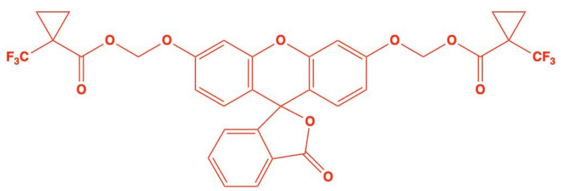
F3C
O
CF3

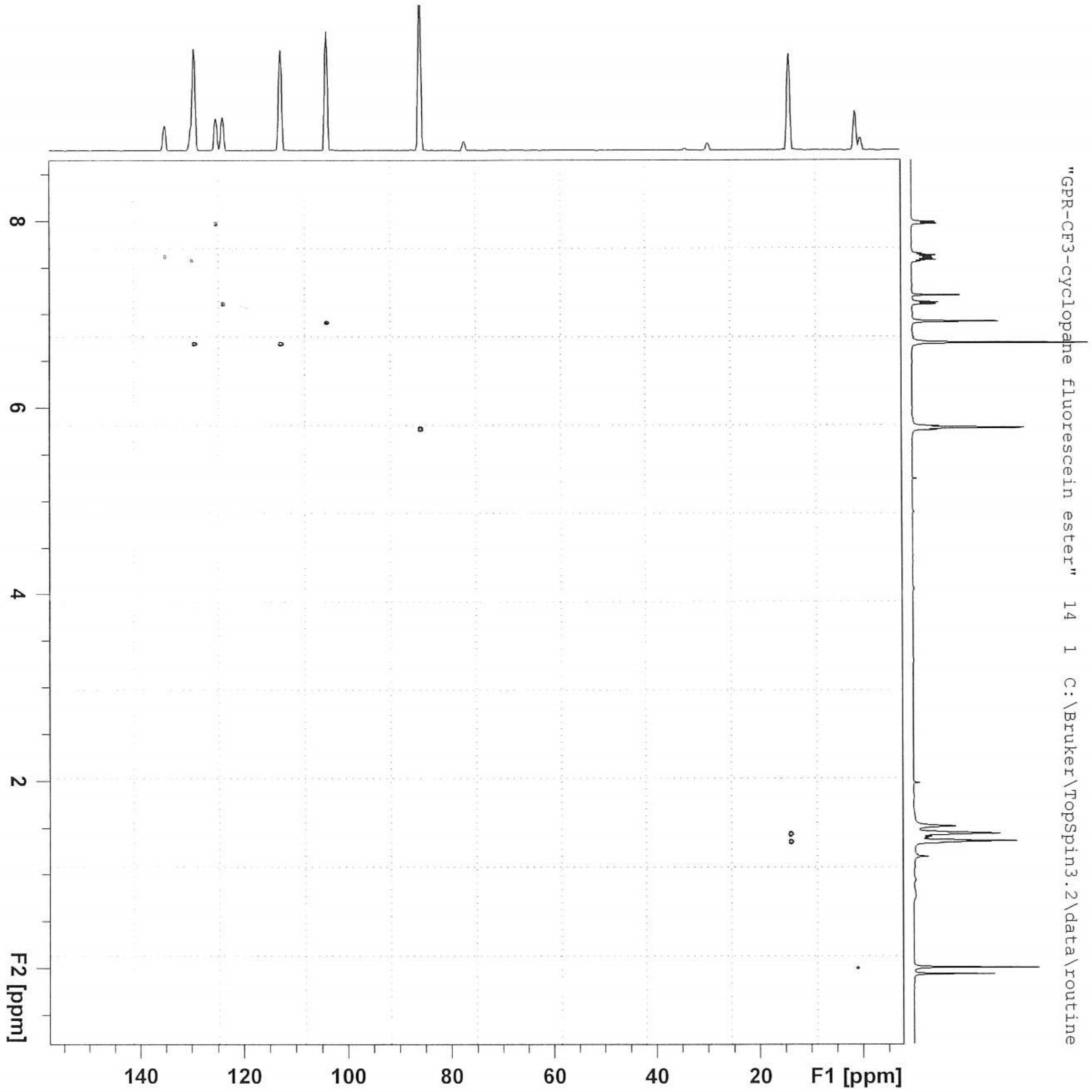
"GPR-CF3-cyclopane fluorescein ester" 14 1 C:\Bruker\TopSpin3.2\data\routine
F2 [ppm]
F1 [ppm]
140
120
100
80
60
40
20
8
6
4
2

Figure S136

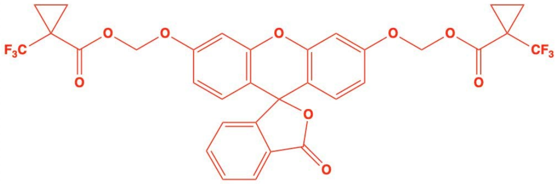

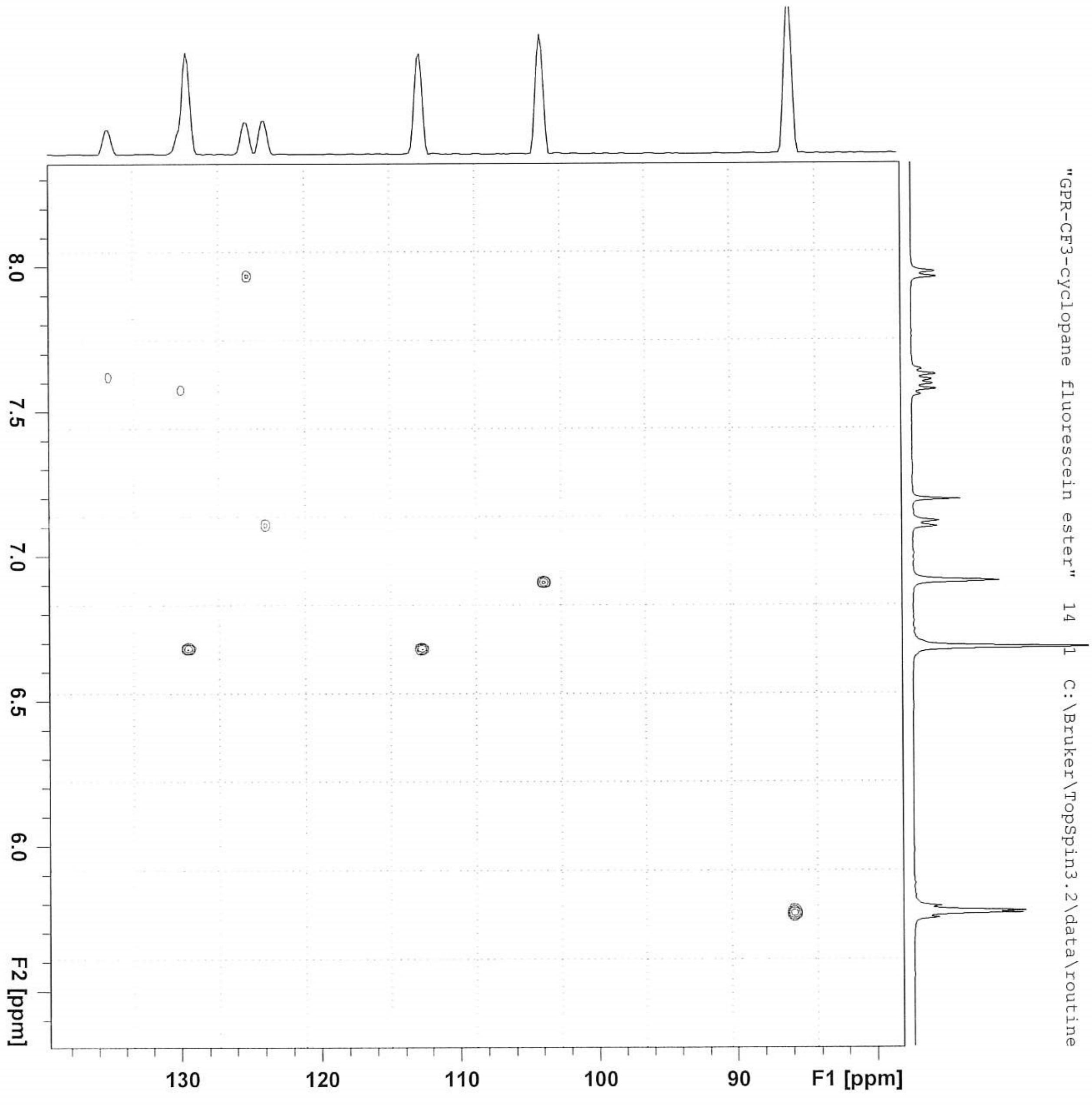
"GPR-CF3-cyclopane fluorescein ester" 14 1 C:\Bruker\TopSpin3.2\data\routine
F1 [ppm]
130
120
110
100
90
F2 [ppm]
6.0
6.5
7.0
7.5
8.0

Figure S137

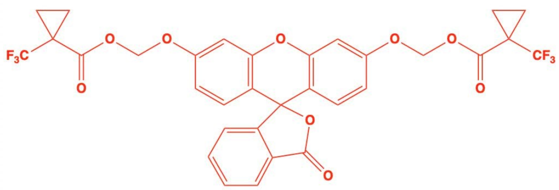
F3C
CF3
O

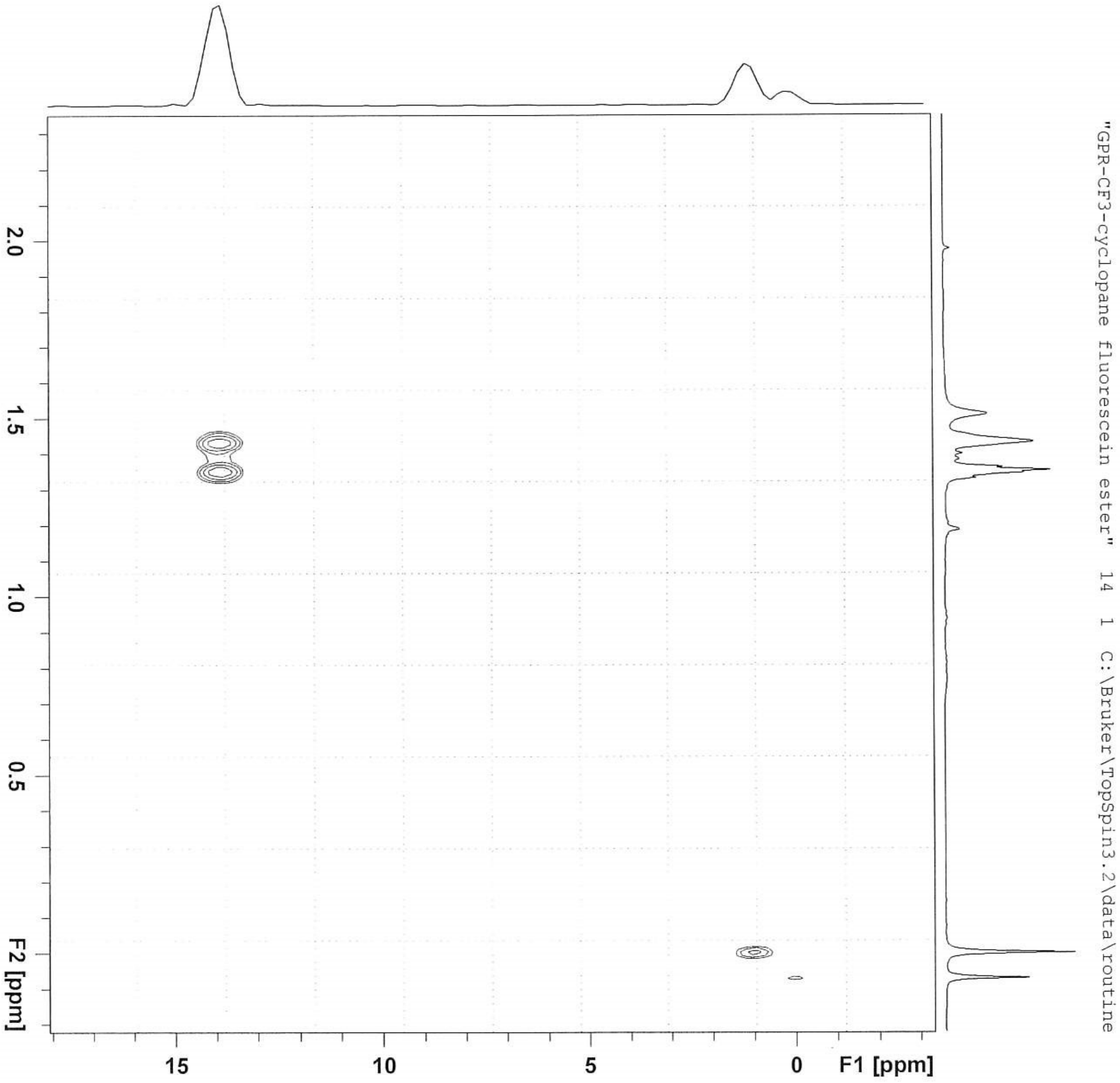
"GPR-CF3-cyclopane fluorescein ester" 14 1 C:\Bruker\TopSpin3.2\data\routine
F2 [ppm]
F1 [ppm]
2.0
1.5
1.0
0.5
15
10
5
0

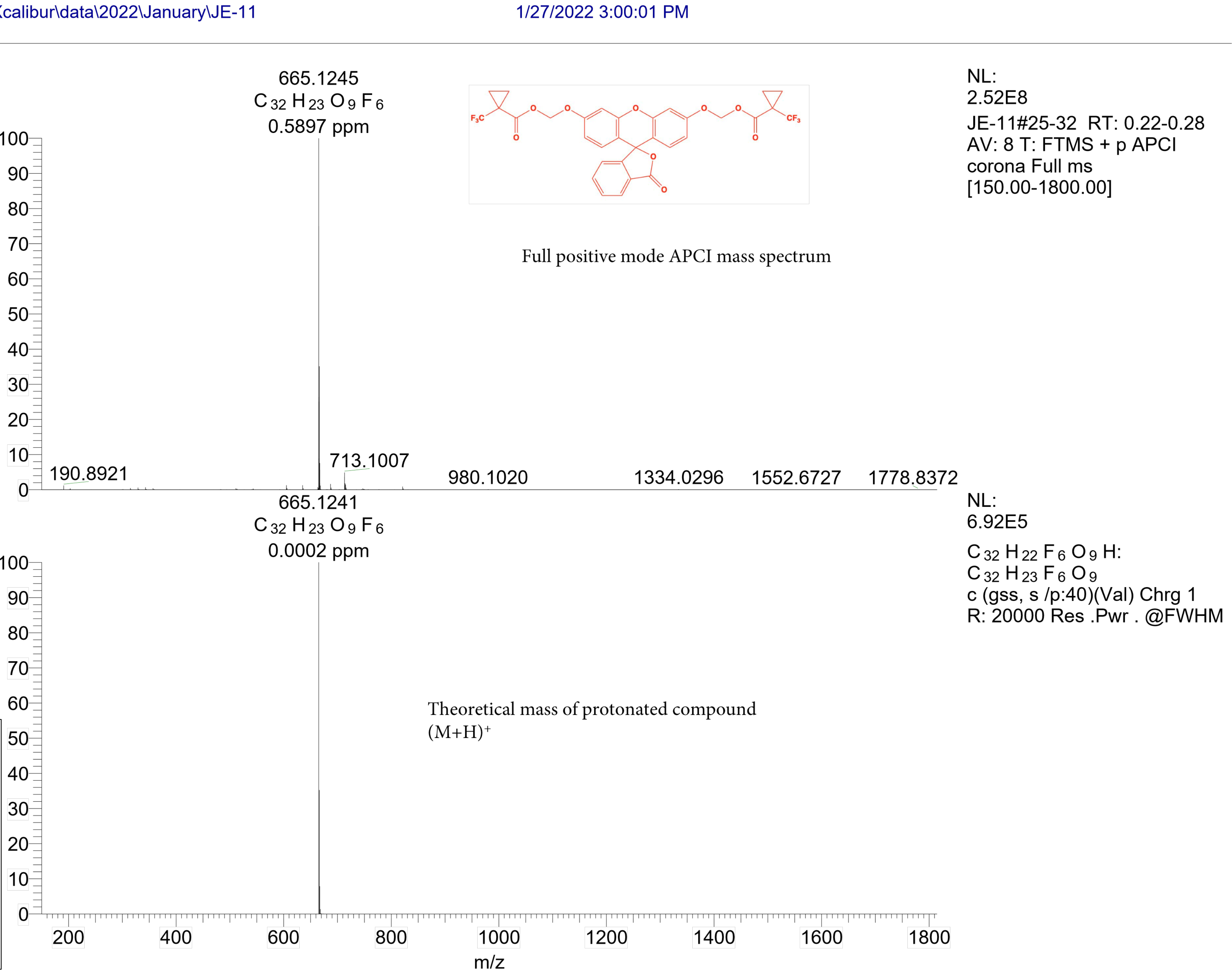
O:\Xcalibur\data\2022\January\JE-11
1/27/2022 3:00:01 PM
665.1245
C 32 H 23 O 9 F 6
0.5897 ppm
NL:
2.52E8
JE-11#25-32 RT: 0.22-0.28
AV: 8 T: FTMS + p APCI
corona Full ms
[150.00-1800.00]
Full positive mode APCI mass spectrum
Relative Abundance
190.8921
713.1007
980.1020
1334.0296
1552.6727
1778.8372
665.1241
C 32 H 23 O 9 F 6
0.0002 ppm
NL:
6.92E5
C 32 H 22 F 6 O 9 H:
C 32 H 23 F 6 O 9
c (gss, s /p:40)(Val) Chrg 1
R: 20000 Res .Pwr . @FWHM
Theoretical mass of protonated compound
(M+H)+
m/z

Figure S138

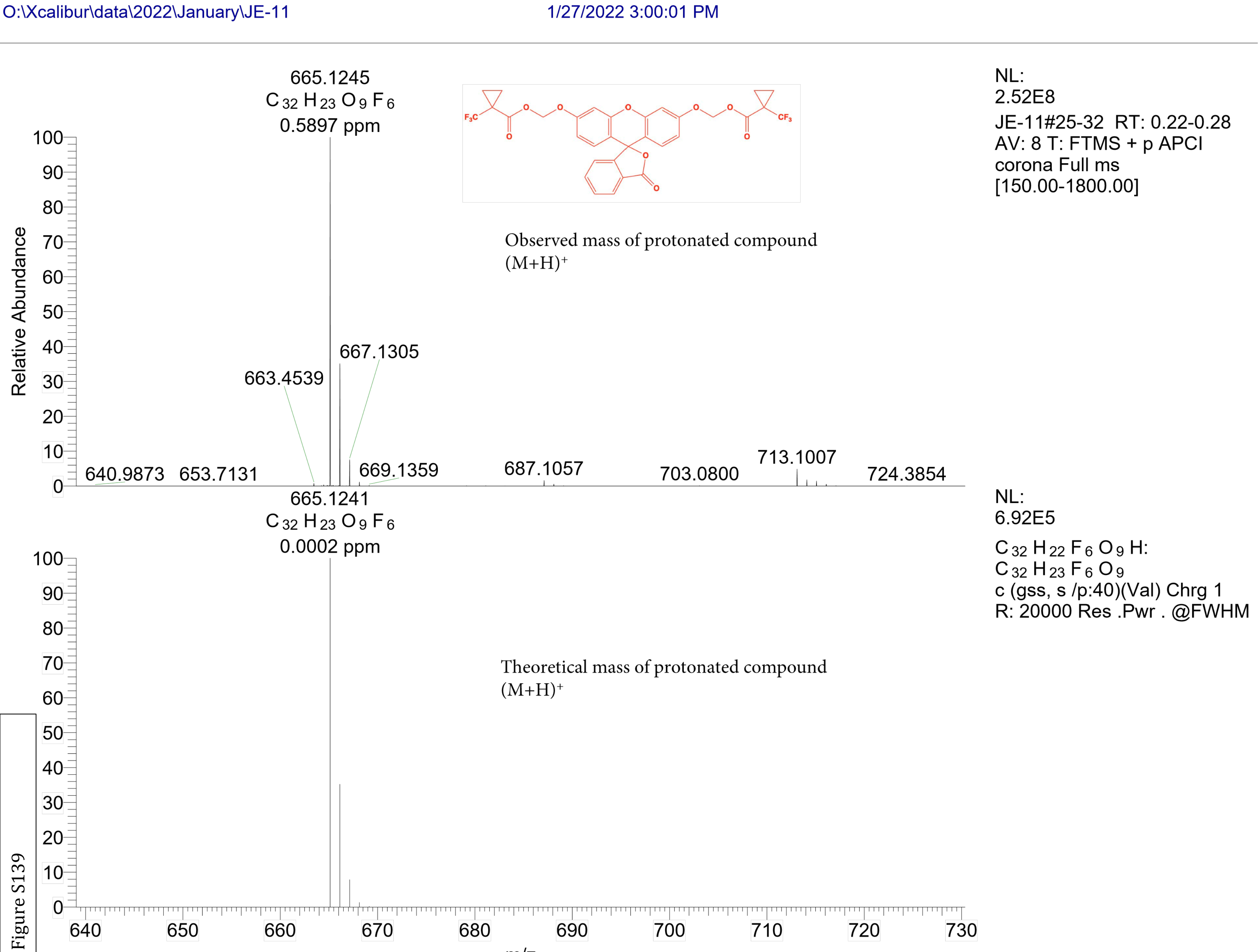
O:\Xcalibur\data\2022\January\JE-11
1/27/2022 3:00:01 PM
665.1245
C 32 H 23 O 9 F 6
0.5897 ppm
F3C
CF3
Observed mass of protonated compound
(M+H)+
NL:
2.52E8
JE-11#25-32 RT: 0.22-0.28
AV: 8 T: FTMS + p APCI
corona Full ms
[150.00-1800.00]
Relative Abundance
663.4539
667.1305
640.9873
653.7131
669.1359
687.1057
703.0800
713.1007
724.3854
665.1241
C 32 H 23 O 9 F 6
0.0002 ppm
NL:
6.92E5
C 32 H 22 F 6 O 9 H:
C 32 H 23 F 6 O 9
c (gss, s /p:40)(Val) Chrg 1
R: 20000 Res .Pwr . @FWHM
Theoretical mass of protonated compound
(M+H)+
m/z

Figure S139
